# Tell Me How Much DNA You Have and I'll Tell You What Your Sex Is: Sex Determination by Flow Cytometry of Spiderlings of *Allocosa marindia*

**DOI:** 10.1002/ece3.73453

**Published:** 2026-04-22

**Authors:** Leticia Bidegaray‐Batista, Nadia Kacevas, Federico F. Santiñaque, Magdalena Vaio, Macarena González

**Affiliations:** ^1^ Departamento de Biodiversidad y Genética, Centro de Investigación en Ciencias Ambientales Instituto de Investigaciones Biológicas Clemente Estable Montevideo Uruguay; ^2^ Departamento de Ecología y Biología Evolutiva, Centro de Investigación en Ciencias Ambientales Instituto de Investigaciones Biológicas Clemente Estable Montevideo Uruguay; ^3^ Plataforma de Citometría de Flujo y Clasificación Celular Instituto de Investigaciones Biológicas Clemente Estable Montevideo Uruguay; ^4^ Departamento de Biología Vegetal, Facultad de Agronomía Universidad de la República Montevideo Uruguay

**Keywords:** Allocosinae, diplodiploid, genome size, sex ratio, wolf spiders

## Abstract

Sex identification at early stages of development is of great interest for studies in evolutionary biology in many animals. Knowing the sex ratio, even more in offspring, allows testing hypotheses related to the cause of sex ratio biases in populations and species. Spiders born with a defined sex, mostly have an X_1_X_2_0 sex chromosome system, but it is not possible to determine their sex phenotypically until the adult or near‐adult stage. The wolf spider *Allocosa marindia* inhabits the sandy coast of Southern South America and shows sex role reversal. Laboratory and field studies suggest a strong bias in the sex ratio in favour of females in this species. Here, we analysed the 2C nuclear DNA content by flow cytometry in females and males of *A. marindia* to determine whether the difference between the sexes is enough to identify the sex of the individuals. The average 2C DNA content for females was 4.96 ± 0.036 pg and for males 4.72 ± 0.020 pg. Then, we tested the usefulness of the technique to sex *A. marindia* frozen spiderlings, in order to be able to decouple the collection time from the processing time. We analysed 59 spiderlings from four known females. Although we found greater variability in frozen samples, the difference in DNA content was enough to determine the sex of 54 frozen spiderlings (43 females and 11 males). Our results show a promising technique for sexing hatchlings of diplodiploid arthropods. In future studies, we will seek to sex spiderlings from a larger number of mothers to understand the causes of female bias in this species.

## Graphical abstract

The manuscript addresses the sex determination of spiderlings of the wolf spider *Allocosa marindia* using flow cytometry. Spiders are born with a defined sex, but it is not possible to determine their sex phenotypically until the adult or near‐adult stage. Sex identification at early stages of development is of great interest for studies in evolutionary biology and ecology in many animals. We analysed the 2C nuclear DNA content by flow cytometry of *A. marindia* spiderlings, and we could determine the sex of 54 spiderlings (43 females and 11 males), which is consistent with the previously recorded female‐biased sex ratio for the species. Our results show a promising technique for sexing hatchlings of diplodiploid arthropods, opening up a vast range of new questions, as well as answers to many existing ones.

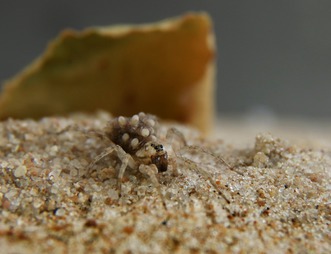

## RESUMEN

La identificación del sexo en las primeras etapas del desarrollo es de gran interés para estudios de biología evolutiva en muchos animales. Conocer la proporción de sexos, especialmente en la descendencia, permite probar hipótesis relacionadas con la causa de los sesgos en la proporción de sexos en poblaciones y especies. Las arañas nacen con un sexo definido, en su mayoría tienen un sistema de cromosomas sexuales X_1_X_2_0, pero no es posible determinar su sexo fenotípicamente hasta la etapa adulta o casi adulta. La araña lobo *Allocosa marindia* habita arenales costeros del sur de Sudamérica y muestra inversión de roles sexuales. Estudios de laboratorio y de campo sugieren un fuerte sesgo en la proporción de sexos a favor de las hembras en esta especie. Aquí, analizamos el contenido nuclear 2C de ADN por citometría de flujo en hembras y machos de *A. marindia* para determinar si la diferencia entre los sexos es suficiente para identificar el sexo de los individuos. El contenido de ADN 2C promedio para las hembras fue de 4,96 ± 0,036 pg y para los machos de 4,72 ± 0,020 pg. A continuación, probamos la utilidad de la técnica para determinar el sexo de crías congeladas de *A. marindia*, con el fin de separar el tiempo de recolección del tiempo de procesamiento. Analizamos 59 crías de cuatro hembras conocidas. Si bien encontramos mayor variabilidad en las muestras congeladas, la diferencia en el contenido de ADN fue suficiente para determinar el sexo de 54 crías congeladas (43 hembras y 11 machos). Nuestros resultados muestran una técnica prometedora para determinar el sexo de crías de artrópodos diplodiploides. En estudios futuros, buscaremos determinar el sexo de crías de un mayor número de madres para comprender las causas del sesgo hacia las hembras en esta especie.

## 1 Introduction

The identification of sex in the early stages of development of many animals is of great interest for studies in evolutionary biology. In most sexually reproducing species, the primary sex ratio is close to 1:1, an equal number of males and females born in a given population (Fisher 1999; Trivers and Willard 1973). However, deviations in sex ratio have been reported in different animal taxa, including mammals, birds, and arthropods, among others (Clutton‐Brock and Iason 1986; Gowaty 1993; Hamilton 1967; Hardy 1992; Jiggins et al. 1998).

Sex ratio can vary due to different causes that can act at different stages in the ontogeny of individuals, either before, during or after fertilisation, or even after birth (Engelstädter and Hurst 2009; Majerus 2003; Werren et al. 2008; Werren and Beukeboom 1998). These variations can imply changes in sexual strategies of species or populations (Elias et al. 2011; Hamilton 1967). For example, a female‐biased sex ratio would make males a scarce resource, which could result in males being more selective in choosing a mate, or in pairs forming through mutual choice, leading to sexual behaviours that differ from the typical expected ones (Aisenberg 2014; Bonduriansky 2001; Jiggins et al. 2000). That is why correct sex determination before adulthood makes it possible to test hypotheses related to the cause of biases in the sex ratio of populations and species (Kappeler et al. 2023). In some groups, this early determination turns out to be very difficult using the classic method of morphological characteristics (Wilkinson et al. 2016).

In spiders, sex cannot usually be determined until individuals reach adulthood or advanced immature stages, when sexual structures become externally distinguishable (Cordellier et al. 2020; Foelix 2011). Spiders are diplodiploids (both sexes are diploids) and mostly have an X_1_X_2_0 sex chromosome system (SCS), males have one set of X_1_X_2_ chromosomes and females have two sets X_1_X_2_X_1_X_2_ (Araujo et al. 2012). This SCS is the most common in wolf spiders according to the spider cytogenetic database (Douglas et al. 2024). This difference in the number of sex chromosomes between sexes also implies a difference in the DNA content. Flow cytometry has multiple applications, including determining the nuclear DNA content of cells from different taxa (Adan et al. 2017). This technique has been used in different organisms to determine genome sizes, including insects, ticks and spiders, among other arthropods (Hare and Johnston 2011; Král et al. 2019). It has also been applied for sex determination in various eukaryotic organisms that are monomorphic or in which males and females are difficult to distinguish during early developmental stages. For this purpose, the technique has been applied to a wide range of organisms, such as insects, birds, and even plants (Boivin and Candau 2007; Doležel and Göhde 1995; Loureiro et al. 2014; Tiersch and Mumme 1993). Additionally, flow cytometry has been used to determine brood sex ratios in haplodiploid insects (Aron et al. 2003), and for sexing sperm in spiders (Vanthournout, Deswarte, et al. 2014; Vanthournout et al. 2018). Its application relies on the presence of differences in DNA content between sexes, typically resulting from polymorphisms in the number or size of sex chromosomes.

**FIGURE 1:**
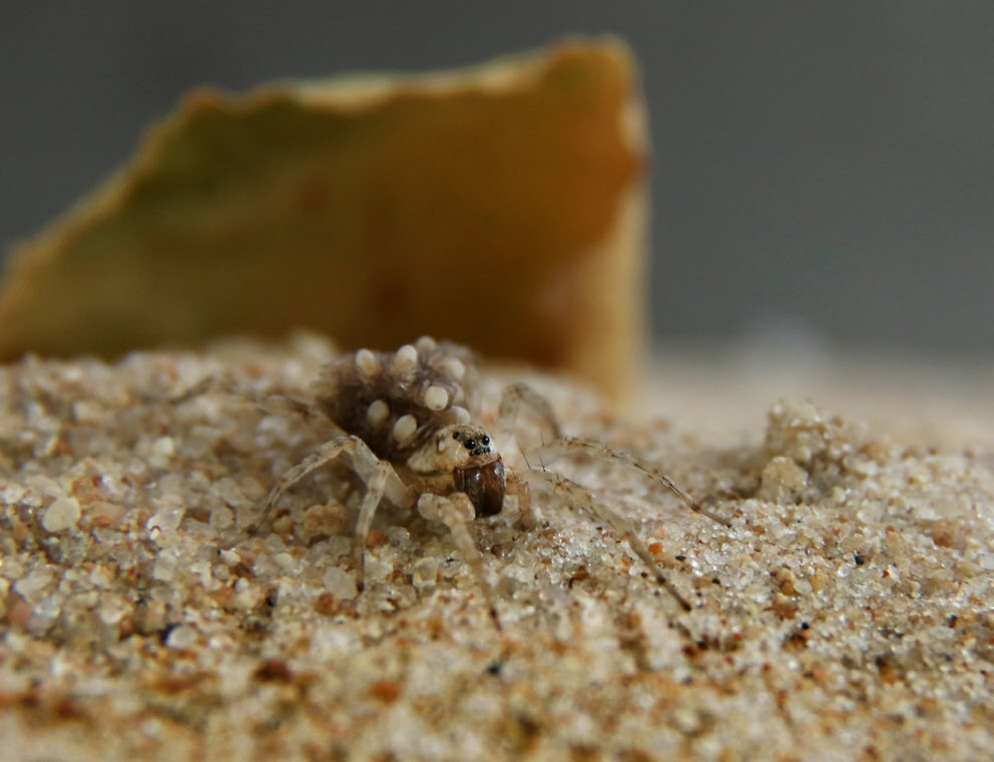
Female of the wolf spider *Allocosa marindia* carrying her spiderlings on her back. Photograph: Marcelo Casacuberta.

The wolf spider *Allocosa marindia* (Simó et al. 2017) (Araneae: Lycosidae) shows a reversal in the typical sex roles and in the sexual size dimorphism expected in spiders (Aisenberg and Costa 2008; Aisenberg 2014). In this species, females are the mobile and courting sex, and males are larger than females (Aisenberg and Costa 2008; Aisenberg et al. 2023). They inhabit the sandy coast of rivers, lakes, and seashores of Southern South America (Figure 1) (Costa 1995; Costa et al. 2006; Simó et al. 2017), where they construct burrows in the sand and shelter during daylight and winter, becoming active for foraging and searching for mates during summer nights (Aisenberg 2014; Bidegaray‐Batista et al. 2017). Mating occurs inside male burrows and, after that, males donate their burrow to females (Aisenberg and Costa 2008; Aisenberg 2014). Females lay their egg‐sacs in the male burrows and leave burrows at the time of dispersing their hatchlings (Aisenberg and Costa 2008; Aisenberg 2014). As is typical for wolf spiders, after the spiderlings emerge from the egg‐sac, they climb on their mother's back and remain there until the time of dispersal (Foelix 2011).

Laboratory and field studies suggest a strong bias in sex ratio in favour of females in *A. marindia* (Aisenberg and Costa 2008; Aisenberg et al. 2011; Cavassa et al. 2022). This bias was reported in studies based on field sampling using pitfall traps or hand capture collections (Aisenberg and Costa 2008; Aisenberg et al. 2011; Cavassa et al. 2022; Costa et al. 2006). Under laboratory conditions, after maintaining a female with an egg‐sac until the spiderlings emerged and reached adulthood, it was confirmed that none were male (Aisenberg and Costa 2008). On the other hand, the presence of the endosymbiont bacteria *Wolbachia*, known to cause a sex ratio bias towards females in many arthropods (Werren 1997; Werren et al. 2008), has been detected in this species (Lerette et al. 2015). However, this evidence is still insufficient to support a cause‐effect relationship between the presence of *Wolbachia* and female bias in *A. marindia* . Further studies are needed to test this hypothesis (Lerette et al. 2015).

Here we set out to test if the difference in DNA content between sexes in *A. marindia* , which has an X_1_X_2_0 SCS (Postiglioni and Postiglioni 2008), is enough to be measurable by flow cytometry, and if it is feasible to sex their hatchlings. For that purpose, we first determined the nuclear DNA content of adult spiders, and secondly, we determined the nuclear DNA content of the hatchlings of four mothers of *A. marindia* . This species has a limited sexual period of 3 months during the summer, and its presence on beaches is not uniform along the coast (Bidegaray‐Batista et al. 2017; Costa et al. 2006), something that influences its inclusion in the list of priority arachnids for conservation in Uruguay (Ghione et al. 2017). Furthermore, it is a particularly difficult species to rear (which takes an average of 323 days to reach adulthood) and reproduce under laboratory conditions (Aisenberg and Costa 2008). In species with any of these characteristics, sexing non‐adults with an accurate method becomes especially valuable. That is why achieving a reliable detection of differences between the nuclear DNA content of female and male offspring would allow this technique to be considered for sexing and testing hypotheses regarding sex ratio biases in this species and other diplodiploid arthropods.

## 2 Materials and Methods

We collected twelve females, four of them carrying spiderlings, and ten males of *A. marindia* at San José de Carrasco beach, Canelones department, Uruguay (34°51′06.06″ S, 55°58′46.71″ W). Spiders were collected at night using headlights during the summer of the Southern Hemisphere, the season of highest surface activity reported for the species (Aisenberg et al. 2023; Bidegaray‐Batista et al. 2017). After capture, in the laboratory, spiders were anaesthetised by cooling and euthanised by mechanical destruction of the central nervous system using a fine needle. Legs were dissected and placed individually into sterile tubes. Fresh tissue was processed immediately, whereas tissue intended for frozen analyses was stored at −80°C until processing.

In order to test if the difference in nuclear DNA content (2C value) between females and males was sufficient to determine the sex of individuals by flow cytometry, we first analysed fresh tissue from three females and three males. The analyses were carried out on different days, with one male and one female being measured on each day. After that, to determine the sex of spider hatchlings, we analysed frozen tissue (previously stored at −80°C according to Král et al. 2019) from nine females, their spiderlings (from four of these females), and seven males. Measurements of each mother and her spiderlings were performed on different days, as well as of males and the remaining females. Adult leg tissues and spiderlings were frozen separately at −80°C and used only once, thus avoiding freezing and thawing. The use of frozen tissue enables storage of both mothers and offspring, thereby separating field collection from cytometer measurement when continuous access to the equipment is not possible.

We used Otto buffers for nuclei isolation that were prepared according to Doležel et al. (2007). Briefly, Otto I solution: 0.1 M citric acid. 0.5% Tween 20 (Sigma‐Aldrich. cat. no. P2287) (5°C), Otto II solution: 0.4 M Na_2_HPO_4_·12H_2_O (room temperature ~20°C). Samples were prepared and measured one at a time. In all cases we chopped the spider tissue (males: leg I, female: leg I without femur, spiderlings: prosoma with legs) in a Petri dish, together with a fresh plant leaf of tomato *Lycopersicon esculentum* ‘cultivar Stupické’ using a sharp razor blade in cold Otto I buffer (500 μL for adults, 300 μL for spiderlings). *Lycopersicon esculentum* was used as DNA reference standard, which has a known 2C value of 1.96 pg (Doležel et al. 1992). The use of plant DNA standards to measure DNA content of spiders was shown to be successful in previous studies (Král et al. 2019; Sheffer et al. 2022). Prosome width, a measure considered to be representative of total body size in spiders (Moya‐Laraño and Cabeza 2003) was 0.60 ± 0.09 mm for spiderlings, and tomato leaf area was 31.82 ± 6.06 mm^2^ (average ± SD, respectively). After chopping the tissues, the suspension was filtered through a 50 μm nylon mesh and an equal volume of Otto II buffer was added to the filtrate. The sample was then mixed using a vortex. A volume of 475 μL of the suspension was transferred to another tube, and 25 μL of propidium iodide (PI) at a final concentration of 50 μg/mL was added and mixed by vortex to stain the nuclei. The sample was incubated at room temperature in darkness for 40 min.

After incubation, DNA content measures were performed with the flow cytometer and cell sorter MoFlo Astrios EQ (Beckman Coulter, USA). Each measurement was done using a 488 nm laser and a 100 μm nozzle (25 psi). Flow cytometer calibration and quality control were carried out using 3.0 μm Ultra Rainbow Fluorescent Particles (Spherotech, USA). Fluorescence emitted from PI was detected with a 620/29 bandpass filter. The following parameters were analysed: forward scatter (FSC‐Height), side scatter (SSC‐Height), 620/29‐Area (PI fluorescence intensity), and 620/29‐Width. Doublets were excluded using dot plots of 620/29 pulse‐area versus 620/29 pulse‐width. Chicken Erythrocyte Nuclei (CEN, DNA QC particles, Becton Dickinson, USA) were used to check instrument linearity (±0.05). Flow cytometry data was acquired and analysed with Summit software (Beckman Coulter, USA). The estimation of nuclear DNA contents (2C) was calculated by the equation: sample peak mean/standard peak mean × 2C DNA content of standard (pg).

In order to avoid bias during the analyses of samples and the spiderling sex assignment, the researchers who processed the samples in the lab were different from those who analysed them in the flow cytometer and also analysed the data, and they did not know the identity of the samples. To determine the sex of spiderlings, DNA content values from frozen tissue samples of reference females and males were used to define female and male ranges based on the minimum and maximum values. These ranges were then used to assign the sex of spiderlings. Individuals with DNA content values outside these ranges were classified as undetermined.

## 3 Results

**FIGURE 2:**
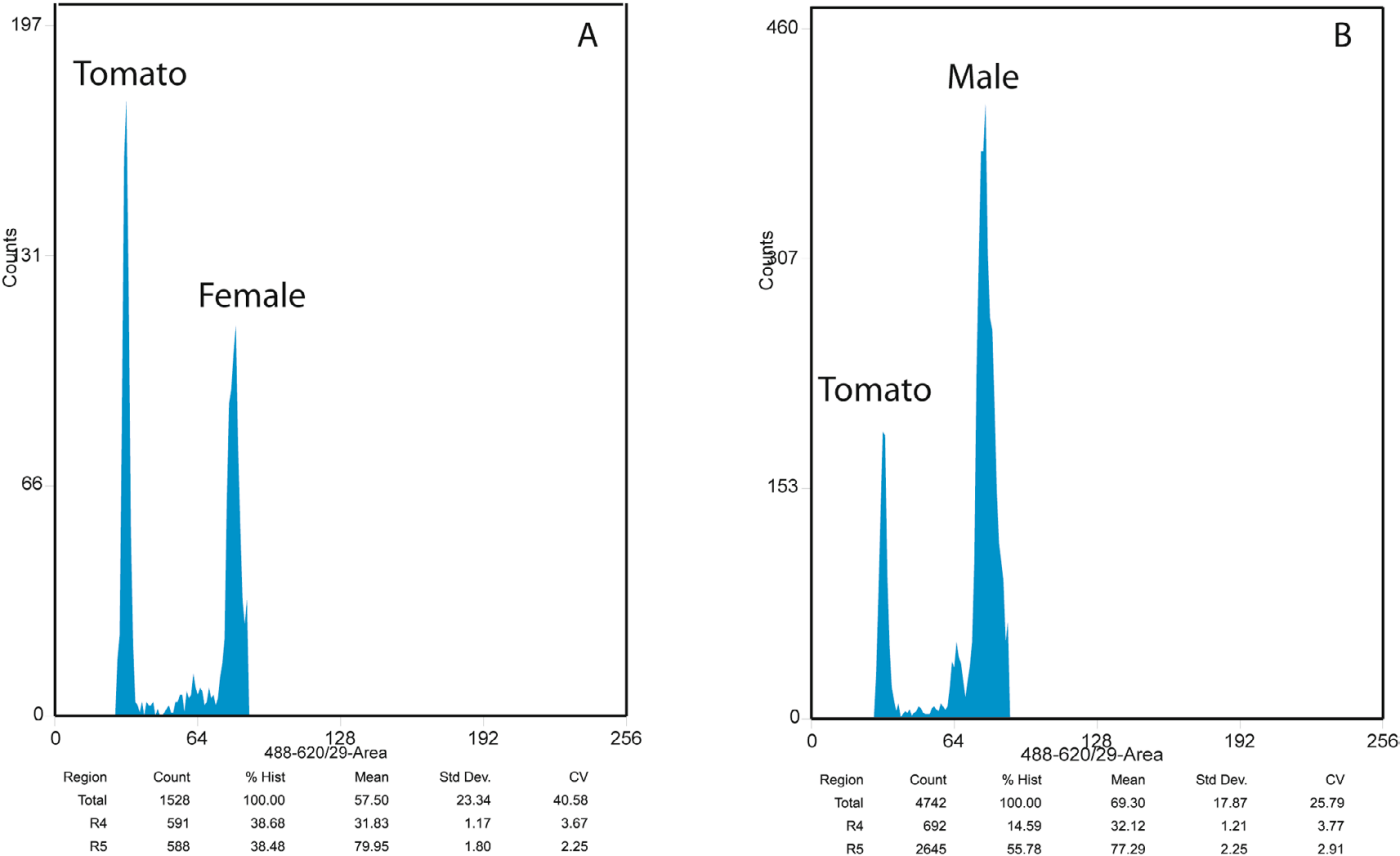
Flow cytometric DNA histograms obtained from fresh tissue of adults of *Allocosa marindia* (A: Female, B: Male) and *Lycopersicon esculentum* , tomato (2C = 1.96 pg) used as plant DNA reference standard. Each histogram shows the nuclear DNA content profile of a single sample. The first high peak corresponds to the nuclear DNA content of tomato (nuclei in G1 phase), and the second high peak corresponds to the nuclear DNA content of spiders (G1 nuclei). Below each histogram the name of the analysed region is shown, indicating where the gates were placed to calculate the mean measurement for nuclear DNA content determination based on tomato DNA content. Number of nuclei under each peak, and the coefficient of variation (CV), are also shown. Note the low CV of each peak indicating precise measurements.

**FIGURE 3:**
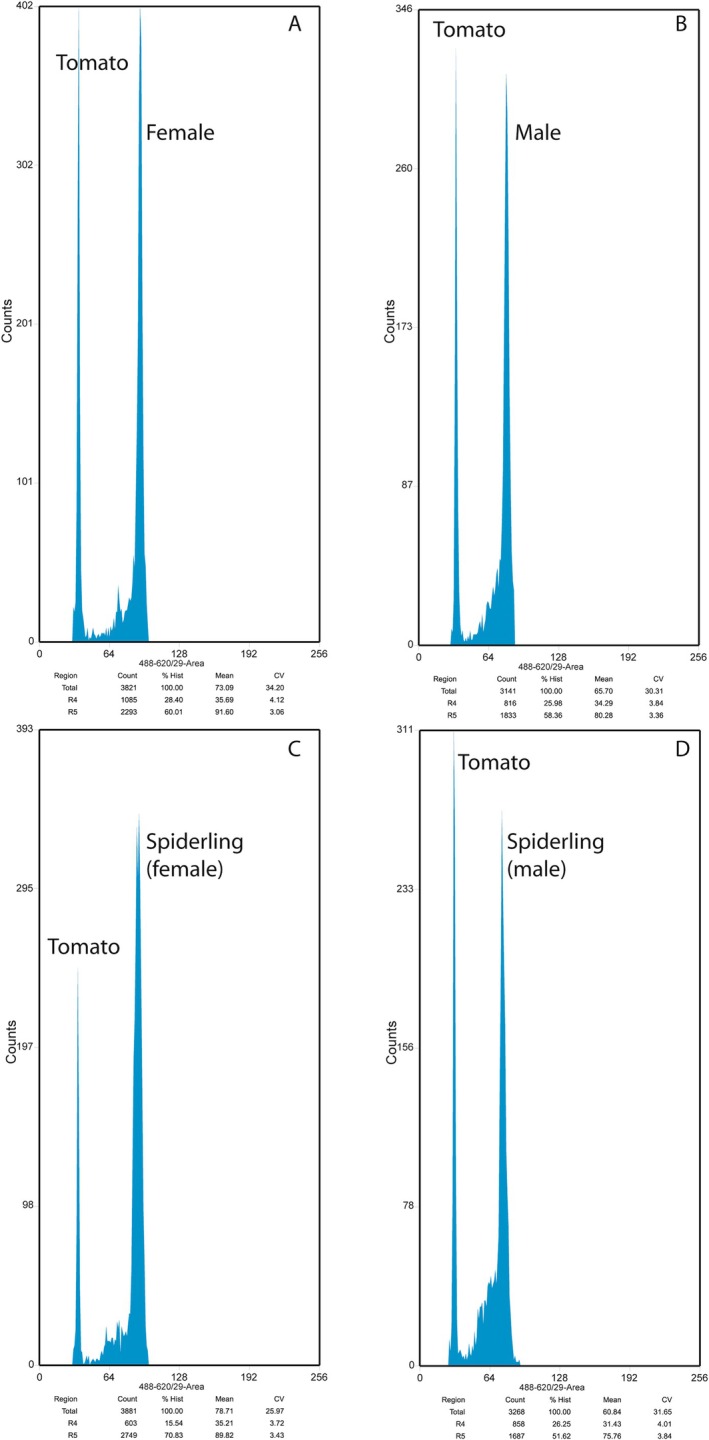
Flow cytometric DNA histogram of frozen tissue of adults and spiderlings of *Allocosa marindia* (A: Female, B: Male, C: Spiderling determined as female, D: Spiderling determined as male) with the plant DNA reference standard *Lycopersicon esculentum* , tomato (2C = 1.96 pg). The first high peak corresponds to the nuclear DNA content of tomato (nuclei in G1 phase), and the second high peak corresponds to the nuclear DNA content of spiders (G1 nuclei). Each histogram shows the nuclear DNA content profile of a single sample. Note the low CV of each peak.

DNA content of fresh tissue of three females and three males was measured in *A. marindia* (Table 1, Figure 2A,B). The mean DNA content (2C value) was 4.96 ± 0.036 pg for females and 4.72 ± 0.020 pg for males. The difference in DNA content between sexes was 0.24 pg (4.84%), exceeding the expected measurement errors (±0.05 pg). The mean 2C value obtained from frozen tissue was 5.08 ± 0.172 pg (min. = 4.93 pg, max. = 5.57 pg) for females and 4.67 ± 0.079 pg (min. = 4.52 pg, max. = 4.84 pg) for males (Table 1). Although the number of nuclei detected was relatively small, flow cytometry analysis of both fresh and frozen spider tissue produced low data variability and high‐quality DNA content histograms (Figures 2A,B and 3A,B).

**TABLE 1:** DNA content measurements from fresh and frozen tissue of females and males of *Allocosa marindia* .

Females		Males	
Individual codes	2C values (pg)	Individual codes	2C values (pg)
**Fresh tissue**			
	4.99		4.72
	4.92		4.70
	4.97		4.74
**Frozen tissue**			
IIBCELB793	5.02	IIBCELB787	4.66
IIBCELB794	5.04	IIBCELB788	4.64
IIBCELB795	5.02	IIBCELB789	4.61
IIBCELB796	5.04	IIBCELB790	4.70
IIBCELB797	5.00	IIBCELB791	4.62
IIBCELB801	5.01	IIBCELB798	4.65
IIBCELB802	4.98	IIBCELB799	4.57
IIBCELB793	5.03	IIBCELB787	4.52
IIBCELB794	4.95	IIBCELB788	4.59
IIBCELB795	4.96	IIBCELB789	4.57
IIBCELB796	4.94	IIBCELB790	4.65
IIBCELB797	5.00	IIBCELB791	4.67
IIBCELB801	5.09	IIBCELB798	4.63
IIBCELB802	5.02	IIBCELB799	4.67
IIBCELB793	5.02	IIBCELB787	4.77
IIBCELB794	5.03	IIBCELB788	4.74
IIBCELB795	4.98	IIBCELB789	4.84
IIBCELB796	5.13	IIBCELB790	4.75
IIBCELB797	4.93	IIBCELB791	4.76
IIBCELB801	5.15	IIBCELB798	4.74
IIBCELB802	5.09	IIBCELB799	4.72
AmC3SJC	5.57		
AmC6SJC	5.45		
AmC6SJC	5.48		

*Note:* Values are the content of diploid cells (2C values) in picograms (pg).

A total of 59 spiderlings of *A. marindia* were analysed (Table 2, Figure 3C,D). The time required for sexing all the spiderlings was 13 h of work (including the lab processing and cytometer measurement). We could determine the sex of 54 spiderlings, of which 43 were females and 11 were males, while the sex of five spiderlings could not be assigned (undetermined). DNA content values (2C) from frozen tissues were higher than those from fresh tissues and showed greater variation, even though high‐quality DNA content histograms were still obtained.

**TABLE 2:** DNA content measurements from frozen tissue of *Allocosa marindia* spiderlings.

Spiderlings with their mother's code	2C values (pg)	Sex determination
Spiderling of IIBCELB793	5.13	f
Spiderling of IIBCELB793	4.61	m
Spiderling of IIBCELB793	5.11	f
Spiderling of IIBCELB793	4.76	m
Spiderling of IIBCELB793	5.11	f
Spiderling of IIBCELB793	4.83	m
Spiderling of IIBCELB793	5.16	f
Spiderling of IIBCELB793	4.77	m
Spiderling of IIBCELB793	5.15	f
Spiderling of IIBCELB793	4.80	m
Spiderling of IIBCELB793	4.78	m
Spiderling of IIBCELB793	5.15	f
Spiderling of IIBCELB793	4.79	m
Spiderling of IIBCELB793	5.25	f
Spiderling of IIBCELB794	4.72	m
Spiderling of IIBCELB794	5.21	f
Spiderling of IIBCELB794	4.85	und.
Spiderling of IIBCELB794	4.82	m
Spiderling of IIBCELB794	5.25	f
Spiderling of IIBCELB794	5.19	f
Spiderling of IIBCELB794	4.88	und.
Spiderling of IIBCELB794	4.85	und.
Spiderling of IIBCELB794	5.34	f
Spiderling of IIBCELB794	4.95	f
Spiderling of IIBCELB794	4.95	f
Spiderling of IIBCELB794	4.95	f
Spiderling of IIBCELB794	5.52	f
Spiderling of IIBCELB794	4.95	f
Spiderling of IIBCELB794	5.41	f
Spiderling of IIBCELB795	5.22	f
Spiderling of IIBCELB795	5.30	f
Spiderling of IIBCELB795	4.70	m
Spiderling of IIBCELB795	5.25	f
Spiderling of IIBCELB795	4.81	m
Spiderling of IIBCELB795	5.36	f
Spiderling of IIBCELB795	4.86	und.
Spiderling of IIBCELB795	4.85	und.
Spiderling of IIBCELB795	5.29	f
Spiderling of IIBCELB795	5.34	f
Spiderling of IIBCELB795	5.34	f
Spiderling of IIBCELB795	5.00	f
Spiderling of IIBCELB795	5.41	f
Spiderling of IIBCELB795	5.46	f
Spiderling of IIBCELB795	5.20	f
Spiderling of mother AmC3	5.48	f
Spiderling of mother AmC3	5.49	f
Spiderling of mother AmC3	5.01	f
Spiderling of mother AmC3	4.99	f
Spiderling of mother AmC3	5.18	f
Spiderling of mother AmC3	5.06	f
Spiderling of mother AmC3	5.49	f
Spiderling of mother AmC3	5.66	f
Spiderling of mother AmC3	5.37	f
Spiderling of mother AmC3	5.35	f
Spiderling of mother AmC3	5.03	f
Spiderling of mother AmC3	5.38	f
Spiderling of mother AmC3	5.06	f
Spiderling of mother AmC3	5.52	f
Spiderling of mother AmC3	5.19	f

*Note:* Values are the nuclear DNA content of somatic diploid cells (2C values) in picograms (pg). Spiderlings sex determinations are denoted with f = female, m = male, and und. = undetermined.

## 4 Discussion

This study provides the first empirical evidence that flow cytometry can be used for sex determination in spiders. We also report, for the first time, the 2C nuclear DNA content of females and males of *A. marindia* , showing that the difference between sexes is sufficient to identify the sex of spiderlings with this rapid technique.

With this approach, we can avoid the time‐consuming process of rearing individuals to adulthood and the concomitant challenges in spiders (such as the associated mortality rates) (Aisenberg and Costa 2008), as well as the need of karyotyping spiderlings for sex identification (Avilés and Maddison 1991; Mahmoudi et al. 2008; Sheffer et al. 2022). Furthermore, here we found that 80% of the spiderlings sexed from four *A. marindia* mothers were female. Our result is consistent with the female‐biased sex ratio previously recorded for the species, based on the rearing of offspring from an egg sac until adulthood (Aisenberg and Costa 2008).

We fine‐tuned the flow cytometry technique for sexing spiderlings by measuring nuclear DNA content and outlined key considerations that should be taken into account for accurate sex identification. We highlight the importance of including a large number of adult individuals of each sex, due to the variation observed in DNA content measurements from frozen tissue. We also confirmed that the difference in nuclear DNA content between spider sexes is subtle. This contrast is particularly evident when compared to haplodiploid species, such as ants, where females have approximately twice the amount of nuclear DNA as males (Aron et al. 2003). That is why we emphasise the importance of methodological precision. Avoiding freezing and thawing tissues more than once and maintaining them at low temperature during sample processing are essential to achieving reliable measurements.

Variation in DNA content estimation between frozen and fresh samples could be related to differences in chromatin conformation. Propidium iodide, an intercalating fluorochrome, is sensitive to chromatin structure. This means that any alterations in chromatin condensation (caused by tissue type or by physical or chemical treatments) or the presence of cellular compounds that modify dye accessibility may influence the DNA content estimation (Johnston et al. 2019; Loureiro et al. 2021).

Female biased sex ratios have been extensively studied and reported in species with haplodiploid sex determinations such as Hymenoptera, which can control sex allocation (Gardner and Ross 2013; Hardy 1992). However, few studies have been conducted on diplodiploid taxa. Studies in the social spiders *Anelosimus eximius* , *A. domingo* , *Stegodyphus dumicola* , and *S. mimosarum* , and the solitary spiders *Oedothorax gibbosus* and *Pityohyphantes phrygianus* showed they have a female biased primary sex ratio (Avilés et al. 1999, 2000; Gunnarsson et al. 2009; Vanthournout et al. 2011; Vollrath 1986). In the cases of *S. dumicola* and *S. mimosarum* , it has been shown that the bias is due to males producing significantly more X‐carrying sperm than 0‐sperm (Vanthournout et al. 2018). In the cases of solitary spiders, the deviation in the sex ratio in favour of females is related to the presence of the maternally inherited endosymbiont bacteria *Wolbachia* (Gunnarsson et al. 2009; Vanthournout et al. 2011; Vanthournout, Vandomme, and Hendrickx 2014).

A bias in the primary sex ratio of a species or population entails a bias in the operational sex ratio (i.e., females and males ready to breed), which will have implications for the sexual strategies of the species (Gunnarsson and Andersson 1996; Hardy 1992). For example, in the butterfly *Acraea encedon* there are populations with female biased sex ratios due to the presence of *Wolbachia*, and this bias causes the sex role reversal in this species (Jiggins et al. 1998). The evidences found in *A. marindia* (i.e., female biased sex ratio and presence of *Wolbachia*) raise new questions about the cause‐effect of the presence of the bacteria and the observed bias found in the species, and if this is so, about the relationship of the bacteria and the reversal of sexual roles described in this species (Aisenberg and Costa 2008; Lerette et al. 2015). For this reason, in future studies we will seek to sex the offspring of a large number of mothers by flow cytometry to determine whether there is a relationship between the bias towards females in this species and the presence of the endobacteria *Wolbachia*.

Our results raise the possibility of addressing future questions, such as whether the sex ratio of clutches changes throughout the reproductive life of females; whether the sex ratio biases reported in adults of various social or other web spiders are already present at hatchlings; whether different maternal histories or environmental factors influence hatchling sex ratio; and whether dispersal abilities differ between male and female hatchlings. Furthermore, this approach could contribute to identifying morphological traits in hatchlings that allow for early sex determination.

Undoubtedly, flow cytometry is a promising technique for sexing other diplodiploid arthropods, as well as other animal taxa for which sex is genetically determined by other karyotypic configurations. Our approach is straightforward and does not depend on prior knowledge of the genome and species karyotype as is required by real‐time polymerase chain reaction (PCR‐RT) and quantitative polymerase chain reaction (qPCR) based methods, as reported for beetles, birds, canids and deer (González et al. 2015; Houchen et al. 2024; Morera et al. 2025; Sedláková et al. 2022); it only depends on whether the differences in DNA content between sexes exceed the expected measurement error by flow cytometry. This opens up a huge range of new questions, as well as answers to many existing ones, about sexual dimorphism, sexual strategies, differences in dispersal between sexes, trait sex differences in early stages of development and plasticity, population dynamics, among others.

## Author Contributions

**Leticia Bidegaray‐Batista:** conceptualization (lead), data curation (equal), formal analysis (equal), funding acquisition (equal), investigation (equal), methodology (equal), project administration (lead), resources (lead), supervision (lead), writing – original draft (lead), writing – review and editing (lead). **Nadia Kacevas:** data curation (equal), formal analysis (equal), investigation (equal), methodology (equal), writing – review and editing (equal). **Federico F. Santiñaque:** data curation (equal), formal analysis (equal), methodology (equal), writing – review and editing (equal). **Magdalena Vaio:** formal analysis (equal), methodology (equal), writing – review and editing (equal). **Macarena González:** data curation (equal), formal analysis (equal), investigation (equal), methodology (equal), writing – review and editing (equal).

## Funding

This study was funded by Fondo Clemente Estable (FCE), Agencia Nacional de Investigación e Innovación (ANII) project FCE_1_2017_1_136269 and FCE_1_2023_1_176160, Dr. Carlos Carbajal Campi, FAICE GENBIO, and had the support of the Programa de Desarrollo de las Ciencias Básicas (PEDECIBA).

## Conflicts of Interest

The authors declare no conflicts of interest.

## Supporting information


**Data S1:** Flow cytometric DNA histograms obtained from fresh tissue of adults of *A. marindia* .

**Data S2:** Flow cytometric DNA histograms obtained from frozen tissue of adults and spiderlings of *A. marindia* .

## Data Availability

The data that supports the findings of this study is available as Supporting Information (Data S1 and S2).
