# Supplementary material for: Tell Me How Much DNA You Have and I'll Tell You What Your Sex Is: Sex Determination by Flow Cytometry of Spiderlings of Allocosa marindia
Source: Ecol Evol. 2026 Apr 22;16(4):e73453. doi: 10.1002/ece3.73453 (PMC13103279; doi:10.1002/ece3.73453)
Supplement: Supplementary file 1 — Data S1: Flow cytometric DNA histograms obtained from fresh tissue of adults of A. marindia . [file ECE3-16-e73453-s002.pdf]

BECKMAN COULTER

A.marindia_female_1

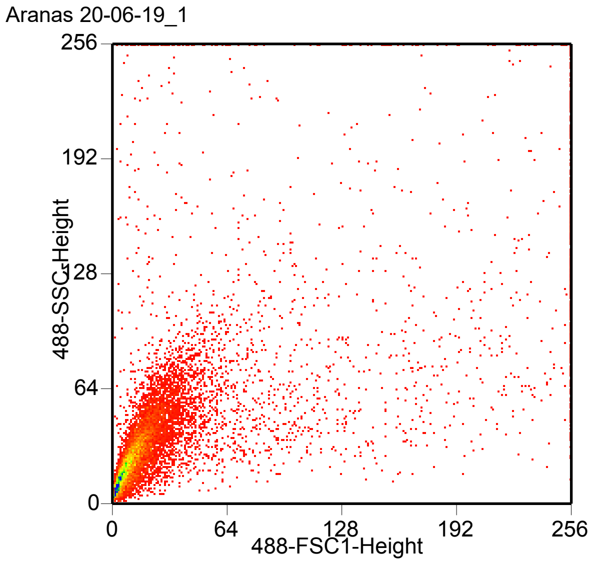

Region	Count	% Hist	% All
Total	10995	100.00	100.00

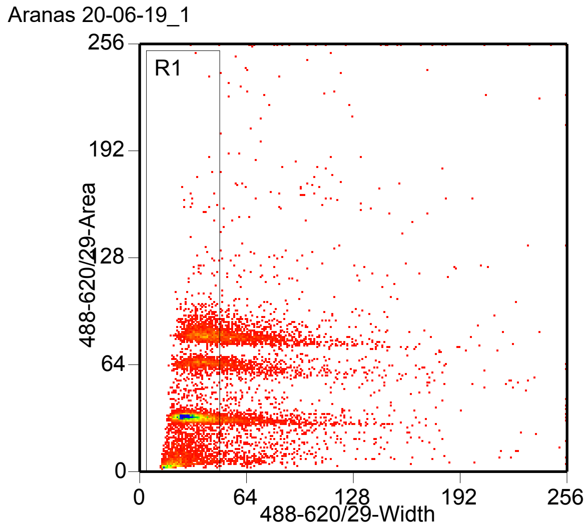

Region	Count	% Hist	% All
Total	10995	100.00	100.00
R1	7544	68.61	68.61

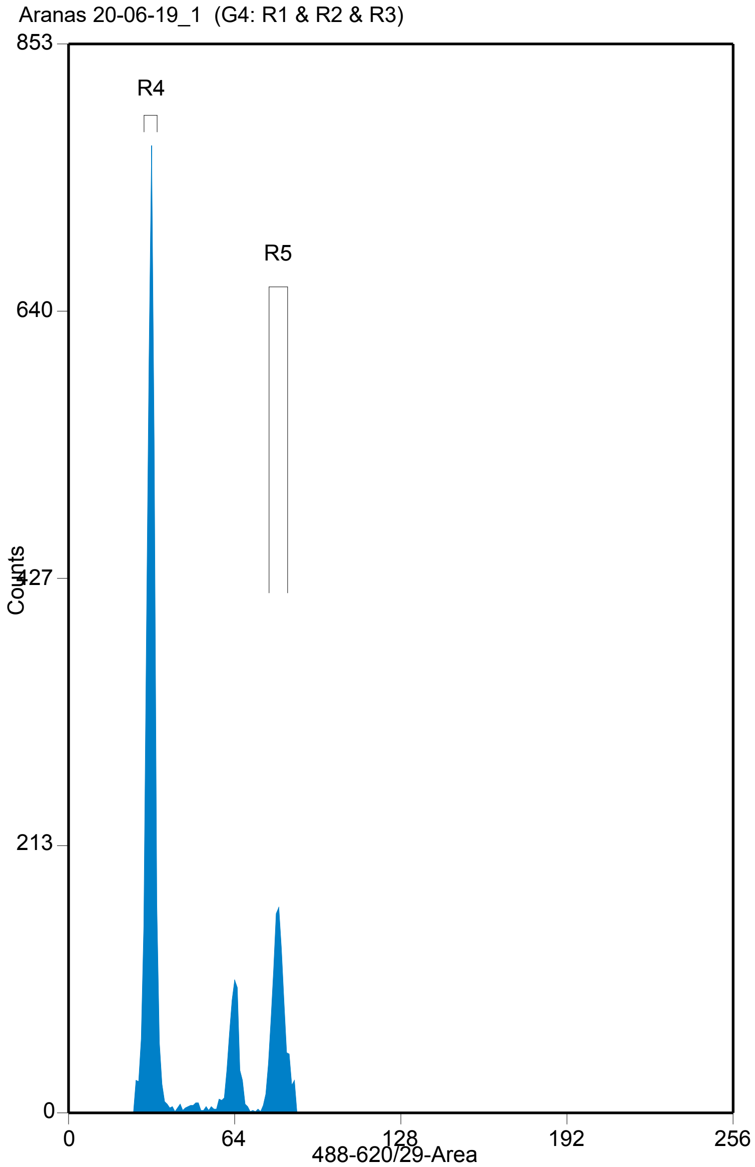

Region	Count	% Hist	Mean	Std Dev.	CV
Total	4329	100.00	46.35	20.99	45.27
R4	2600	60.06	31.64	1.29	4.07
R5	819	18.92	80.62	1.82	2.26

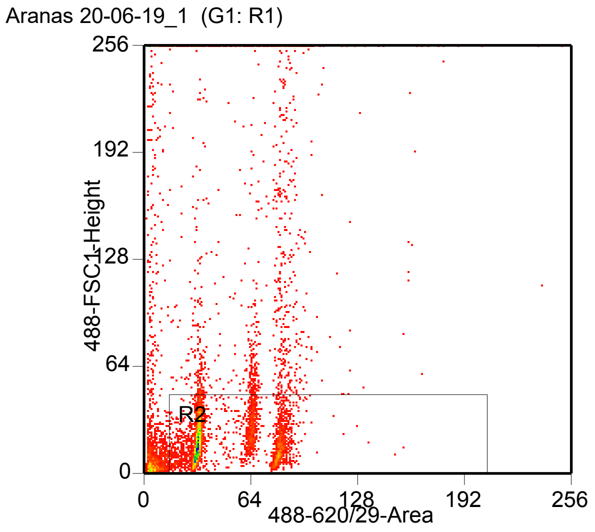

Region	Count	% Hist	% All
Total	7544	100.00	68.61
R2	4987	66.11	45.36

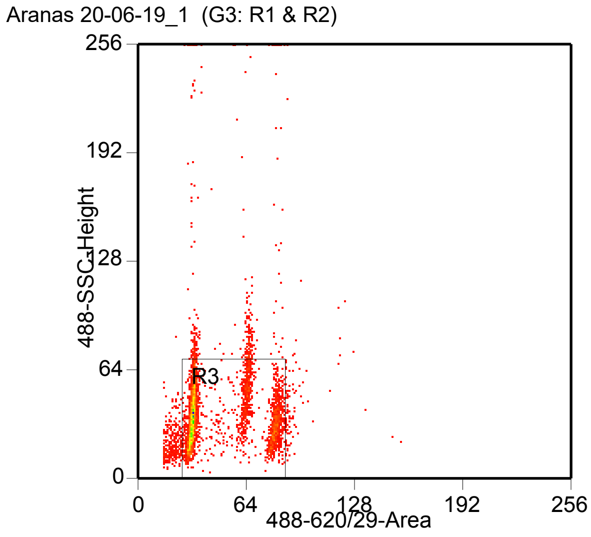

Region	Count	% Hist	% All
Total	4987	100.00	45.36
R3	4329	86.81	39.37

A.marindia_male_1

BECKMAN COULTER

Aranas 20-06-19_2

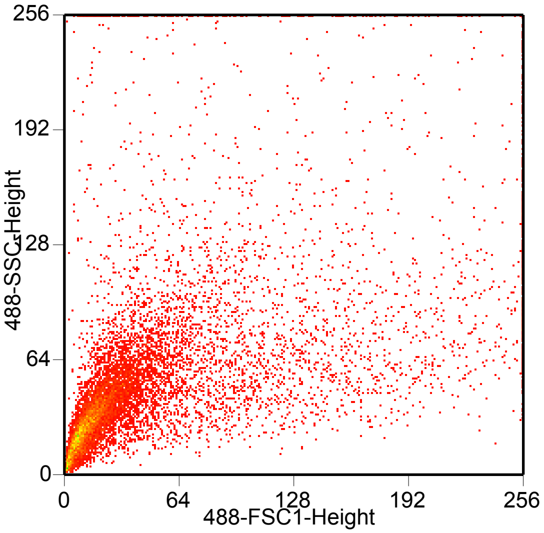

Region	Count	% Hist	% All
Total	10976	100.00	100.00

Aranas 20-06-19_2

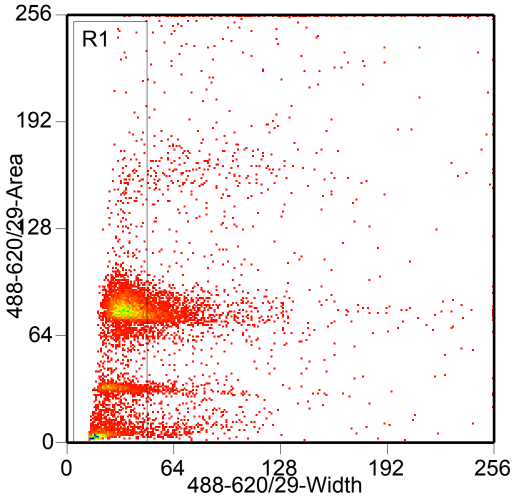

Region	Count	% Hist	% All
Total	10976	100.00	100.00
R1	8213	74.83	74.83

Aranas 20-06-19_2 (G4: R1 & R2 & R3)

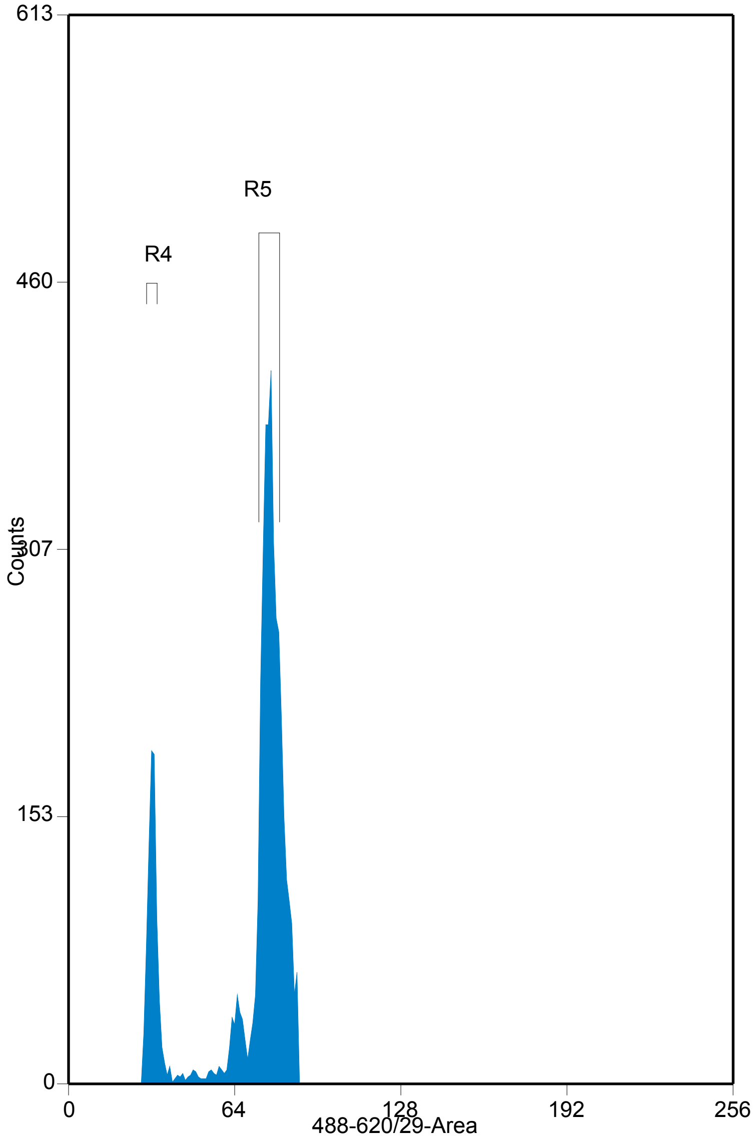

Region	Count	% Hist	Mean	Std Dev.	CV
Total	4742	100.00	69.30	17.87	25.79
R4	692	14.59	32.12	1.21	3.77
R5	2645	55.78	77.29	2.25	2.91

Aranas 20-06-19_2 (G1: R1)

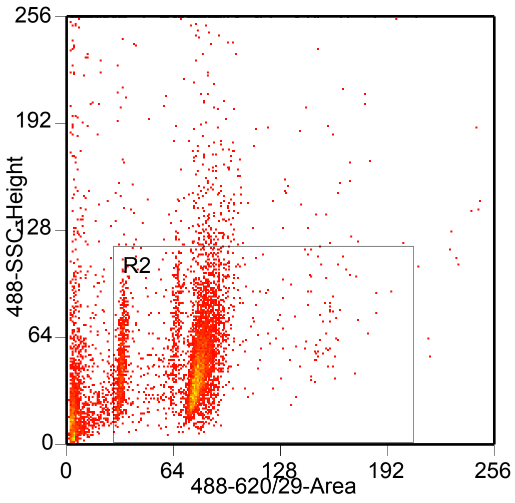

Region	Count	% Hist	% All
Total	8213	100.00	74.83
R2	5962	72.59	54.32

Aranas 20-06-19_2 (G3: R1 & R2)

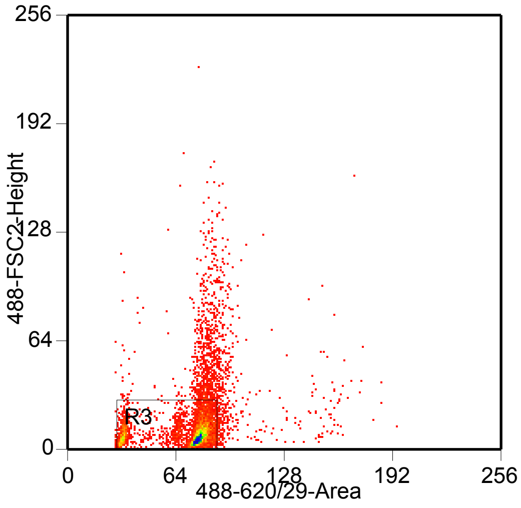

Region	Count	% Hist	% All
Total	5962	100.00	54.32
R3	4742	79.54	43.20

BECKMAN COULTER

A.marindia_female_2

ADN Spiders 16-07-19_1

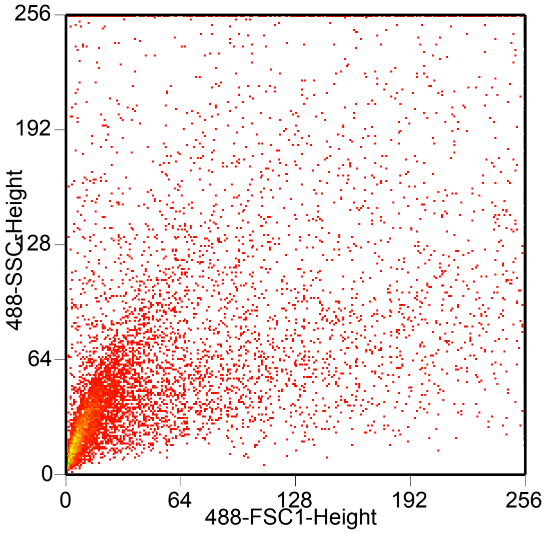

Region	Count	% Hist	% All
Total	10030	100.00	100.00

ADN Spiders 16-07-19_1

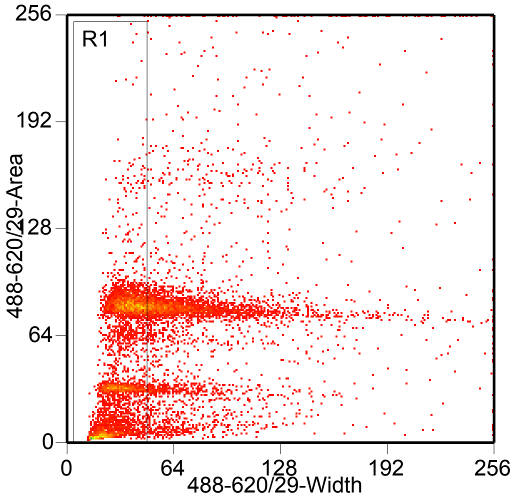

Region	Count	% Hist	% All
Total	10030	100.00	100.00
R1	5766	57.49	57.49

ADN Spiders 16-07-19_1 (G4: R1 & R2 & R3)

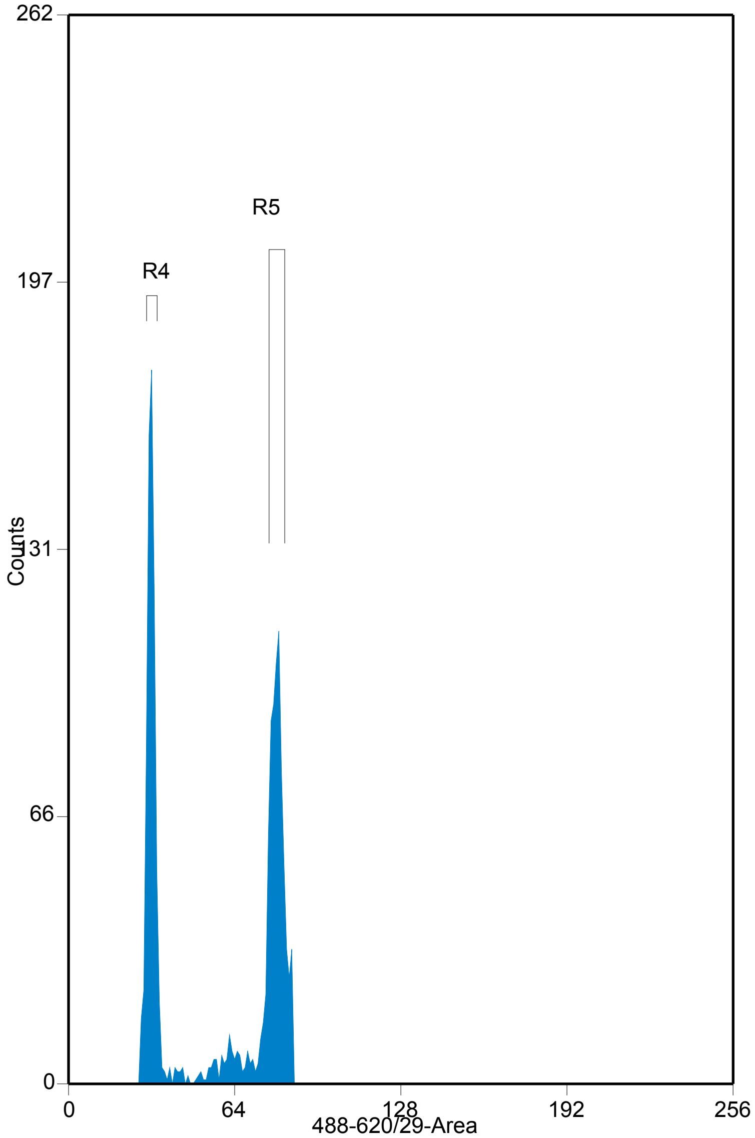

Region	Count	% Hist	Mean	Std Dev.	CV
Total	1528	100.00	57.50	23.34	40.58
R4	591	38.68	31.83	1.17	3.67
R5	588	38.48	79.95	1.80	2.25

ADN Spiders 16-07-19_1 (G1: R1)

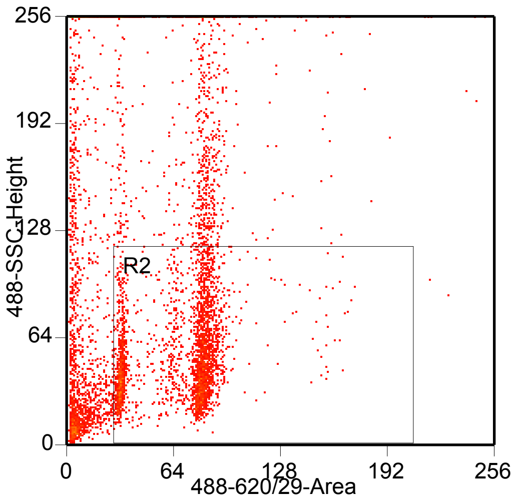

Region	Count	% Hist	% All
Total	5766	100.00	57.49
R2	3229	56.00	32.19

ADN Spiders 16-07-19_1 (G3: R1 & R2)

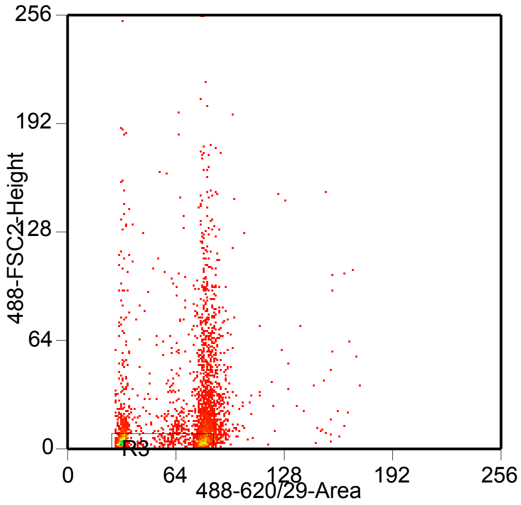

Region	Count	% Hist	% All
Total	3229	100.00	32.19
R3	1528	47.32	15.23

A.marindia_male_2

BECKMAN COULTER

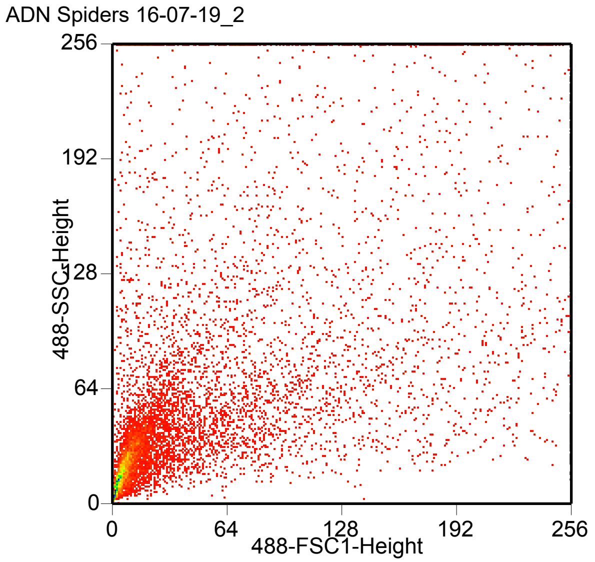

Region	Count	% Hist	% All
Total	10112	100.00	100.00

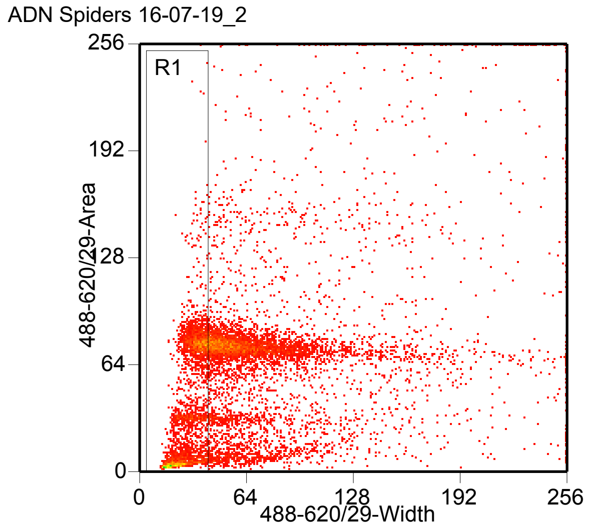

Region	Count	% Hist	% All
Total	10112	100.00	100.00
R1	3774	37.32	37.32

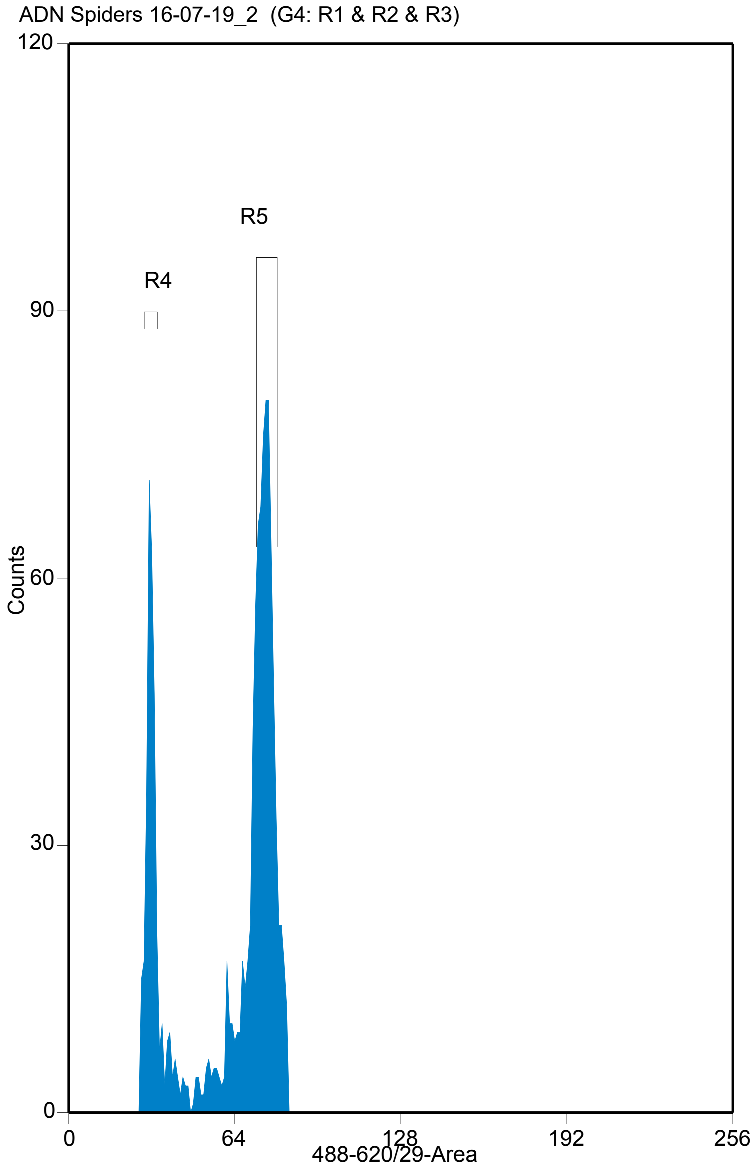

Region	Count	% Hist	Mean	Std Dev.	CV
Total	1199	100.00	62.15	19.07	30.69
R4	254	21.18	31.58	1.33	4.21
R5	572	47.71	75.73	2.33	3.08

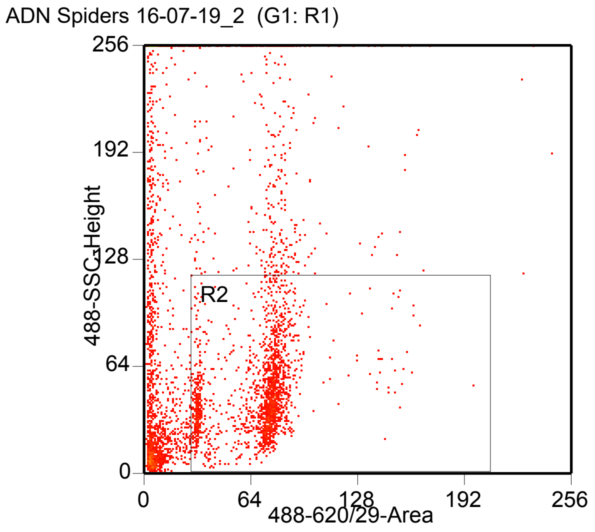

Region	Count	% Hist	% All
Total	3774	100.00	37.32
R2	1854	49.13	18.33

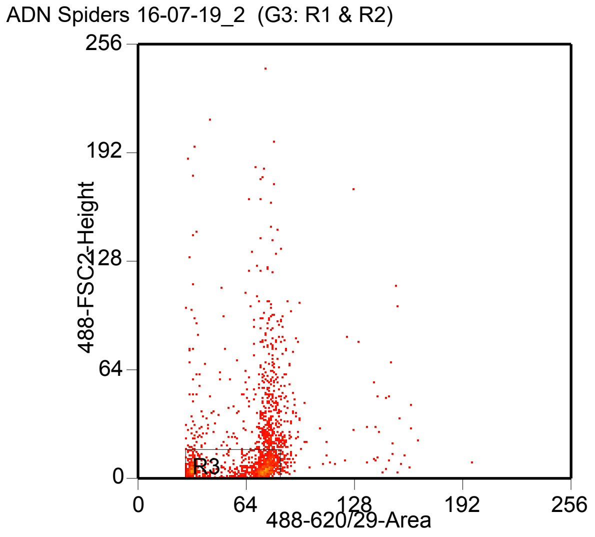

Region	Count	% Hist	% All
Total	1854	100.00	18.33
R3	1199	64.67	11.86

A.marindia_female_3

DNA Spieders_1

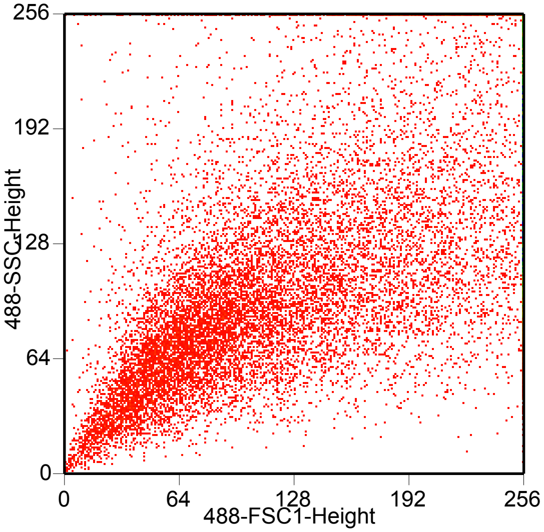

Region	Count	% Hist	% All
Total	23664	100.00	100.00

DNA Spieders_1

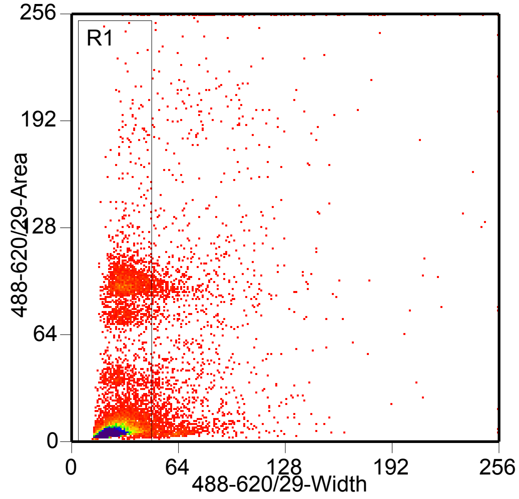

Region	Count	% Hist	% All
Total	23664	100.00	100.00
R1	21546	91.05	91.05

DNA Spieders_1 (G4: R1 & R2 & R3)

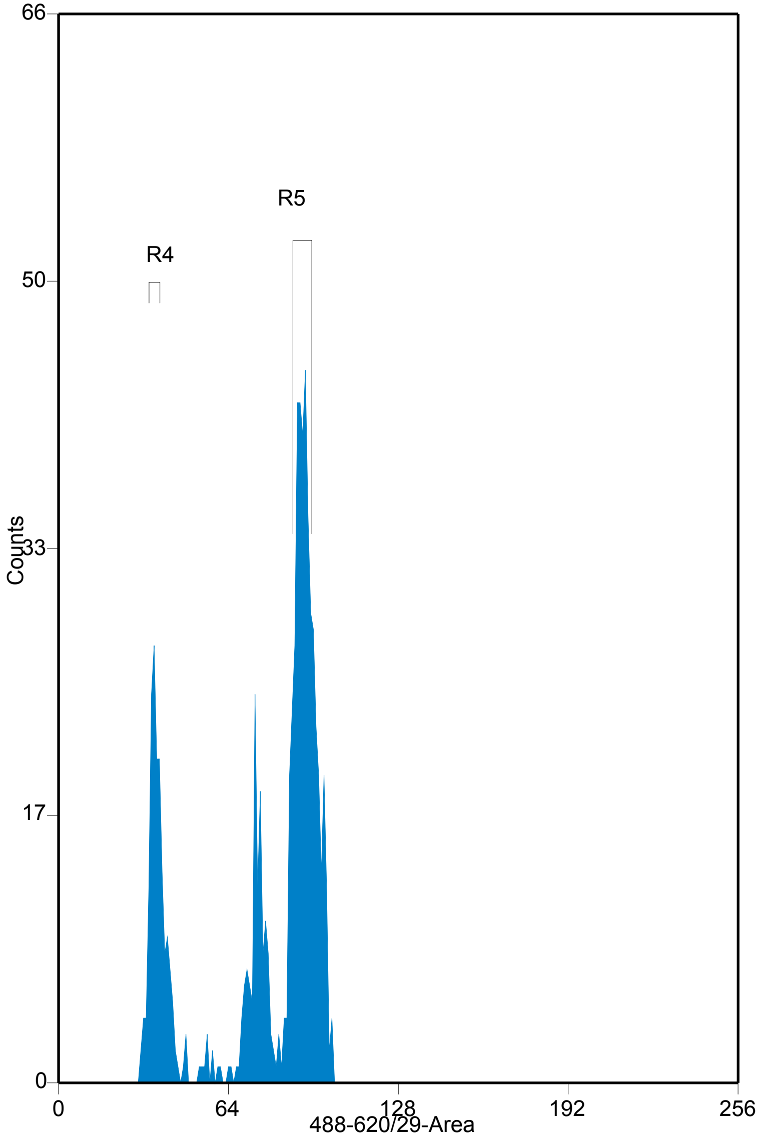

Region	Count	% Hist	Mean	Std Dev.	CV
Total	722	100.00	77.10	22.94	29.75
R4	103	14.27	36.12	1.29	3.56
R5	282	39.06	91.65	2.09	2.28

DNA Spieders_1 (G1: R1)

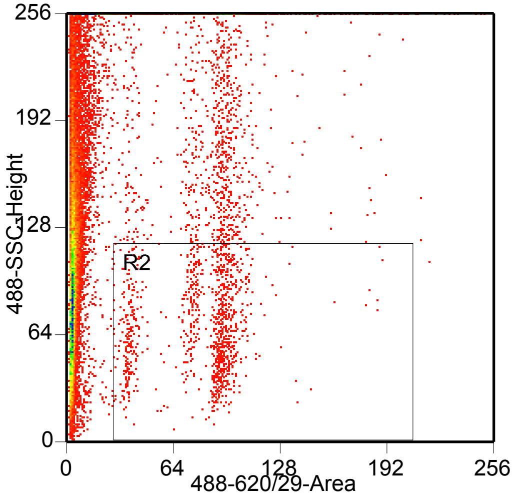

Region	Count	% Hist	% All
Total	21546	100.00	91.05
R2	1091	5.06	4.61

DNA Spieders_1 (G3: R1 & R2)

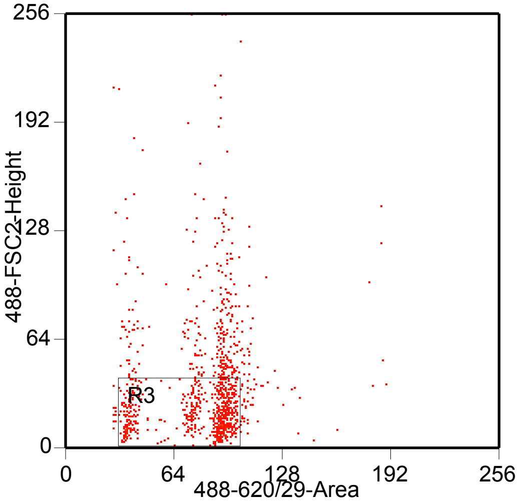

Region	Count	% Hist	% All
Total	1091	100.00	4.61
R3	722	66.18	3.05

A.marindia_male_3

DNA Spieders_2

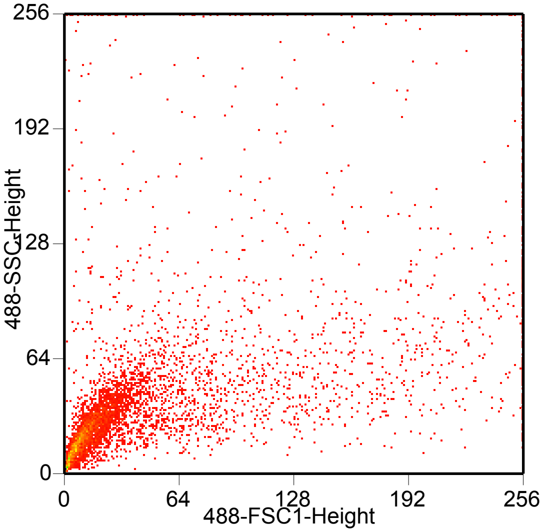

Region	Count	% Hist	% All
Total	5491	100.00	100.00

DNA Spieders_2

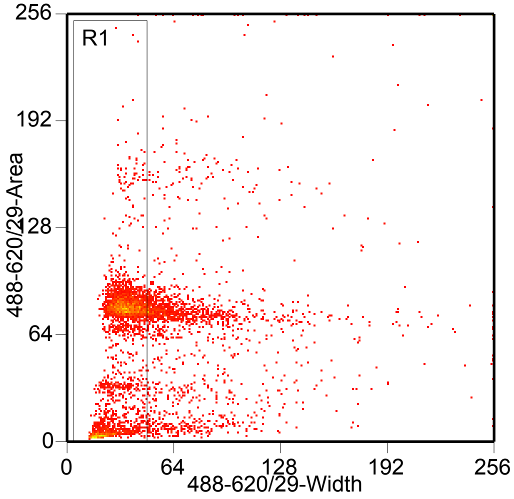

Region	Count	% Hist	% All
Total	5491	100.00	100.00
R1	3796	69.13	69.13

DNA Spieders_2 (G4: R1 & R2 & R3)

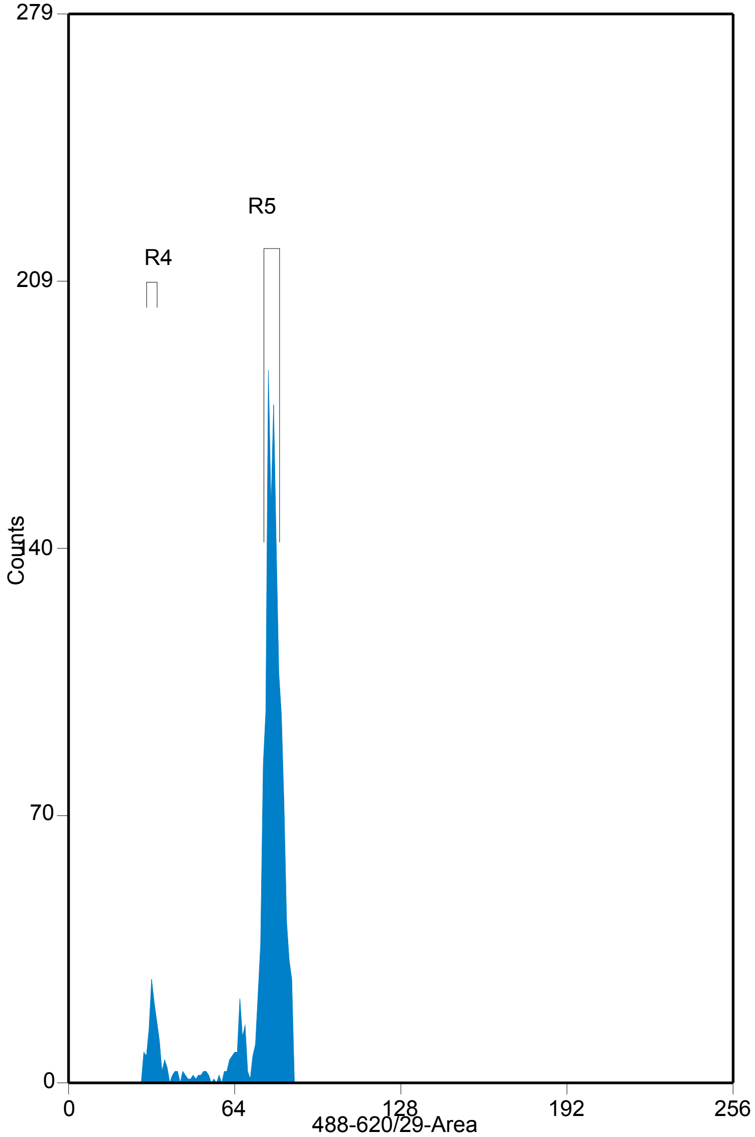

Region	Count	% Hist	Mean	Std Dev.	CV
Total	1525	100.00	73.97	13.36	18.06
R4	85	5.57	32.29	1.19	3.67
R5	941	61.70	78.16	1.79	2.28

DNA Spieders_2 (G1: R1)

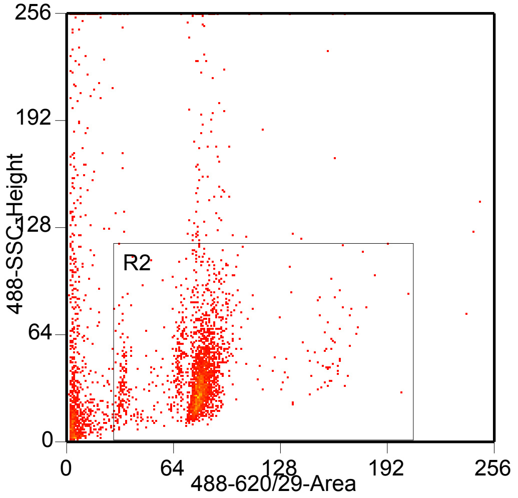

Region	Count	% Hist	% All
Total	3796	100.00	69.13
R2	2715	71.52	49.44

DNA Spieders_2 (G3: R1 & R2)

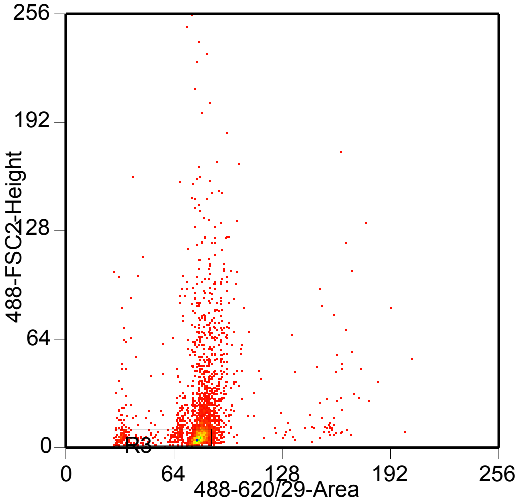

Region	Count	% Hist	% All
Total	2715	100.00	49.44
R3	1525	56.17	27.77
