# Supplementary material for: Tell Me How Much DNA You Have and I'll Tell You What Your Sex Is: Sex Determination by Flow Cytometry of Spiderlings of Allocosa marindia
Source: Ecol Evol. 2026 Apr 22;16(4):e73453. doi: 10.1002/ece3.73453 (PMC13103279; doi:10.1002/ece3.73453)
Supplement: Supplementary file 2 — Data S2: Flow cytometric DNA histograms obtained from frozen tissue of adults and spiderlings of A. marindia . [file ECE3-16-e73453-s001.pdf]

IIBCELB787_1

ADN_07_Jan_25_1 (G3: R1 & R2)

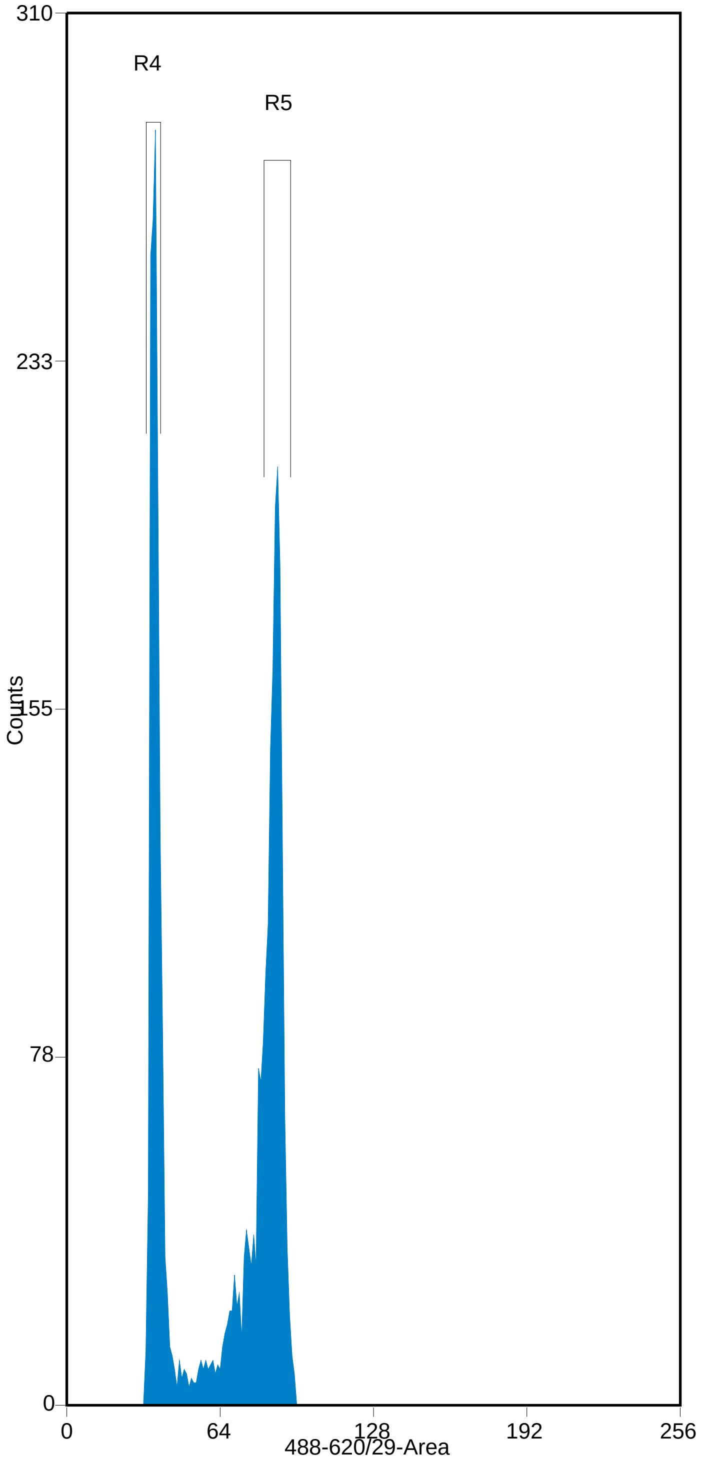

Region	Count	% Hist	Mean	CV
Total	3497	100.00	64.43	35.86
R4	1196	34.20	36.58	3.88
R5	1434	41.01	86.96	3.03

IIBCELB788_1

ADN_07_Jan_25_2 (G3: R1 & R2)

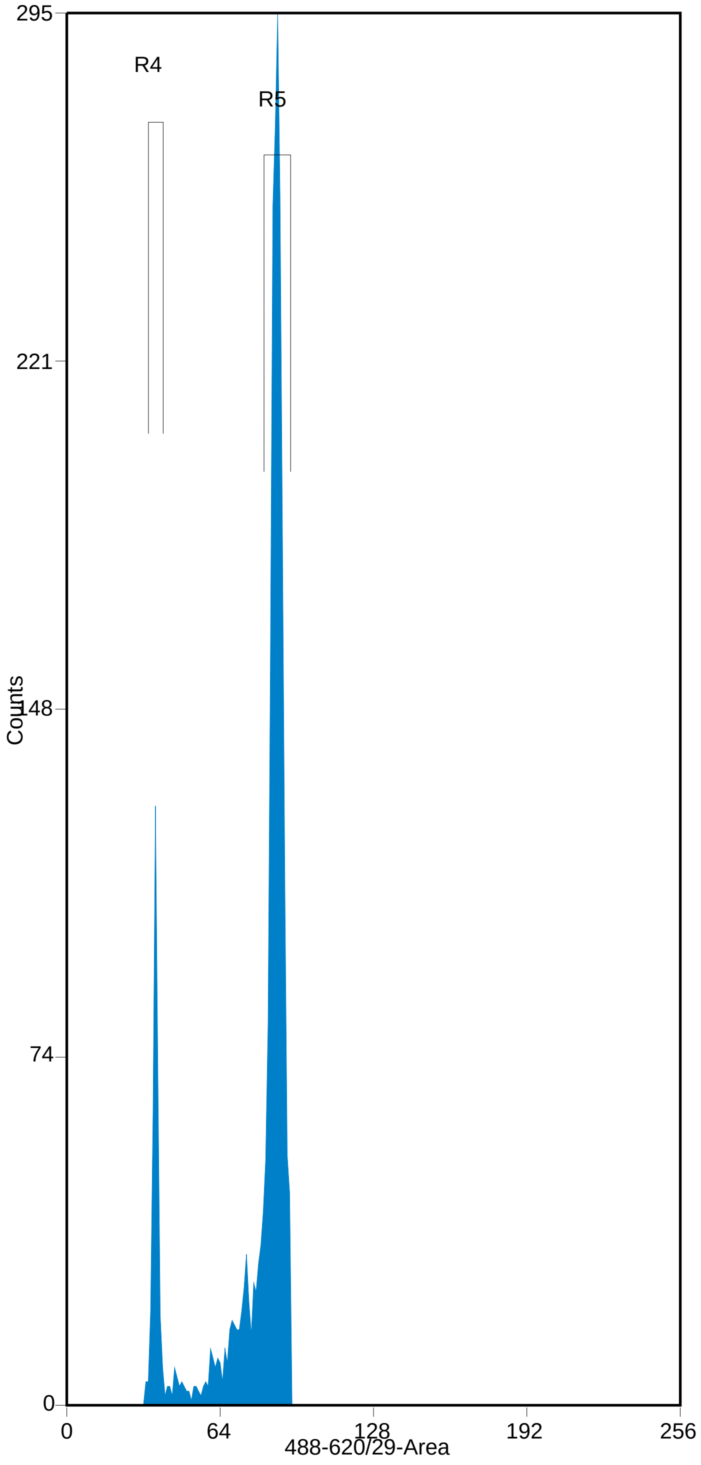

Region	Count	% Hist	Mean	CV
Total	2579	100.00	78.17	22.43
R4	311	12.06	37.05	3.08
R5	1805	69.99	87.62	2.75

IIBCELB789_1

ADN_07_Jan_25_3 (G3: R1 & R2)

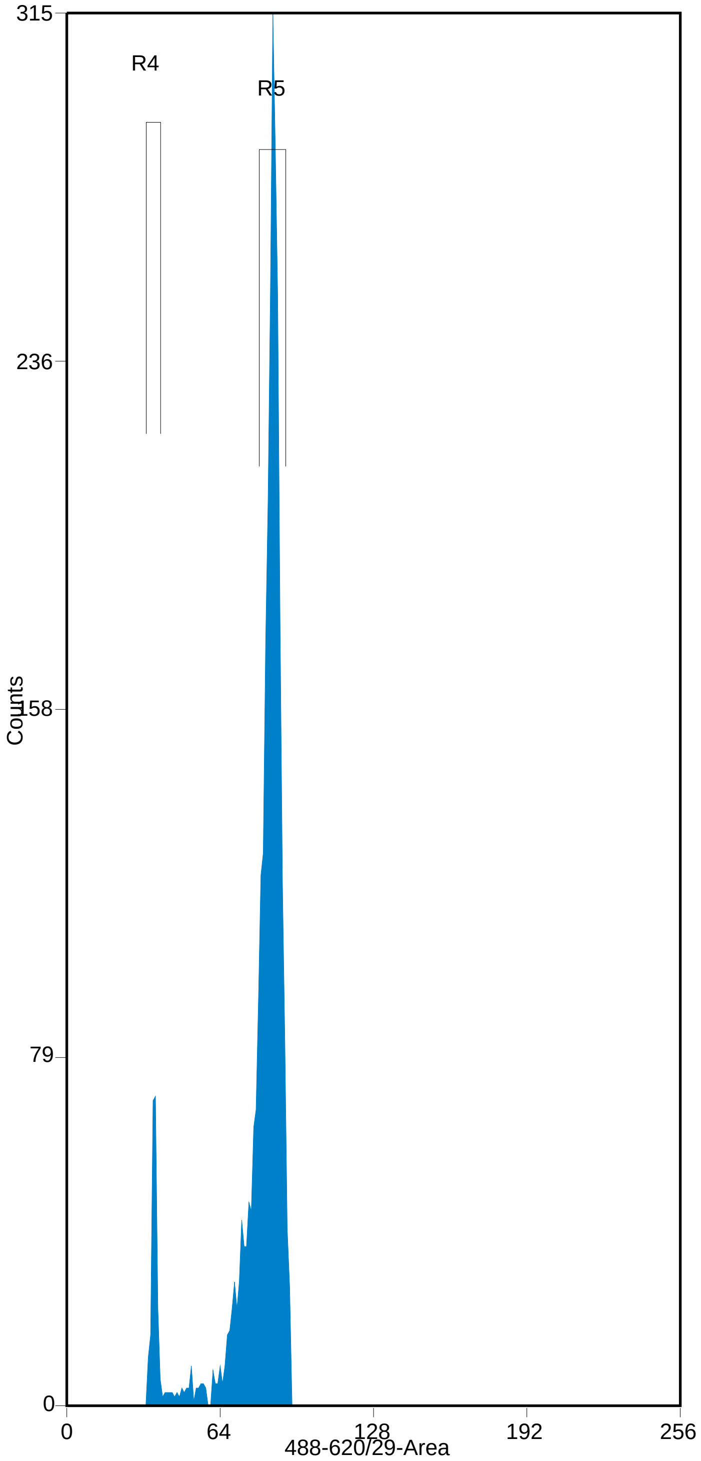

Region	Count	% Hist	Mean	CV
Total	3033	100.00	79.89	17.02
R4	194	6.40	36.48	2.96
R5	2196	72.40	85.71	3.30

IIBCELB790_1

ADN_07_Jan_25_4 (G3: R1 & R2)

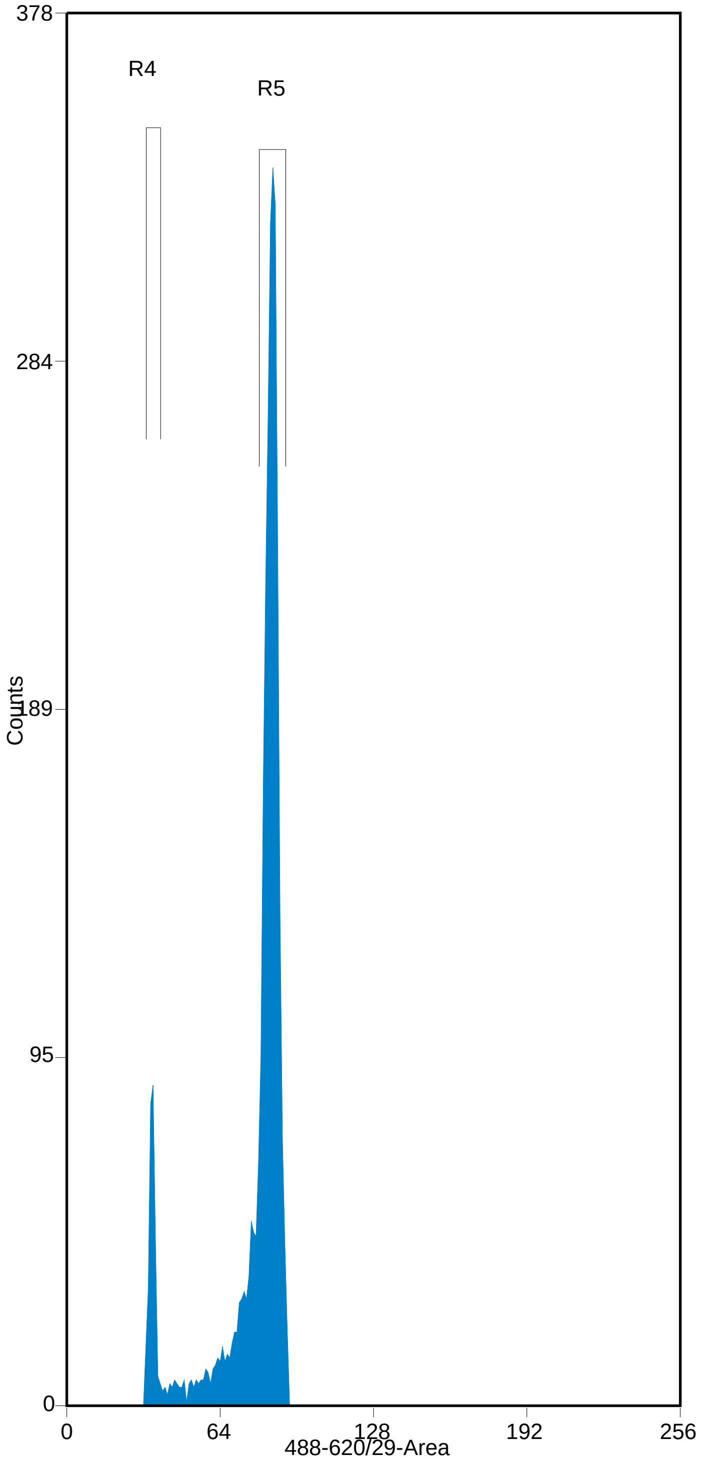

Region	Count	% Hist	Mean	CV
Total	3162	100.00	77.93	19.90
R4	276	8.73	35.58	3.54
R5	2288	72.36	85.40	2.97

BECKMAN COULTER

IIBCELB791_1

ADN_07_Jan_25_5 (G3: R1 & R2)

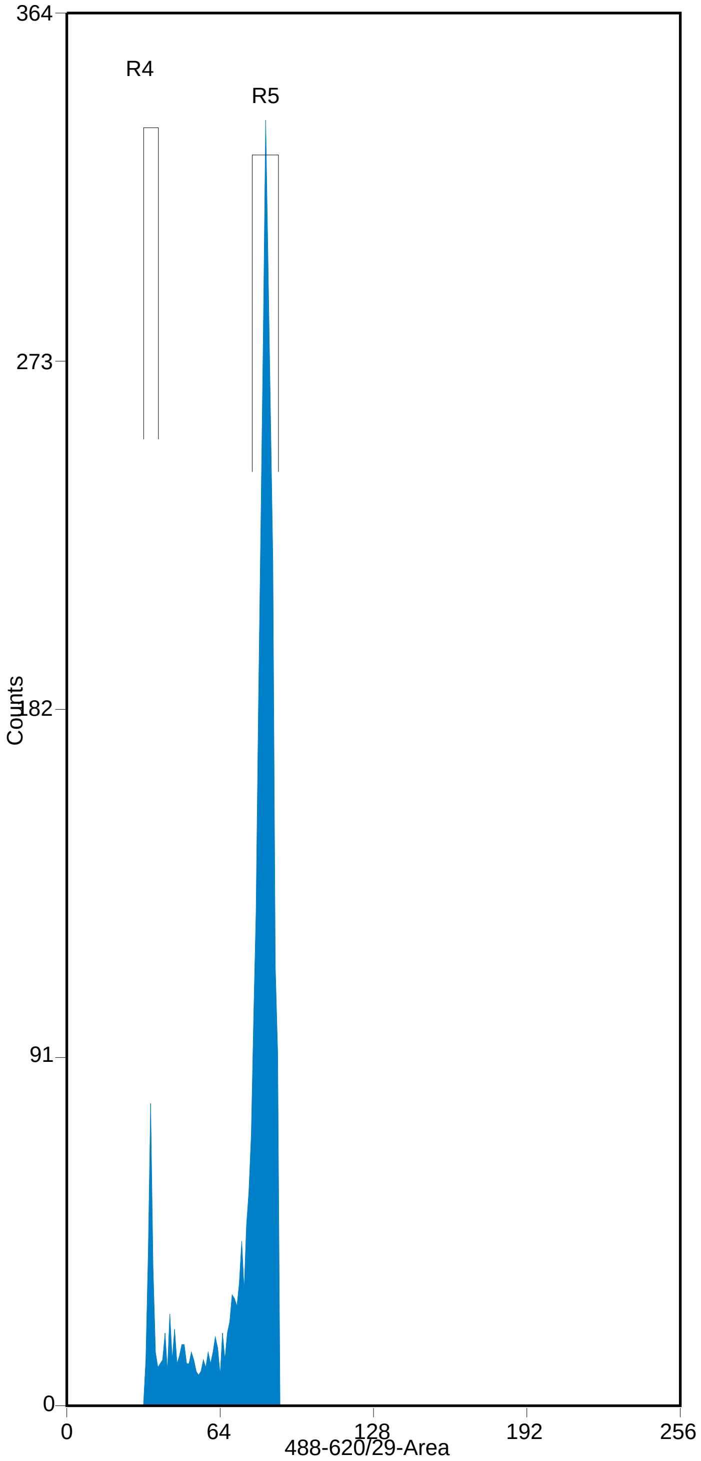

Region	Count	% Hist	Mean	CV
Total	3210	100.00	75.39	19.40
R4	191	5.95	35.16	3.35
R5	2317	72.18	82.85	3.31

IIBCELB798_1

ADN_07_Jan_25_6 (G3: R1 & R2)

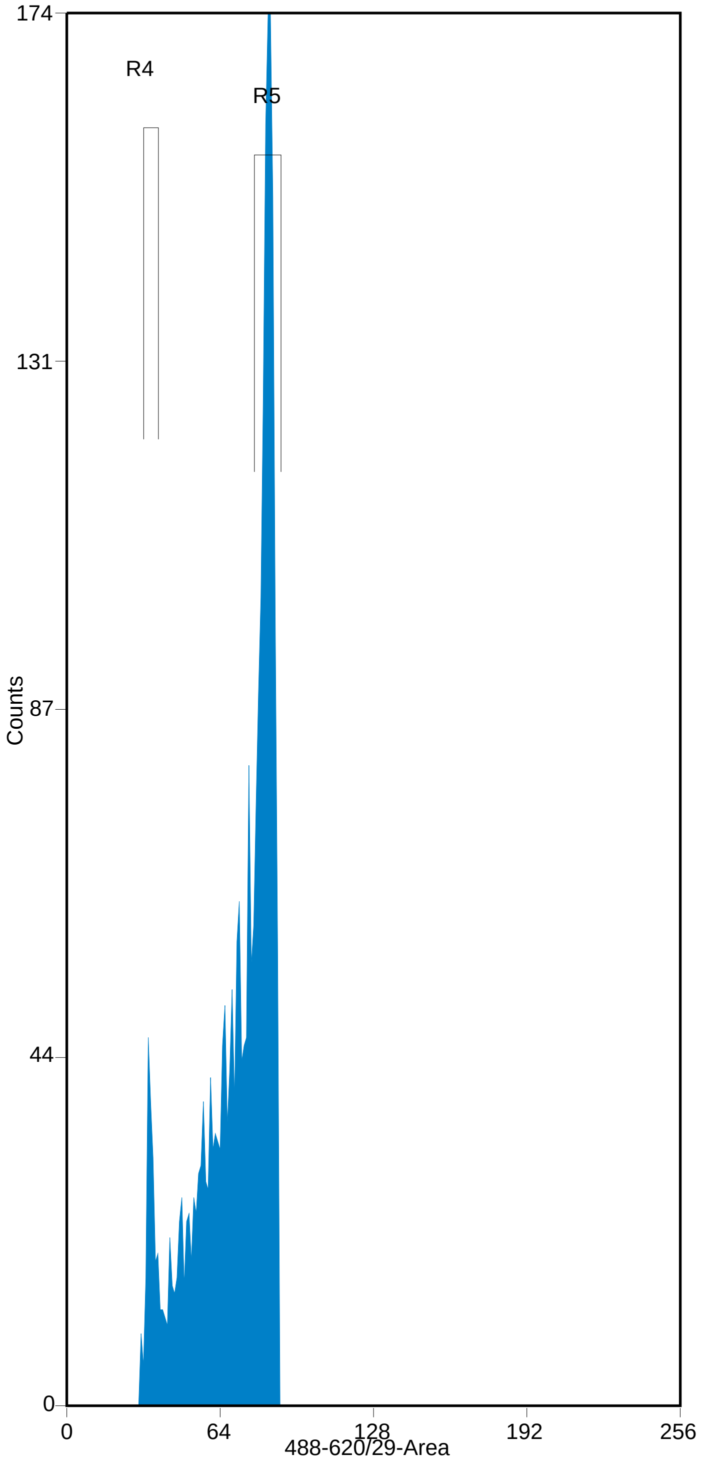

Region	Count	% Hist	Mean	CV
Total	2766	100.00	70.85	21.93
R4	173	6.25	35.18	4.45
R5	1318	47.65	83.41	3.16

BECKMAN COULTER

IIBCELB799_1

ADN_07_Jan_25_7 (G3: R1 & R2)

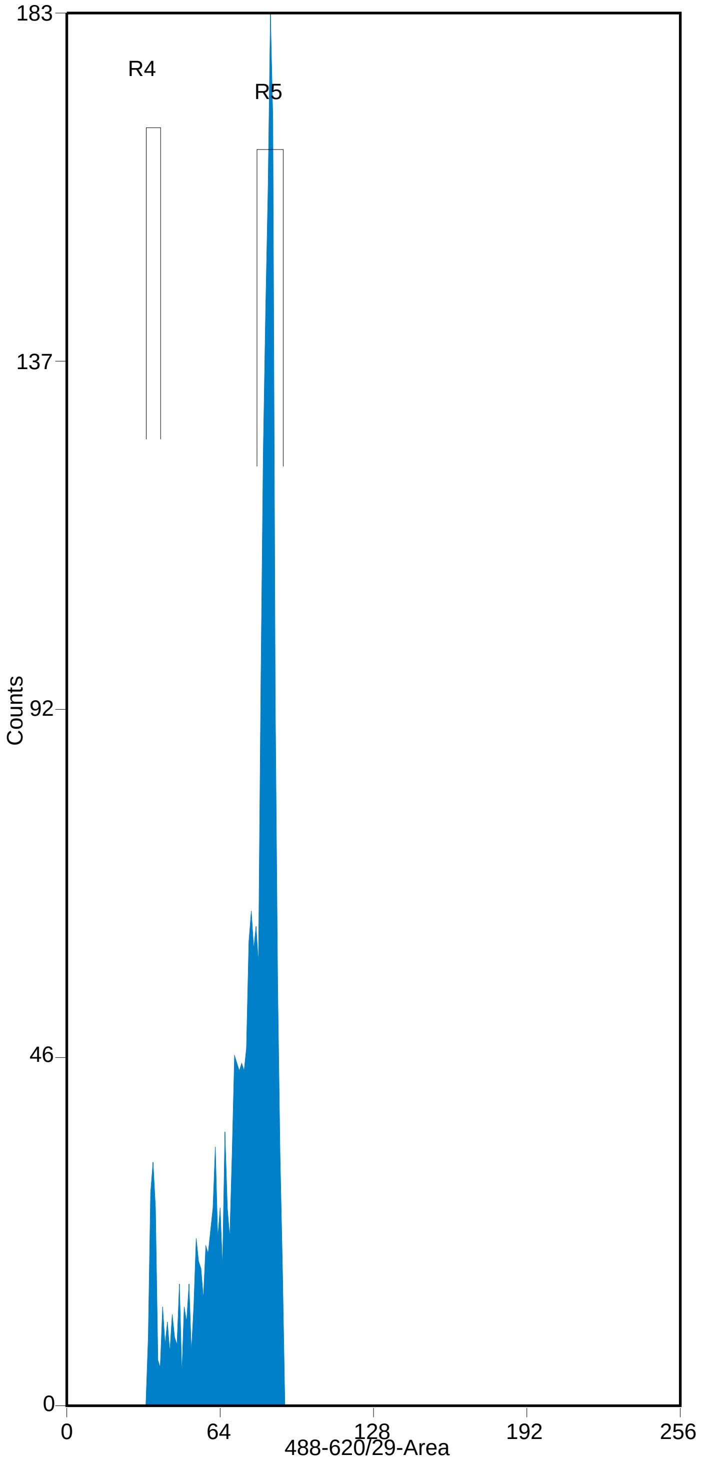

Region	Count	% Hist	Mean	CV
Total	2287	100.00	73.83	19.18
R4	106	4.63	36.07	3.39
R5	1196	52.30	84.05	3.08

IIBCELB793_1

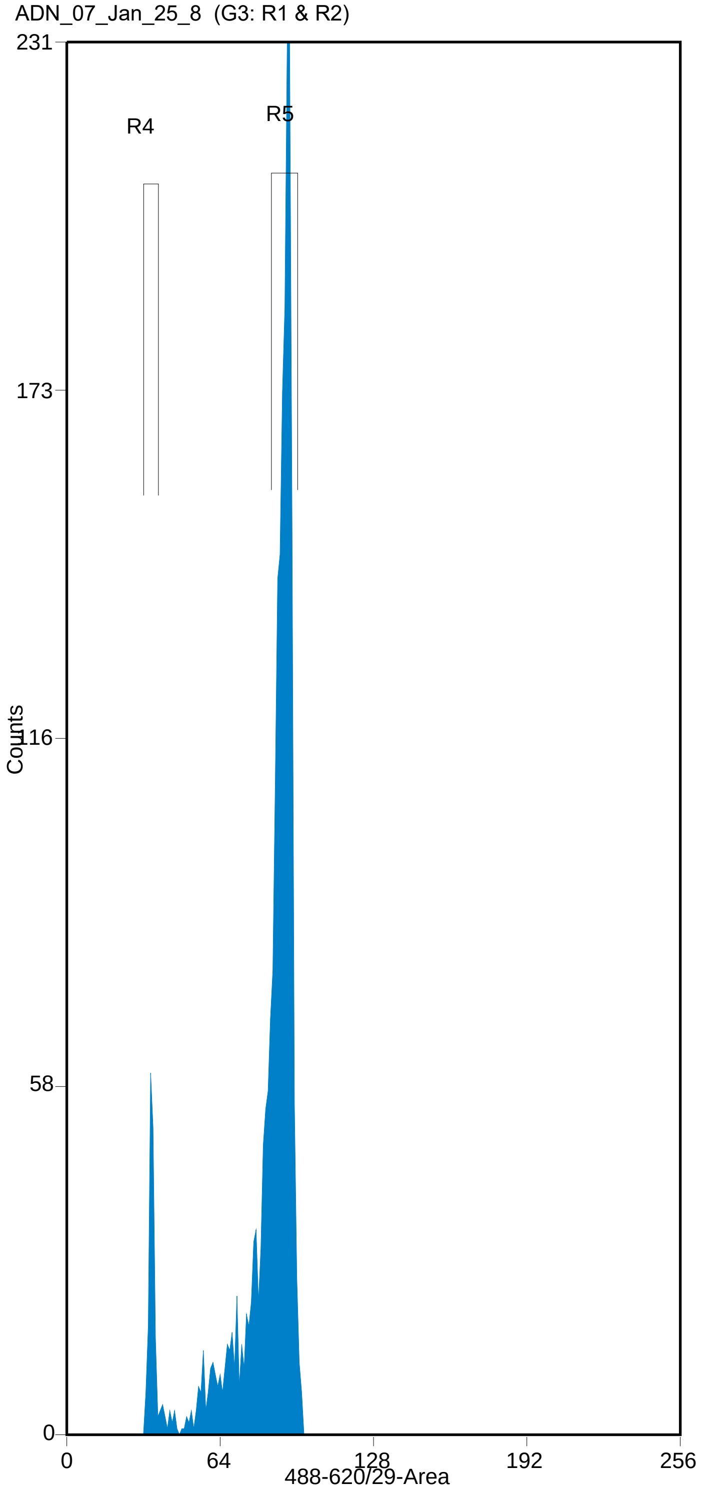

Region	Count	% Hist	Mean	CV
Total	2418	100.00	82.71	19.04
R4	155	6.41	35.39	2.91
R5	1643	67.95	90.67	3.00

IIBCELB794_1

ADN_07_Jan_25_9 (G3: R1 & R2)

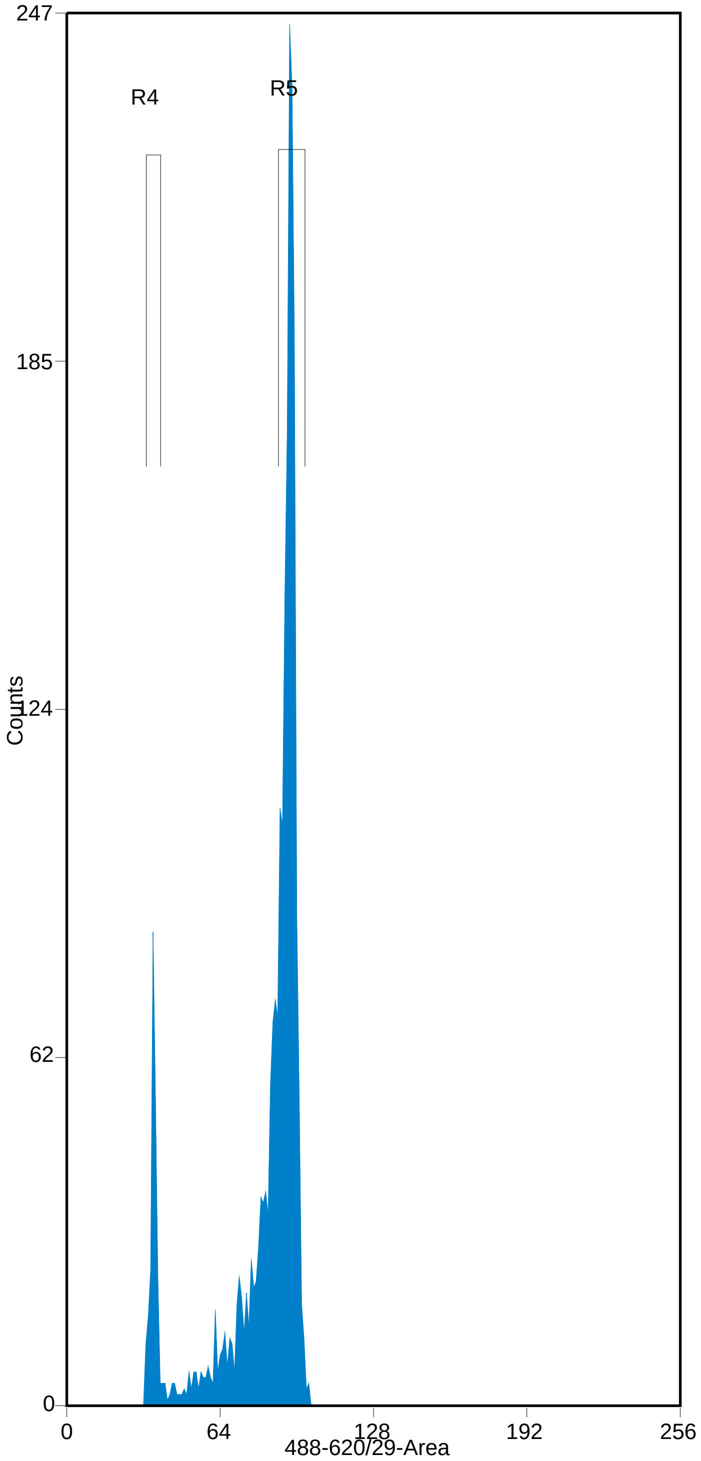

Region	Count	% Hist	Mean	CV
Total	2382	100.00	82.51	21.56
R4	217	9.11	36.11	3.60
R5	1435	60.24	92.80	2.63

IIBCELB795_1

ADN_07_Jan_25_10 (G3: R1 & R2)

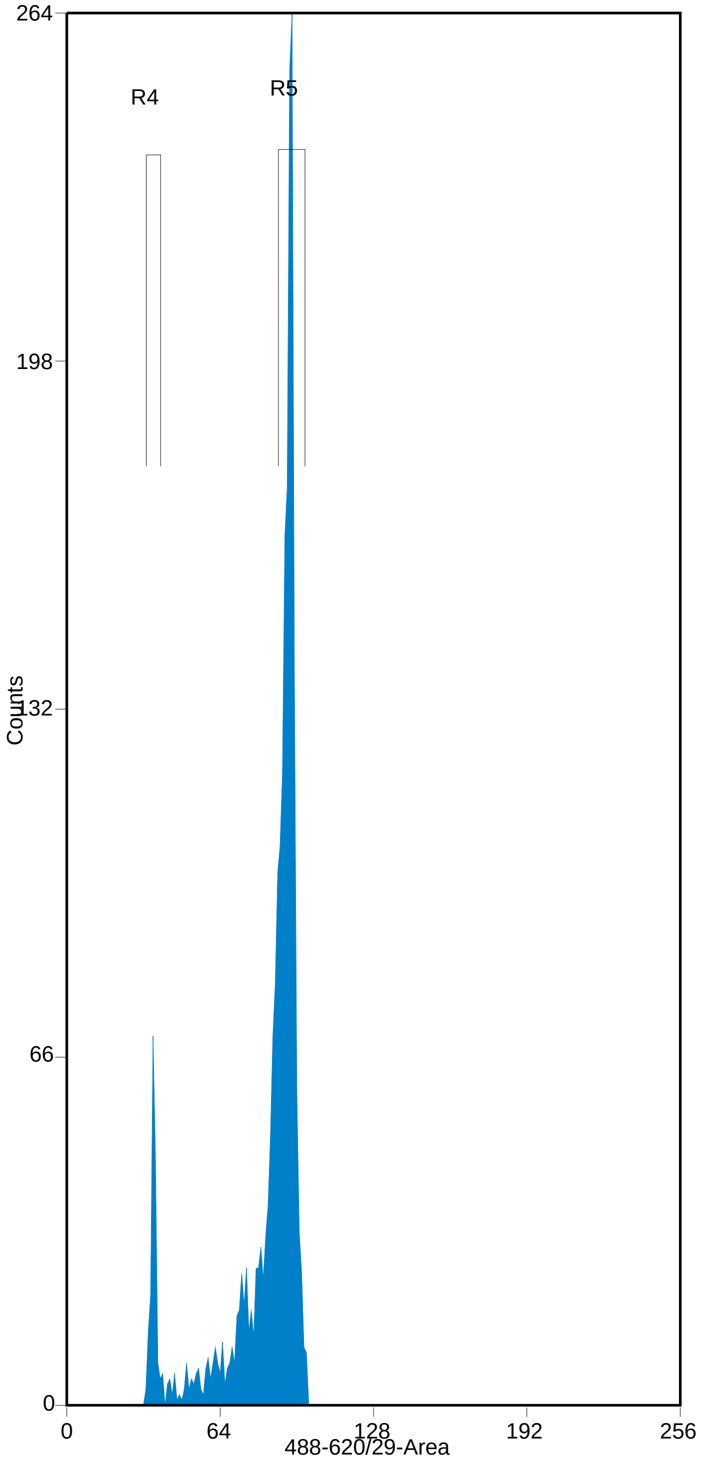

Region	Count	% Hist	Mean	CV
Total	2340	100.00	83.43	19.92
R4	168	7.18	36.12	3.21
R5	1463	62.52	92.50	2.63

BECKMAN COULTER

IIBCELB796_1

ADN_07_Jan_25_11 (G3: R1 & R2)

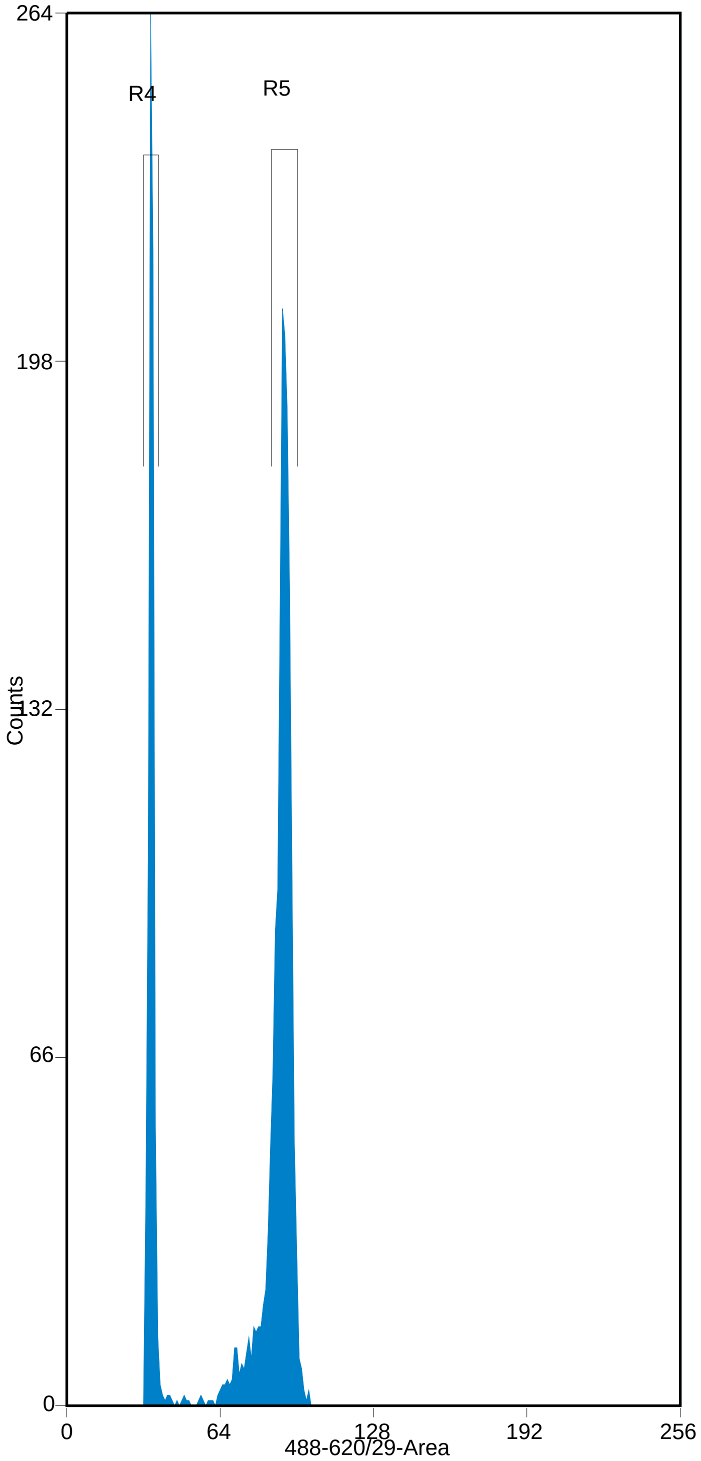

Region	Count	% Hist	Mean	CV
Total	2412	100.00	71.84	35.15
R4	743	30.80	35.24	2.90
R5	1386	57.46	90.55	2.88

IIBCELB797_1

ADN_07_Jan_25_12 (G3: R1 & R2)

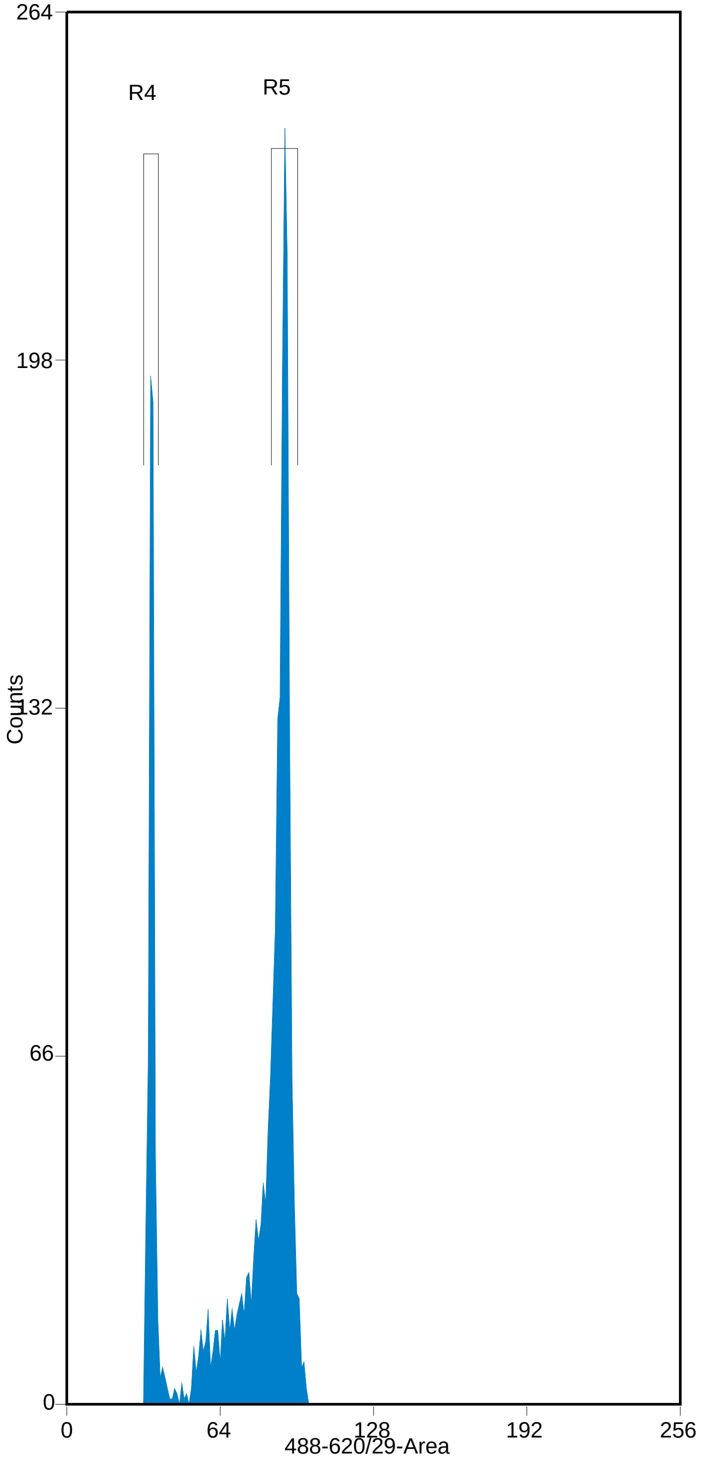

Region	Count	% Hist	Mean	CV
Total	2650	100.00	74.23	29.90
R4	548	20.68	35.36	3.09
R5	1398	52.75	90.25	2.83

IIBCELB801_1

ADN_07_Jan_25_13 (G3: R1 & R2)

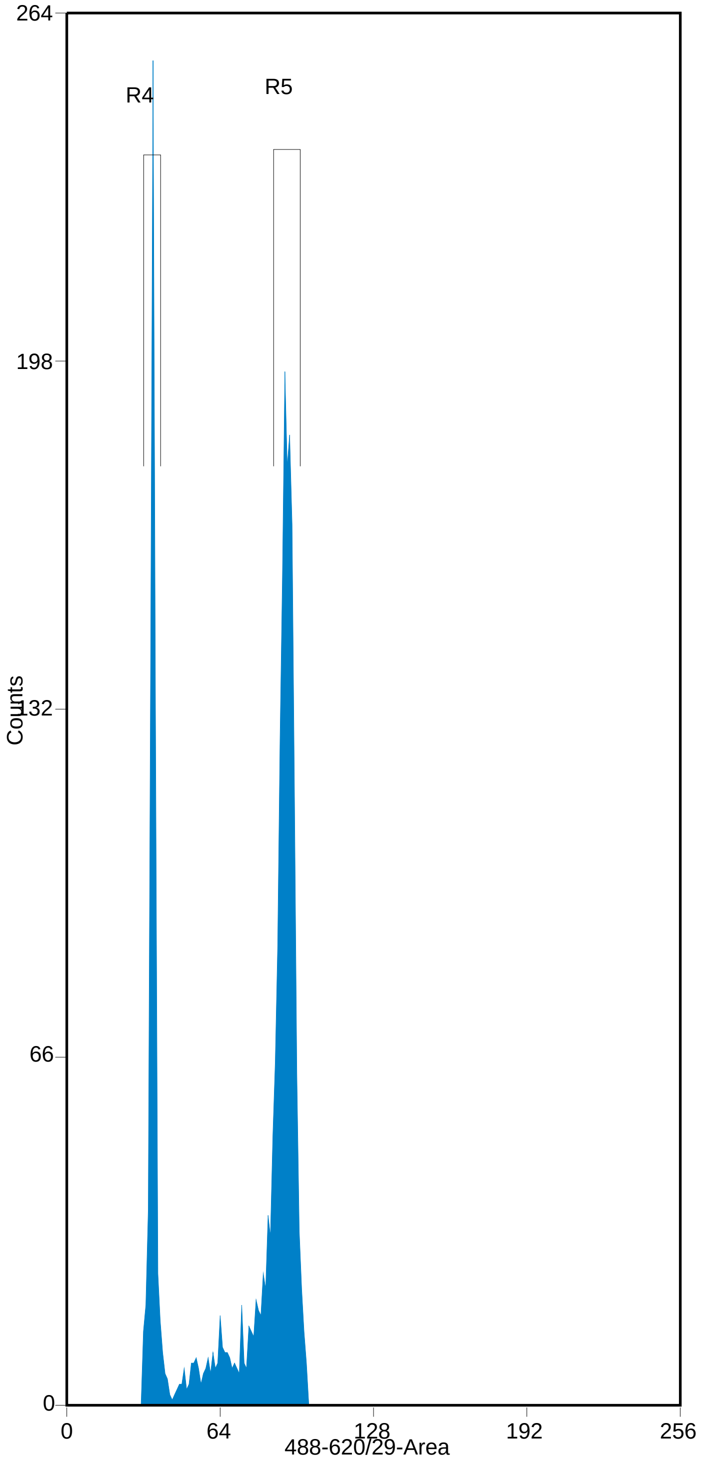

Region	Count	% Hist	Mean	CV
Total	2577	100.00	73.93	32.57
R4	638	24.76	35.85	3.57
R5	1424	55.26	91.59	2.94

IIBCELB802_1

ADN_07_Jan_25_14 (G3: R1 & R2)

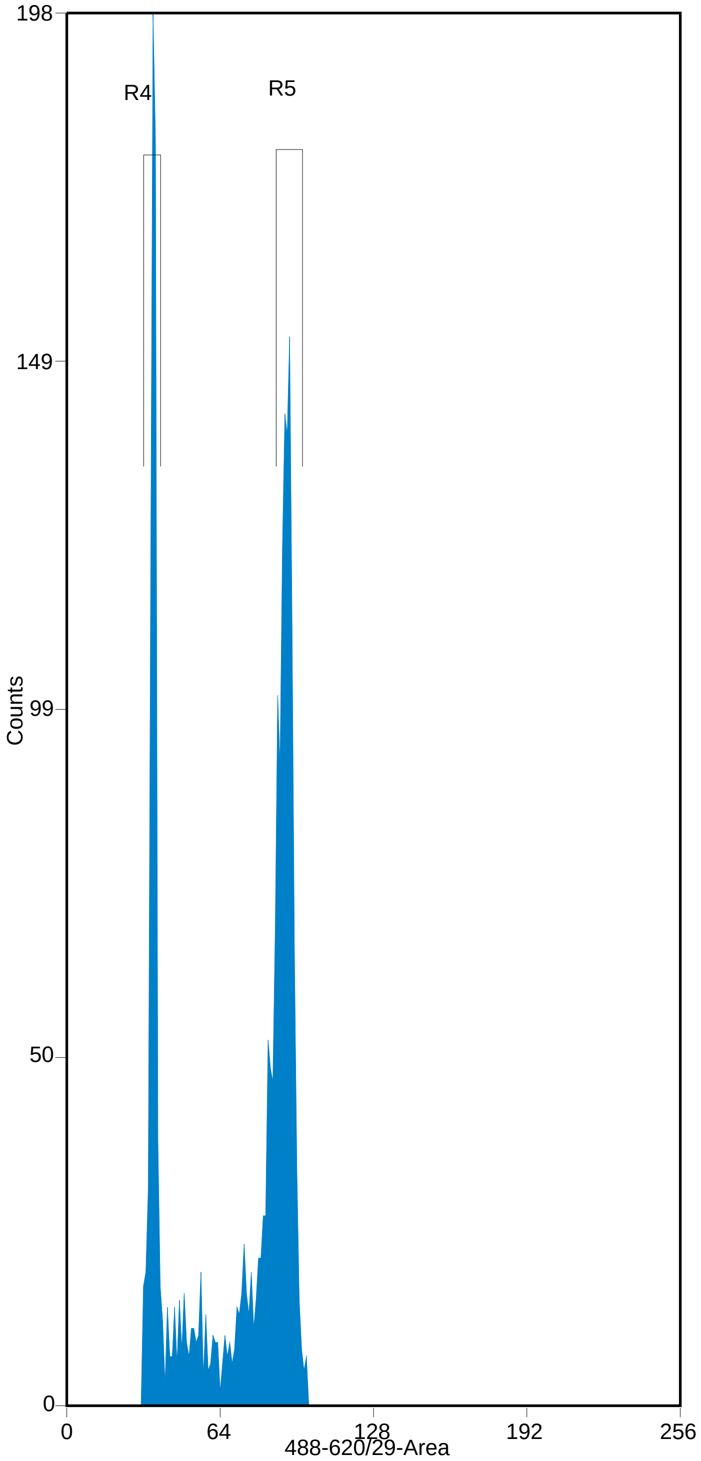

Region	Count	% Hist	Mean	CV
Total	2493	100.00	68.43	36.17
R4	771	30.93	36.01	3.42
R5	1046	41.96	91.45	2.80

spiderling of IIBCELB793_1

ADN_07_Jan_25_15 (G3: R1 & R2)

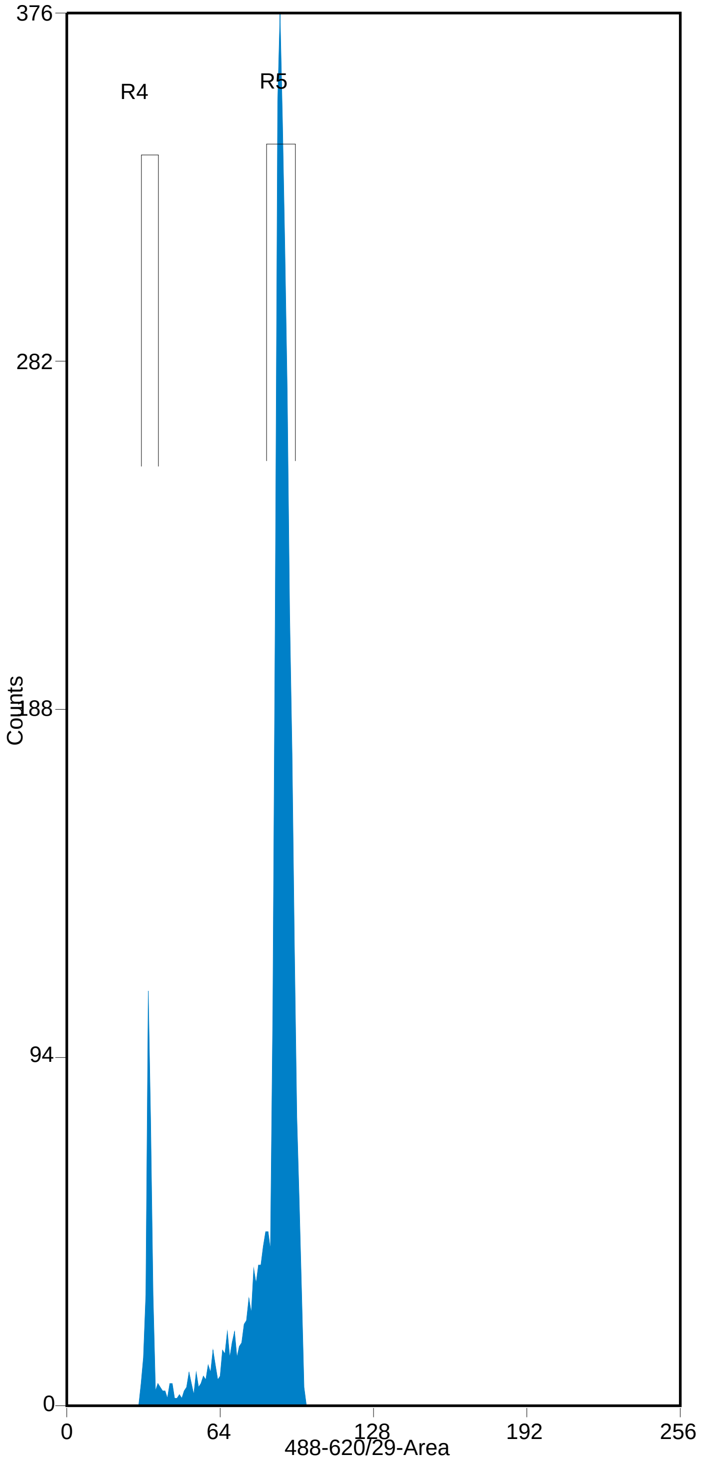

Region	Count	% Hist	Mean	CV
Total	3676	100.00	82.79	20.06
R4	278	7.56	34.36	3.62
R5	2638	71.76	89.94	3.04

BECKMAN COULTER

spiderling of IIBCELB793_2

ADN_07_Jan_25_16 (G3: R1 & R2)

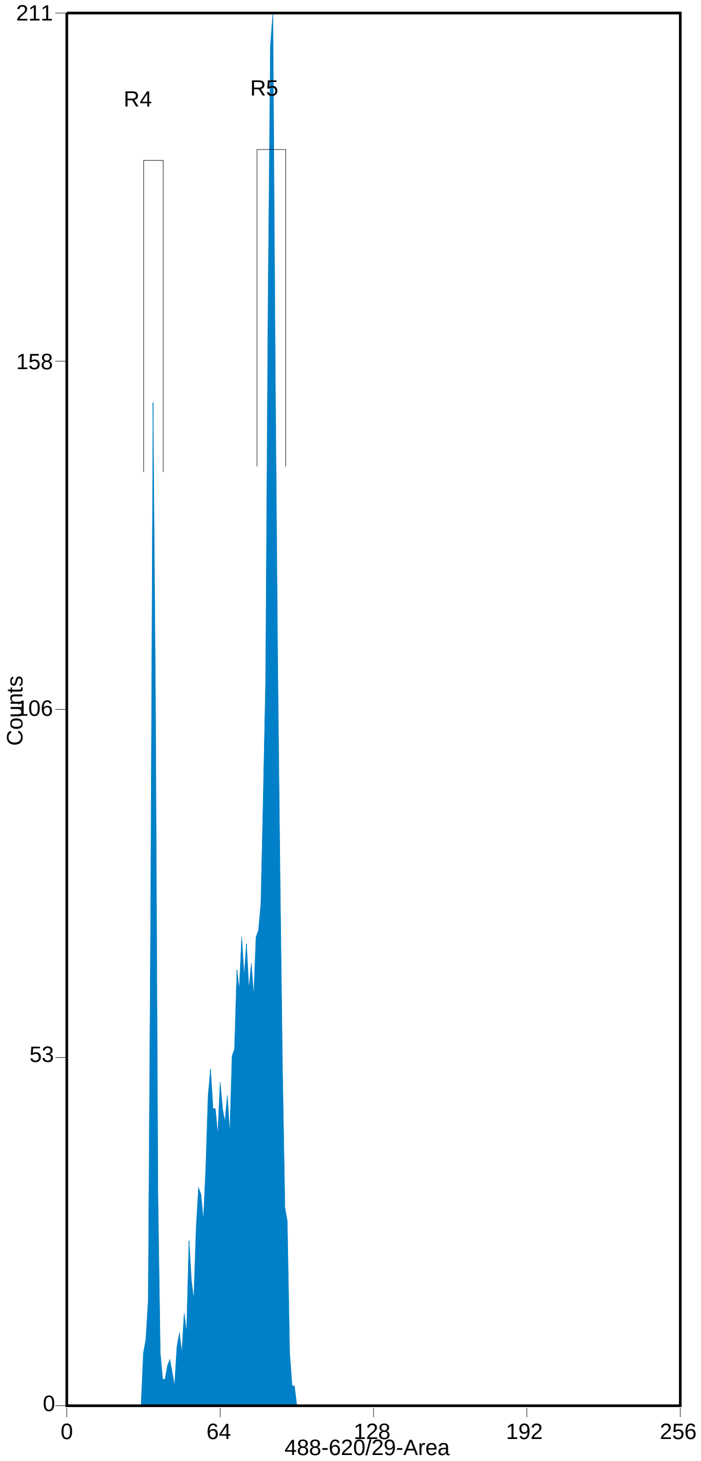

Region	Count	% Hist	Mean	CV
Total	3270	100.00	70.83	24.09
R4	409	12.51	36.11	3.67
R5	1437	43.94	84.91	3.47

BECKMAN COULTER

spiderling of IIBCELB793_3

ADN_07_Jan_25_17 (G3: R1 & R2)

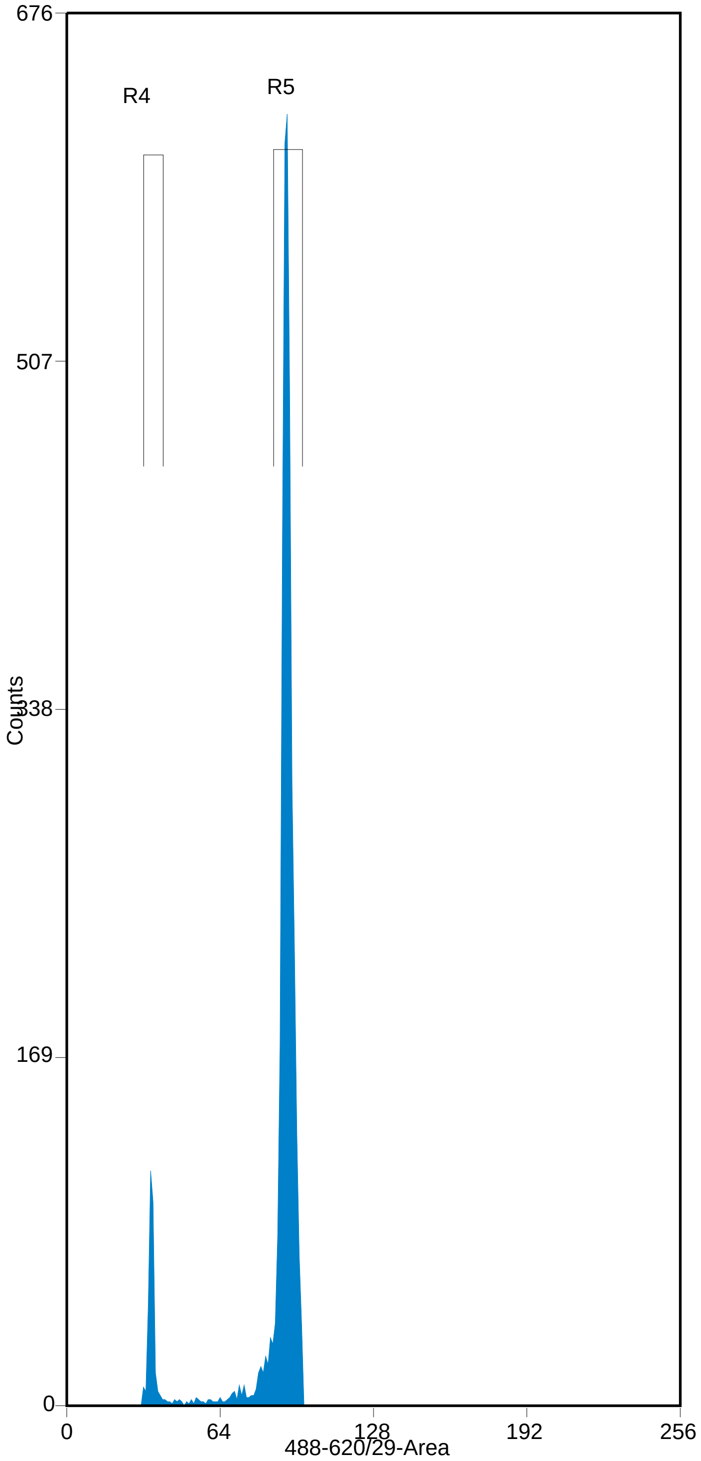

Region	Count	% Hist	Mean	CV
Total	3821	100.00	86.23	18.82
R4	309	8.09	35.31	3.63
R5	3253	85.13	92.06	2.43

spiderling of IIBCELB793_4

ADN_07_Jan_25_18 (G3: R1 & R2)

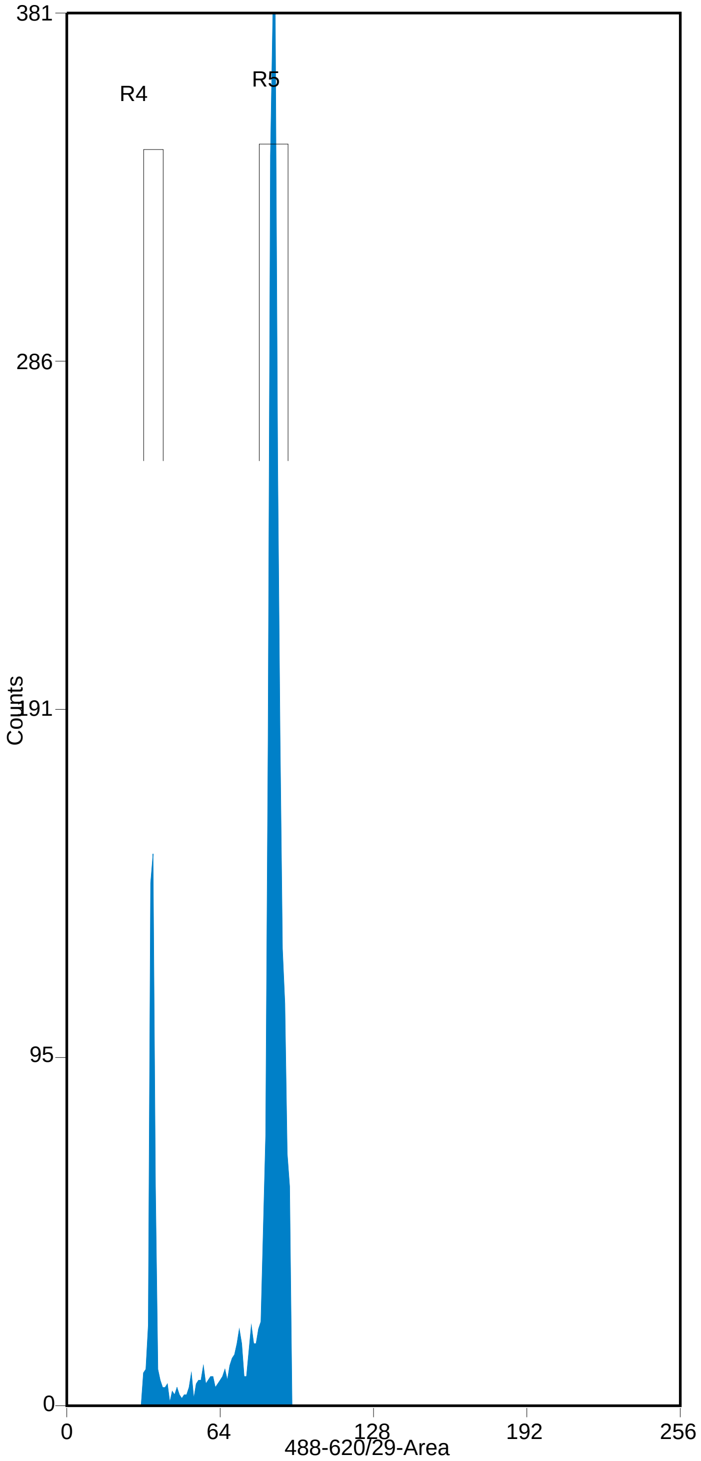

Region	Count	% Hist	Mean	CV
Total	3107	100.00	77.71	23.49
R4	417	13.42	35.68	3.54
R5	2296	73.90	86.69	2.75

spiderling of IIBCELB793_5

ADN_07_Jan_25_19 (G3: R1 & R2)

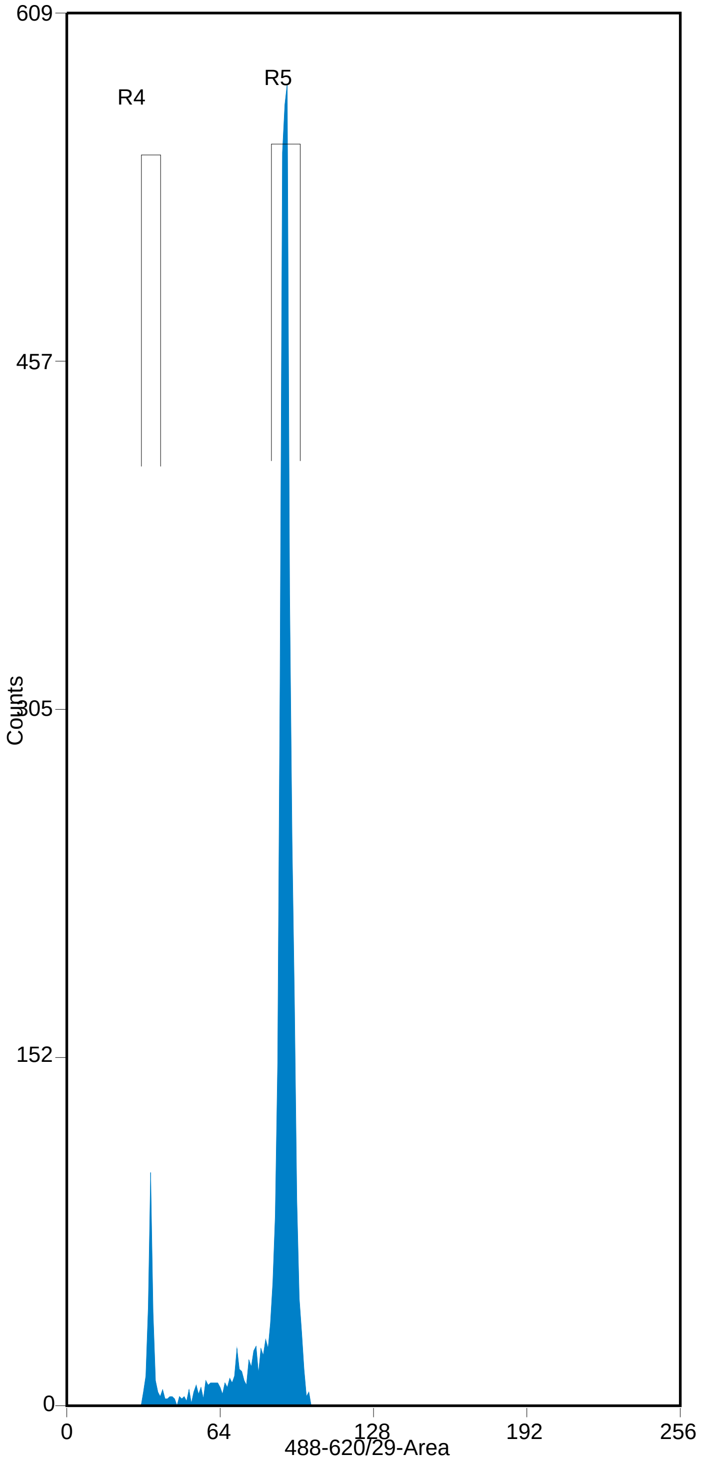

Region	Count	% Hist	Mean	CV
Total	4015	100.00	85.69	17.42
R4	226	5.63	35.04	3.56
R5	3251	80.97	91.32	2.56

BECKMAN COULTER

spiderling of IIBCELB793_6

ADN_07_Jan_25_20 (G3: R1 & R2)

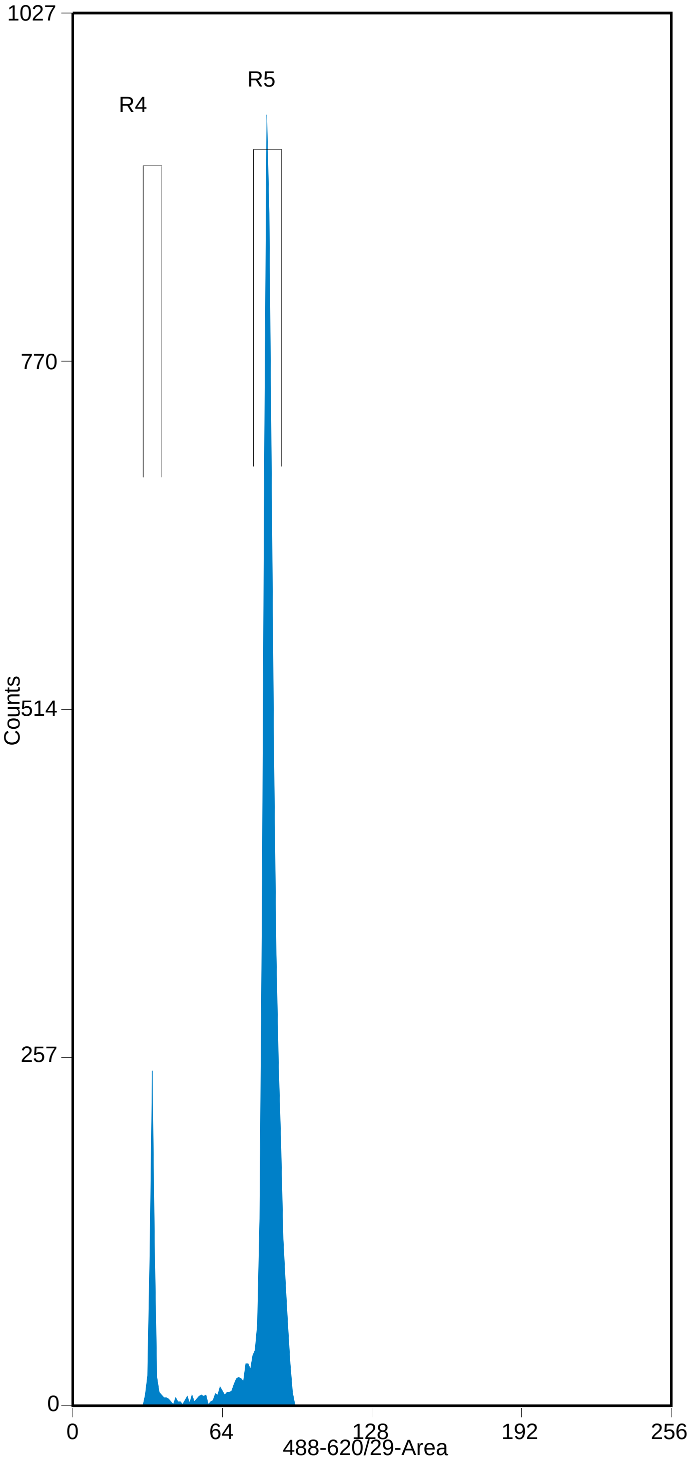

Region	Count	% Hist	Mean	CV
Total	6336	100.00	78.93	18.92
R4	543	8.57	34.07	3.30
R5	5113	80.70	83.97	2.81

spiderling of IIBCELB793_7

ADN_07_Jan_25_21 (G3: R1 & R2)

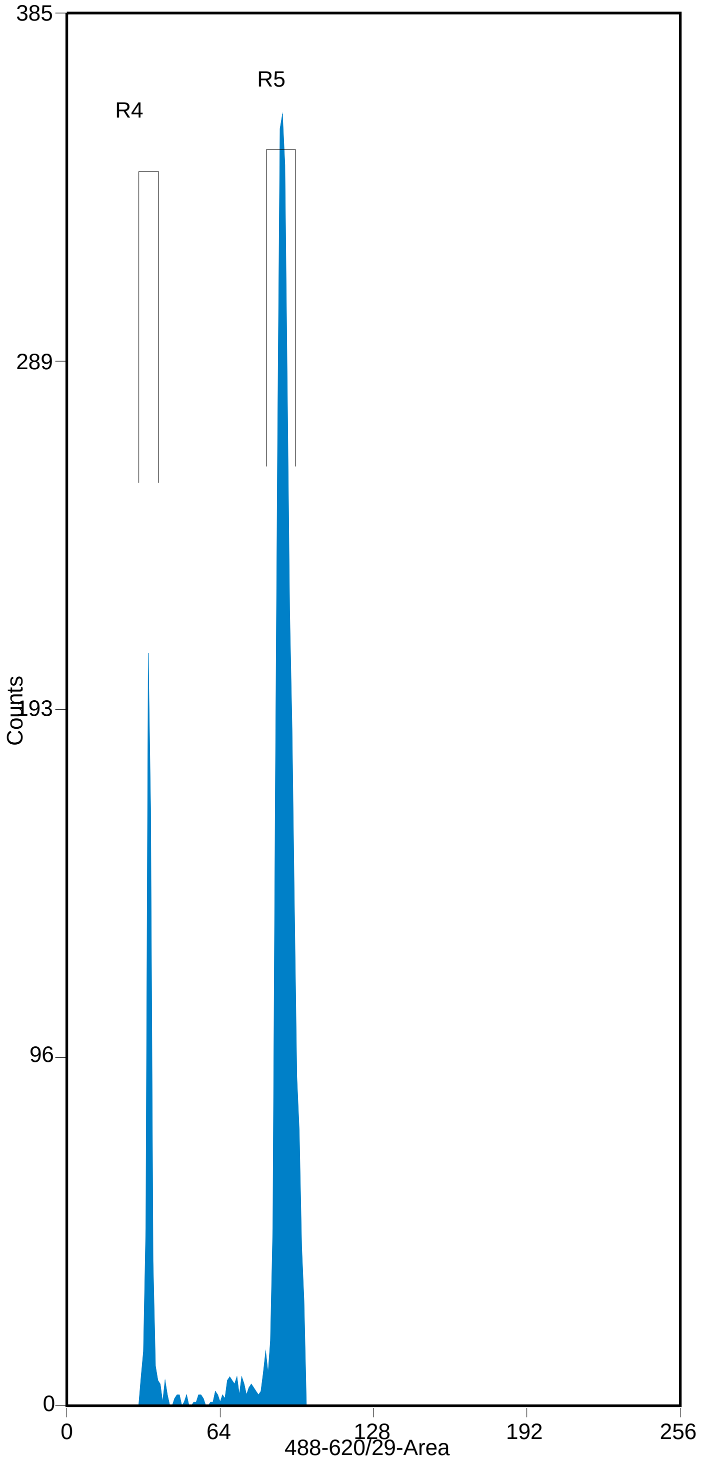

Region	Count	% Hist	Mean	CV
Total	3298	100.00	81.30	25.75
R4	503	15.25	34.40	3.27
R5	2409	73.04	90.48	2.72

spiderling of IIBCELB793_8

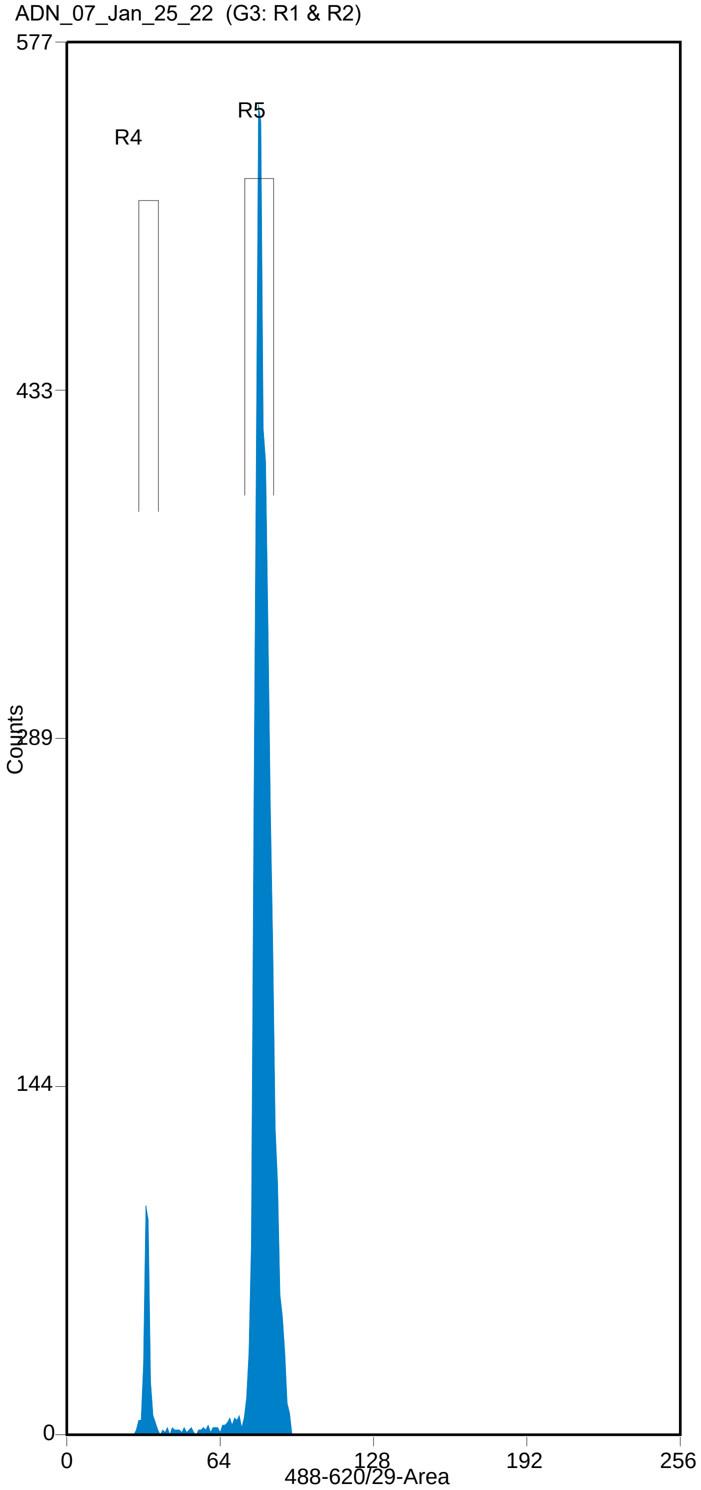

Region	Count	% Hist	Mean	CV
Total	4247	100.00	78.65	15.95
R4	262	6.17	33.49	3.80
R5	3491	82.20	81.45	3.01

spiderling of IIBCELB793_9

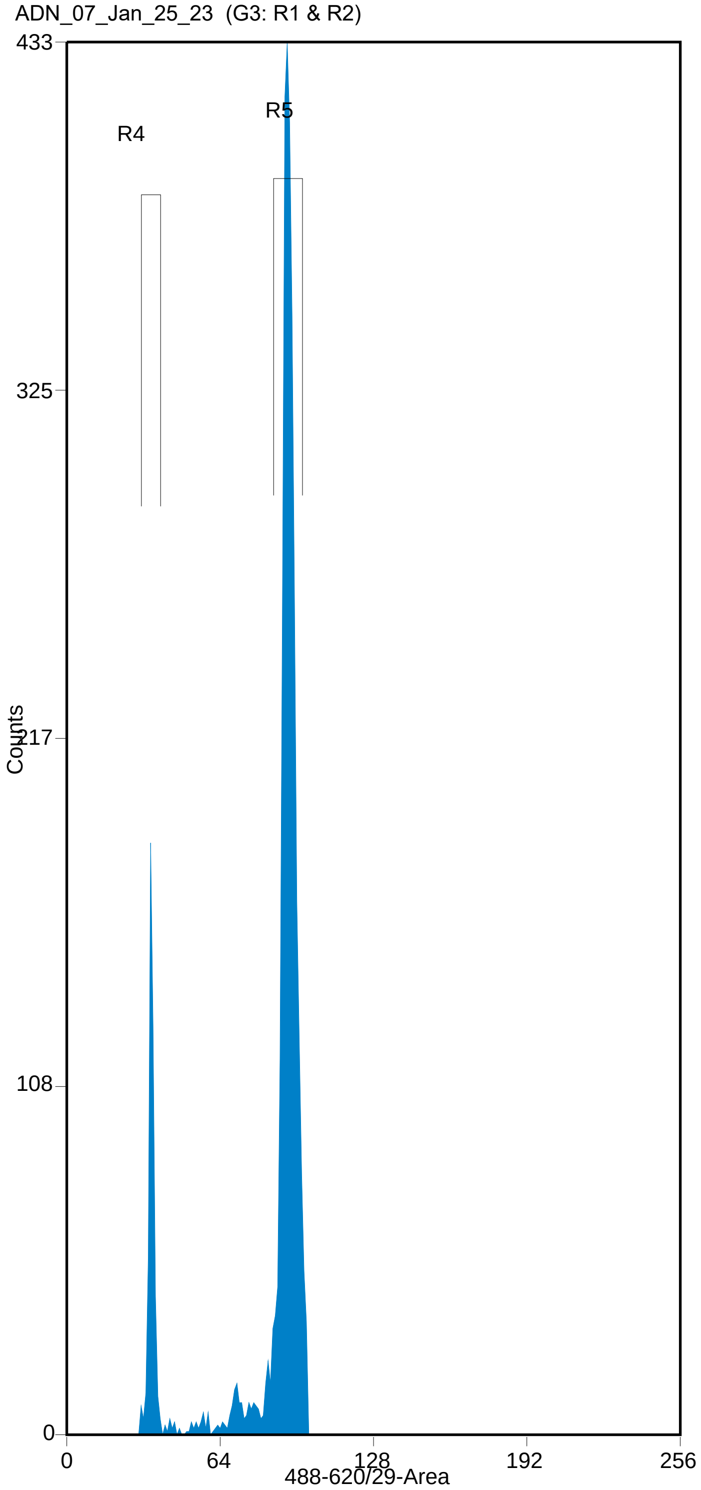

Region	Count	% Hist	Mean	CV
Total	3550	100.00	83.96	23.61
R4	453	12.76	35.30	3.62
R5	2756	77.63	92.66	2.68

spiderling of IIBCELB793_10

ADN_07_Jan_25_24 (G3: R1 & R2)

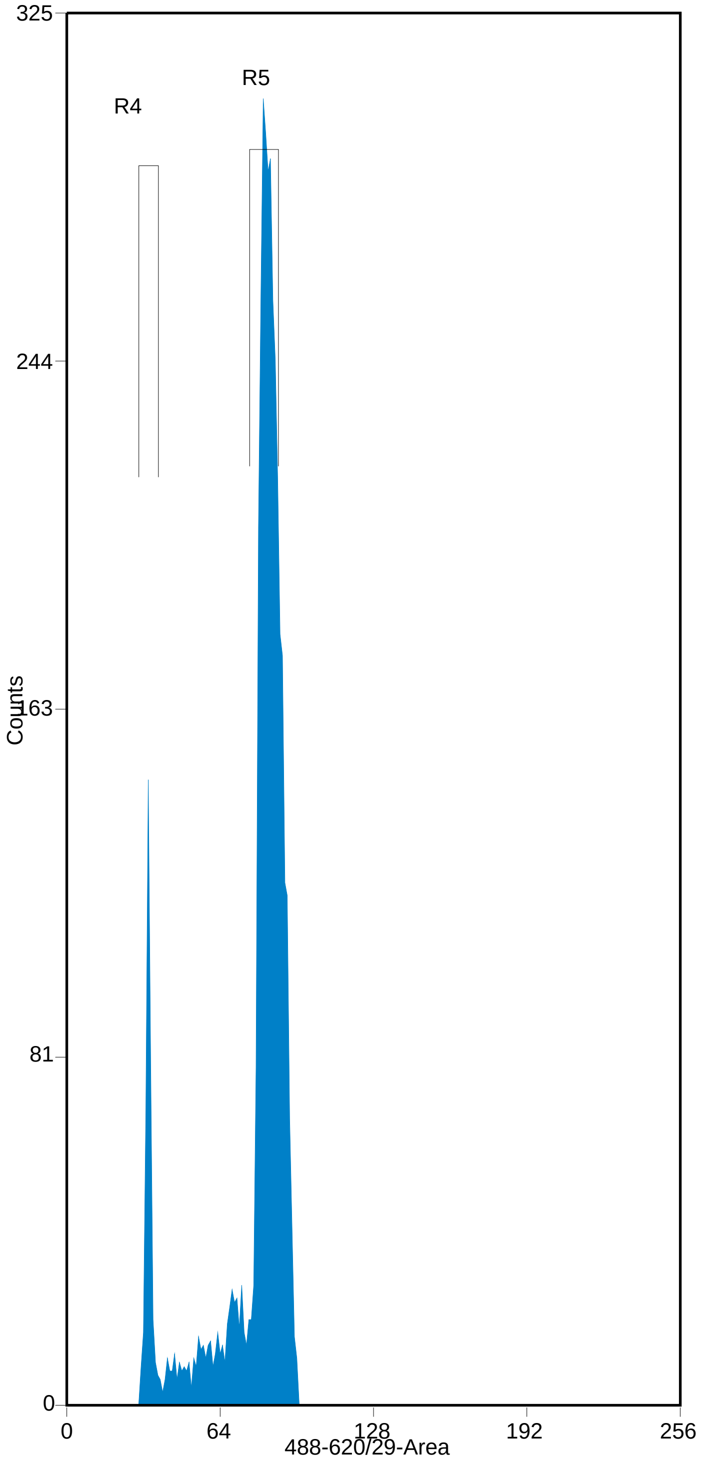

Region	Count	% Hist	Mean	CV
Total	4081	100.00	77.91	21.10
R4	359	8.80	34.14	3.71
R5	2510	61.50	83.62	3.29

spiderling of IIBCELB793_11

ADN_07_Jan_25_25 (G3: R1 & R2)

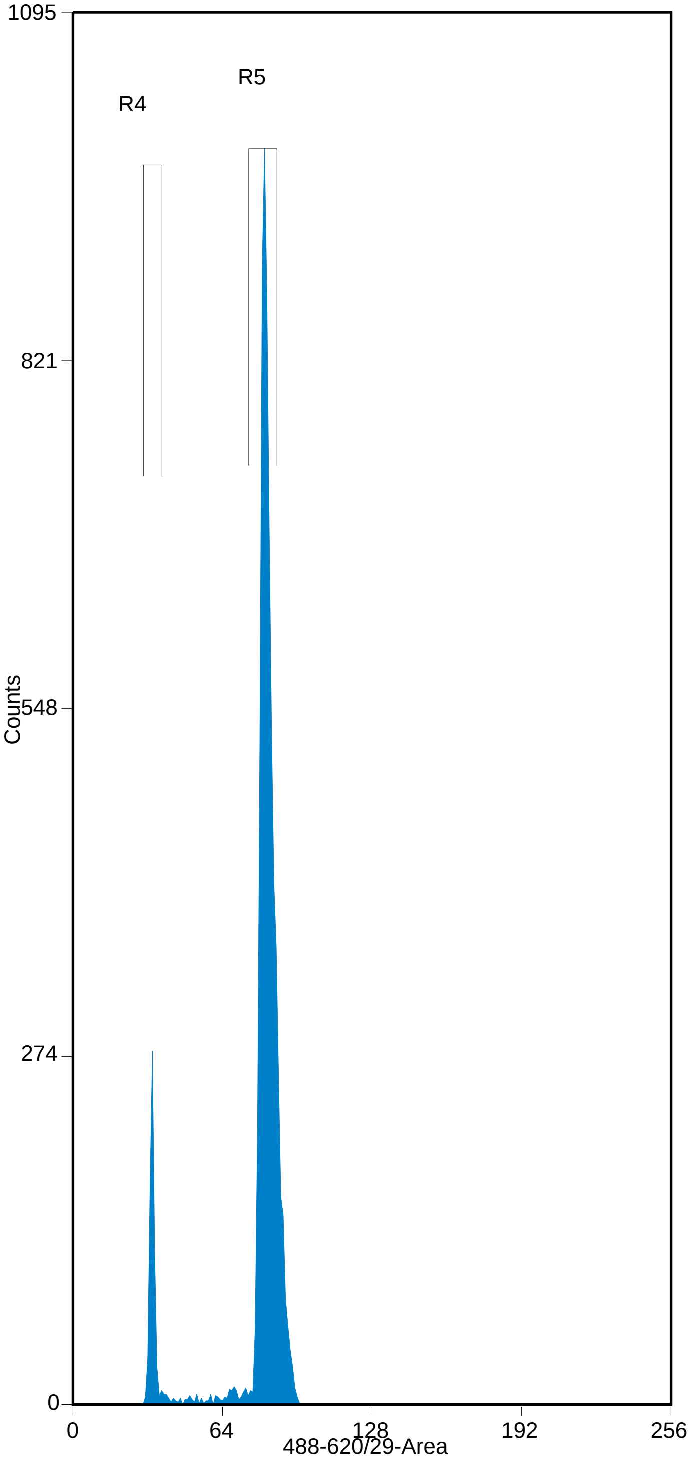

Region	Count	% Hist	Mean	CV
Total	7248	100.00	78.48	19.17
R4	659	9.09	33.96	3.34
R5	5580	76.99	82.77	2.69

spiderling of IIBCELB793_12

ADN_07_Jan_25_26 (G3: R1 & R2)

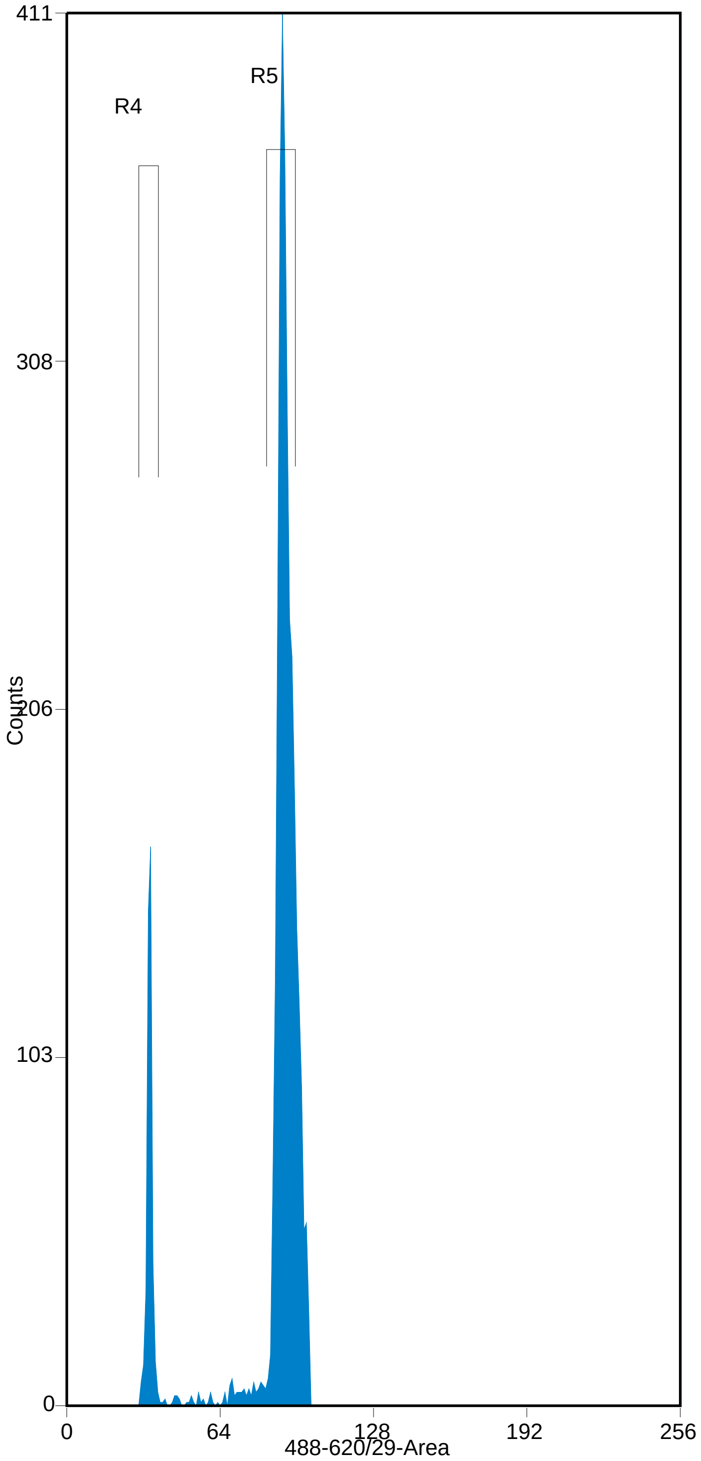

Region	Count	% Hist	Mean	CV
Total	3568	100.00	84.28	22.67
R4	423	11.86	34.53	3.27
R5	2545	71.33	90.71	2.67

spiderling of IIBCELB793_13

ADN_07_Jan_25_28 (G3: R1 & R2)

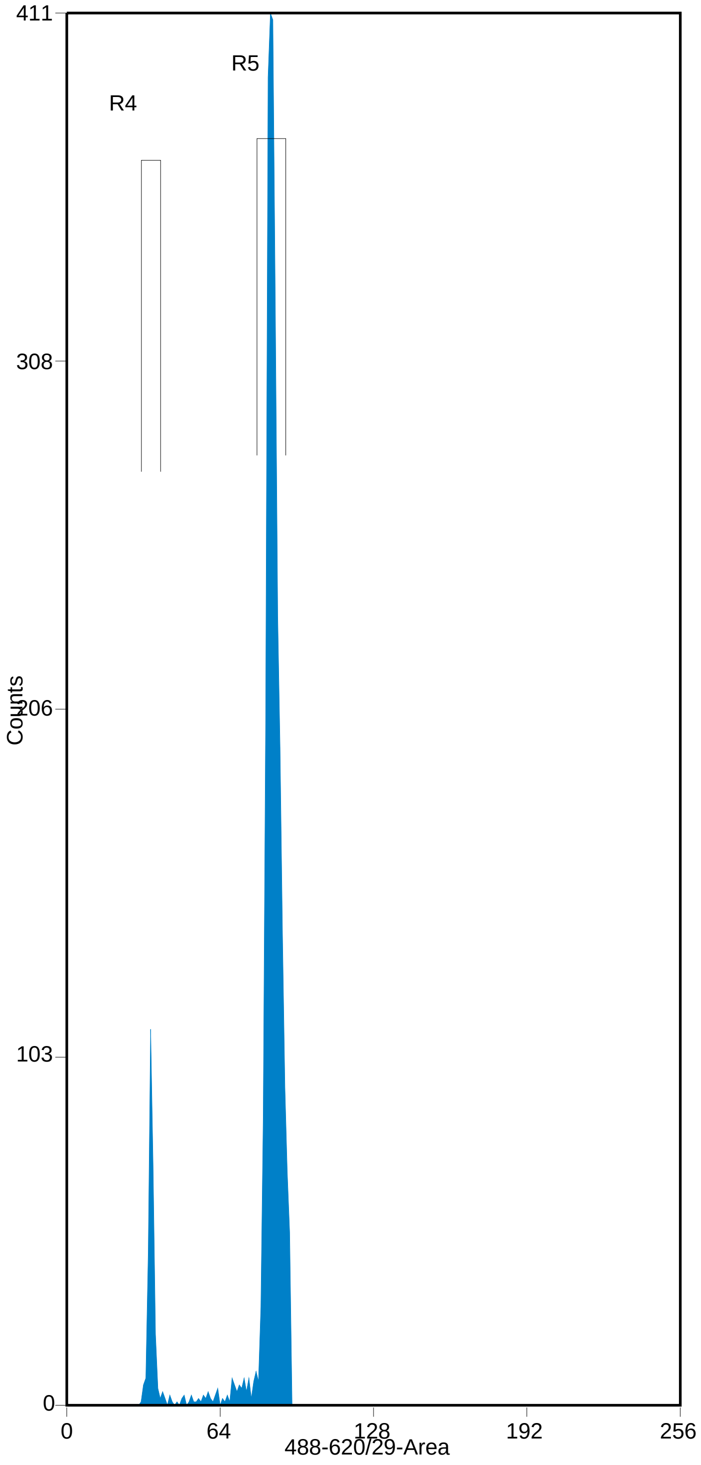

Region	Count	% Hist	Mean	CV
Total	3100	100.00	81.04	18.60
R4	270	8.71	35.20	3.32
R5	2600	83.87	85.97	2.72

BECKMAN COULTER

spiderling of IIBCELB793_14

ADN_07_Jan_25_29 (G3: R1 & R2)

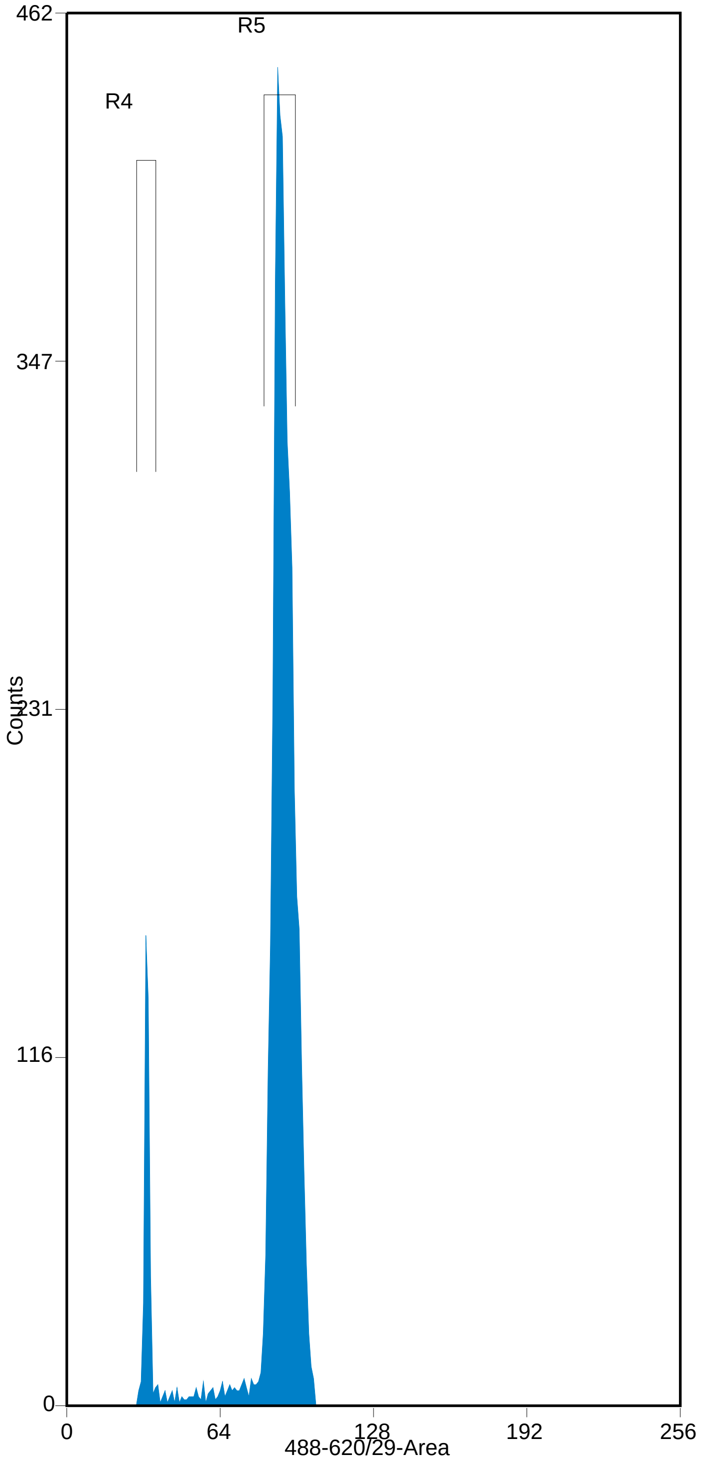

Region	Count	% Hist	Mean	CV
Total	4916	100.00	85.10	19.70
R4	391	7.95	33.49	3.22
R5	3709	75.45	89.65	3.41

ADN_17_Mar_25_1 (G3: R1 & R2)

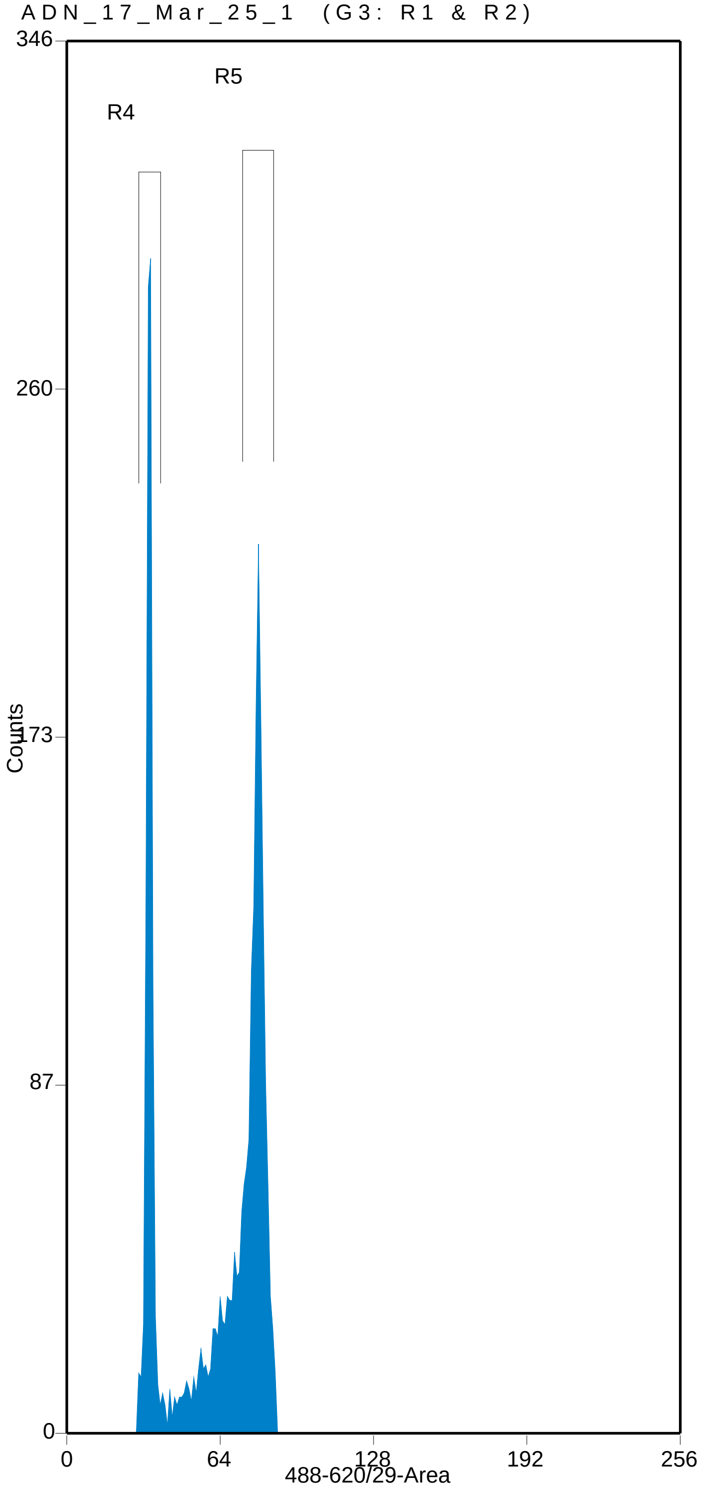

Regi	Cou	% H	Mea	CV
Tot	2	100.	61.	32.
R		30.	34.	4.
R	1	47.	79.	3.

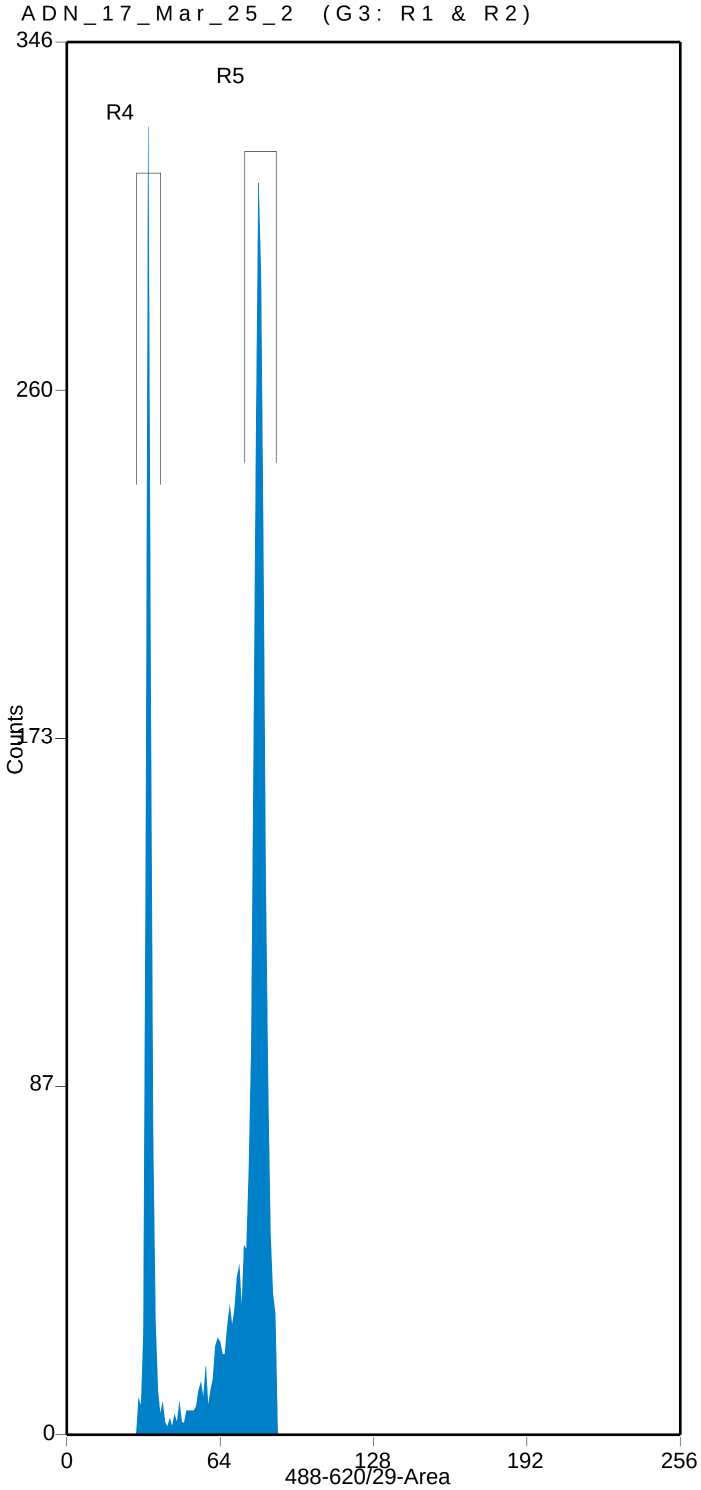

Regi	Cou	% H	Mea	CV
Tot	3	100.	65.	30.
R		25.	34.	3.
R	1	58.	80.	3.

ADN_17_Mar_25_3 (G3: R1 & R2)

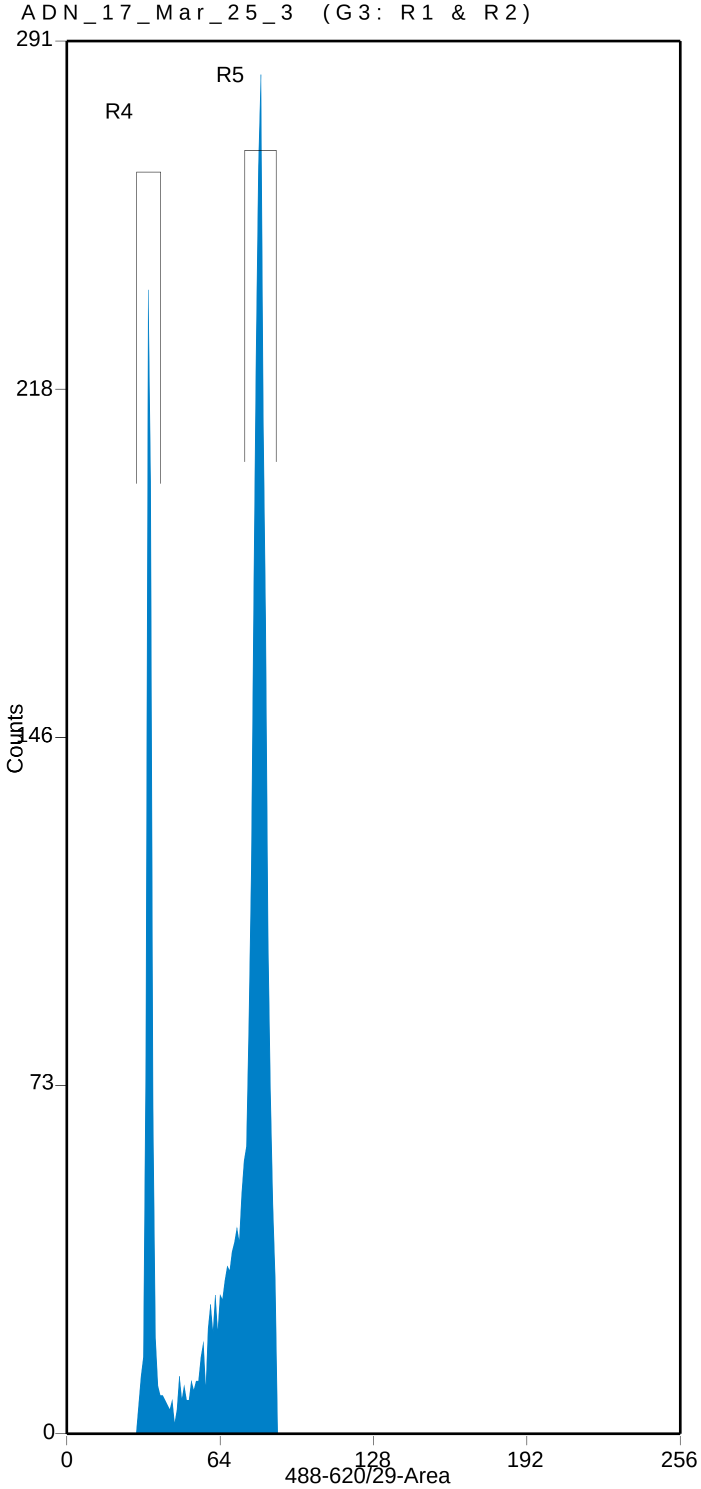

Regi	Cou	% H	Mea	CV
Tot	3	100.	67.	27.
R		20.	34.	3.
R	1	59.	80.	3.

ADN_17_Mar_25_4 (G3: R1 & R2)

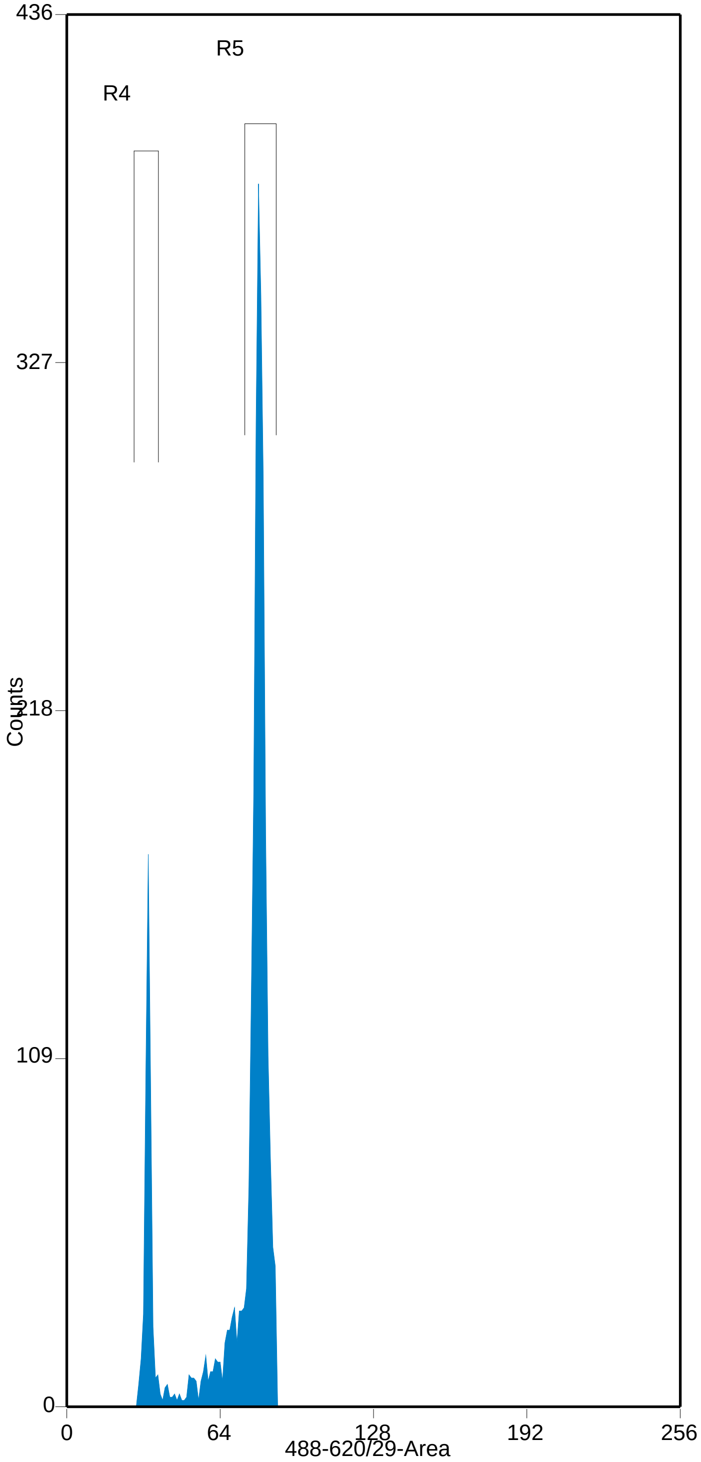

Regi	Cou	% H	Mea	CV
Tot	3	100.	71.	24.
R		15.	33.	4.
R	2	71.	80.	3.

ADN_17_Mar_25_5   (G3: R1 & R2)

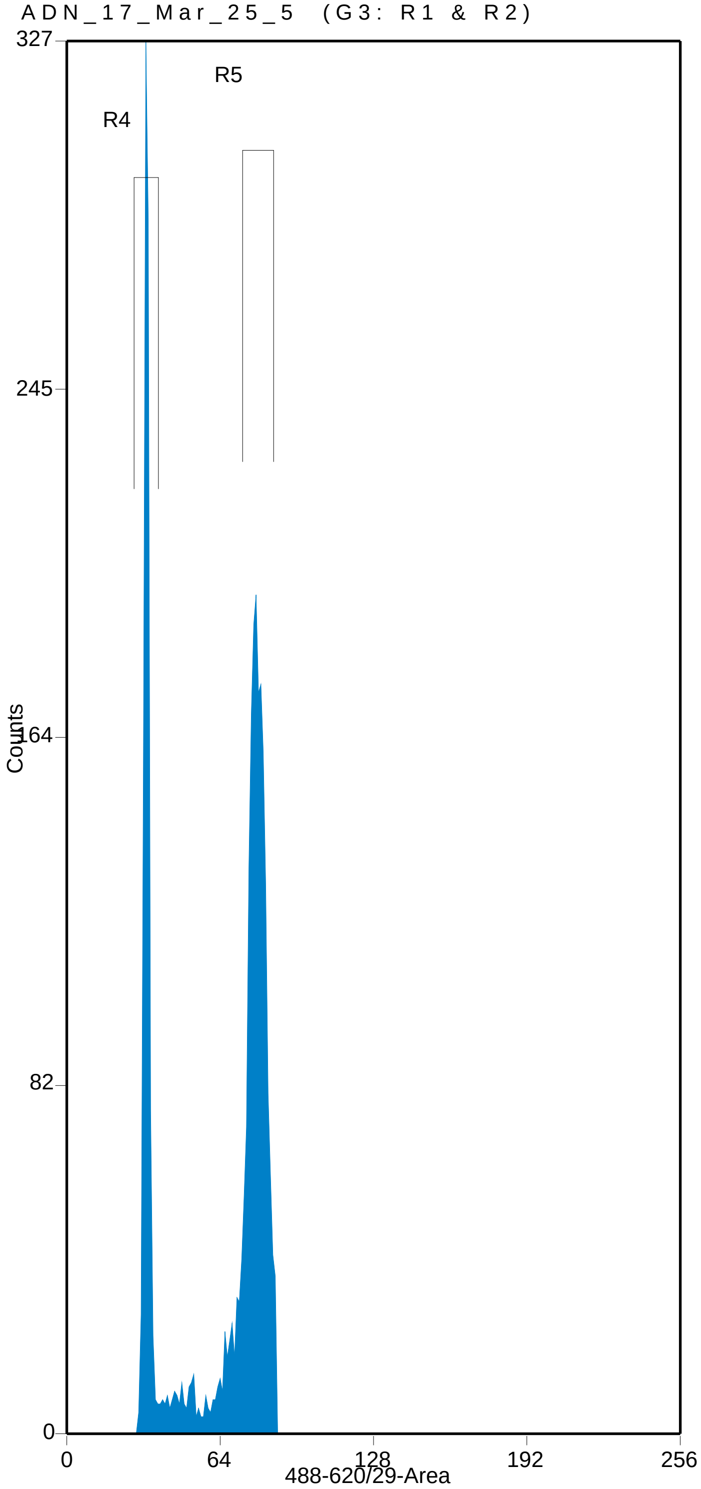

Regi	Cou	% H	Mea	CV
Tot	3	100.	62.	34.
R		31.	33.	3.
R	1	54.	79.	3.

ADN_17_Mar_25_6   (G3: R1 & R2)

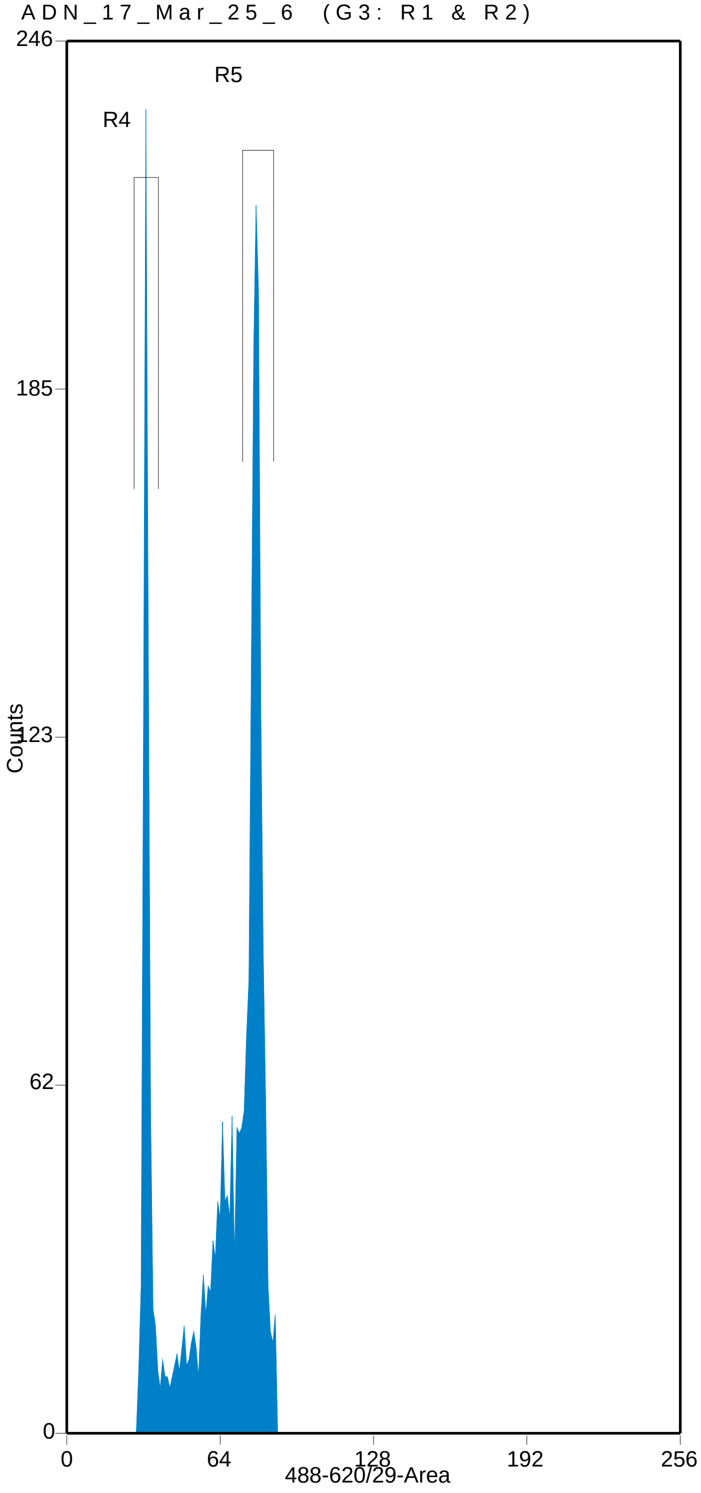

Regi	Cou	% H	Mea	CV
Tot	2	100.	63.	29.
R		22.	33.	4.
R	1	46.	78.	3.

ADN_17_Mar_25_7 (G3: R1 & R2)

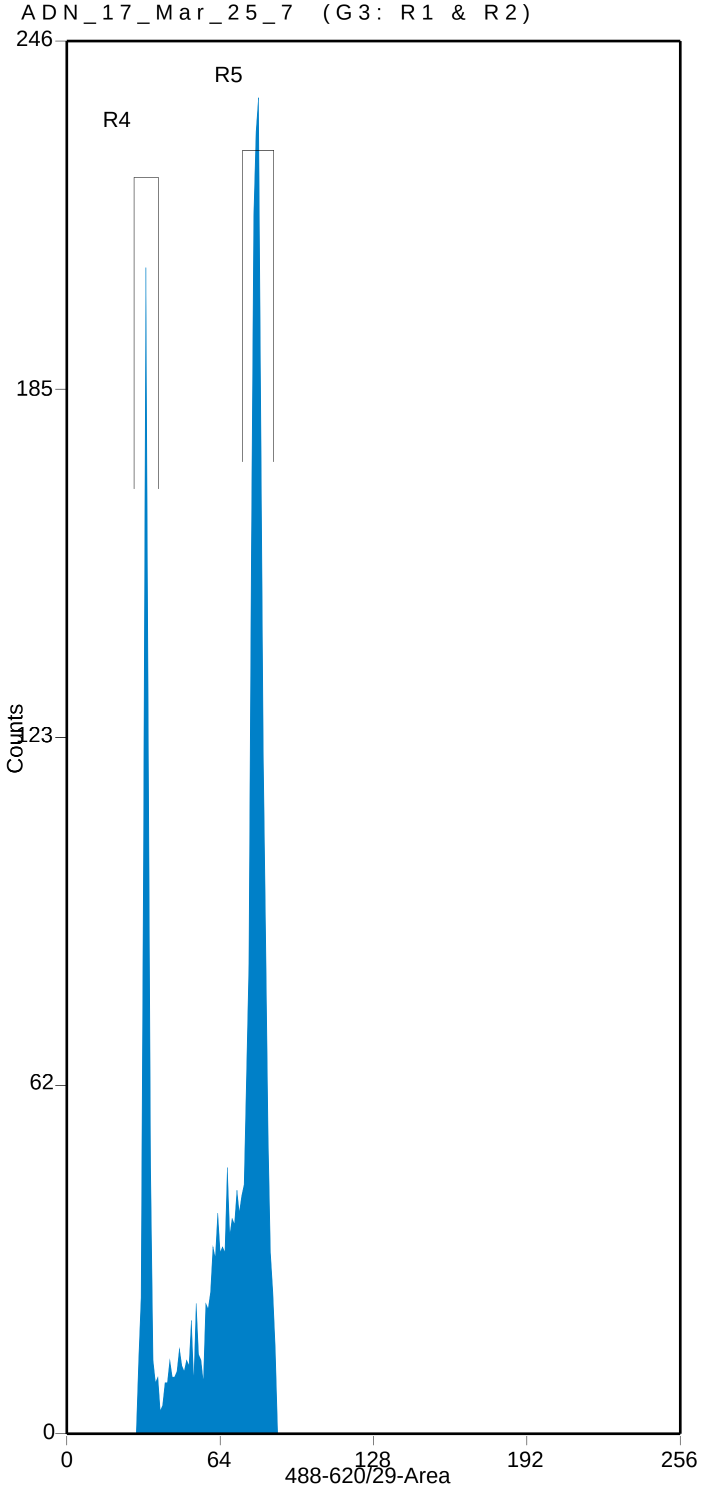

Regi[illegible]	Cou[illegible]	% H[illegible]	Mea[illegible]	CV[illegible]
Tot[illegible]	2[illegible]	100.[illegible]	65.[illegible]	27.[illegible]
R[illegible]		19.[illegible]	33.[illegible]	4.[illegible]
R[illegible]	1[illegible]	54.[illegible]	79.[illegible]	3.[illegible]

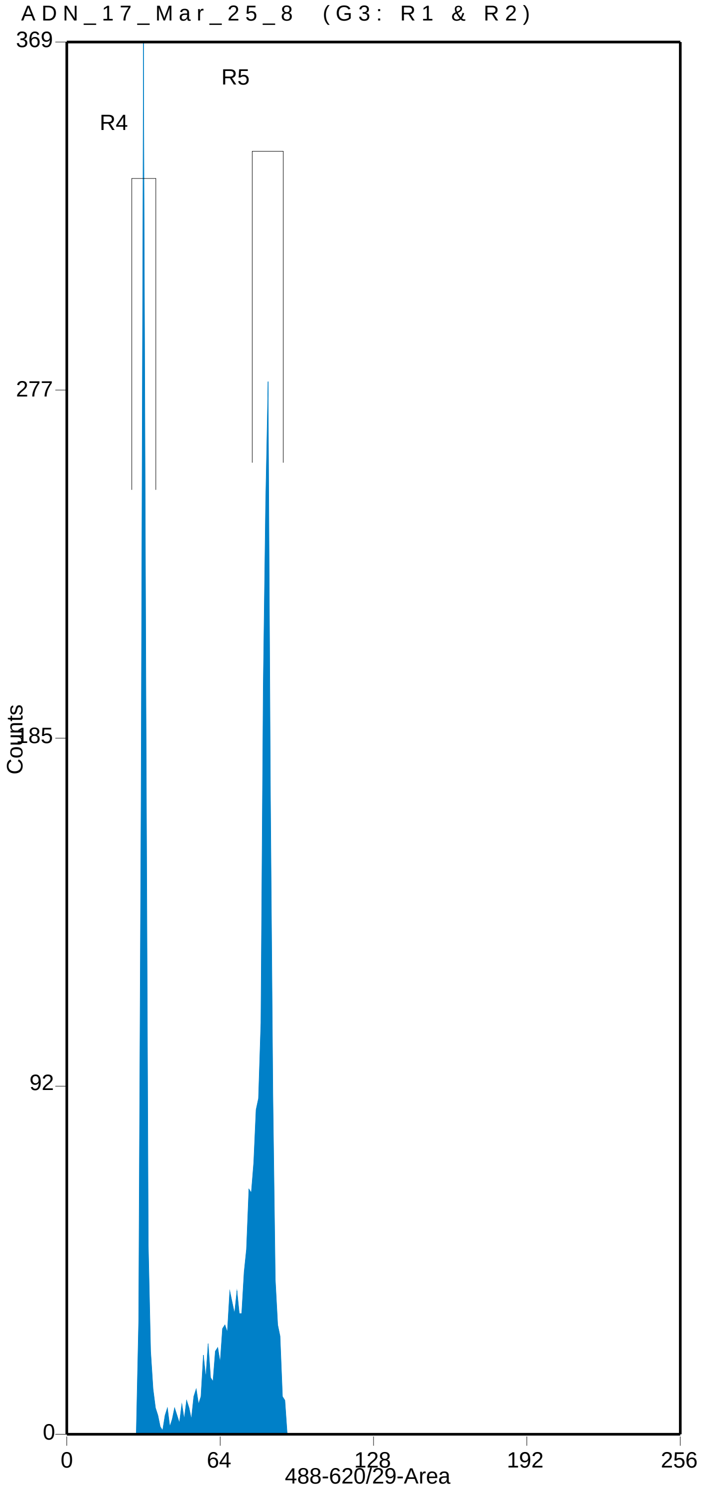

Regi	Cou	% H	Mea	CV
Tot	3	100.	64.	34.
R		28.	32.	3.
R	1	48.	82.	3.

ADN_17_Mar_25_9   (G3: R1 & R2)

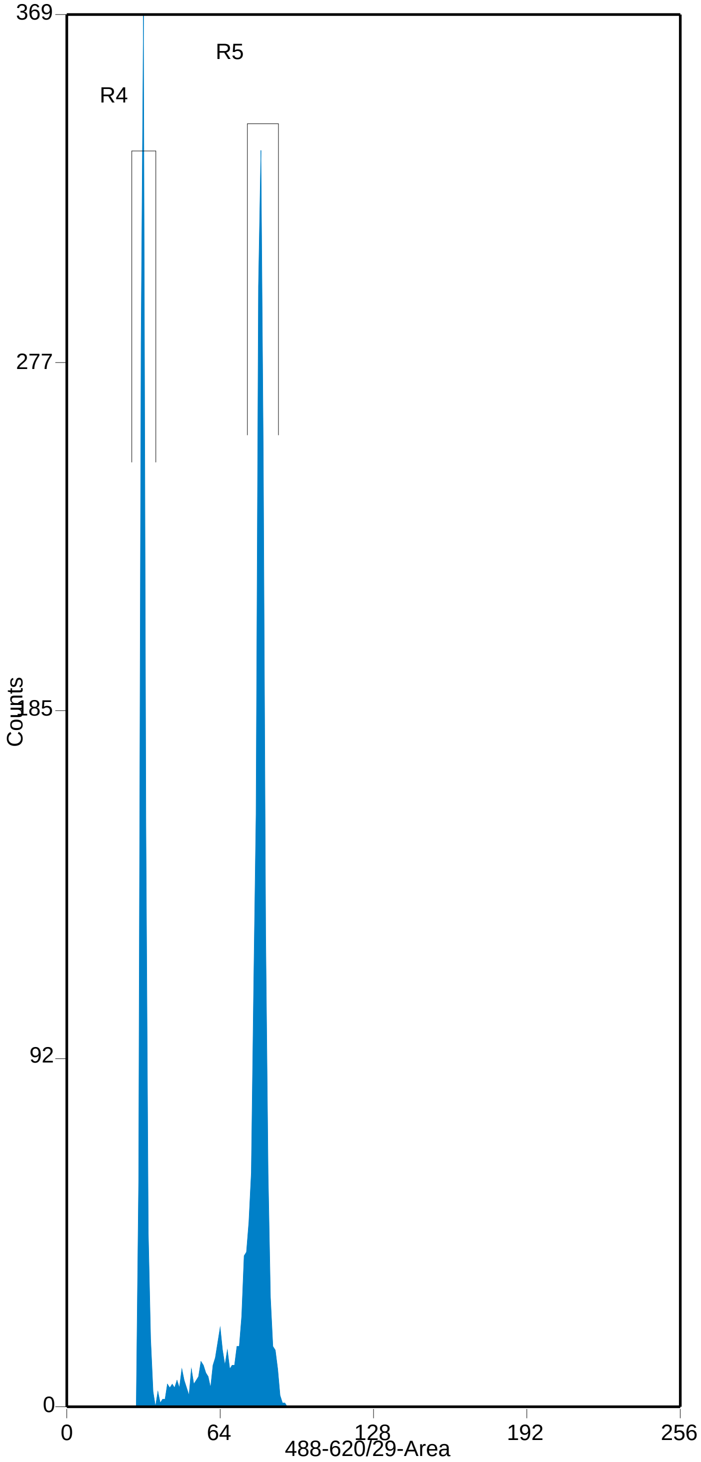

Regi	Cou	% H	Mea	CV
Tot	2	100.	61.	36.
R		33.	31.	3.
R	1	53.	80.	2.

ADN_17_Mar_25_10 (G3: R1 & R2)

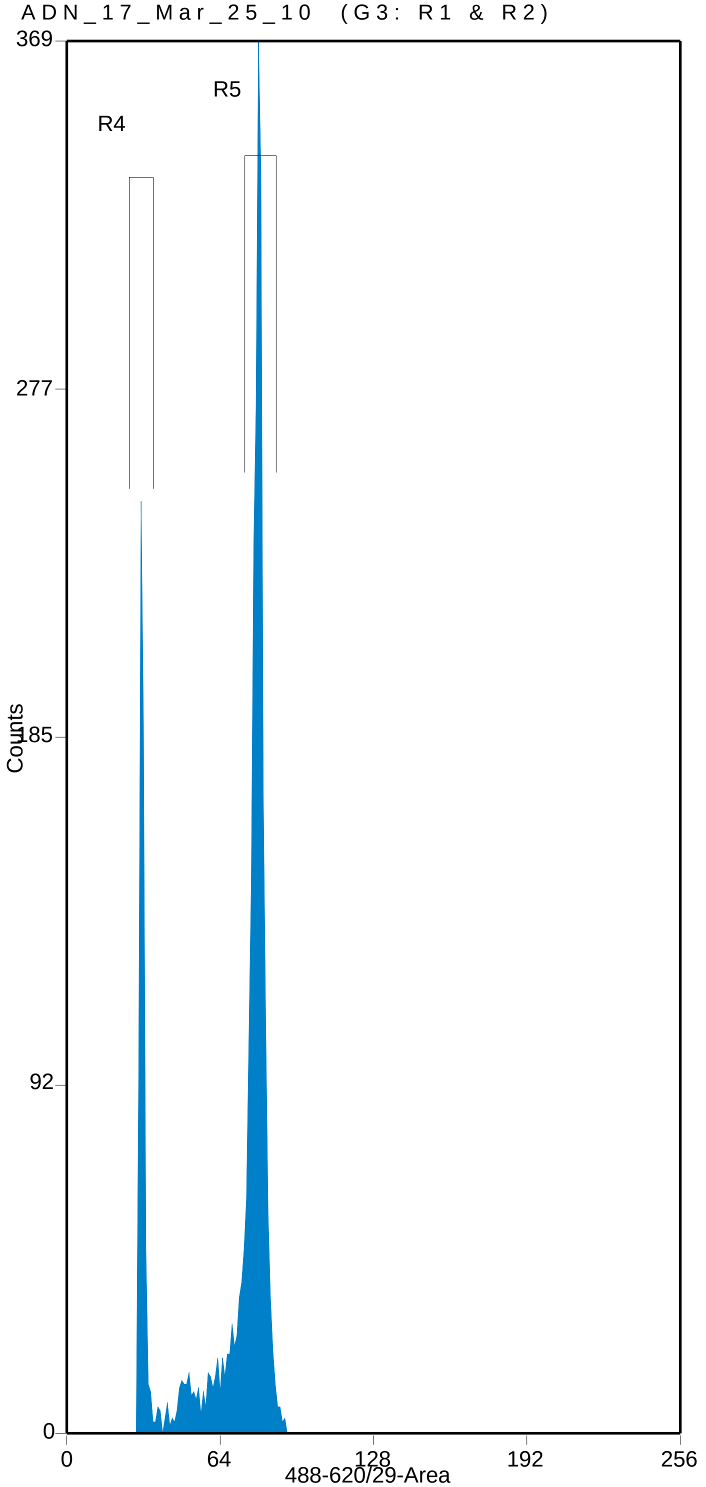

Regi	Cou	% H	Mea	CV
Tot	3	100.	67.	28.
R		19.	31.	3.
R	1	64.	79.	3.

ADN_17_Mar_25_11 (G3: R1 & R2)

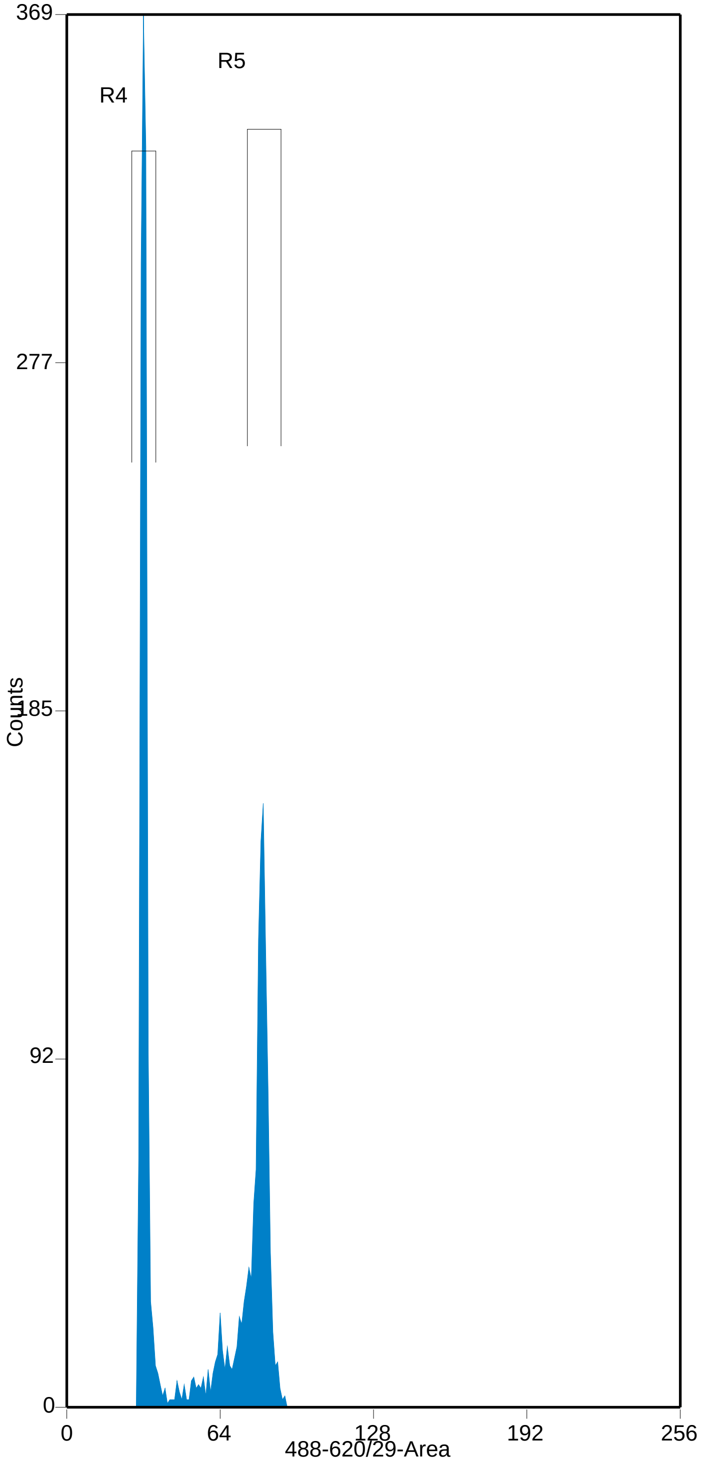

Regi	Cou	% H	Mea	CV
Tot	2	100.	53.	43.
R	1	51.	32.	3.
R		35.	81.	3.

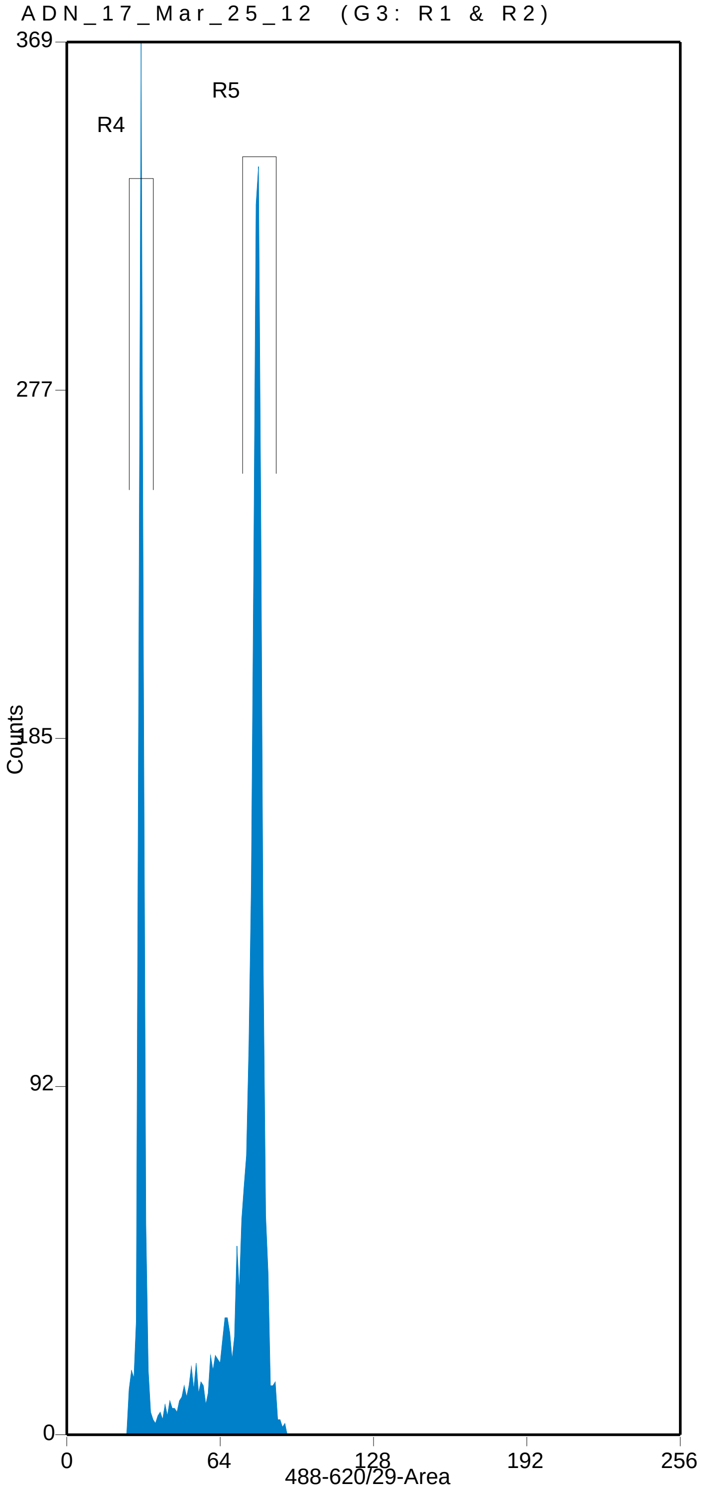

Regi	Cou	% H	Mea	CV
Tot	3	100.	62.	33.
R		27.	30.	4.
R	1	54.	79.	3.

ADN_17_Mar_25_13 (G3: R1 & R2)

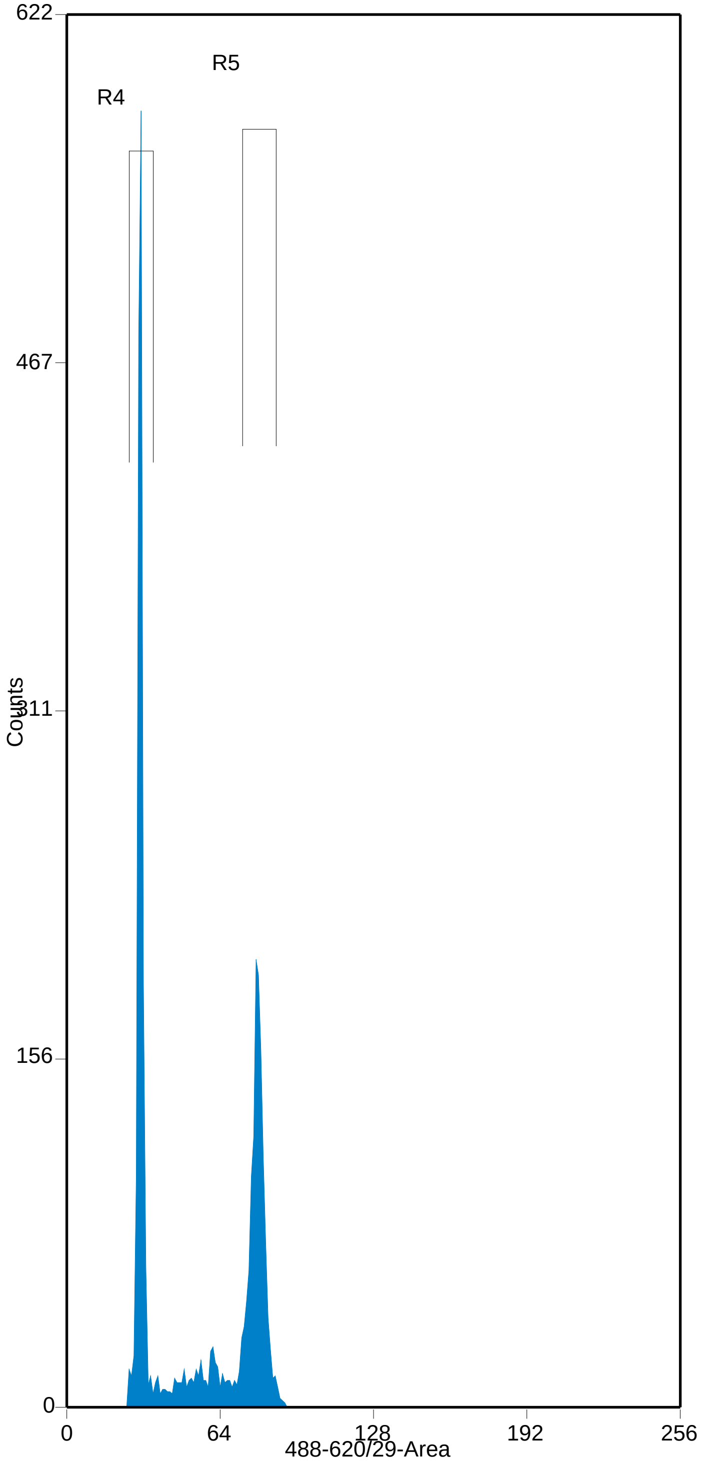

Regi	Cou	% H	Mea	CV
Tot	3	100.	53.	43.
R	1	46.	30.	4.
R	1	38.	79.	3.

ADN_17_Mar_25_14   (G3: R1 & R2)

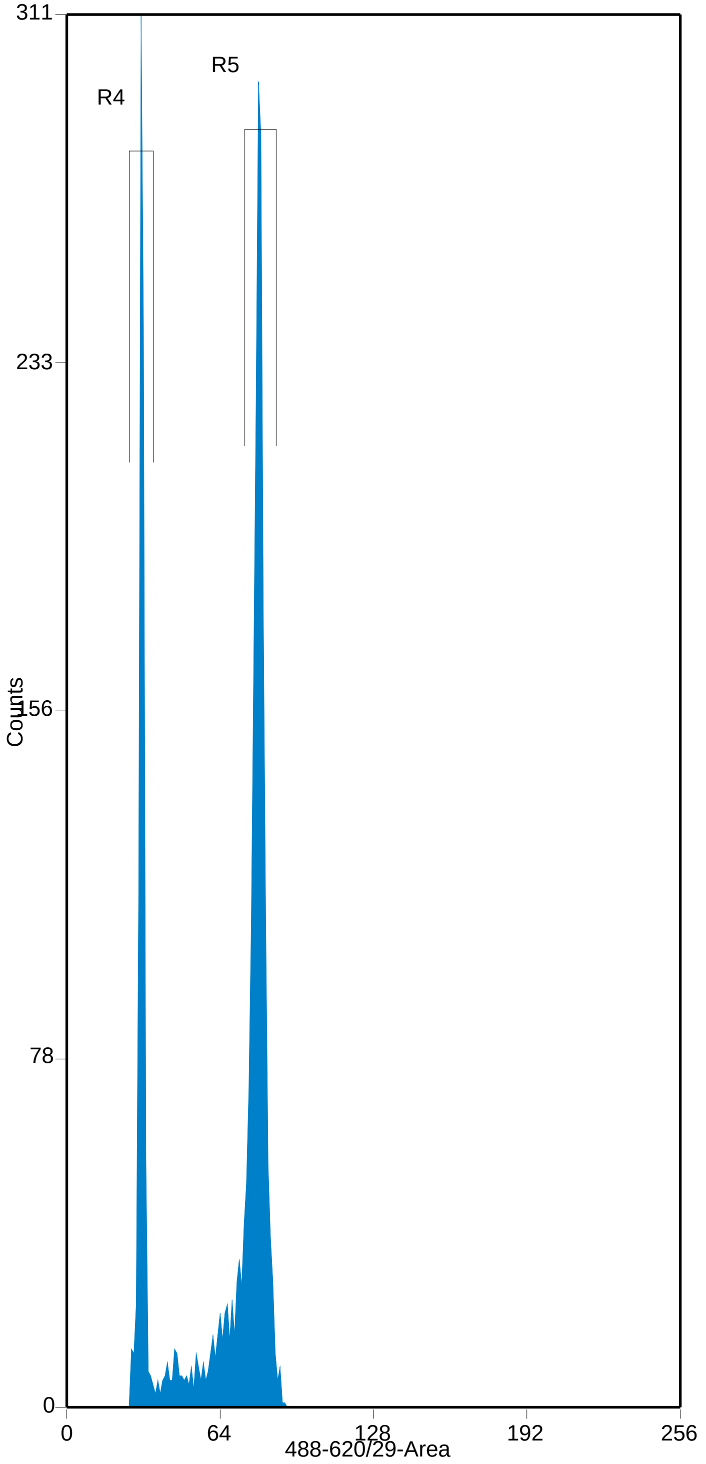

Regi	Cou	% H	Mea	CV
Tot	2	100.	63.	34.
R		28.	31.	3.
R	1	55.	79.	3.

ADN_17_Mar_25_15 (G3: R1 & R2)

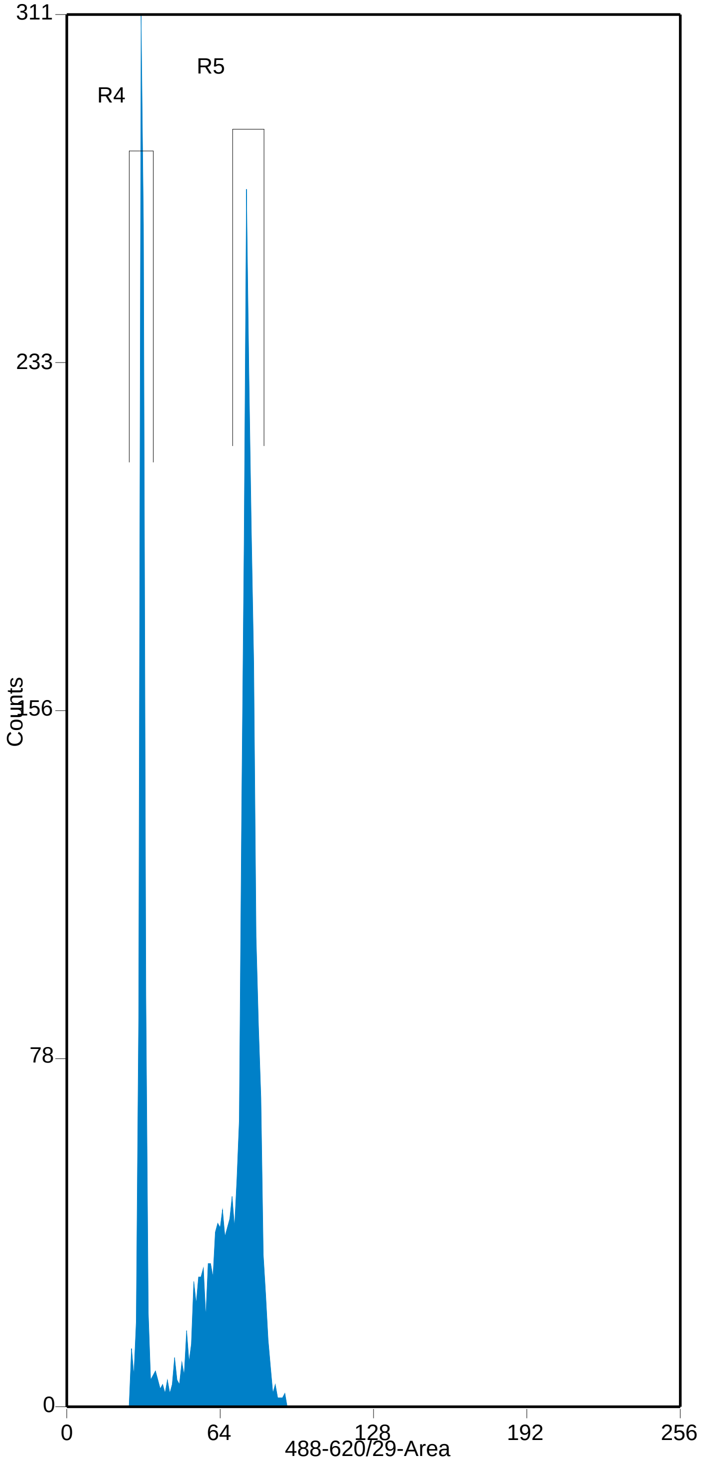

Regi	Cou	% H	Mea	CV
Tot	3	100.	60.	31.
R		26.	31.	4.
R	1	51.	75.	3.

ADN_17_Mar_25_16   (G3: R1 & R2)

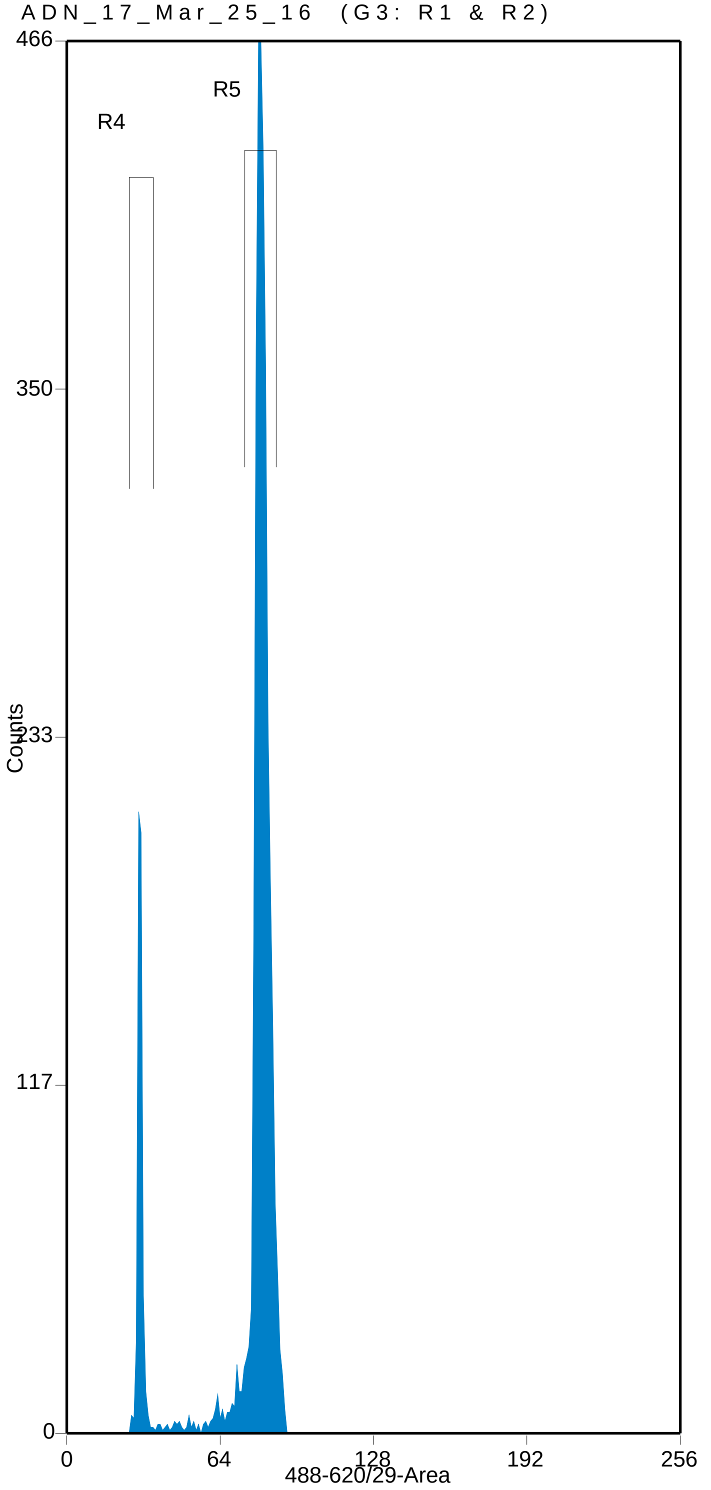

Regi	Cou	% H	Mea	CV
Tot	3	100.	73.	24.
R		13.	30.	3.
R	3	79.	81.	3.

ADN_17_Mar_25_17 (G3: R1 & R2)

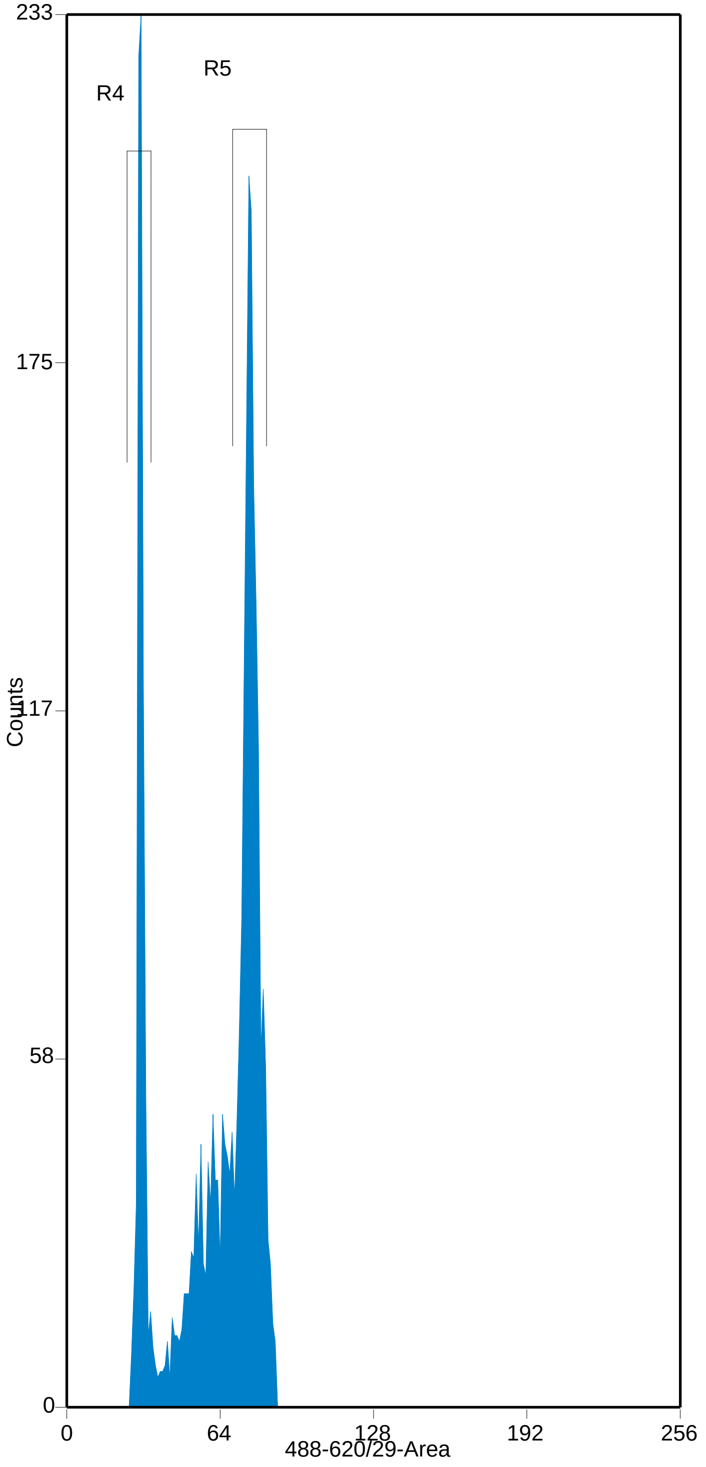

Regi	Cou	% H	Mea	CV
Tot	3	100.	60.	32.
R		24.	30.	4.
R	1	48.	76.	4.

ADN_17_Mar_25_18 (G3: R1 & R2)

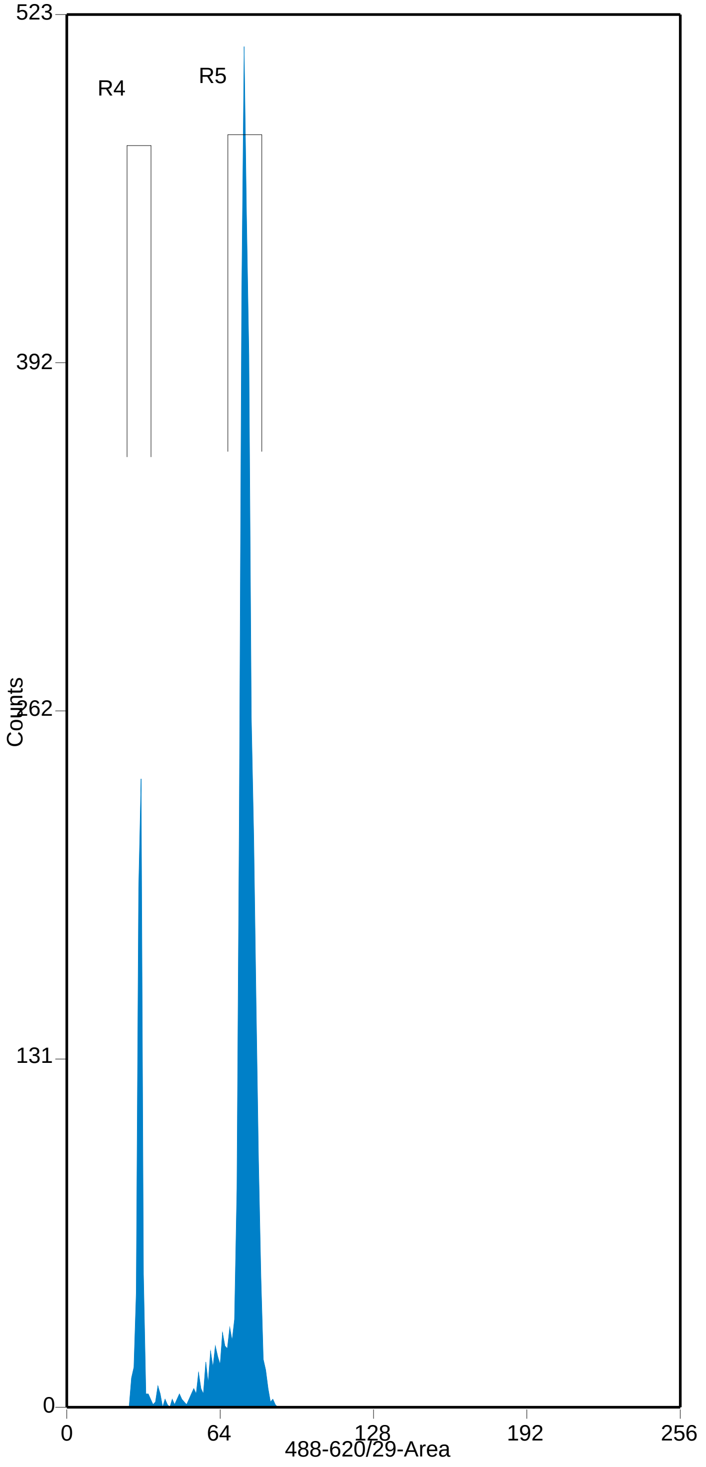

Regi	Cou	% H	Mea	CV
Tot	3	100.	67.	24.
R		14.	30.	3.
R	2	77.	74.	3.

ADN_17_Mar_25_19 (G3: R1 & R2)

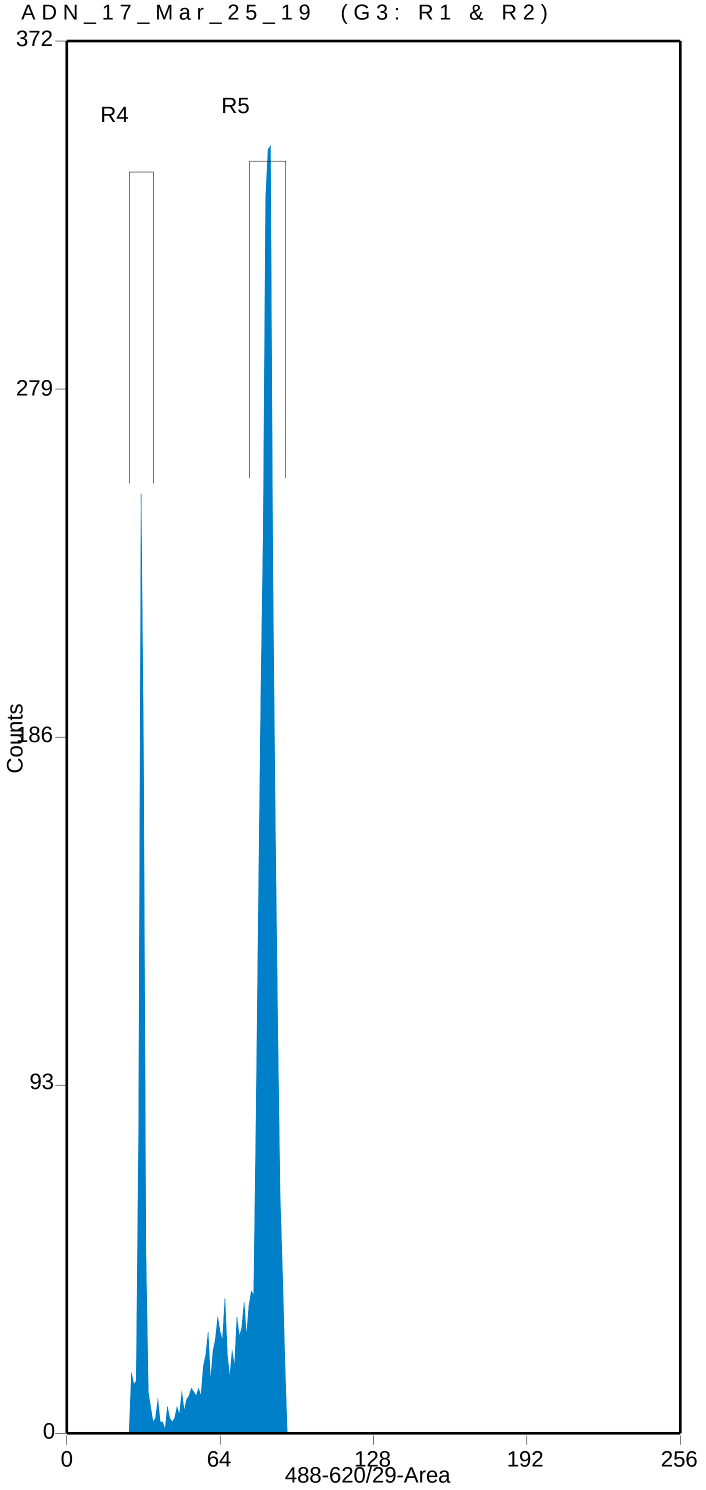

Regi	Cou	% H	Mea	C
Tot	3	100.	71.	28.
R		17.	31.	4.
R	2	66.	83.	3.

ADN_17_Mar_25_20   (G3: R1 & R2)

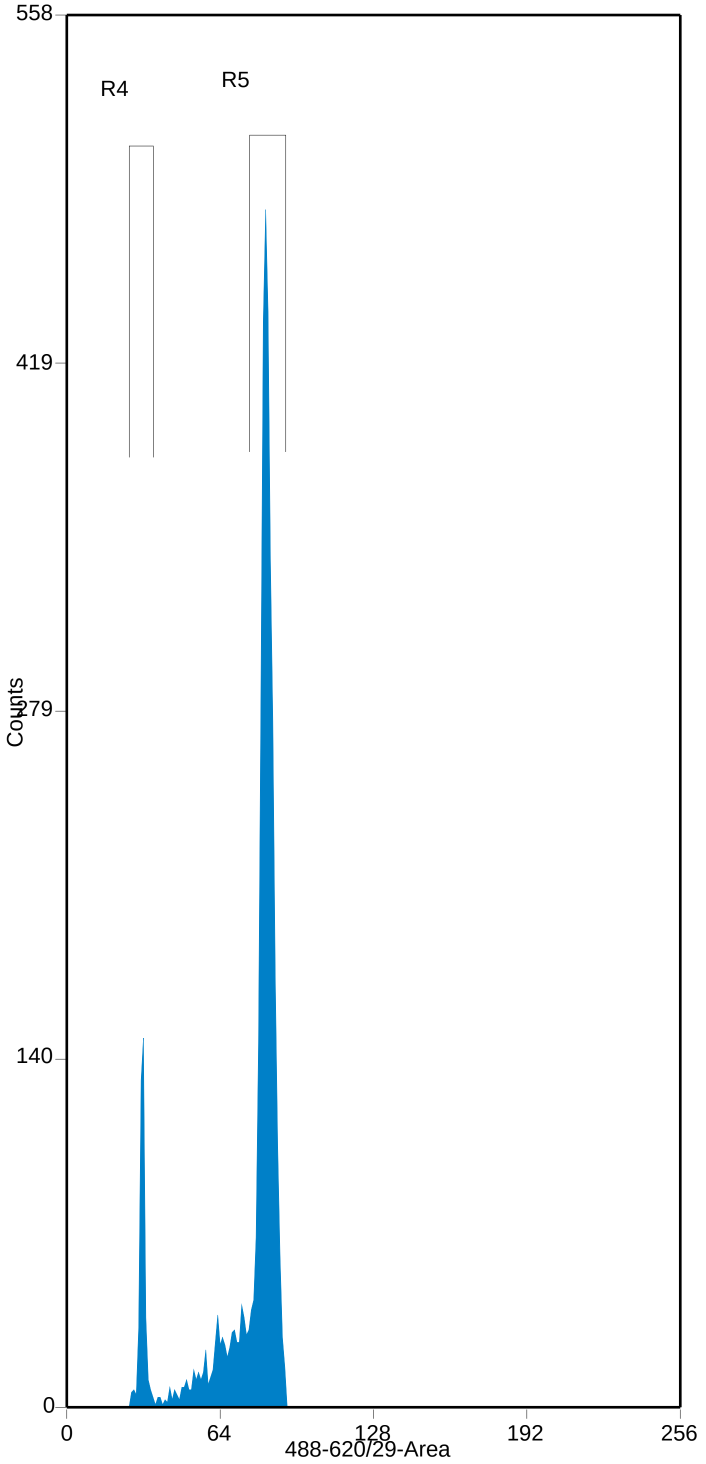

Regi	Cou	% H	Mea	CV
Tot	3	100.	75.	22.
R		9.	31.	4.
R	2	74.	83.	3.

ADN_17_Mar_25_21 (G3: R1 & R2)

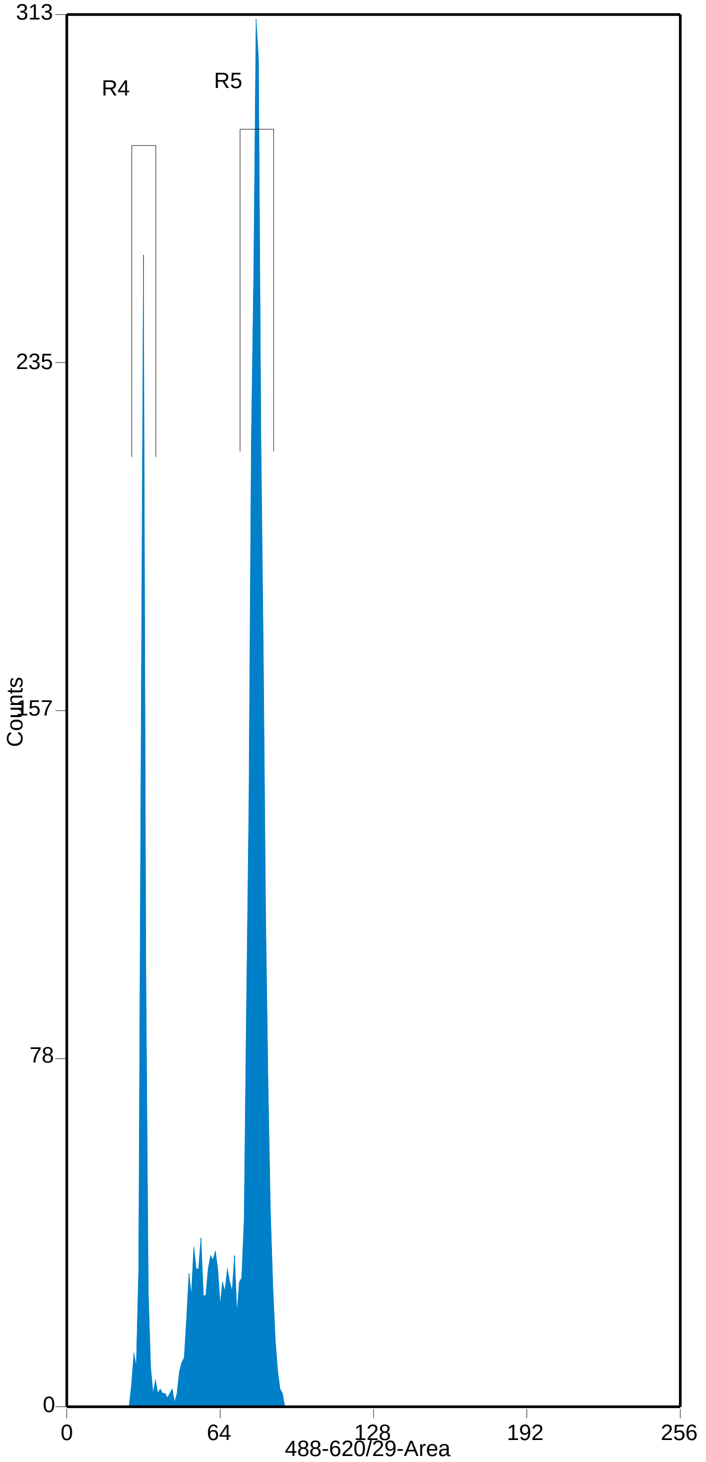

Regi	Cou	% H	Mea	CV
Tot	3	100.	66.	27.
R		17.	31.	4.
R	2	60.	79.	3.

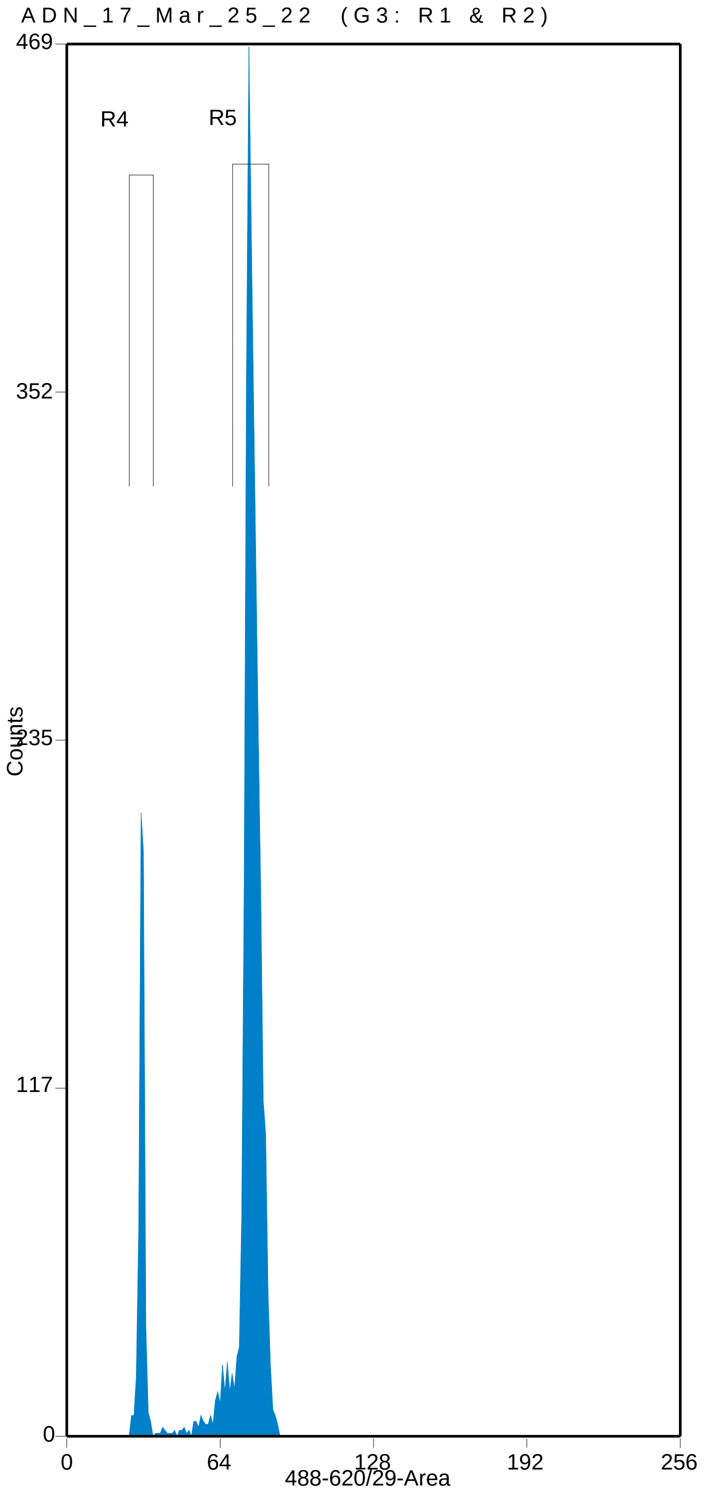

Regi	Cou	% H	Mea	CV
Tot	3	100.	69.	24.
R		15.	31.	3.
R	2	78.	77.	3.

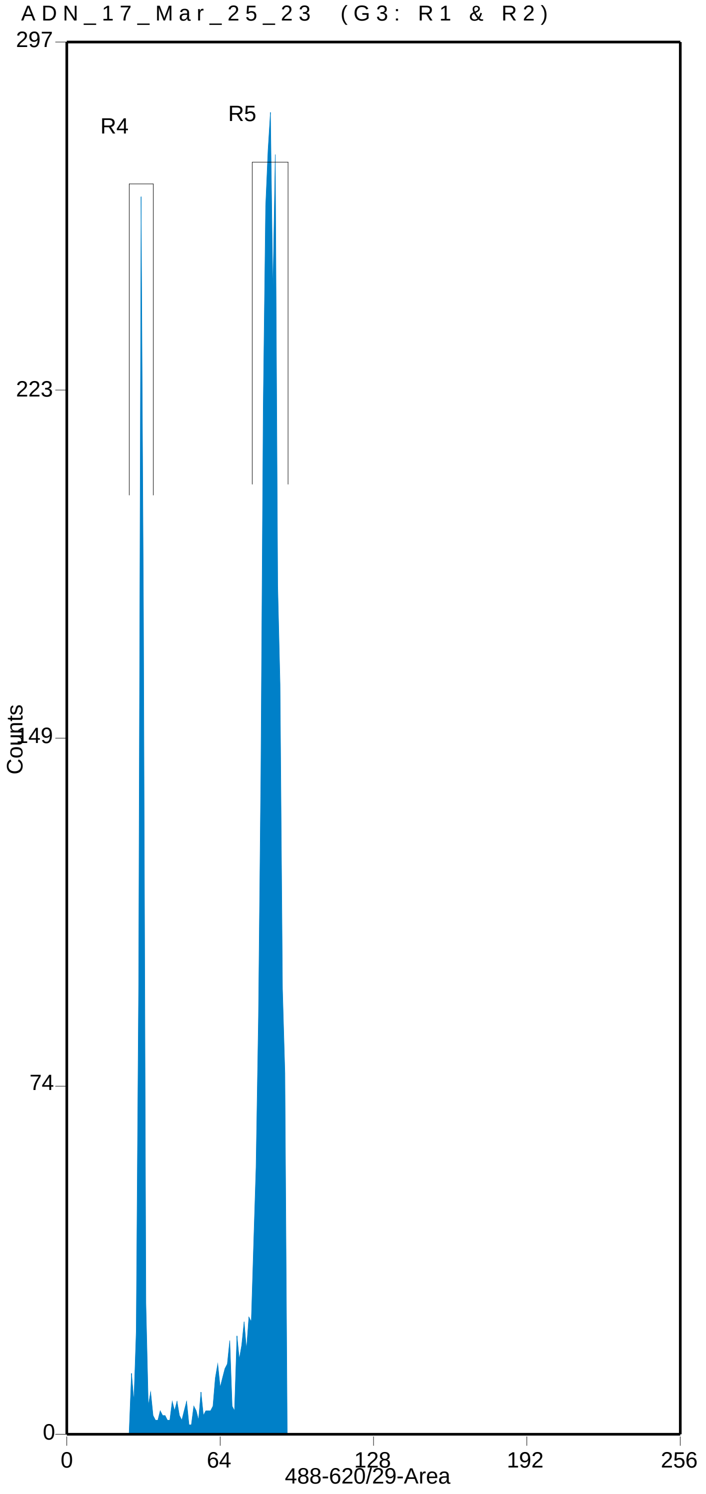

Regi	Cou	% H	Mea	CV
Tot	3	100.	72.	29.
R		18.	31.	4.
R	2	71.	84.	3.

ADN_17_Mar_25_24 (G3: R1 & R2)

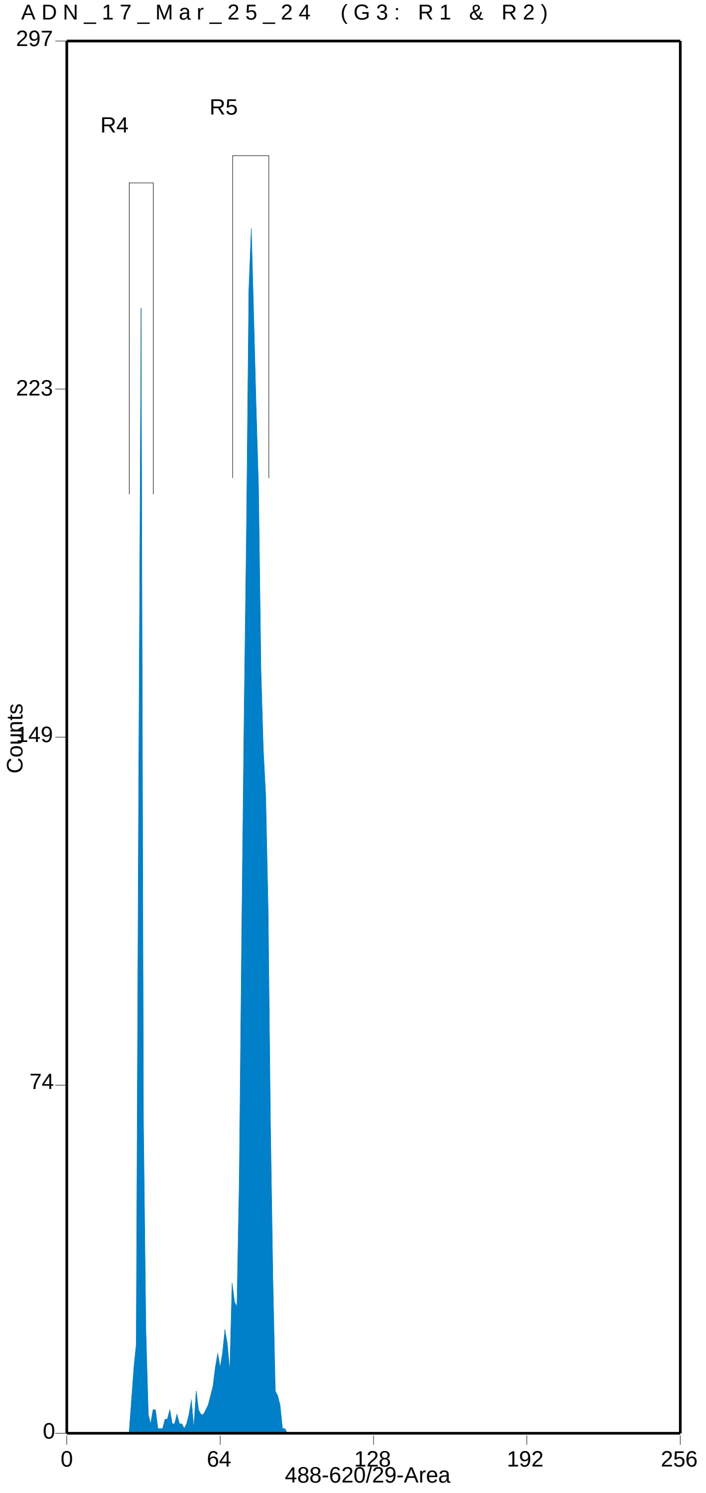

Regi	Cou	% H	Mea	CV
Tot	3	100.	69.	26.
R		16.	30.	3.
R	2	73.	77.	4.

ADN_17_Mar_25_25 (G3: R1 & R2)

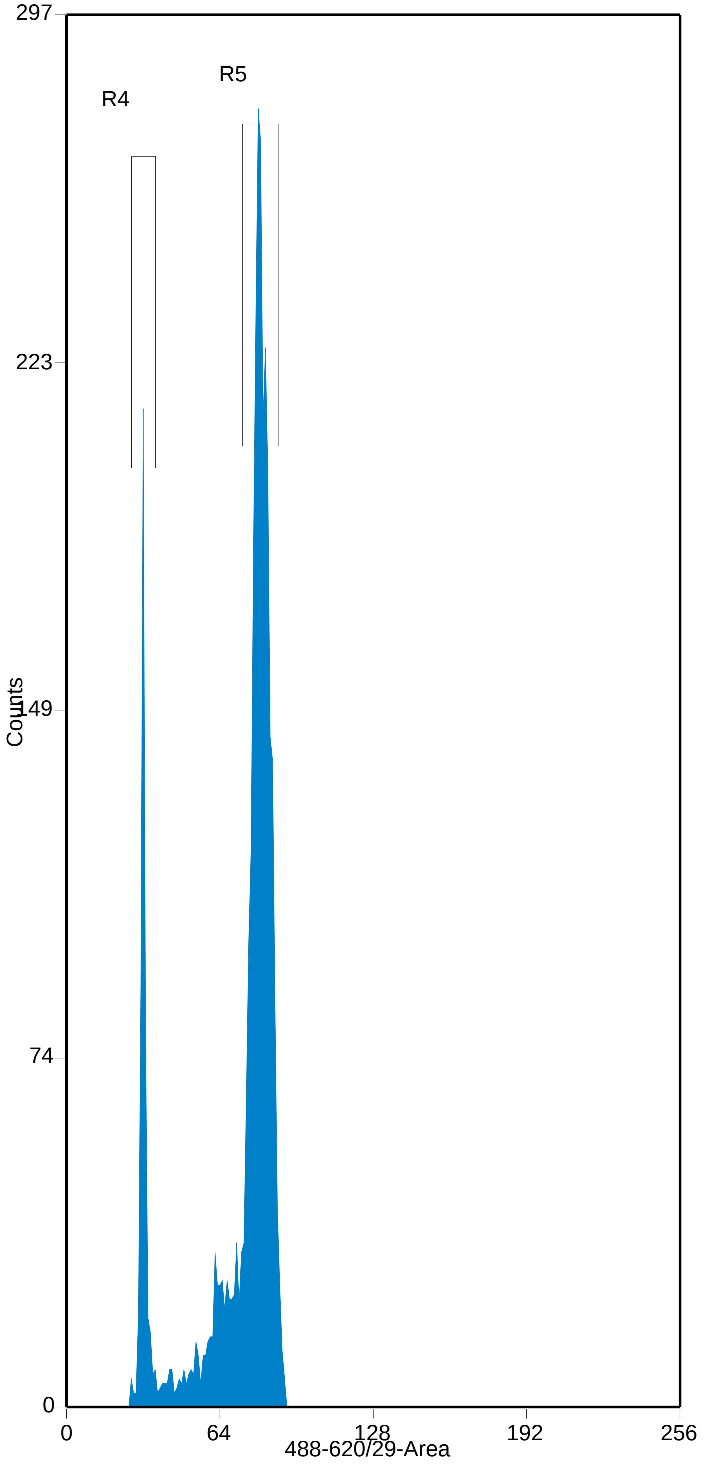

Regi	Cou	% H	Mea	CV
Tot	3	100.	71.	25.
R		13.	32.	4.
R	2	70.	81.	4.

ADN_17_Mar_25_26 (G3: R1 & R2)

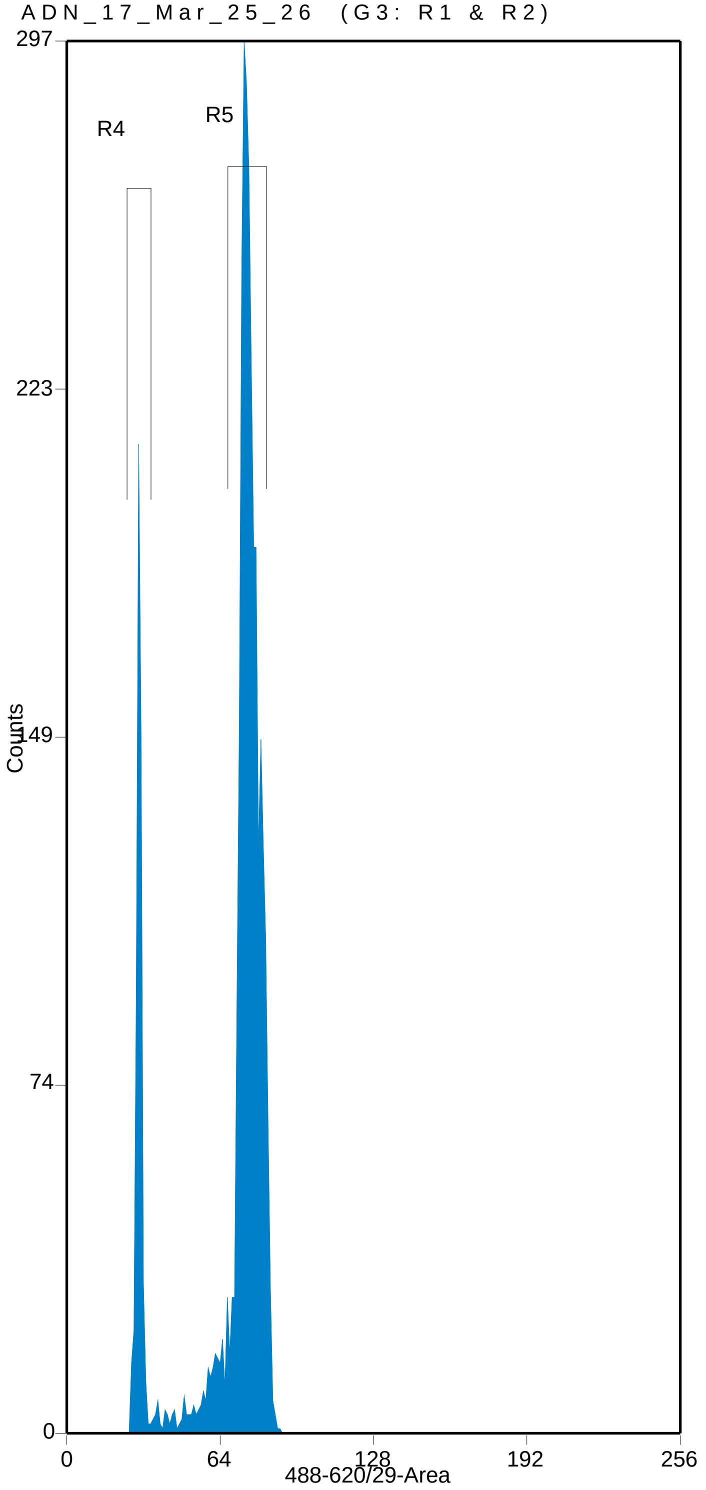

Regi	Cou	% H	Mea	CV
Tot	3	100.	67.	25.
R		15.	30.	3.
R	2	74.	76.	4.

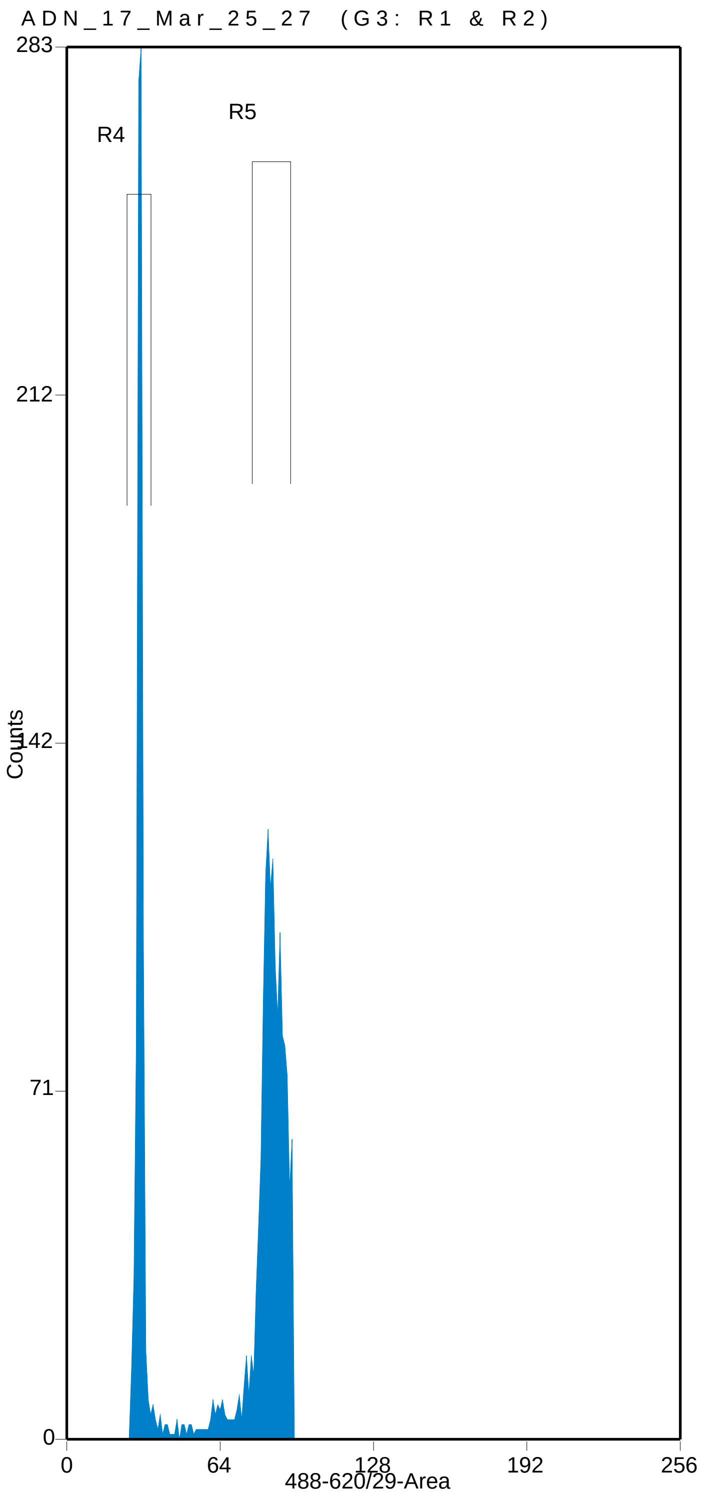

Regi	Cou	% H	Mea	CV
Tot	2	100.	64.	41.
R		36.	30.	3.
R	1	54.	86.	4.

ADN_17_Mar_25_28 (G3: R1 & R2)

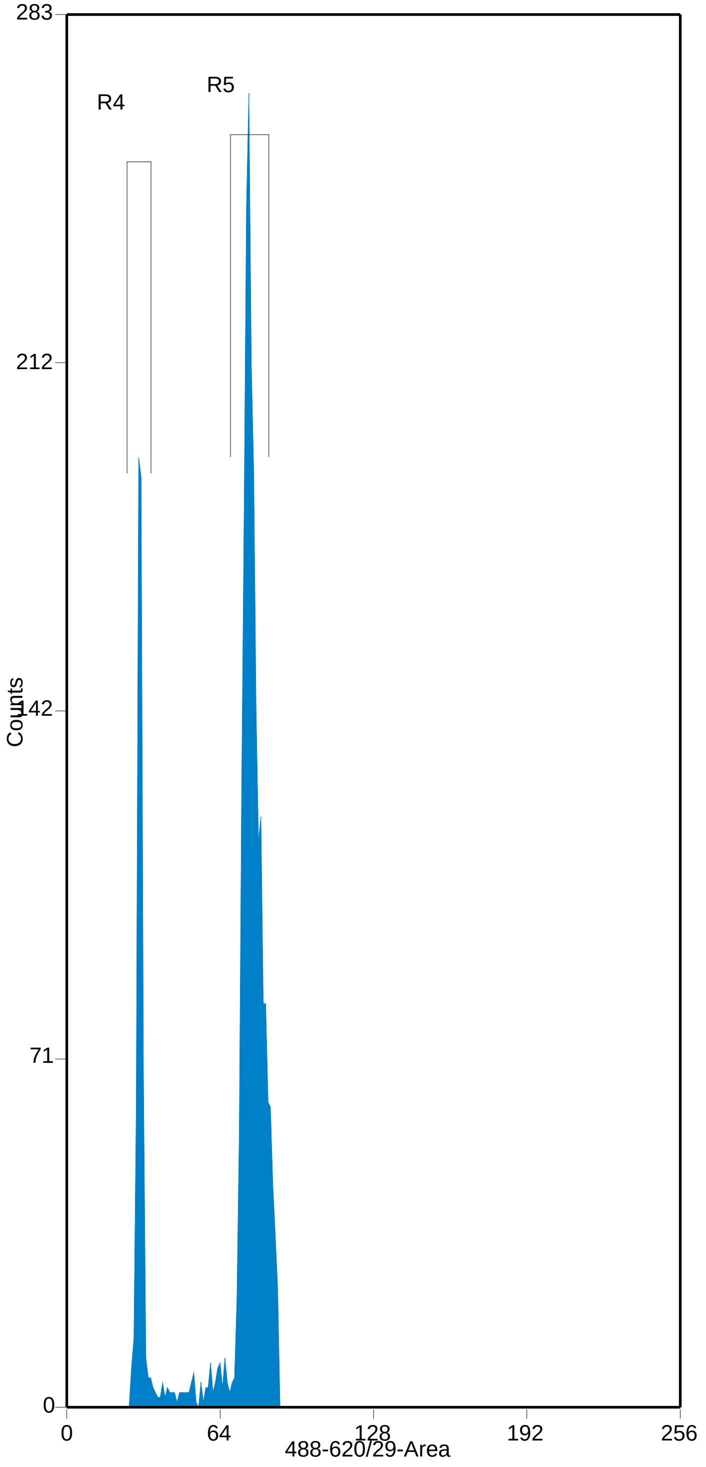

Regi	Cou	% H	Mea	CV
Tot	2	100.	67.	28.
R		20.	30.	4.
R	1	69.	77.	4.

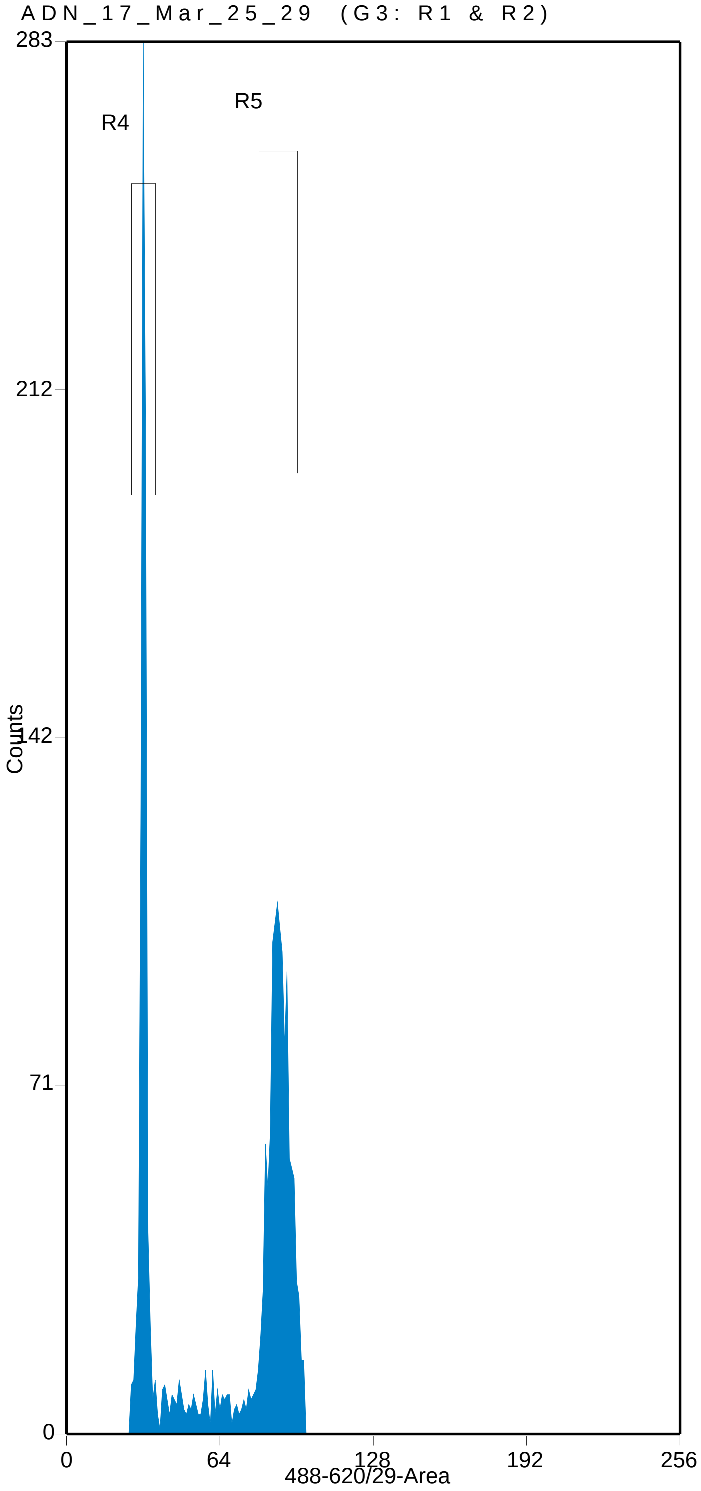

Regi	Cou	% H	Mea	CV
Tot	2[illegible]	100.[illegible]	64.[illegible]	41.[illegible]
R[illegible]	[illegible]	36.[illegible]	32.[illegible]	4.[illegible]
R[illegible]	[illegible]	49.[illegible]	88.[illegible]	4.[illegible]

IIBCELB787_3

ADN_01_Apr_25_1 (G3: R1 & R2)

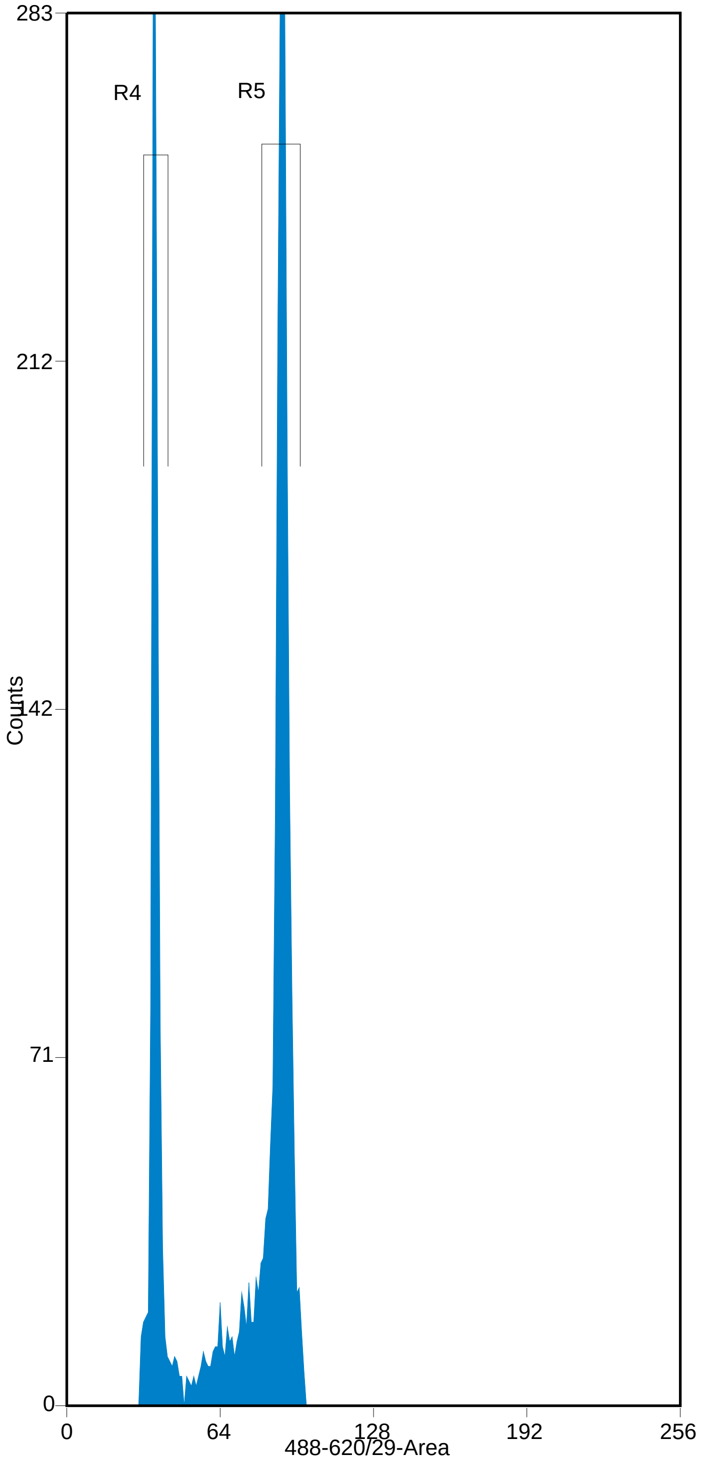

Region	Count	% Hist	Mean	CV
Total	3957	100.00	69.15	35.66
R4	1325	33.48	36.82	3.96
R5	2147	54.26	89.68	3.30

IIBCELB788_3

ADN_01_Apr_25_2 (G3: R1 & R2)

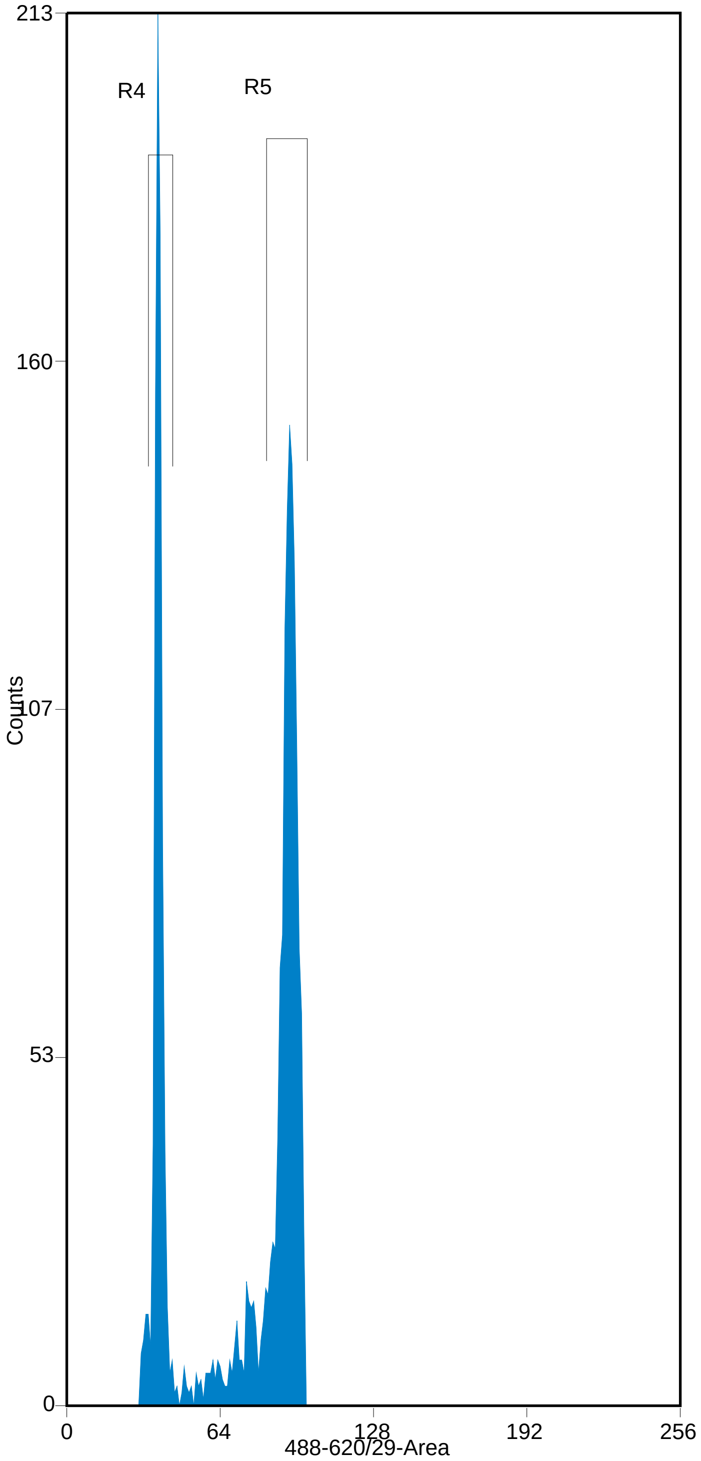

Region	Count	% Hist	Mean	CV
Total	2372	100.00	69.39	37.32
R4	876	36.93	38.32	3.97
R5	1222	51.52	92.63	3.78

IIBCELB789_3

ADN_01_Apr_25_3 (G3: R1 & R2)

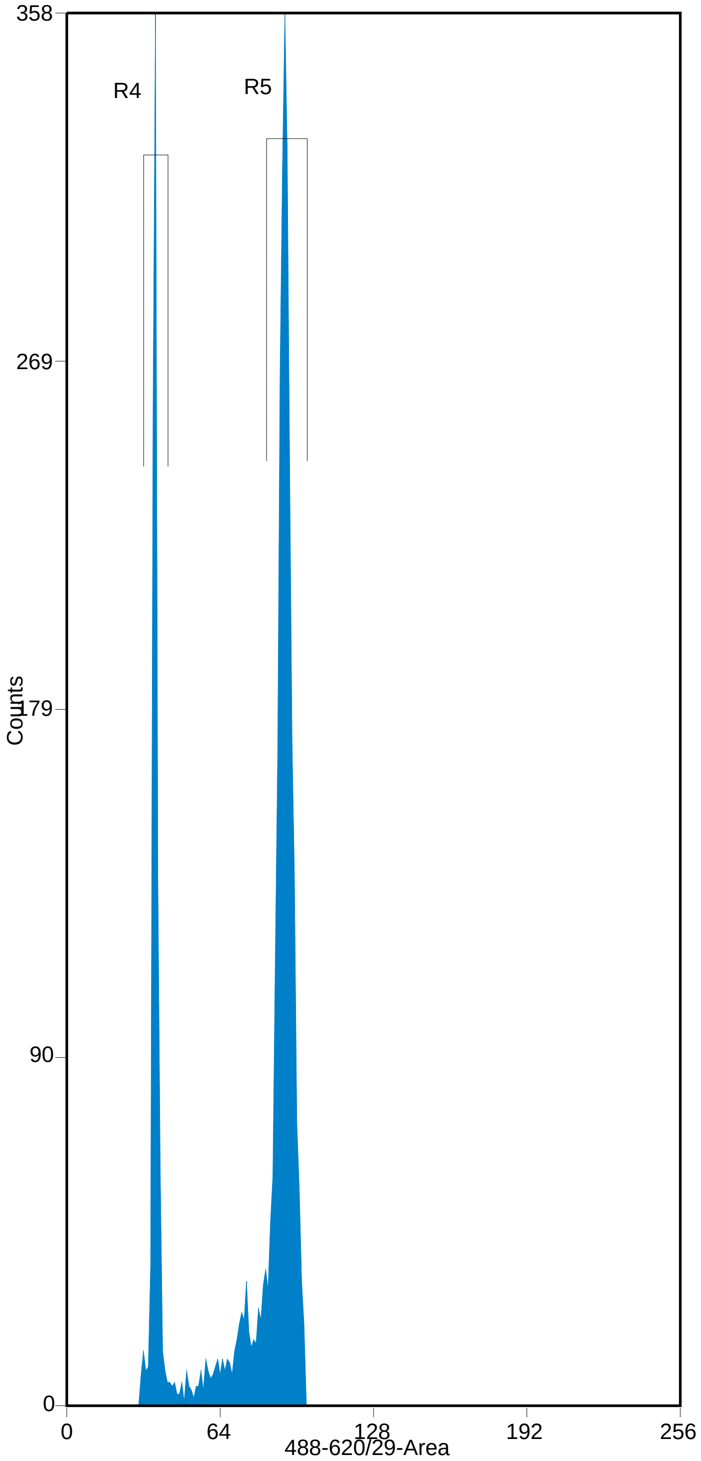

Region	Count	% Hist	Mean	CV
Total	3883	100.00	75.08	31.09
R4	946	24.36	36.89	3.71
R5	2463	63.43	91.03	3.38

IIBCELB790_3

ADN_01_Apr_25_4 (G3: R1 & R2)

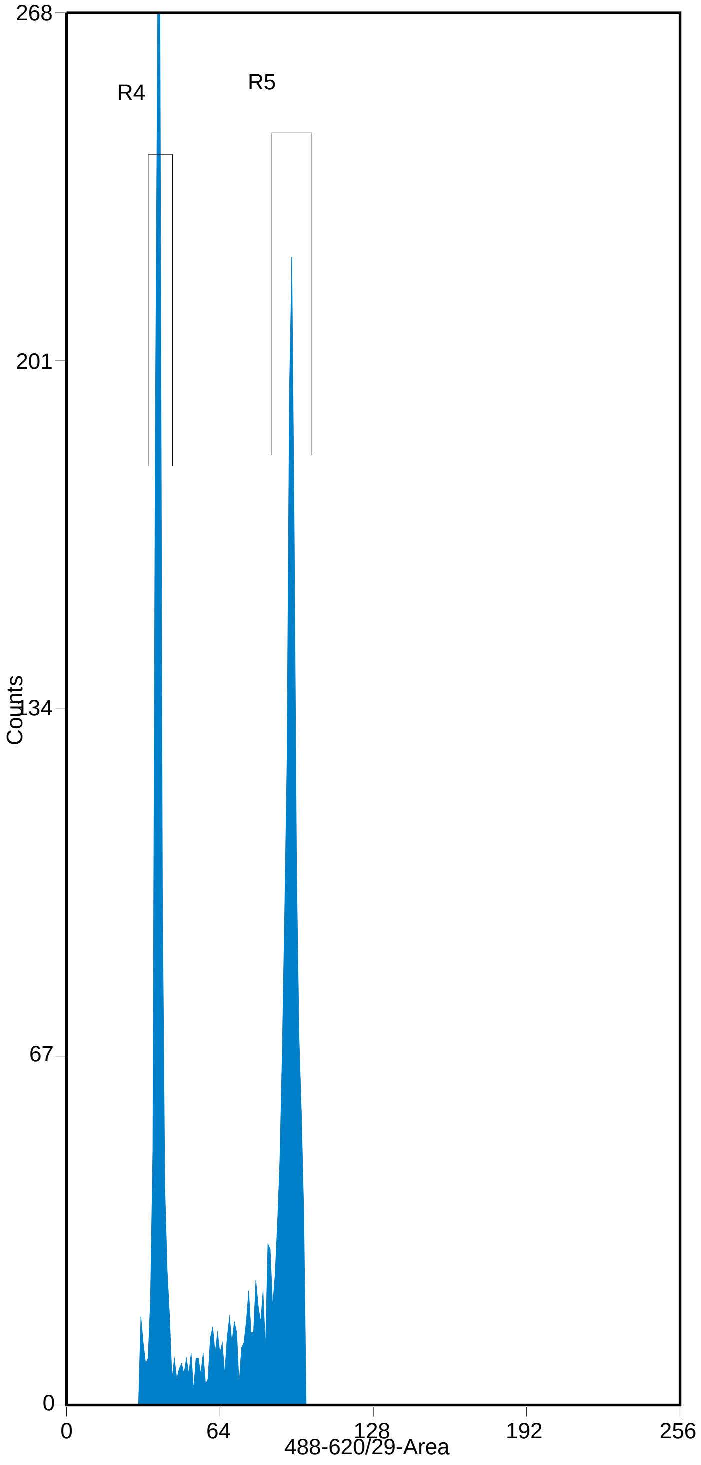

Region	Count	% Hist	Mean	CV
Total	3024	100.00	66.55	38.70
R4	1217	40.24	38.42	3.83
R5	1296	42.86	93.19	3.28

IIBCELB791_3

ADN_01_Apr_25_5 (G3: R1 & R2)

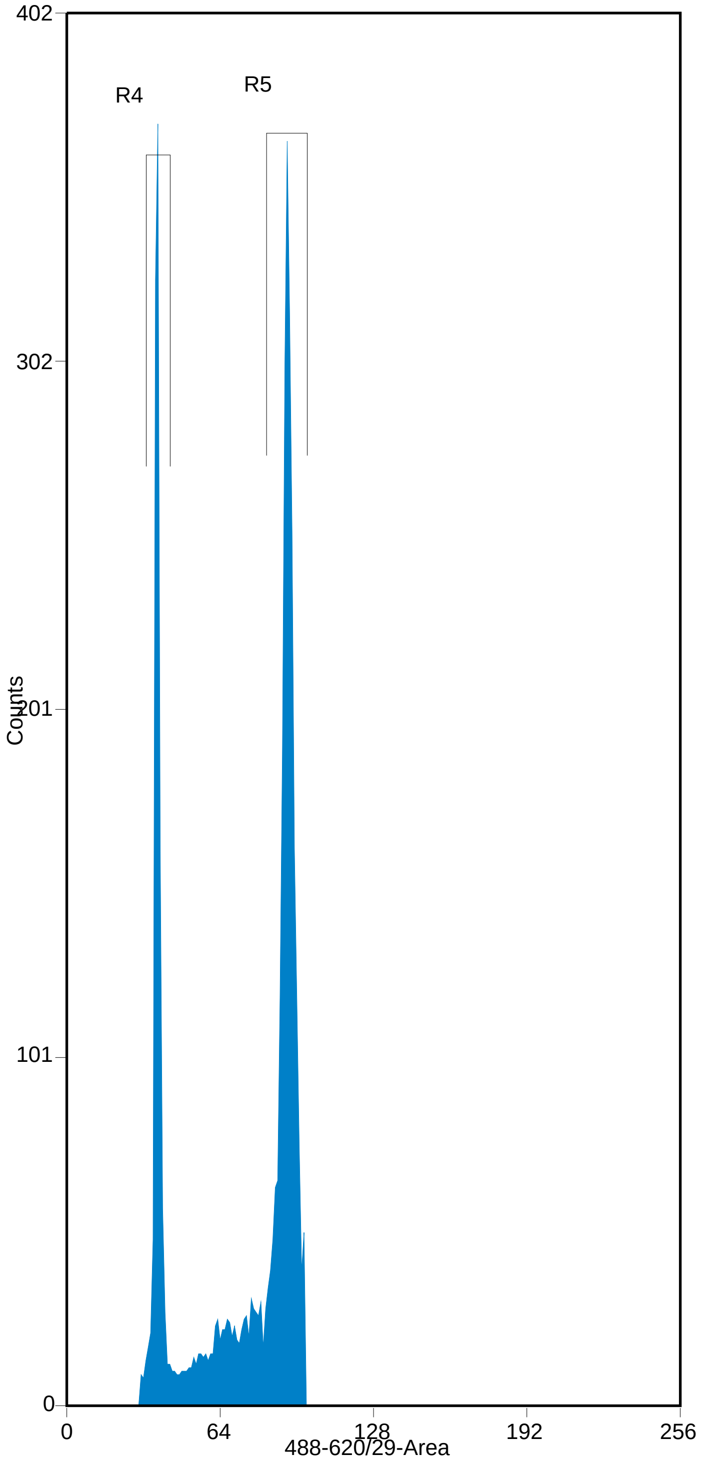

Region	Count	% Hist	Mean	CV
Total	4051	100.00	73.08	32.56
R4	1063	26.24	37.86	3.97
R5	2261	55.81	92.02	3.48

IIBCELB798_3

ADN_01_Apr_25_6 (G3: R1 & R2)

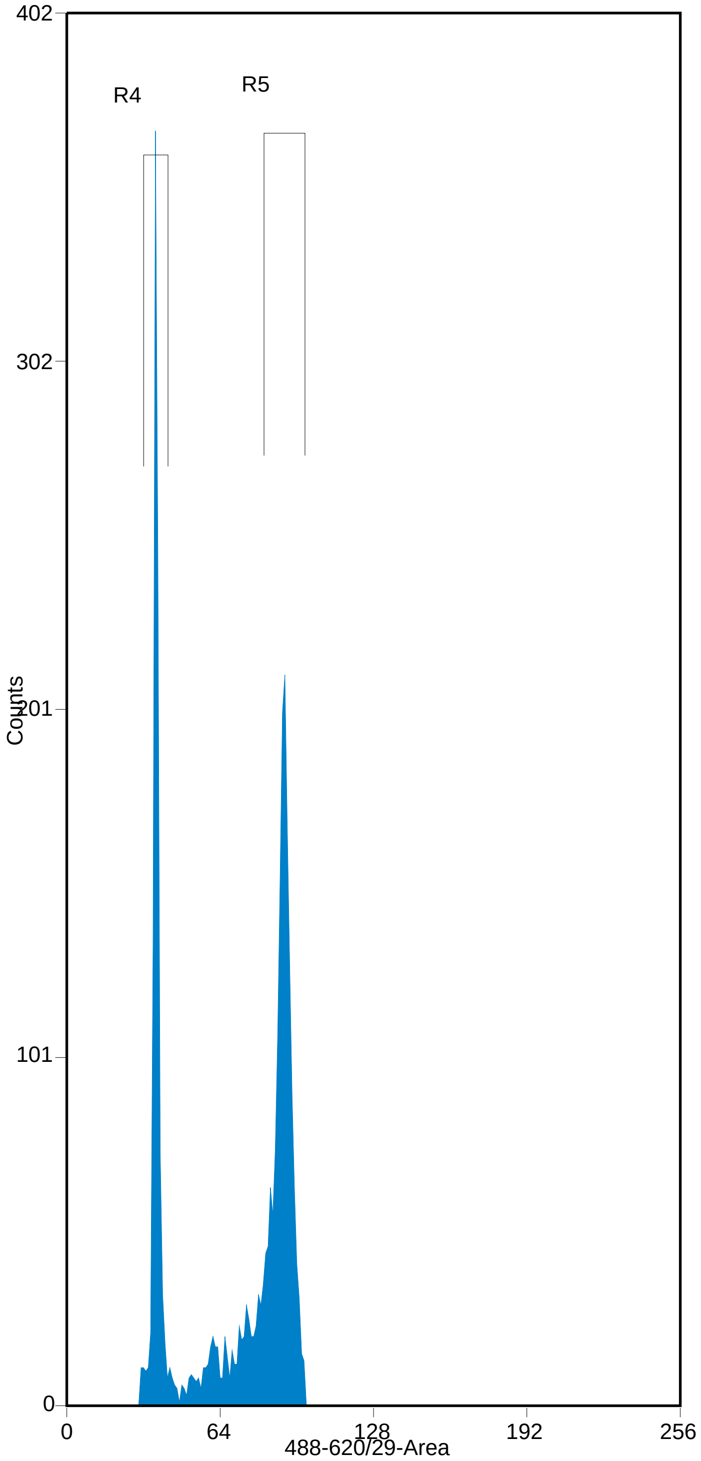

Region	Count	% Hist	Mean	CV
Total	3009	100.00	69.58	34.38
R4	930	30.91	37.32	3.90
R5	1535	51.01	90.21	3.98

BECKMAN COULTER

IIBCELB799_3

ADN_01_Apr_25_7 (G3: R1 & R2)

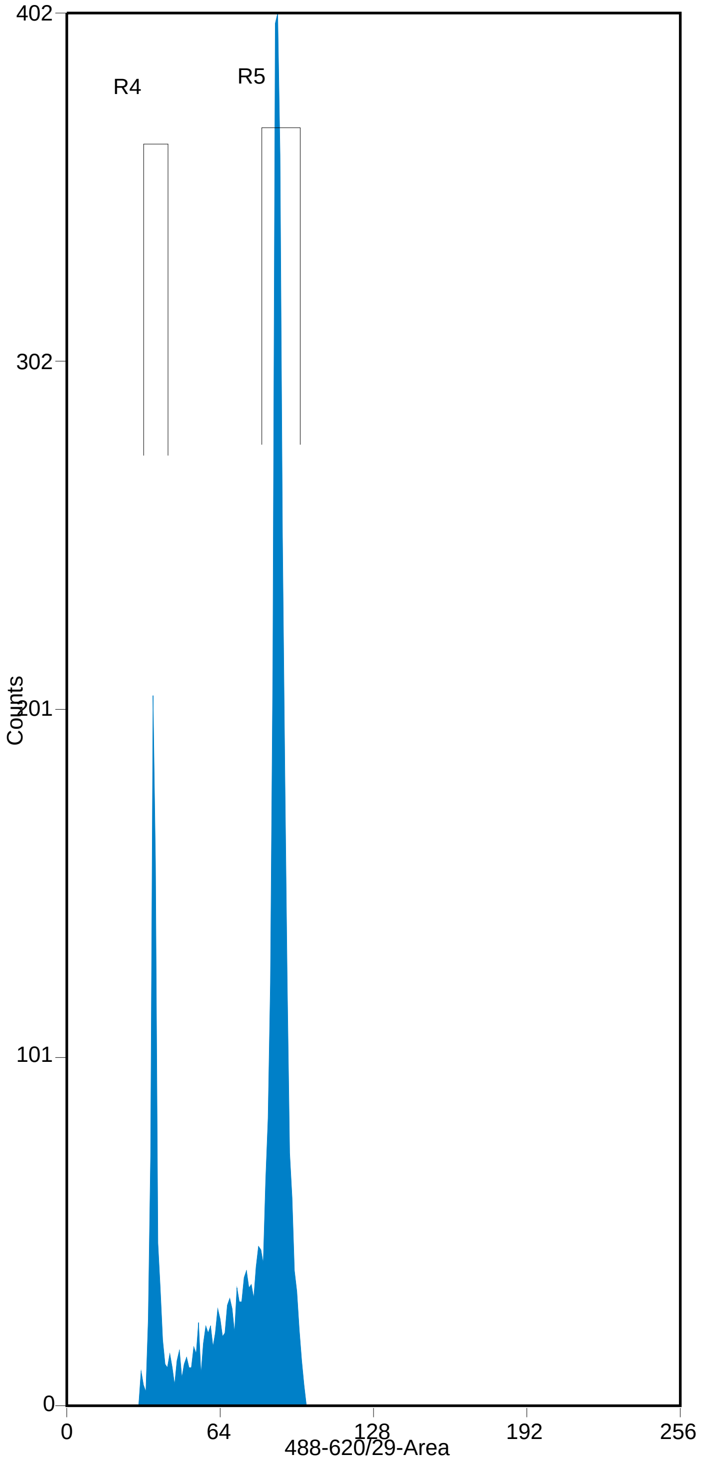

Region	Count	% Hist	Mean	CV
Total	4025	100.00	75.83	25.81
R4	592	14.71	36.67	4.61
R5	2540	63.11	88.37	3.43

IIBCELB793_3

ADN_01_Apr_25_8 (G3: R1 & R2)

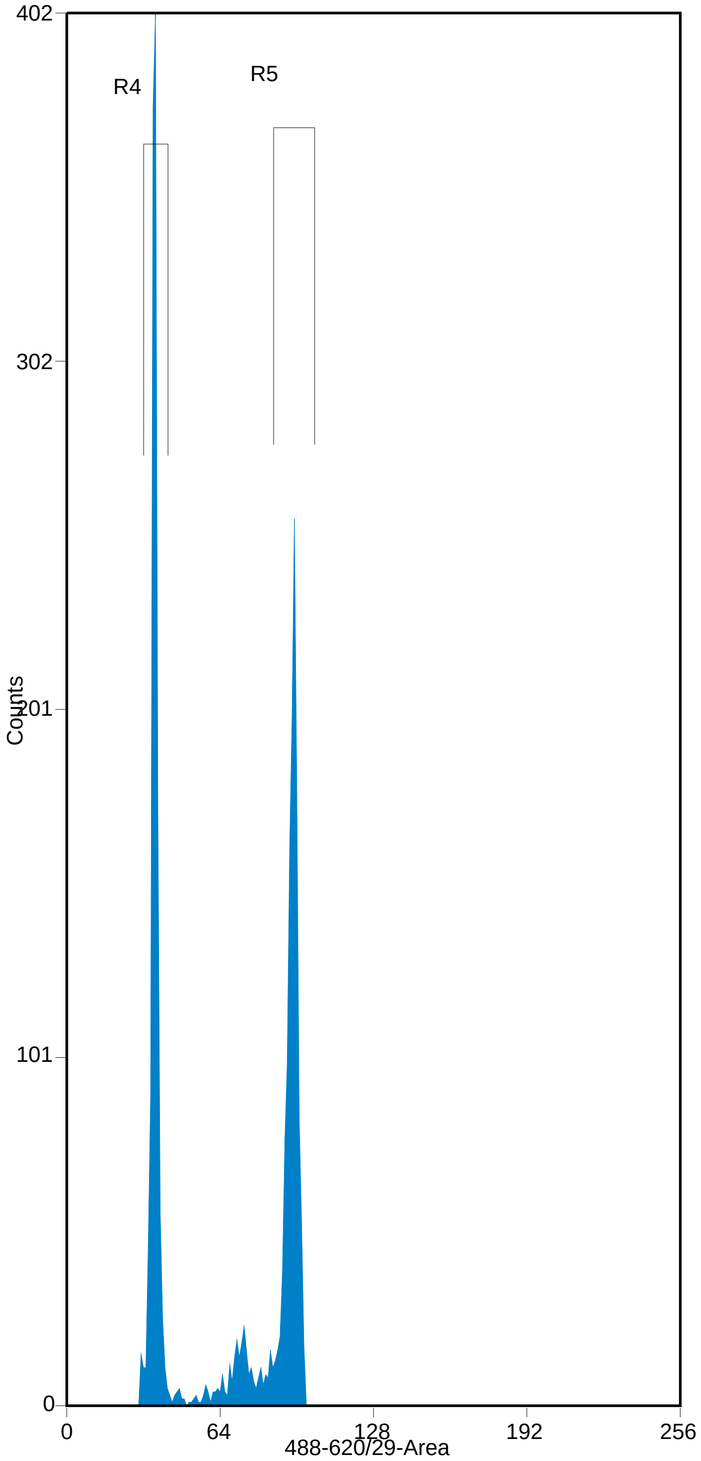

Region	Count	% Hist	Mean	CV
Total	2764	100.00	65.56	41.90
R4	1239	44.83	36.70	3.81
R5	1221	44.18	94.03	2.59

IIBCELB794_3

ADN_01_Apr_25_9 (G3: R1 & R2)

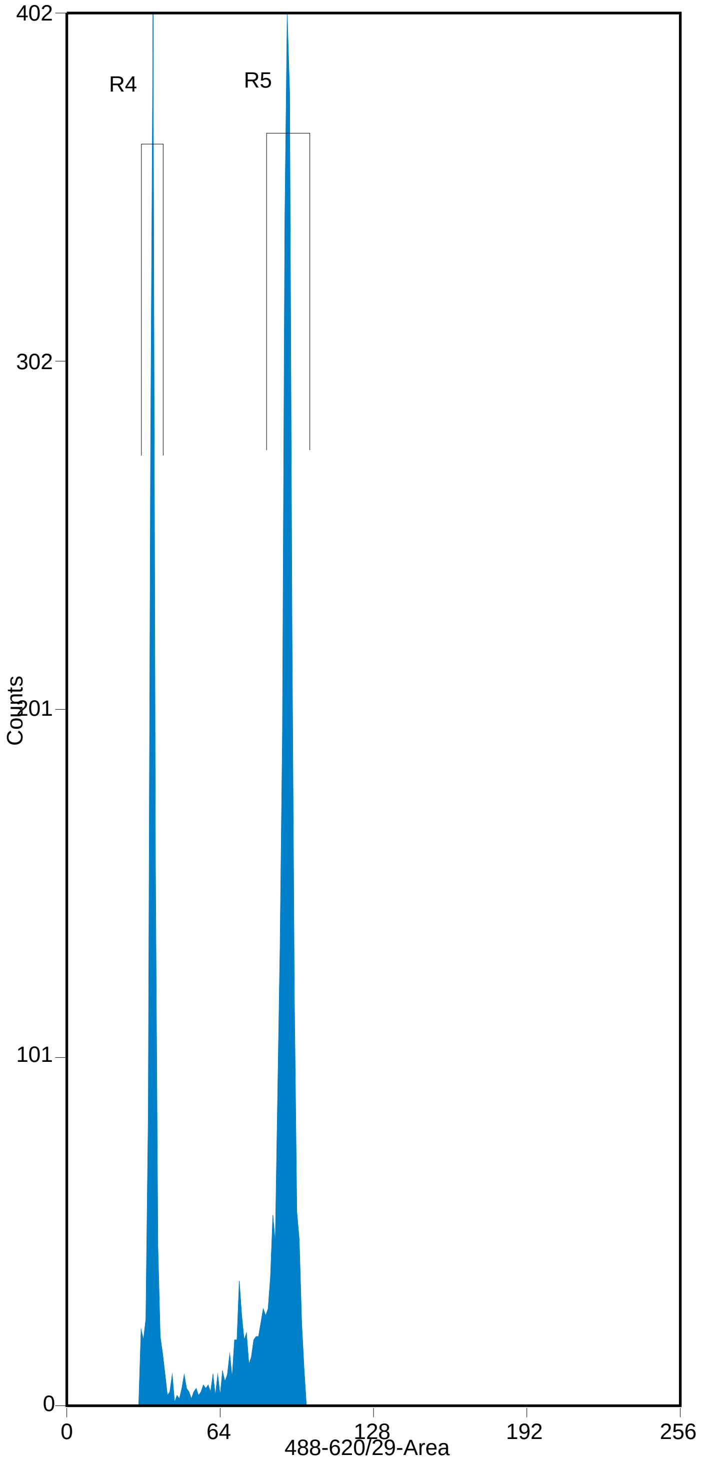

Region	Count	% Hist	Mean	CV
Total	3821	100.00	73.09	34.20
R4	1085	28.40	35.69	4.12
R5	2293	60.01	91.60	3.06

IIBCELB795_3

ADN_01_Apr_25_10 (G3: R1 & R2)

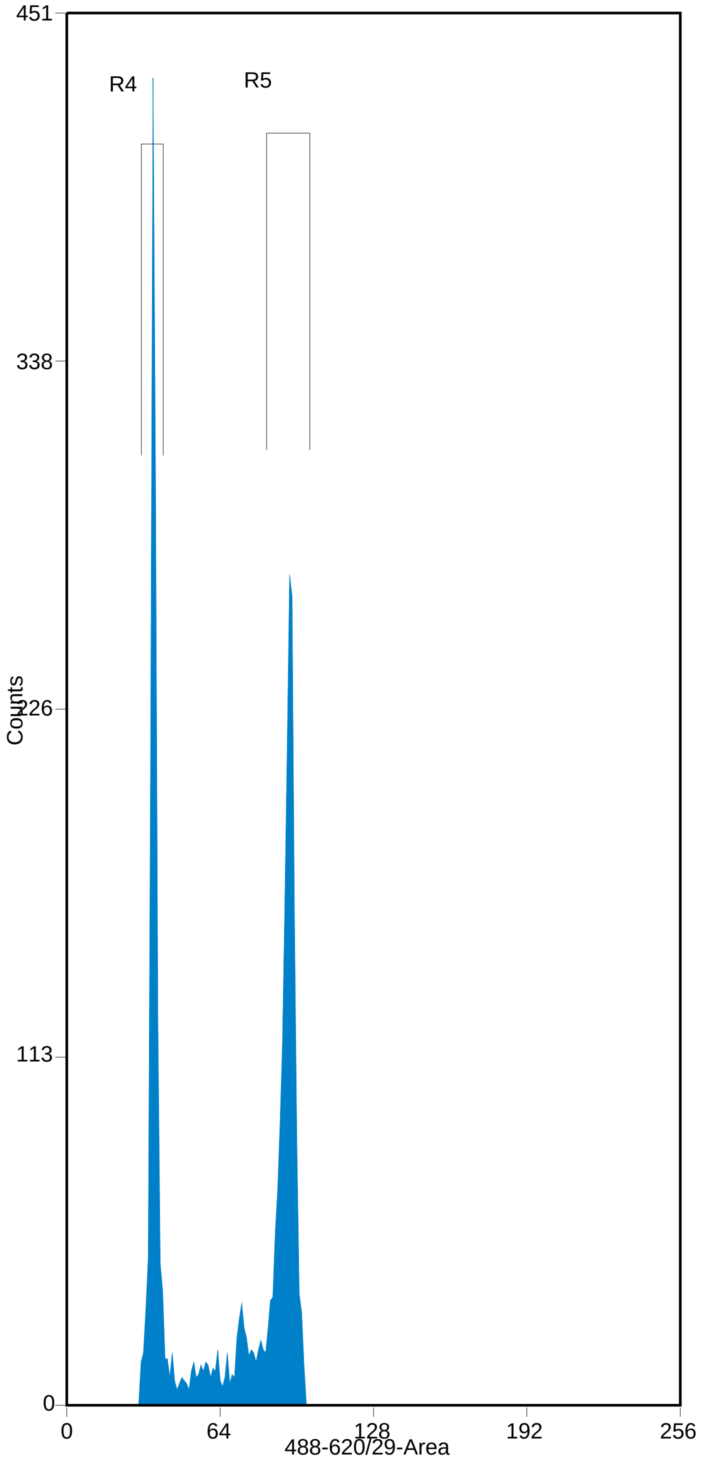

Region	Count	% Hist	Mean	CV
Total	3507	100.00	67.50	38.37
R4	1262	35.99	36.24	4.28
R5	1677	47.82	92.02	3.42

IIBCELB796_3

ADN_01_Apr_25_11 (G3: R1 & R2)

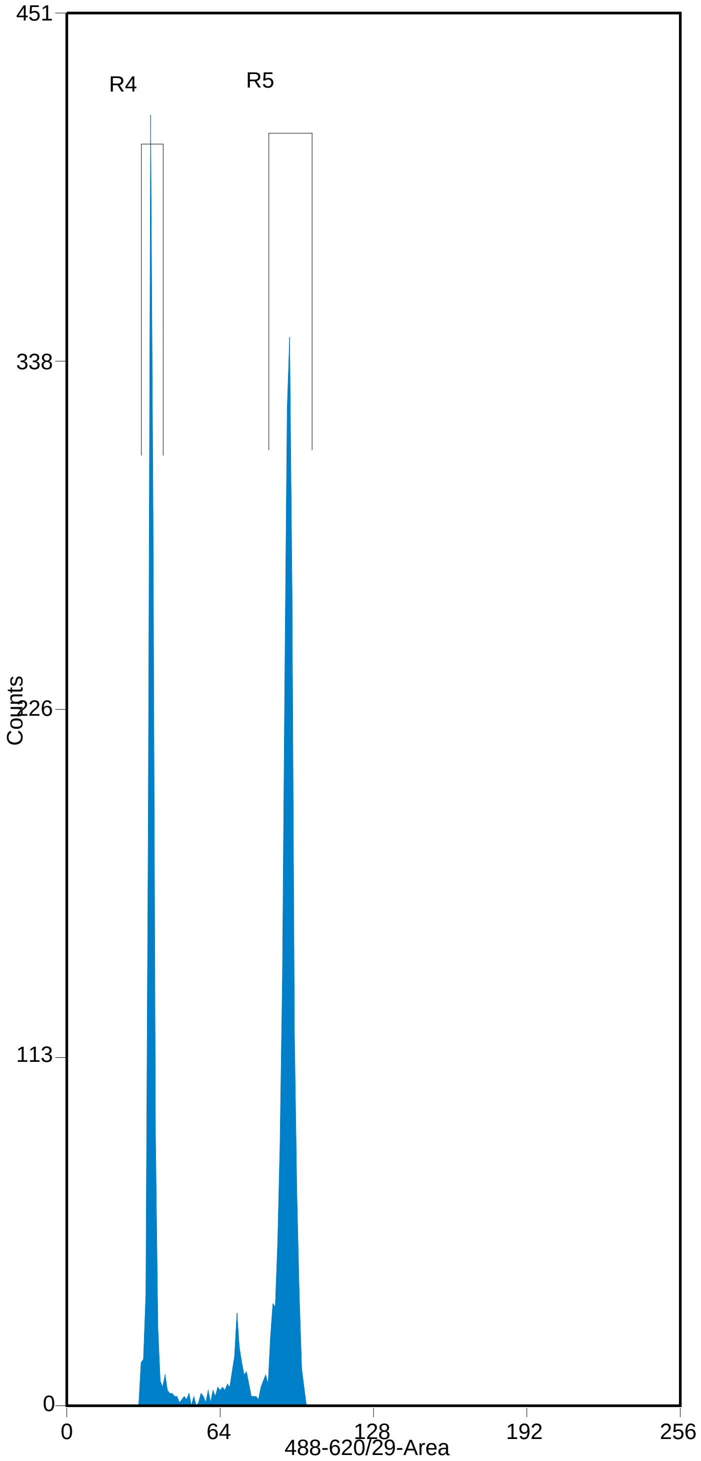

Region	Count	% Hist	Mean	CV
Total	3127	100.00	70.27	38.08
R4	1087	34.76	35.22	3.64
R5	1785	57.08	92.14	2.71

IIBCELB797_3

ADN_01_Apr_25_12 (G3: R1 & R2)

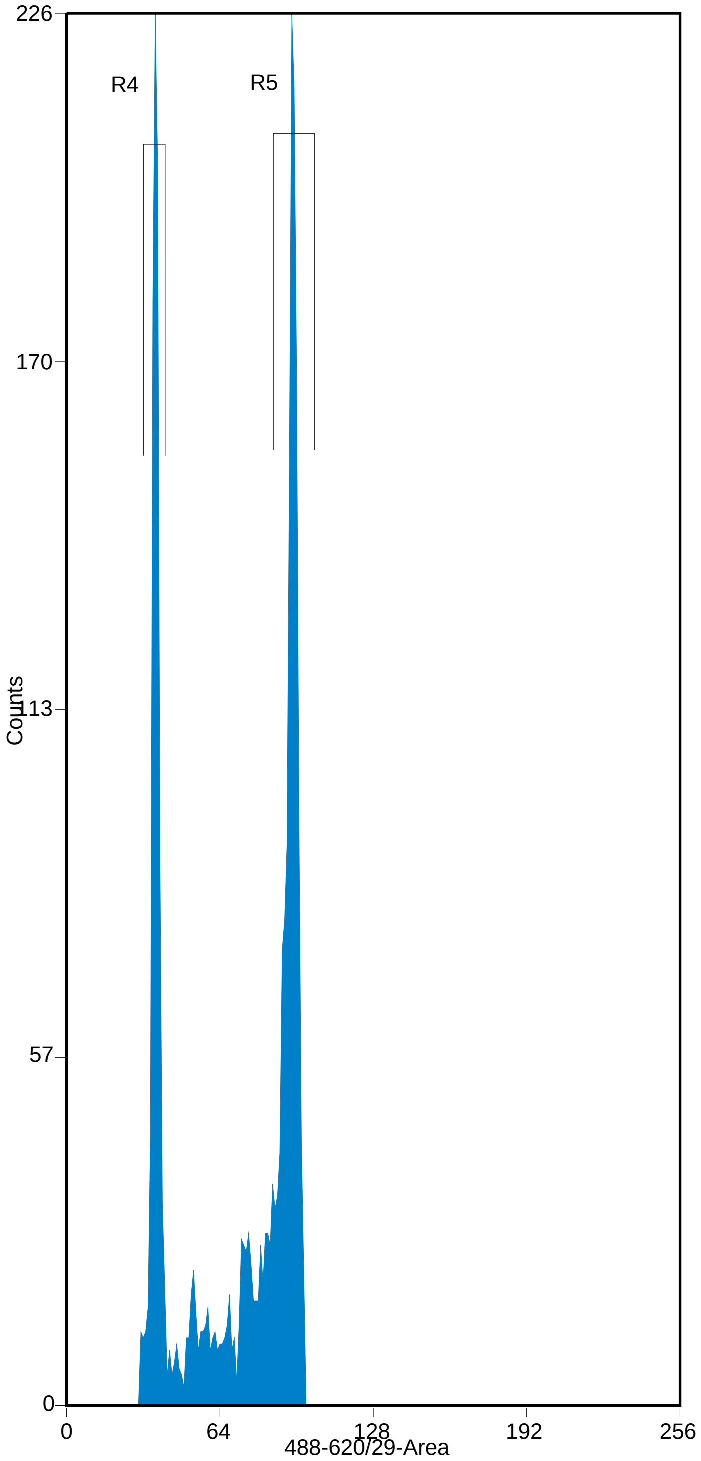

Region	Count	% Hist	Mean	CV
Total	2921	100.00	69.21	36.74
R4	959	32.83	37.14	3.99
R5	1313	44.95	93.49	3.12

BECKMAN COULTER

IIBCELB7801_3

ADN_01_Apr_25_13 (G3: R1 & R2)

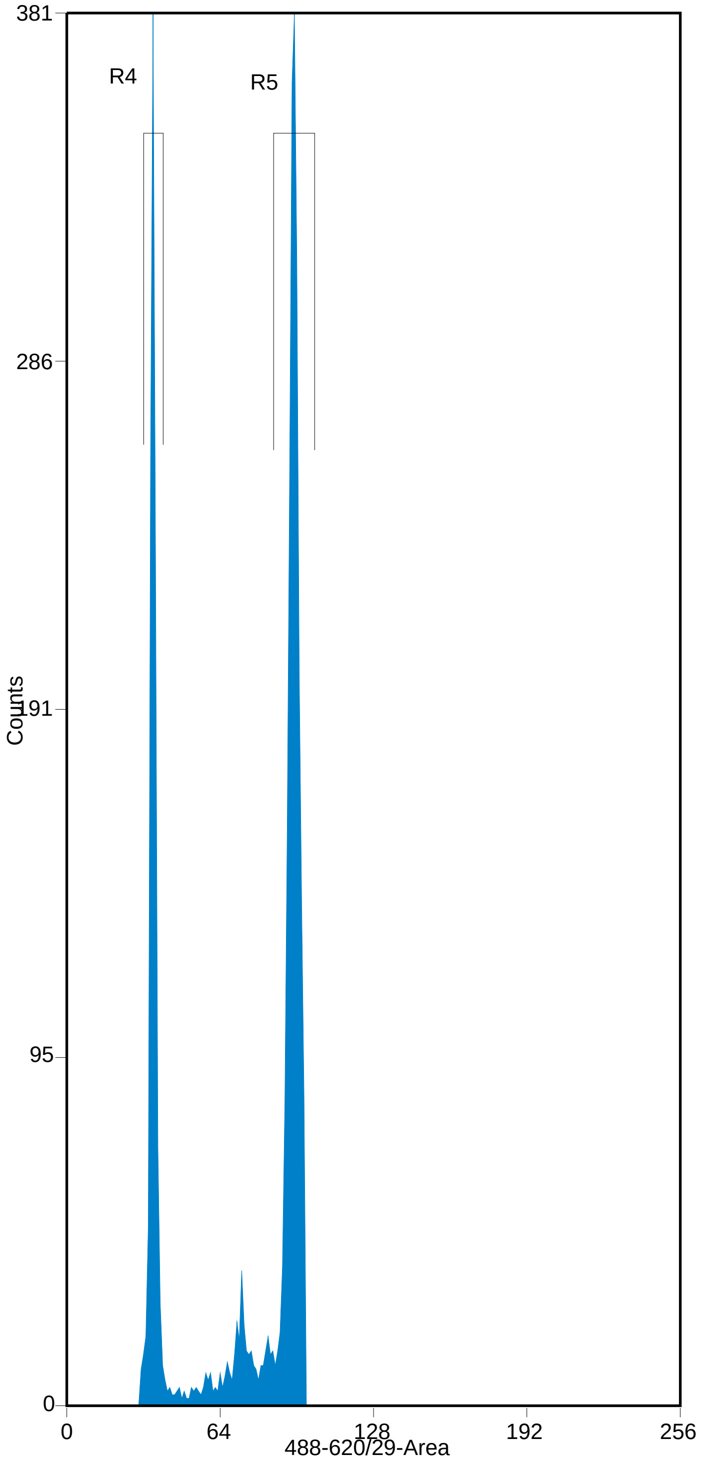

Region	Count	% Hist	Mean	CV
Total	3684	100.00	73.63	36.29
R4	1131	30.70	36.03	3.50
R5	2132	57.87	94.60	2.49

BECKMAN COULTER

IIBCELB802_3

ADN_01_Apr_25_14 (G3: R1 & R2)

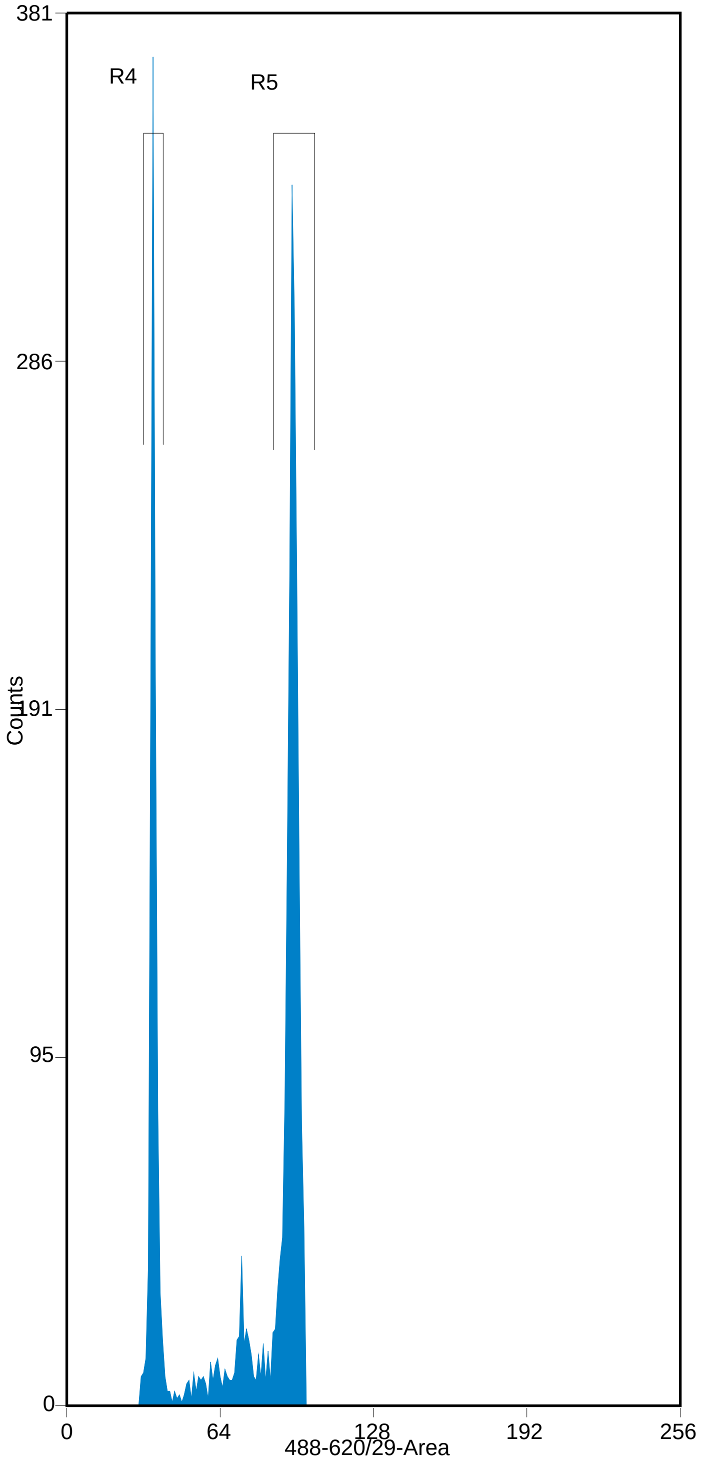

Region	Count	% Hist	Mean	CV
Total	3130	100.00	72.92	35.96
R4	949	30.32	36.19	3.65
R5	1756	56.10	94.05	2.75

BECKMAN COULTER

spiderling of IIBCELB795_1

ADN_01_Apr_25_15 (G3: R1 & R2)

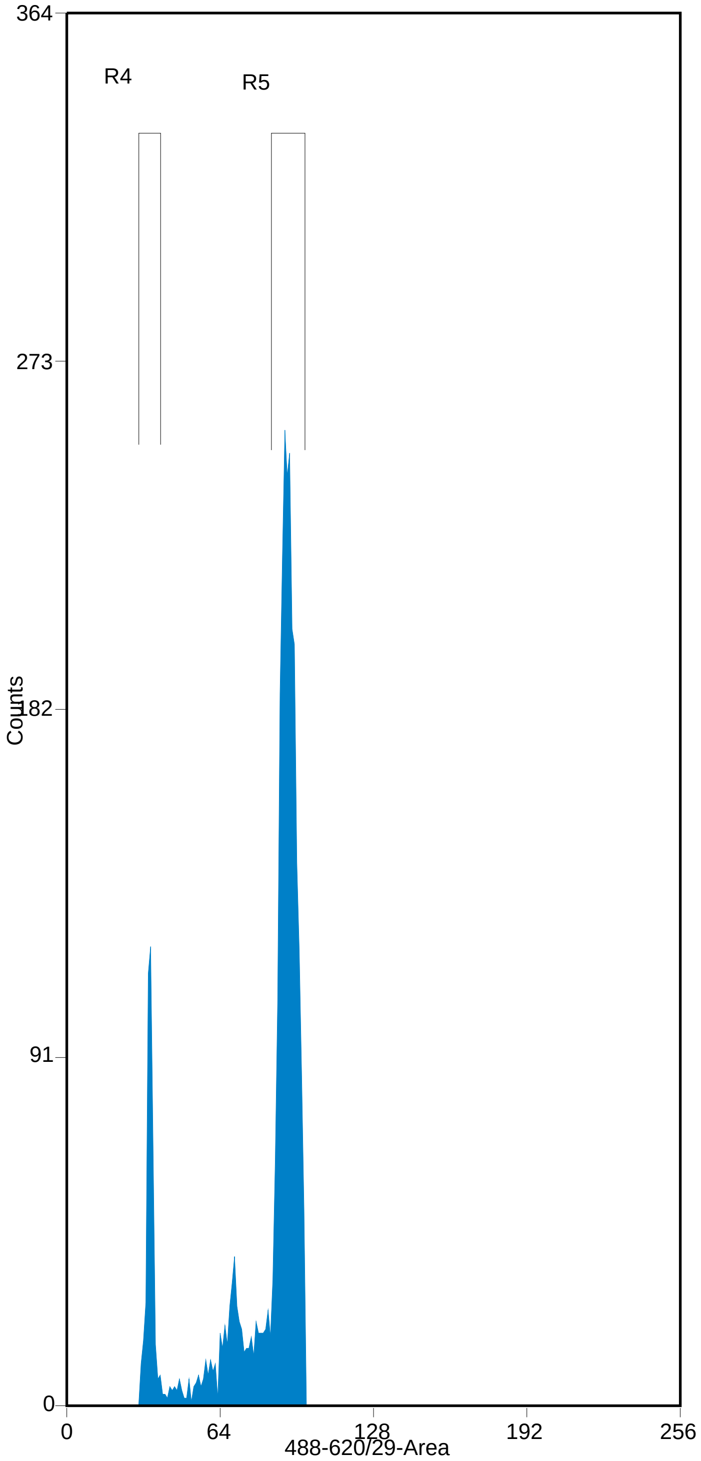

Region	Count	% Hist	Mean	CV
Total	3132	100.00	81.05	24.94
R4	387	12.36	34.72	4.25
R5	2176	69.48	92.44	3.40

BECKMAN COULTER

spiderling of IIBCELB795_2

ADN_01_Apr_25_16 (G3: R1 & R2)

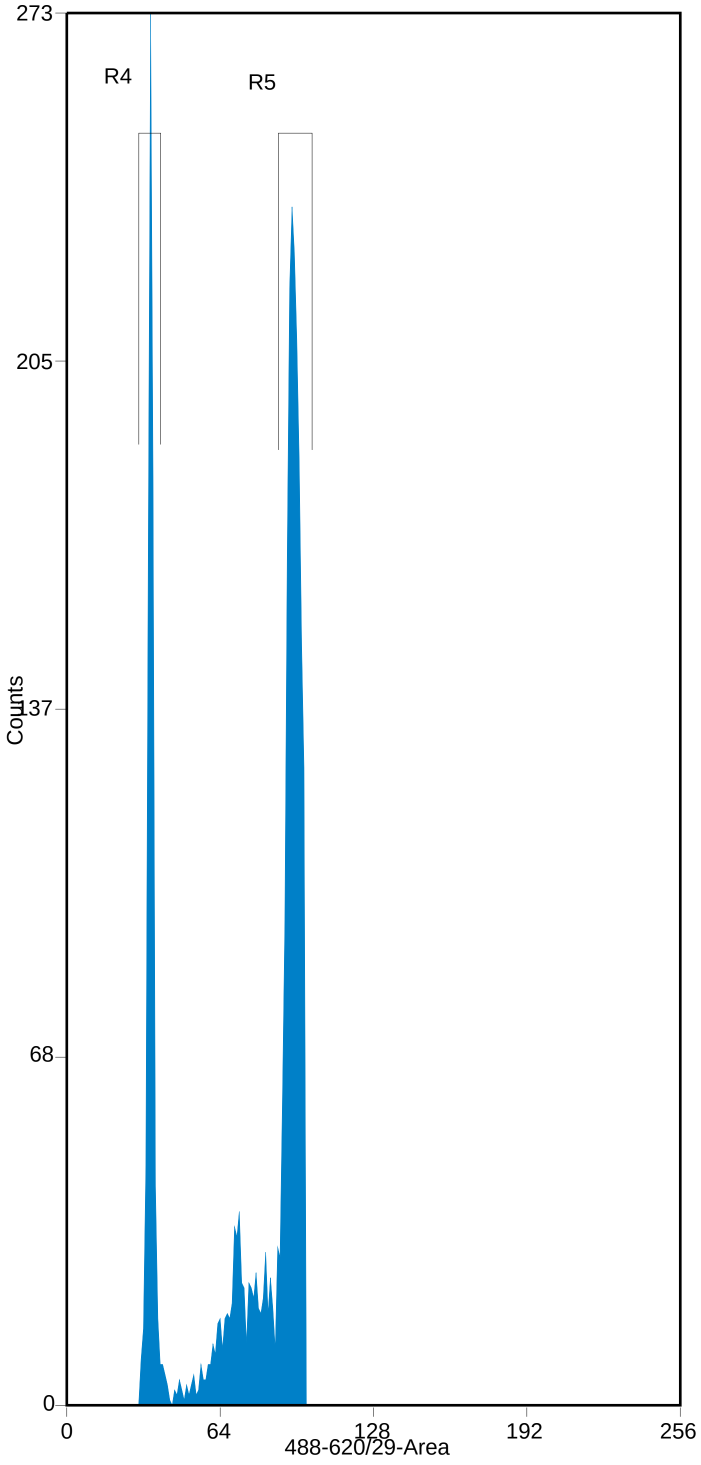

Region	Count	% Hist	Mean	CV
Total	3176	100.00	74.84	33.86
R4	801	25.22	35.01	3.60
R5	1729	54.44	94.59	2.81

BECKMAN COULTER

ADN_01_Apr_25_17 (G3: R1 & R2)

spiderling of IIBCELB795_3

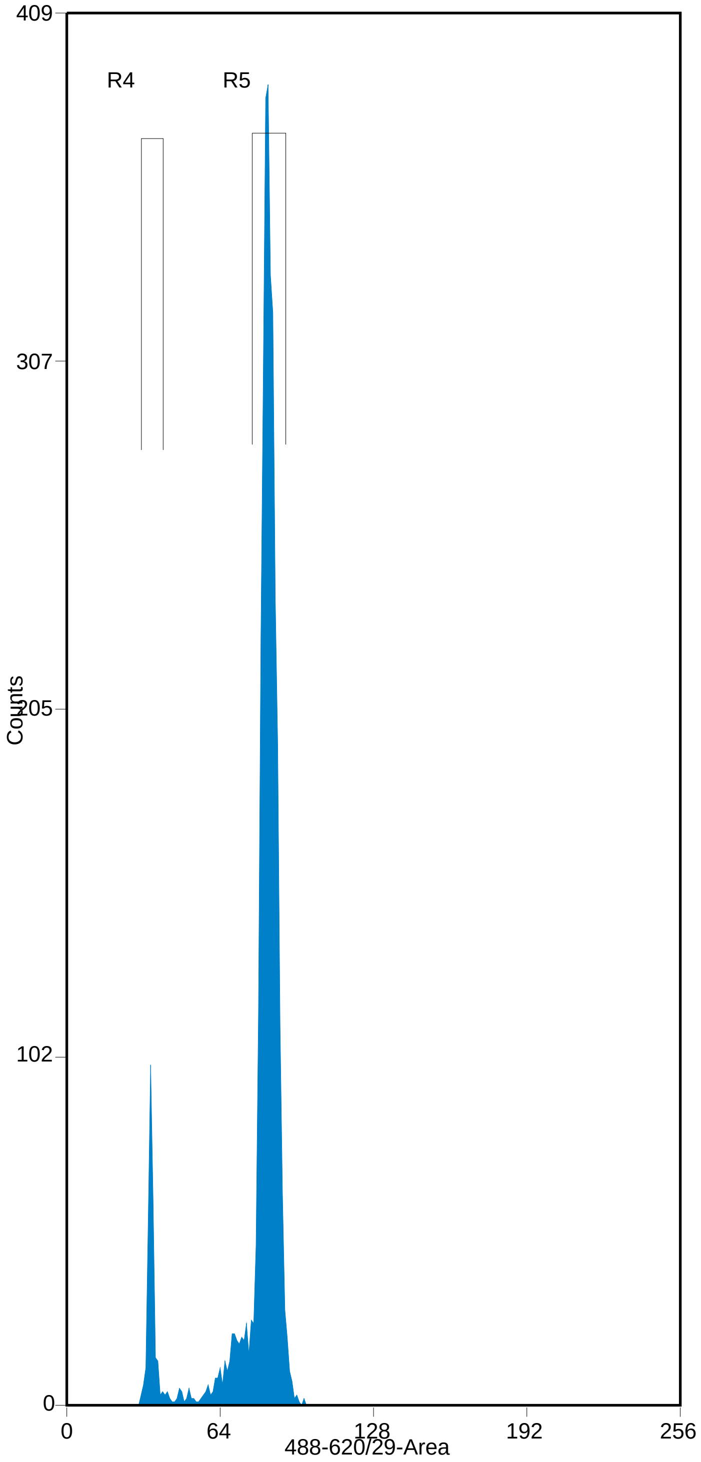

Region	Count	% Hist	Mean	CV
Total	3394	100.00	79.04	18.11
R4	270	7.96	35.20	4.14
R5	2795	82.35	84.33	3.30

spiderling of IIBCELB795_4

ADN_01_Apr_25_18 (G3: R1 & R2)

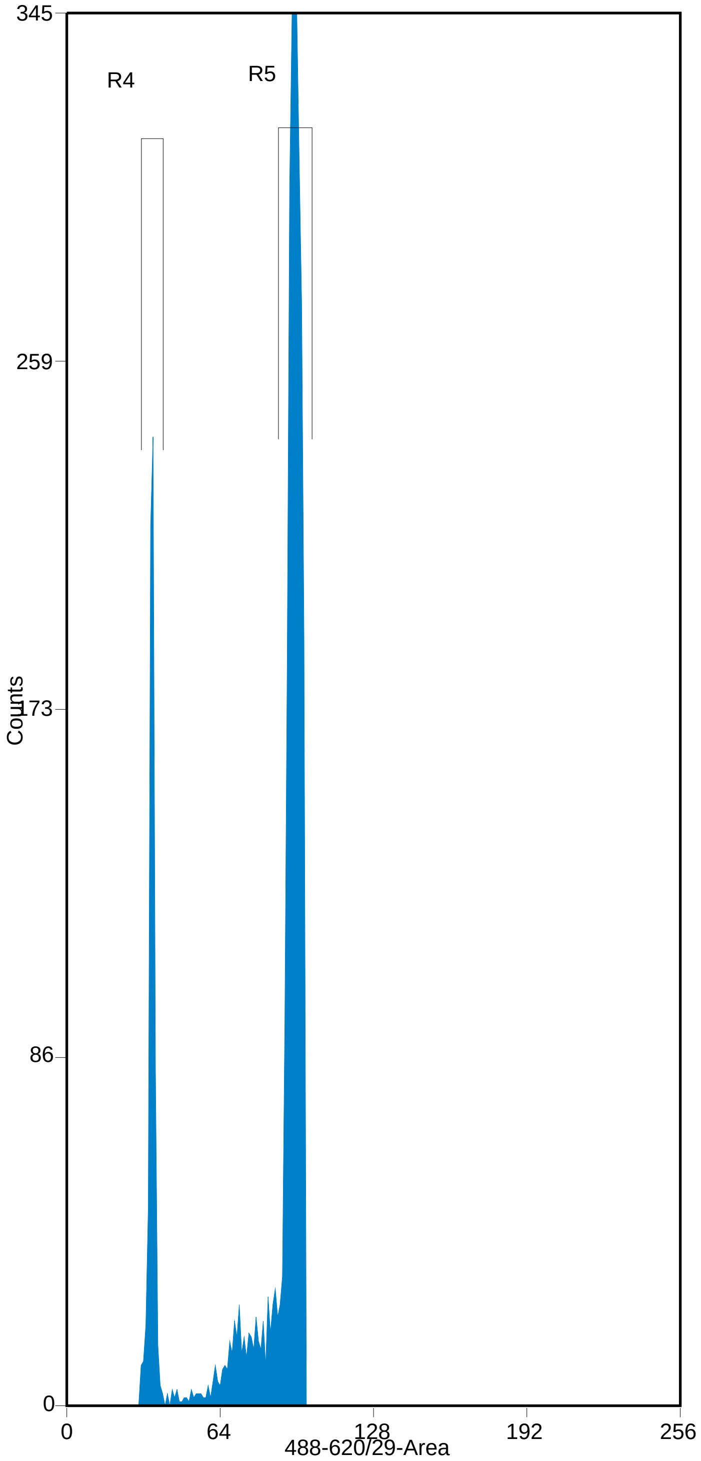

Region	Count	% Hist	Mean	CV
Total	3665	100.00	81.74	28.18
R4	654	17.84	35.50	3.64
R5	2545	69.44	95.07	2.52

spiderling of IIBCELB795_5

ADN_01_Apr_25_19 (G3: R1 & R2)

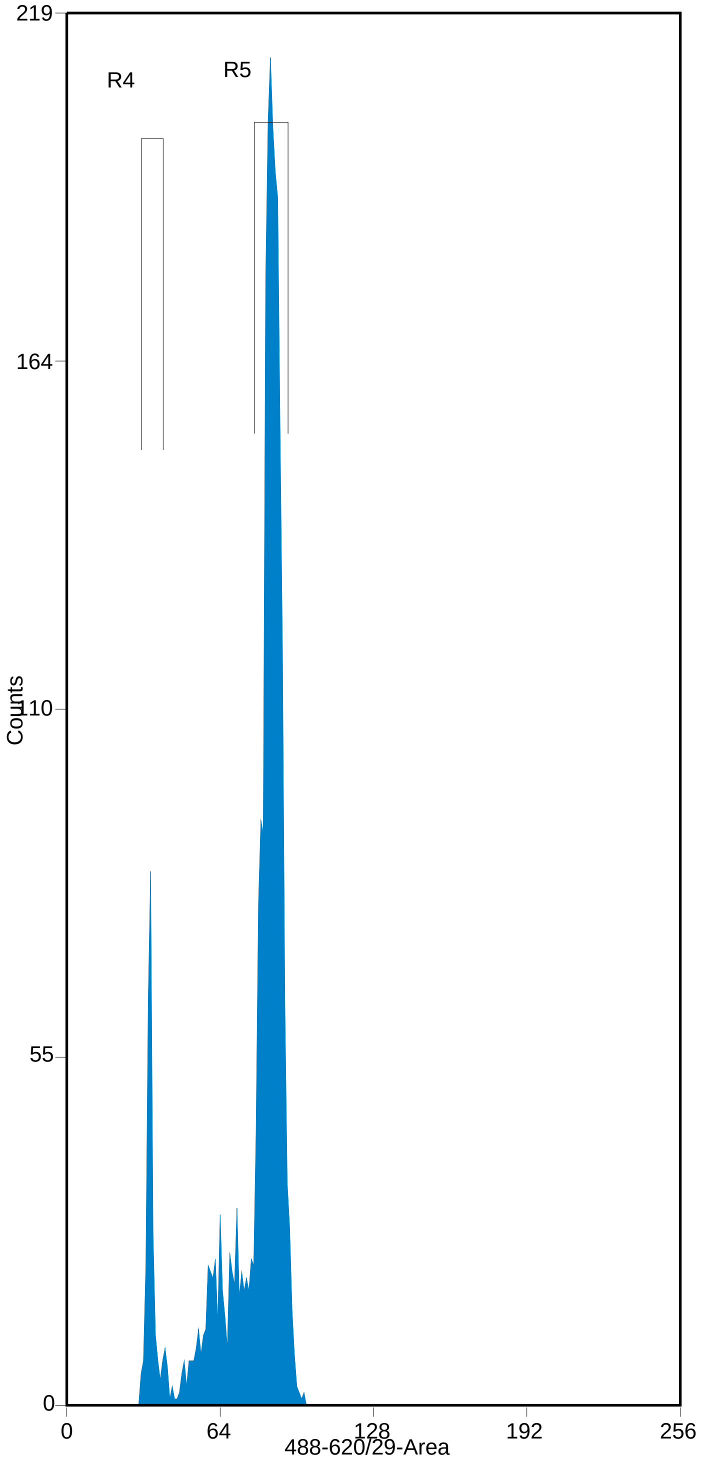

Region	Count	% Hist	Mean	CV
Total	2663	100.00	77.26	21.18
R4	238	8.94	34.88	4.79
R5	1873	70.33	85.53	3.76

BECKMAN COULTER

spiderling of IIBCELB795_6

ADN_01_Apr_25_20 (G3: R1 & R2)

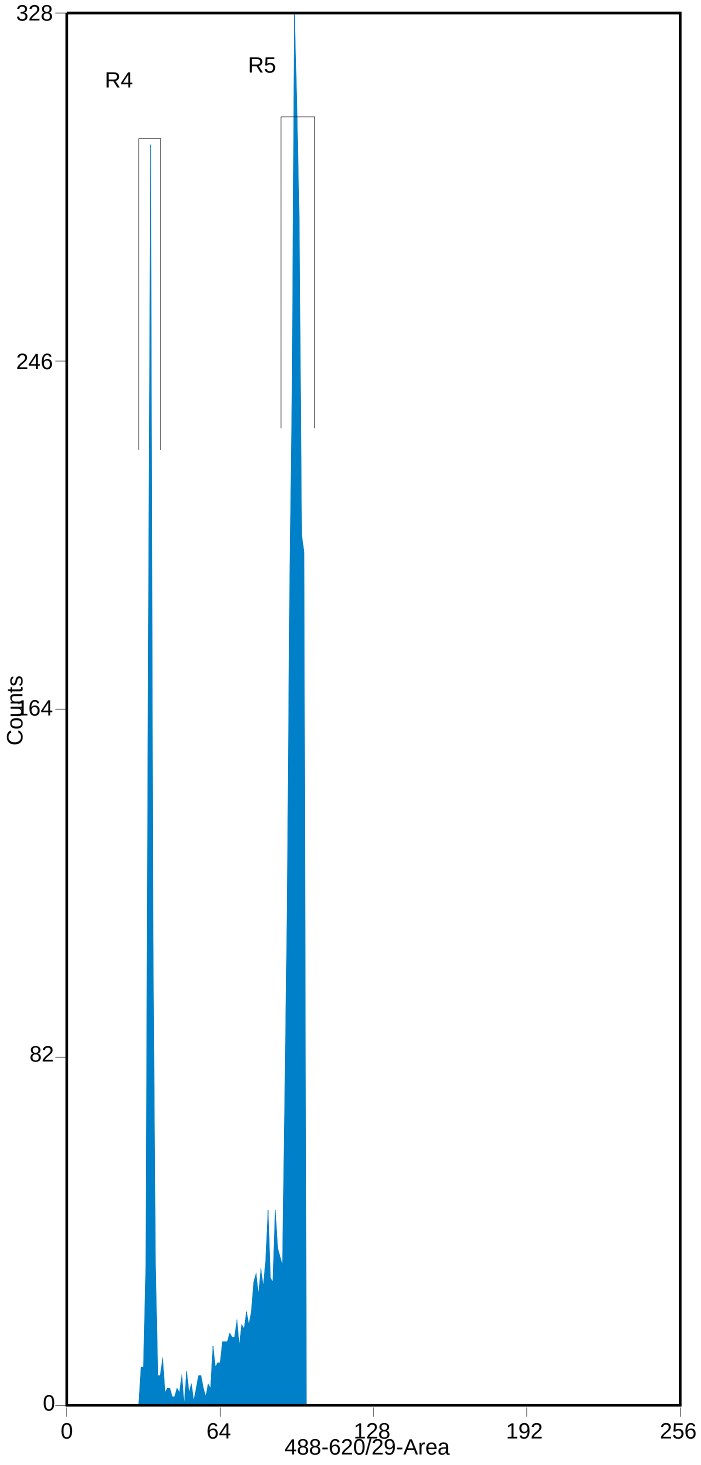

Region	Count	% Hist	Mean	CV
Total	3436	100.00	78.85	30.59
R4	682	19.85	34.88	3.37
R5	2025	58.93	95.31	2.51

spiderling of IIBCELB795_7

ADN_01_Apr_25_21 (G3: R1 & R2)

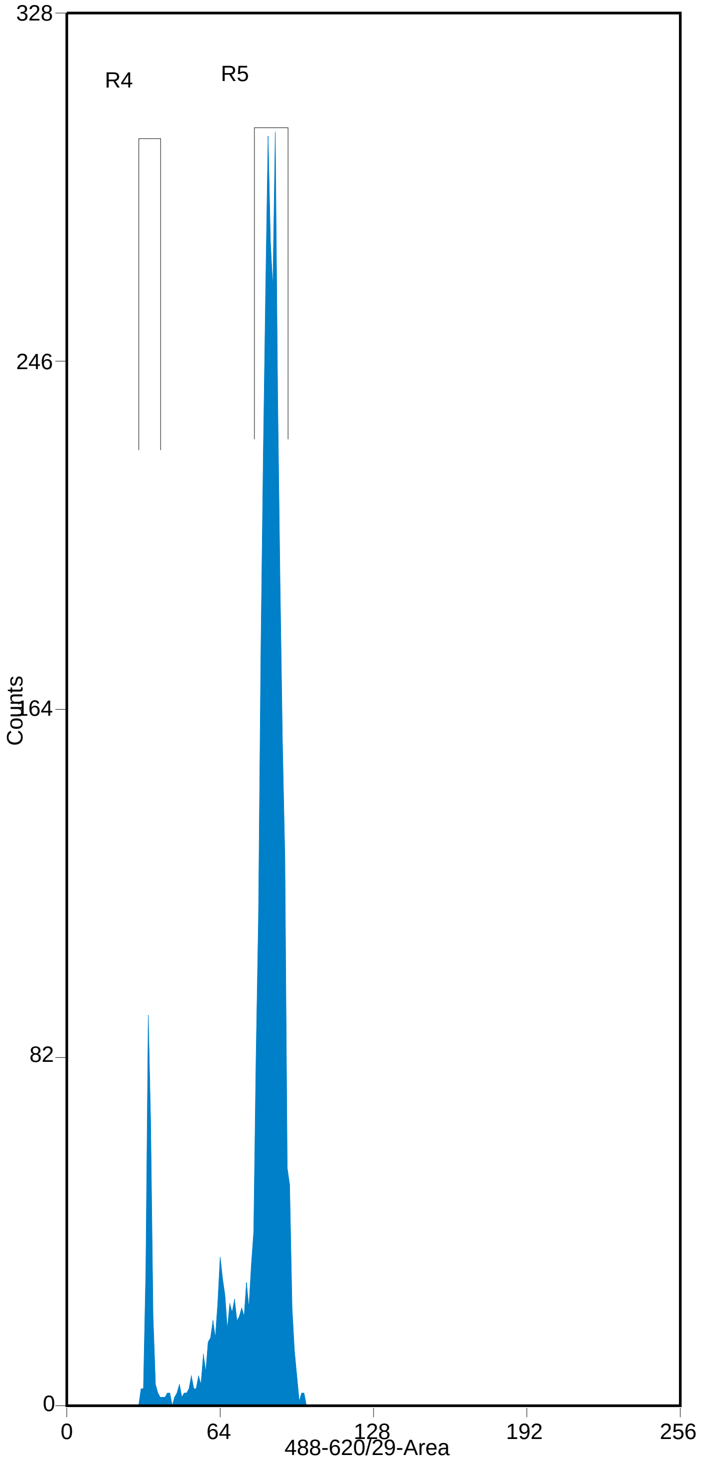

Region	Count	% Hist	Mean	CV
Total	3662	100.00	79.57	17.92
R4	230	6.28	34.41	3.53
R5	2810	76.73	85.26	3.95

spiderling of IIBCELB795_8

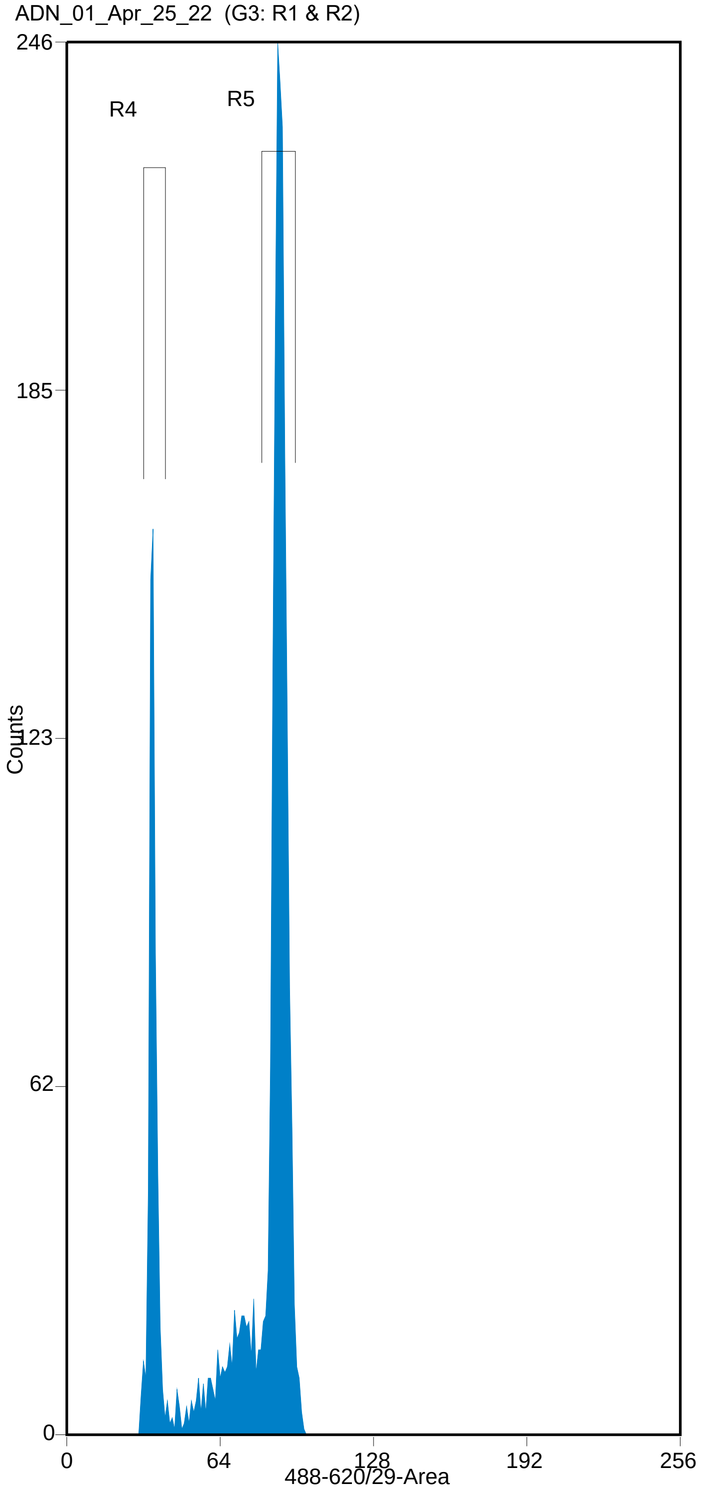

Region	Count	% Hist	Mean	CV
Total	2632	100.00	74.66	29.13
R4	538	20.44	35.93	4.26
R5	1658	62.99	88.91	3.05

spiderling of IIBCELB795_9

ADN_01_Apr_25_23 (G3: R1 & R2)

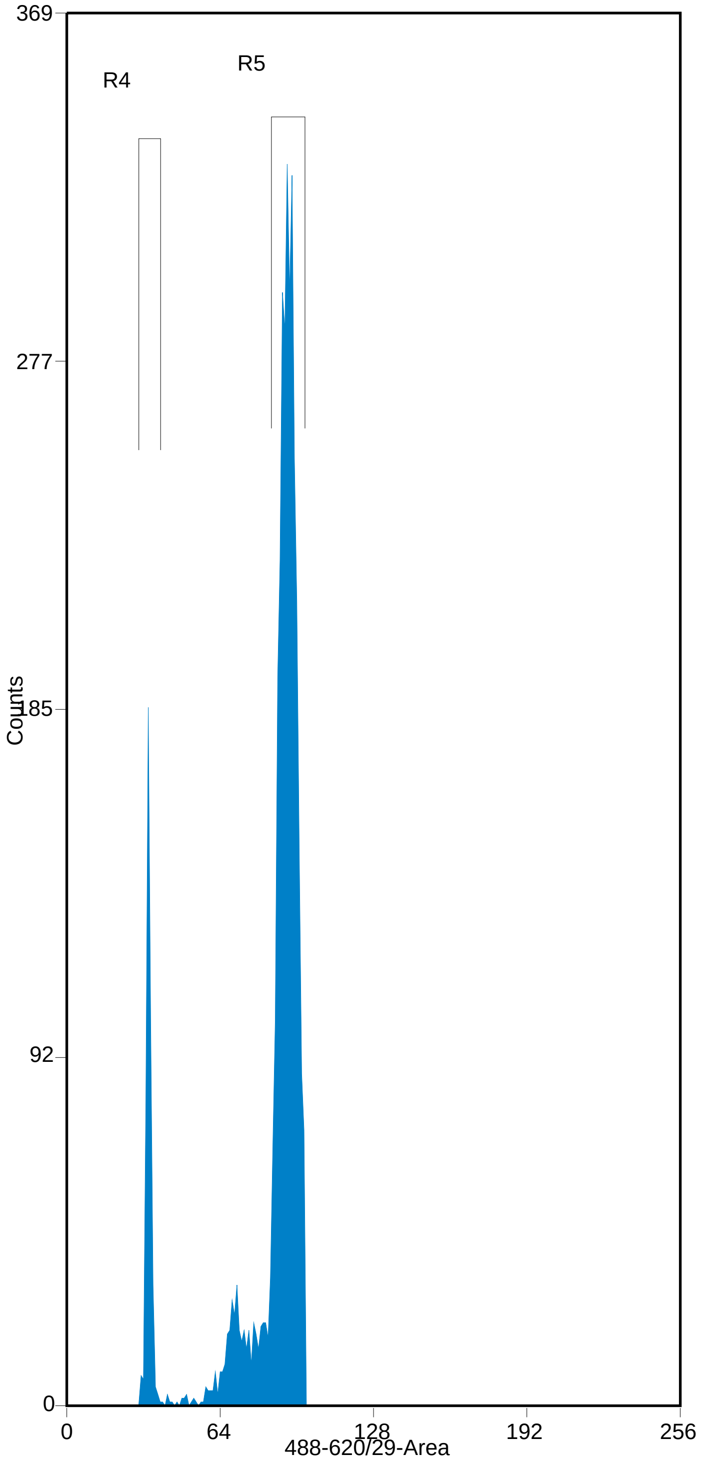

Region	Count	% Hist	Mean	CV
Total	3792	100.00	83.45	22.83
R4	425	11.21	34.19	3.22
R5	2924	77.11	92.29	3.51

spiderling of IIBCELB795_10

ADN_01_Apr_25_24 (G3: R1 & R2)

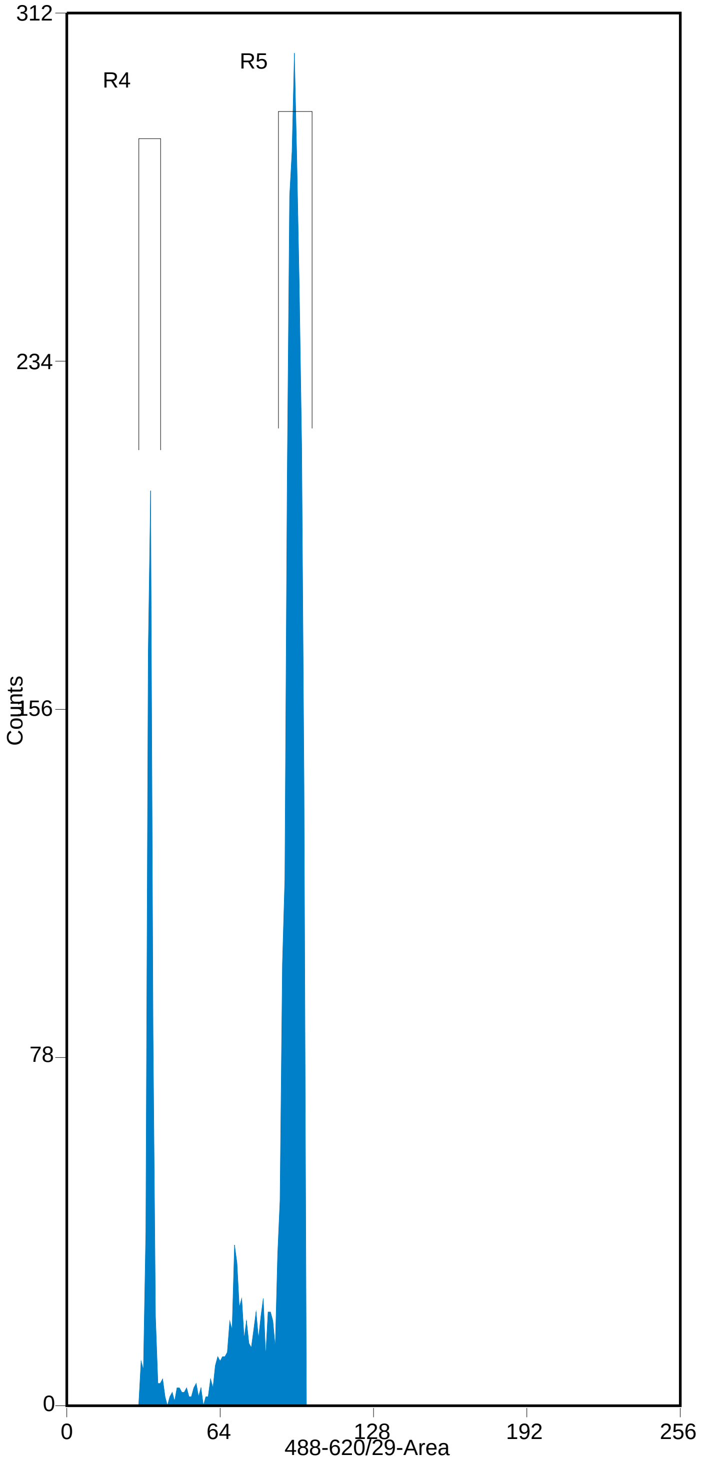

Region	Count	% Hist	Mean	CV
Total	3309	100.00	81.19	27.87
R4	544	16.44	34.72	3.50
R5	2243	67.78	94.58	2.82

BECKMAN COULTER

spiderling of IIBCELB795_11

ADN_01_Apr_25_25 (G3: R1 & R2)

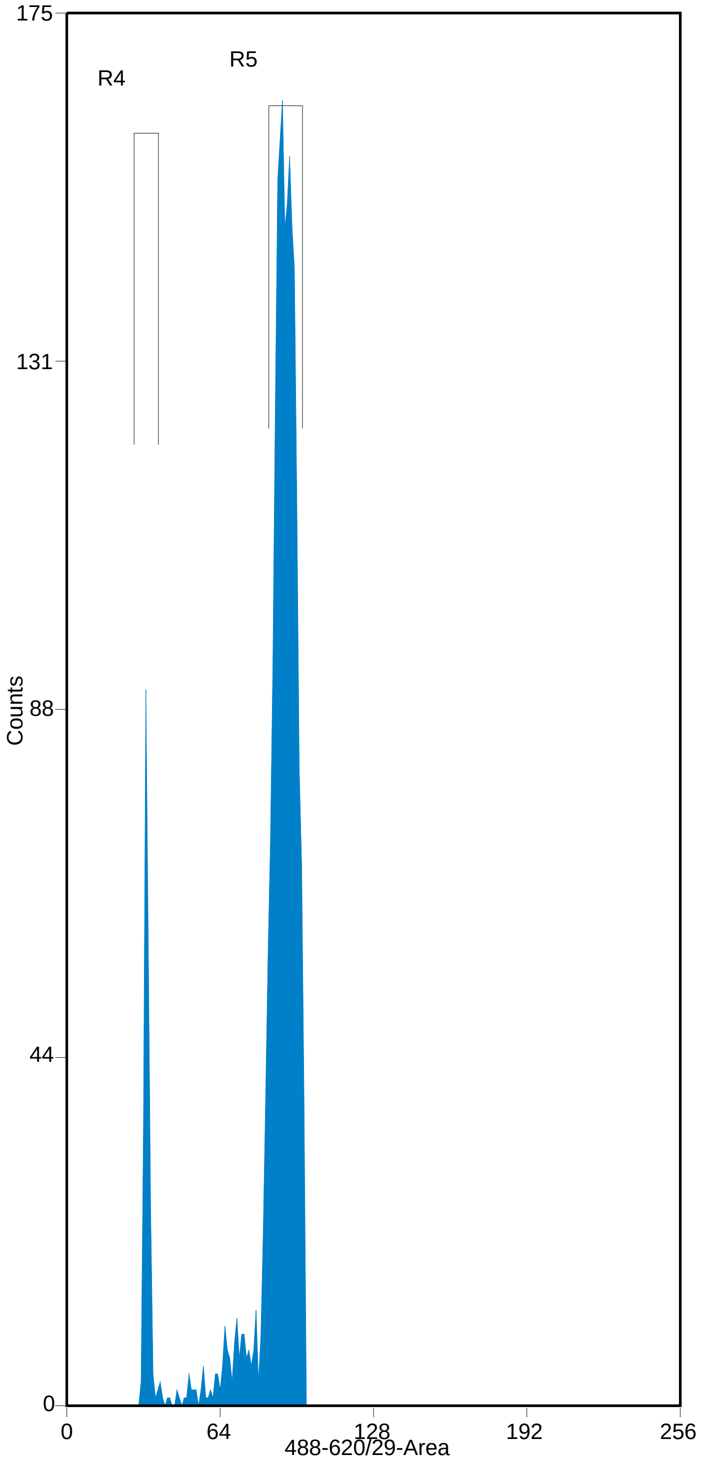

Region	Count	% Hist	Mean	CV
Total	2316	100.00	83.98	21.34
R4	218	9.41	33.43	3.31
R5	1832	79.10	91.15	4.08

BECKMAN COULTER

spiderling of IIBCELB795_12

ADN_01_Apr_25_26 (G3: R1 & R2)

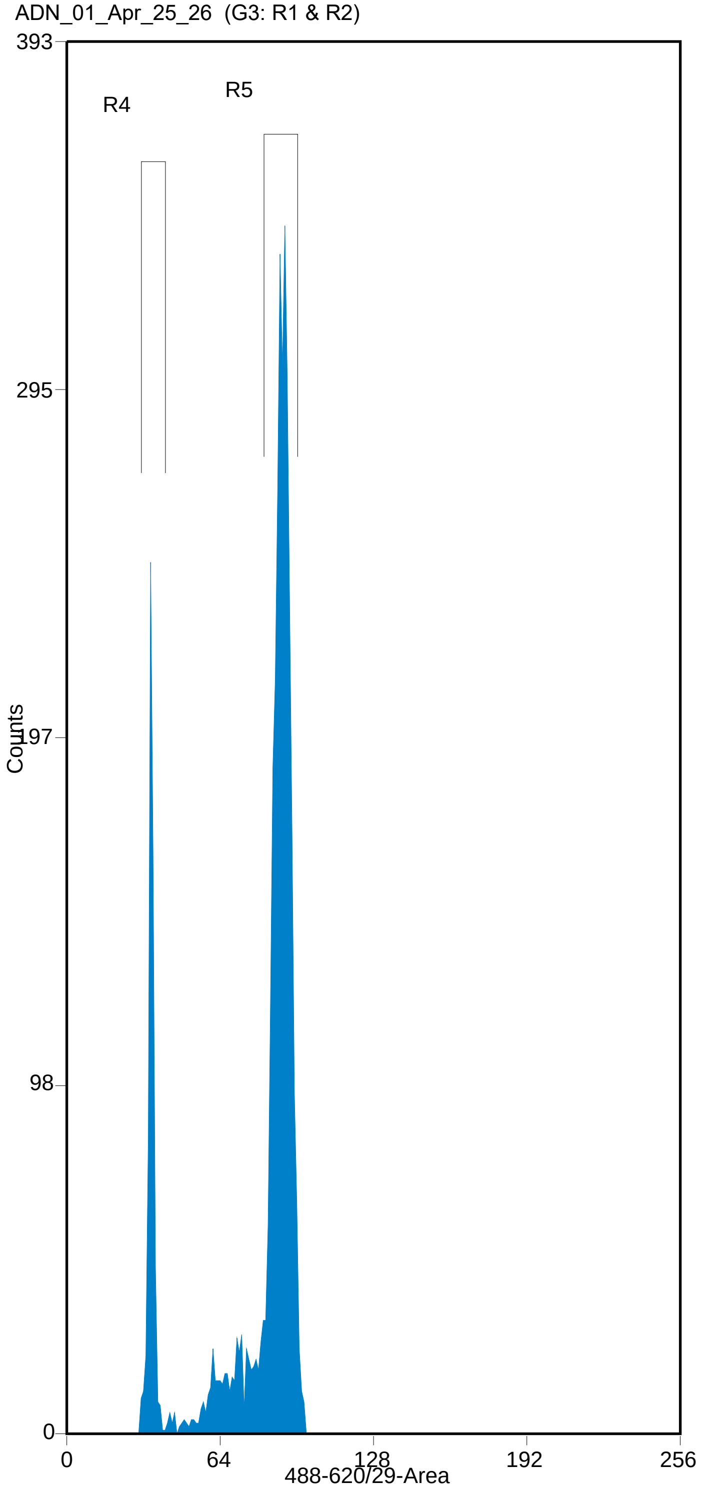

Region	Count	% Hist	Mean	CV
Total	3881	100.00	78.71	25.97
R4	603	15.54	35.21	3.72
R5	2749	70.83	89.82	3.43

BECKMAN COULTER

spiderling of IIBCELB795_13

ADN_01_Apr_25_27 (G3: R1 & R2)

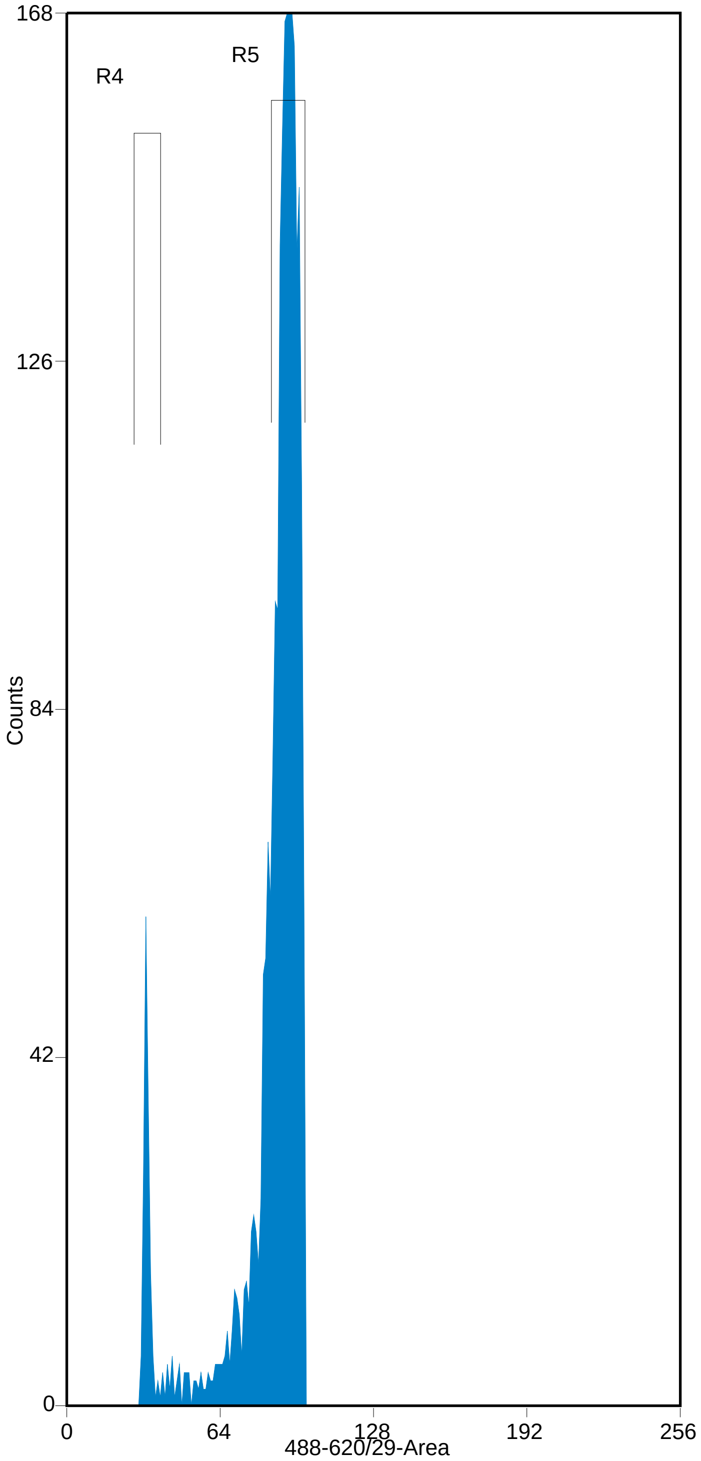

Region	Count	% Hist	Mean	CV
Total	2599	100.00	85.47	18.75
R4	157	6.04	33.46	4.14
R5	1962	75.49	92.39	4.00

spiderling of IIBCELB795_14

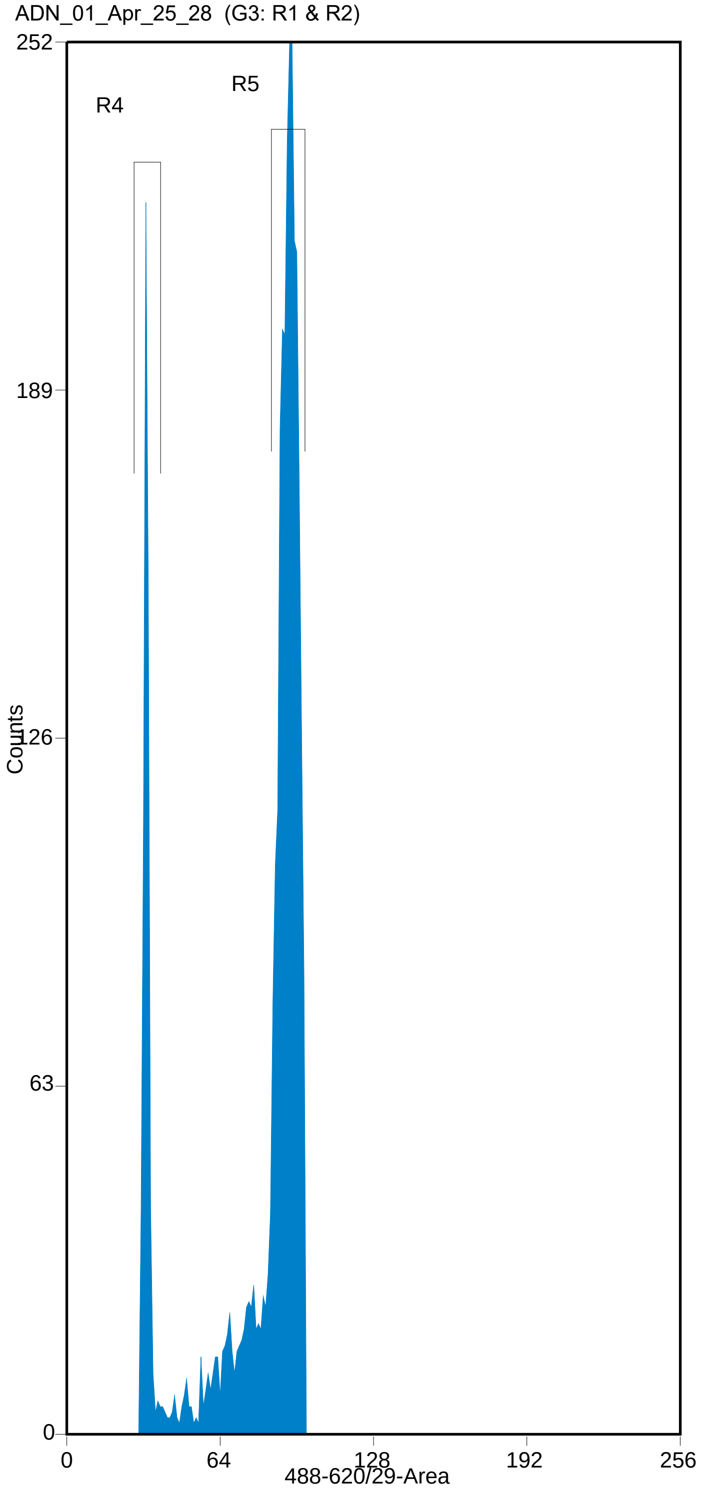

Region	Count	% Hist	Mean	CV
Total	3628	100.00	79.33	28.54
R4	595	16.40	33.26	3.99
R5	2475	68.22	92.65	3.78

spiderling of IIBCELB795_15

ADN_01_Apr_25_29 (G3: R1 & R2)

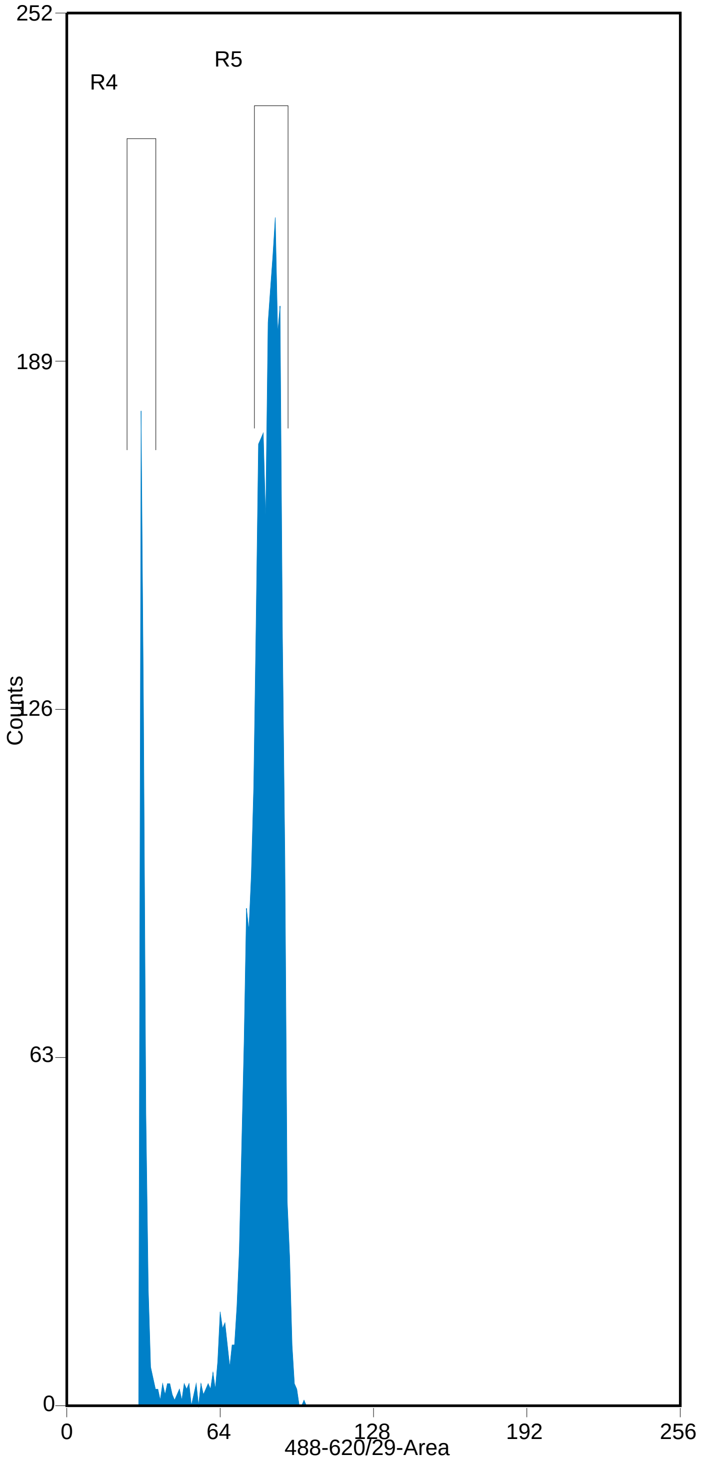

Region	Count	% Hist	Mean	CV
Total	3463	100.00	76.41	22.92
R4	396	11.44	31.93	3.63
R5	2427	70.08	84.72	4.47

ADN_31_Jul_23_1 (G3: R1 & R2)

AmC6SJC_1

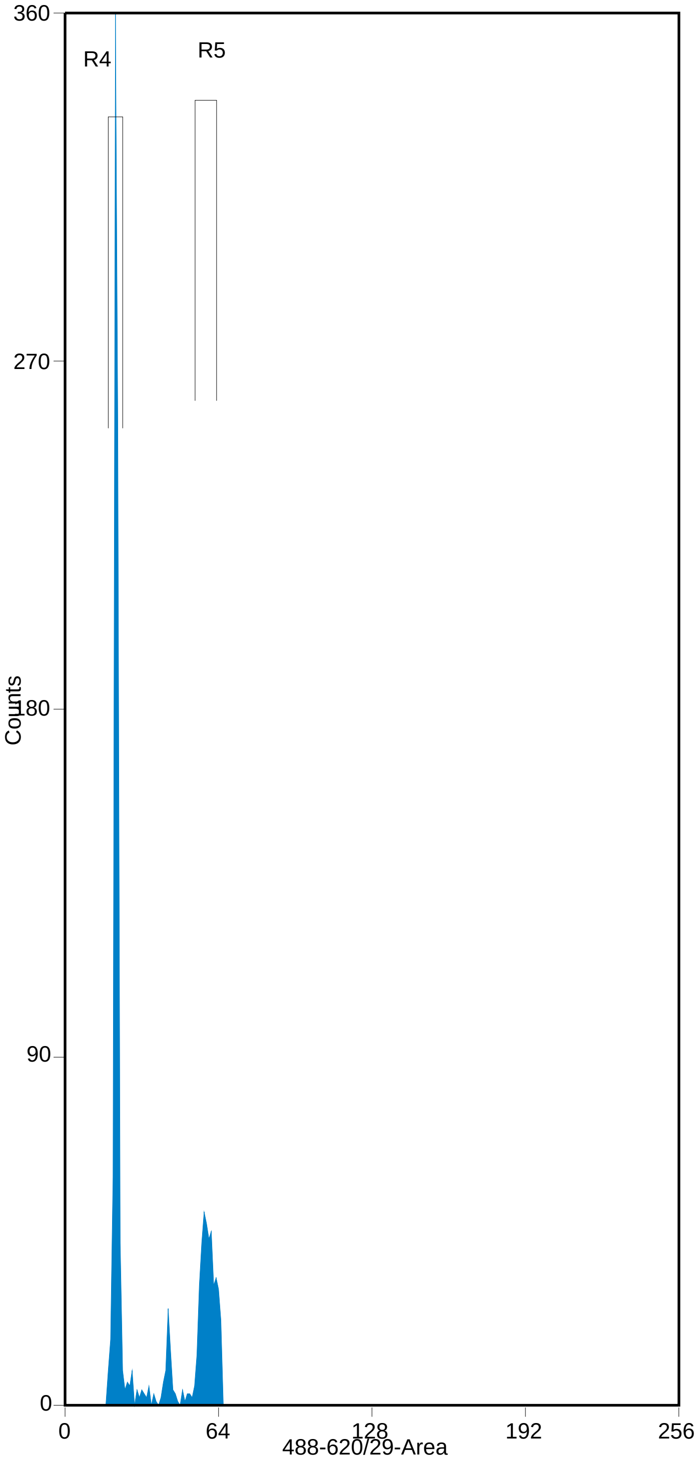

Region	Count	% Hist	Mean	CV
Total	1348	100.00	34.14	51.11
R4	831	61.65	21.29	4.12
R5	340	25.22	59.16	3.94

AmC6SJC_2

ADN_31_Jul_23_2 (G3: R1 & R2)

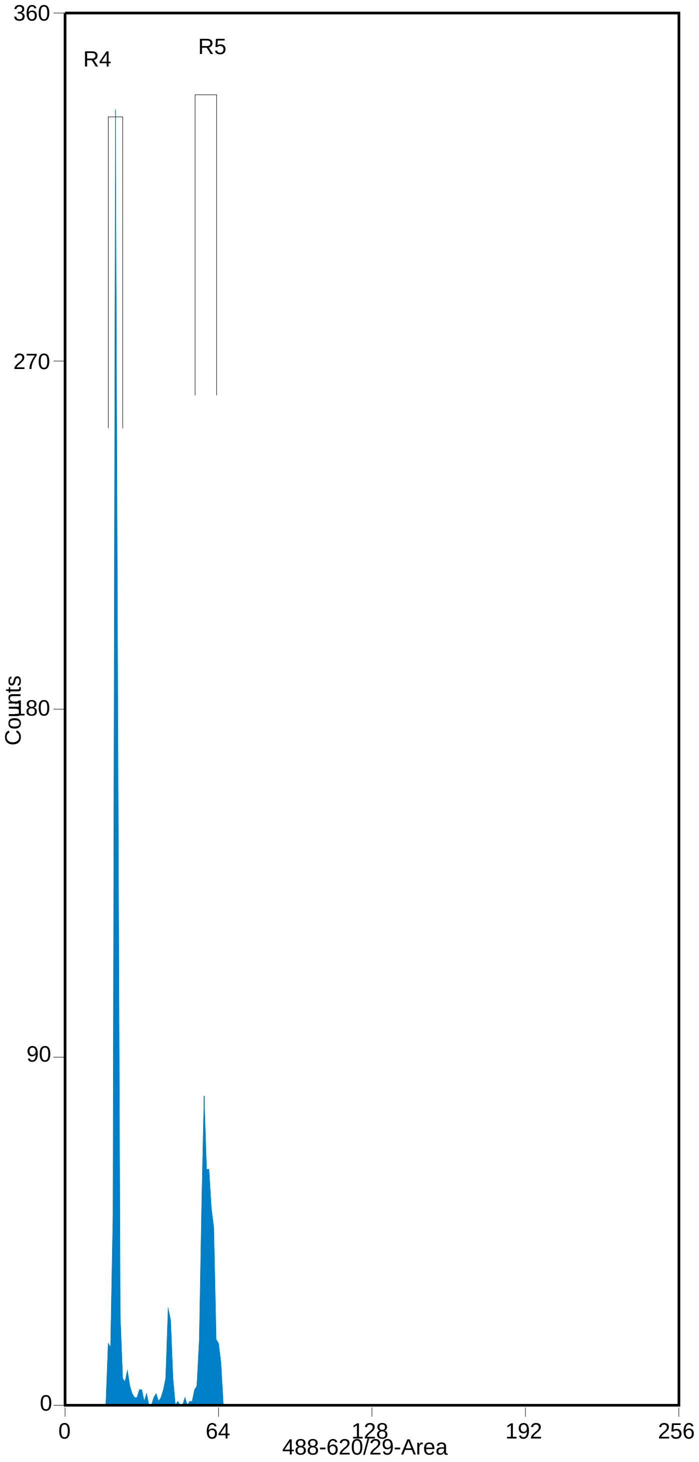

Region	Count	% Hist	Mean	CV
Total	1162	100.00	36.82	49.11
R4	624	53.70	21.19	4.49
R5	394	33.91	59.24	3.35

AmC3SJC

ADN_28_Jul_23_4 (G3: R1 & R2)

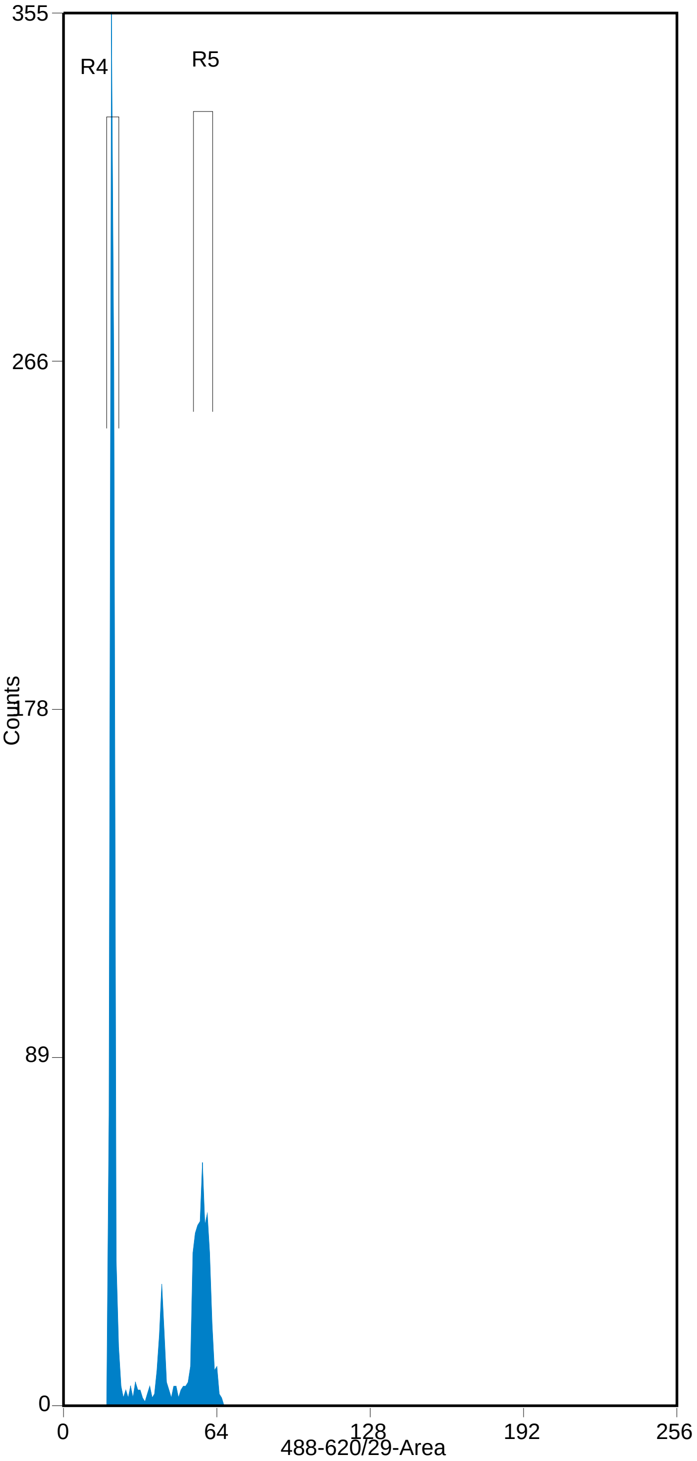

Region	Count	% Hist	Mean	CV
Total	1540	100.00	32.92	51.25
R4	942	61.17	20.33	3.65
R5	393	25.52	57.79	4.06

ADN_31_Jul_23_5 (G3: R1 & R2)

spiderling of AmC3_1

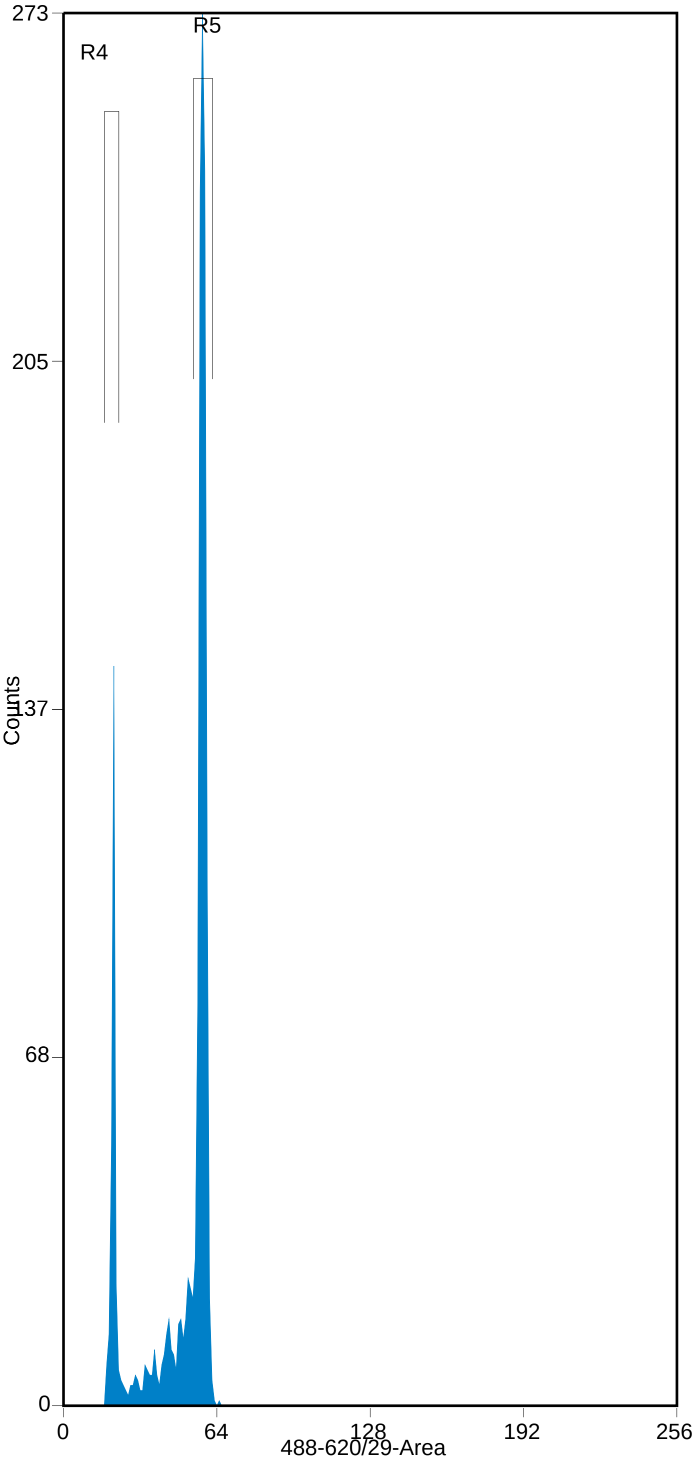

Region	Count	% Hist	Mean	CV
Total	1542	100.00	49.34	28.74
R4	249	16.15	20.74	4.47
R5	1016	65.89	57.97	2.47

ADN_31_Jul_23_6 (G3: R1 & R2)

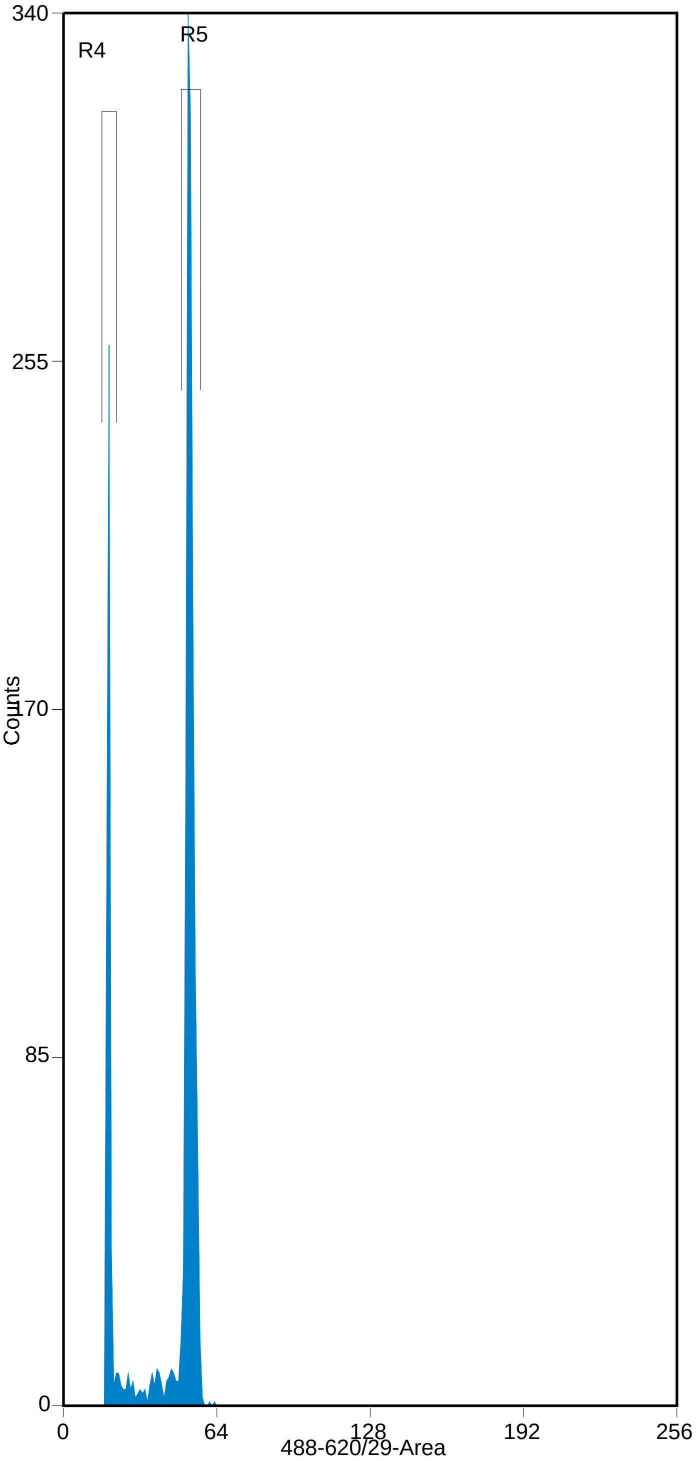

spiderling of AmC3_2

Region	Count	% Hist	Mean	CV
Total	1828	100.00	43.43	33.94
R4	441	24.12	18.87	4.02
R5	1243	68.00	52.88	2.91

spiderling of AmC3_3

ADN_31_Jul_23_7 (G3: R1 & R2)

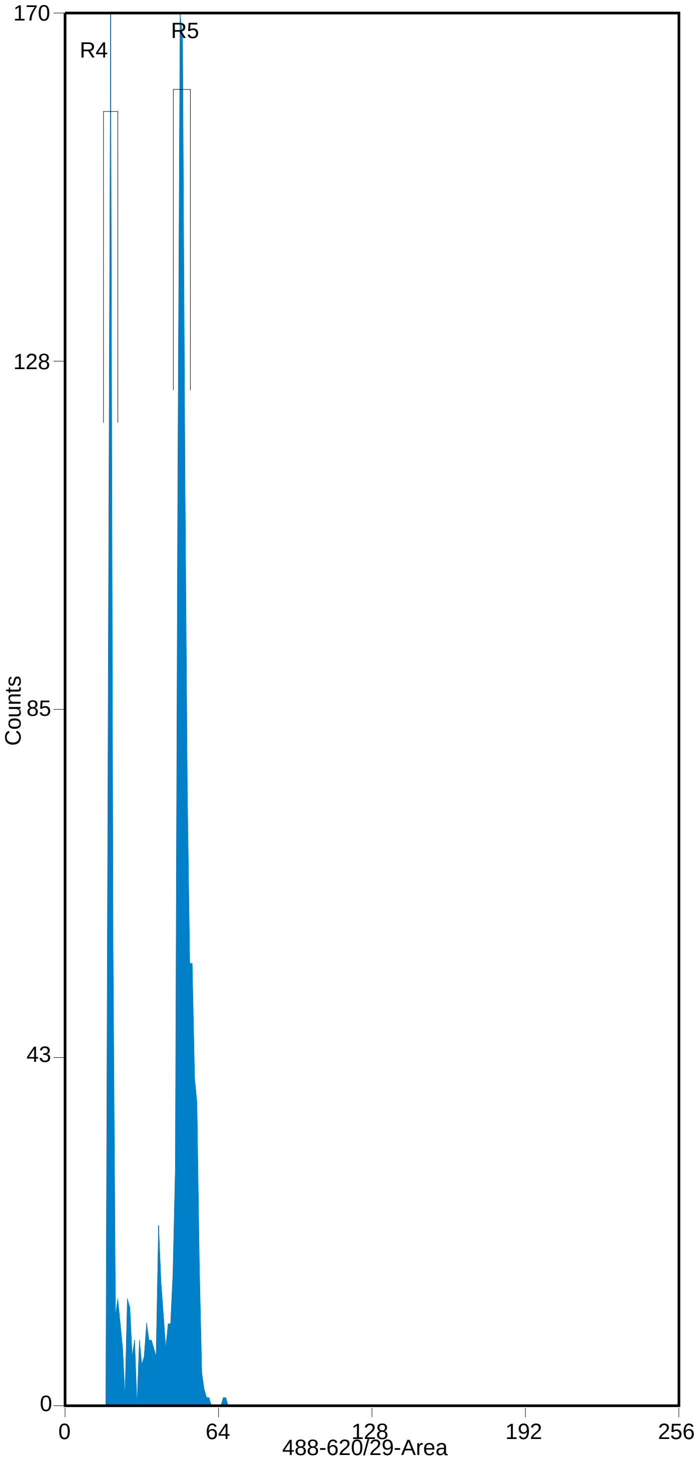

Region	Count	% Hist	Mean	CV
Total	1512	100.00	40.00	33.94
R4	386	25.53	19.08	4.64
R5	778	51.46	48.79	3.32

BECKMAN COULTER

spiderling of AmC3_4

ADN_31_Jul_23_8 (G3: R1 & R2)

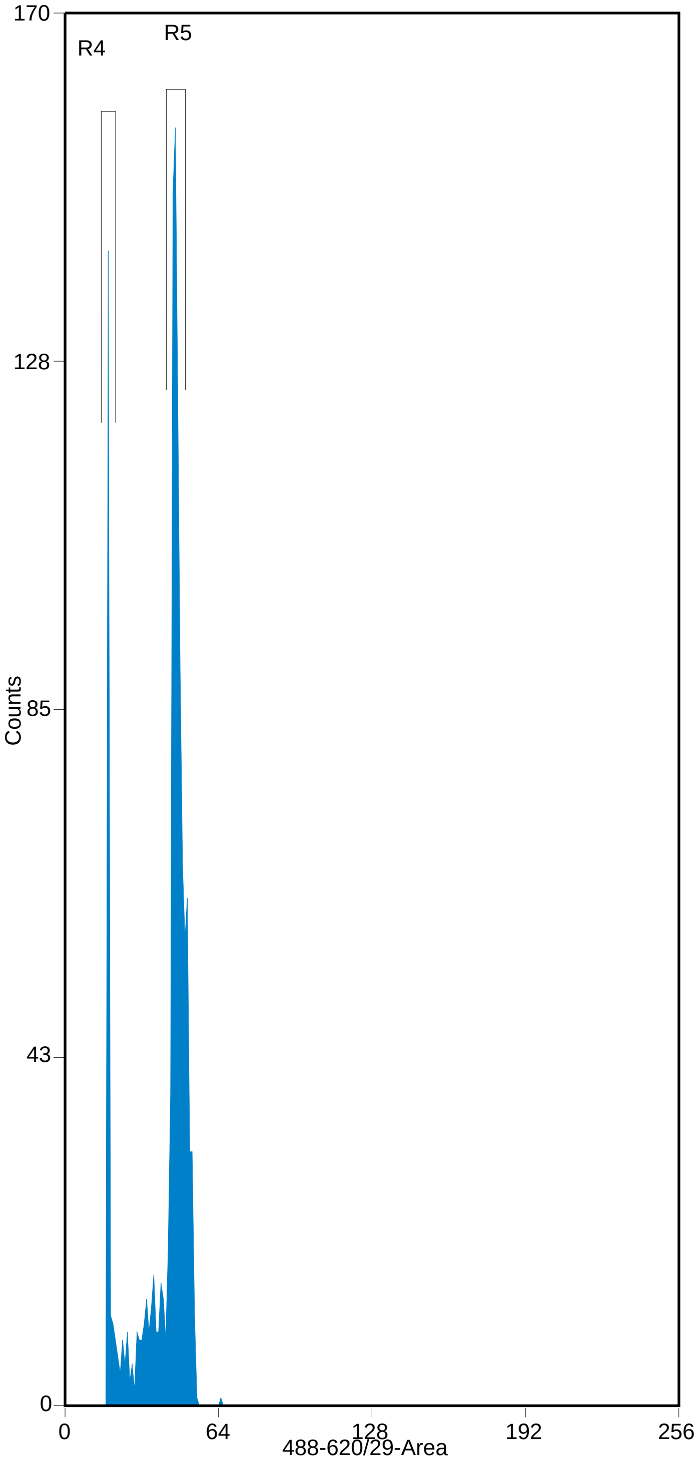

Region	Count	% Hist	Mean	CV
Total	1185	100.00	41.23	26.78
R4	170	14.35	18.32	4.29
R5	705	59.49	46.59	3.91

spiderling of AmC3_5

ADN_31_Jul_23_9 (G3: R1 & R2)

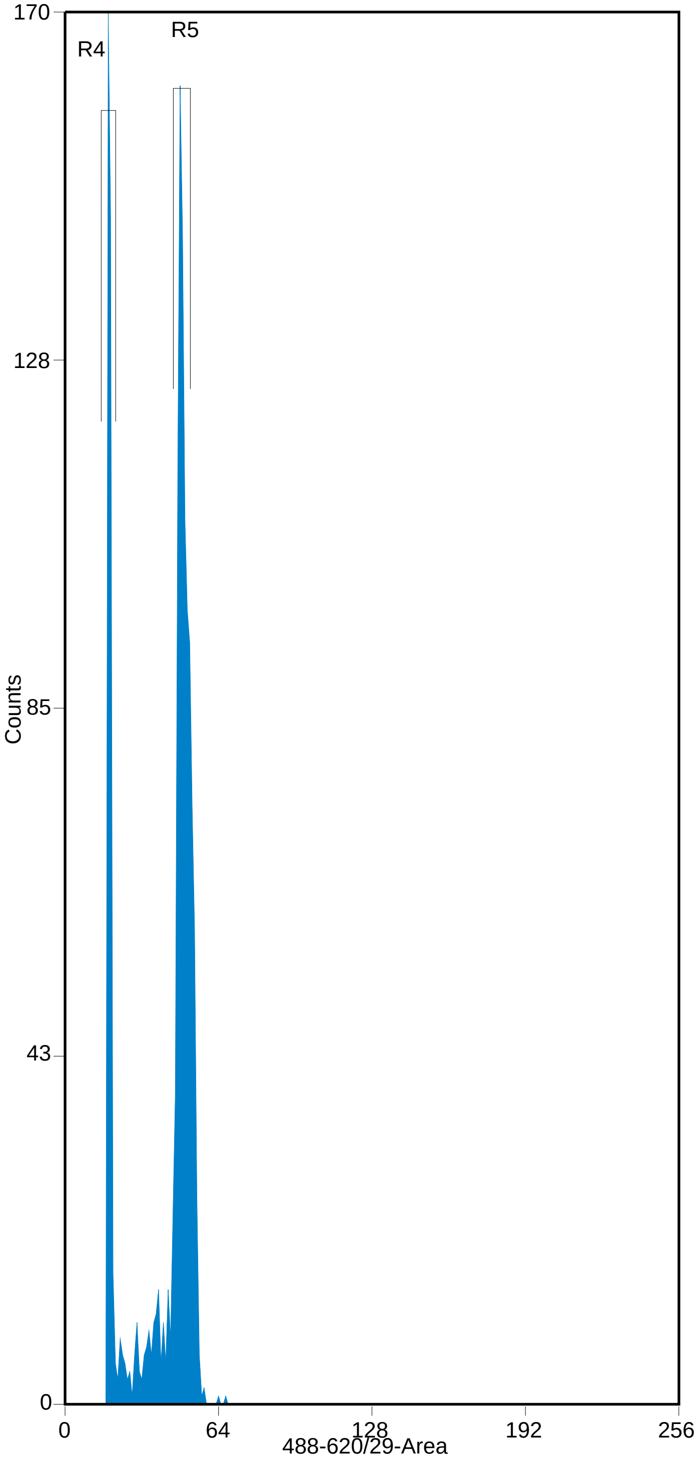

Region	Count	% Hist	Mean	CV
Total	1451	100.00	40.70	33.48
R4	349	24.05	18.55	3.51
R5	777	53.55	48.98	3.78

spiderling of AmC3_6

ADN_31_Jul_23_10 (G3: R1 & R2)

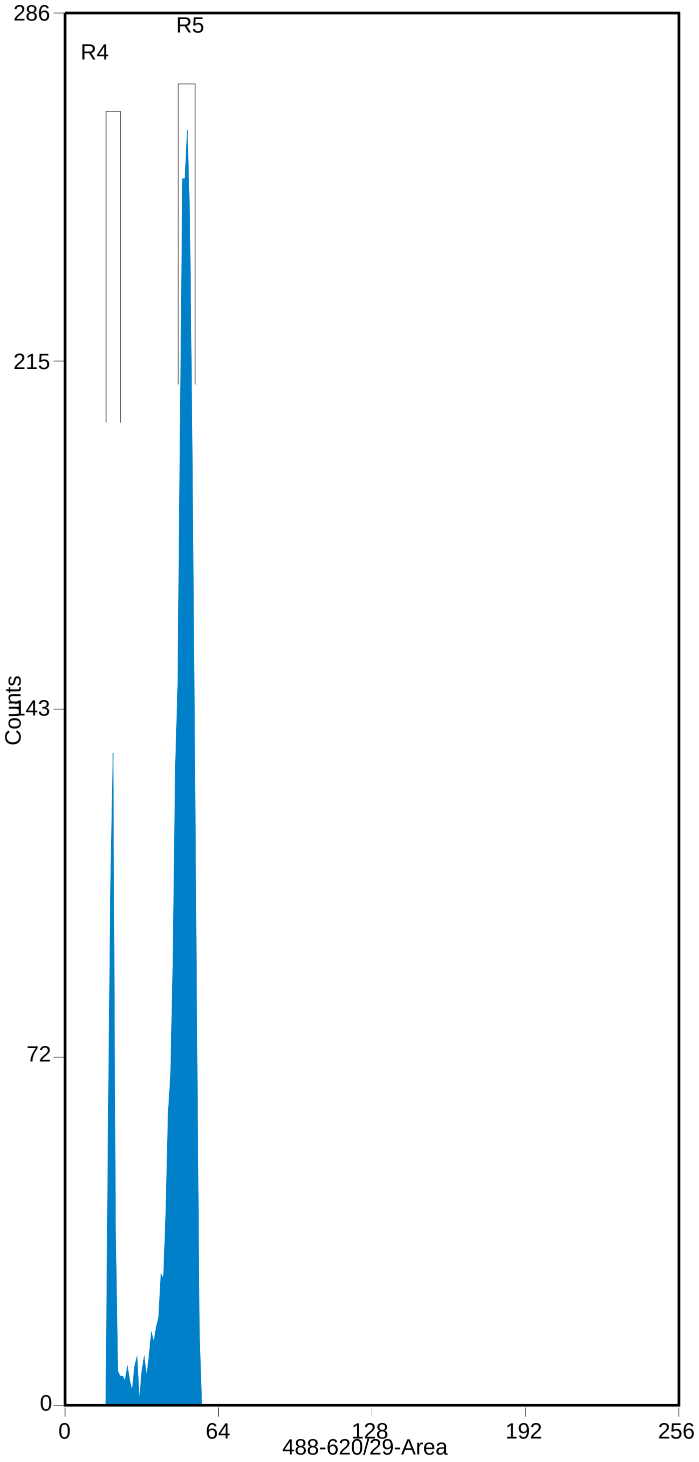

Region	Count	% Hist	Mean	CV
Total	2716	100.00	44.71	24.21
R4	350	12.89	19.55	5.42
R5	1693	62.33	50.47	4.09

spiderling of AmC3_7

ADN_31_Jul_23_11 (G3: R1 & R2)

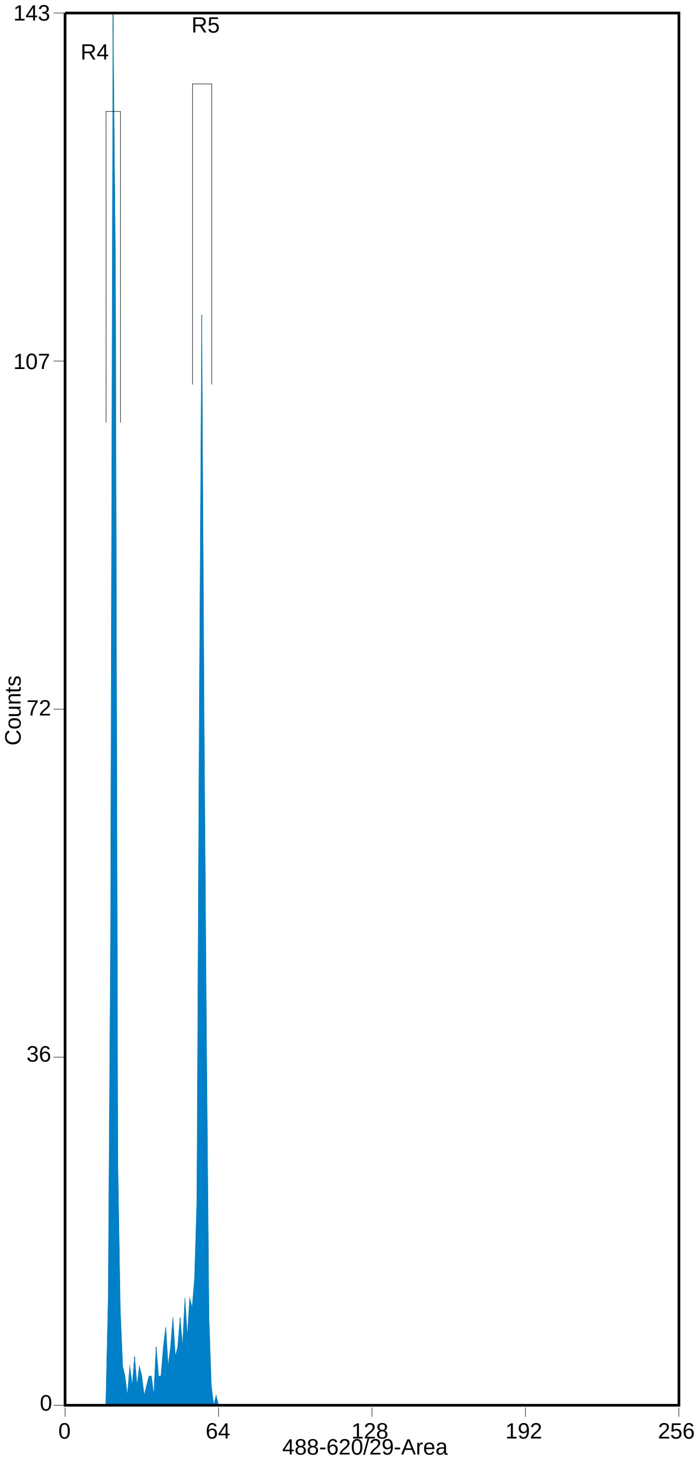

Region	Count	% Hist	Mean	CV
Total	852	100.00	38.67	44.43
R4	368	43.19	20.35	4.83
R5	345	40.49	56.96	2.61

ADN_31_Jul_23_12 (G3: R1 & R2)

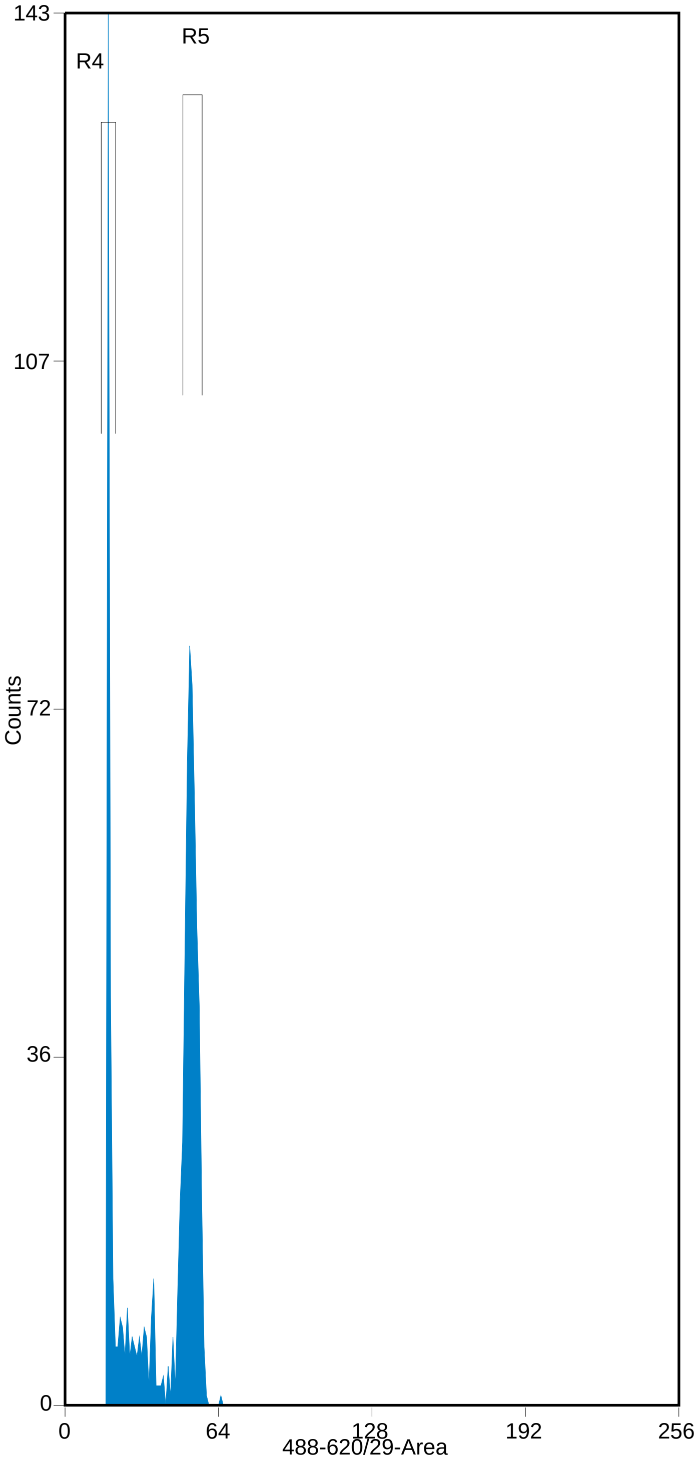

spiderling of AmC3_8

Region	Count	% Hist	Mean	CV
Total	954	100.00	38.39	41.62
R4	313	32.81	18.27	3.45
R5	465	48.74	52.80	4.06

ADN_31_Jul_23_14 (G3: R1 & R2)

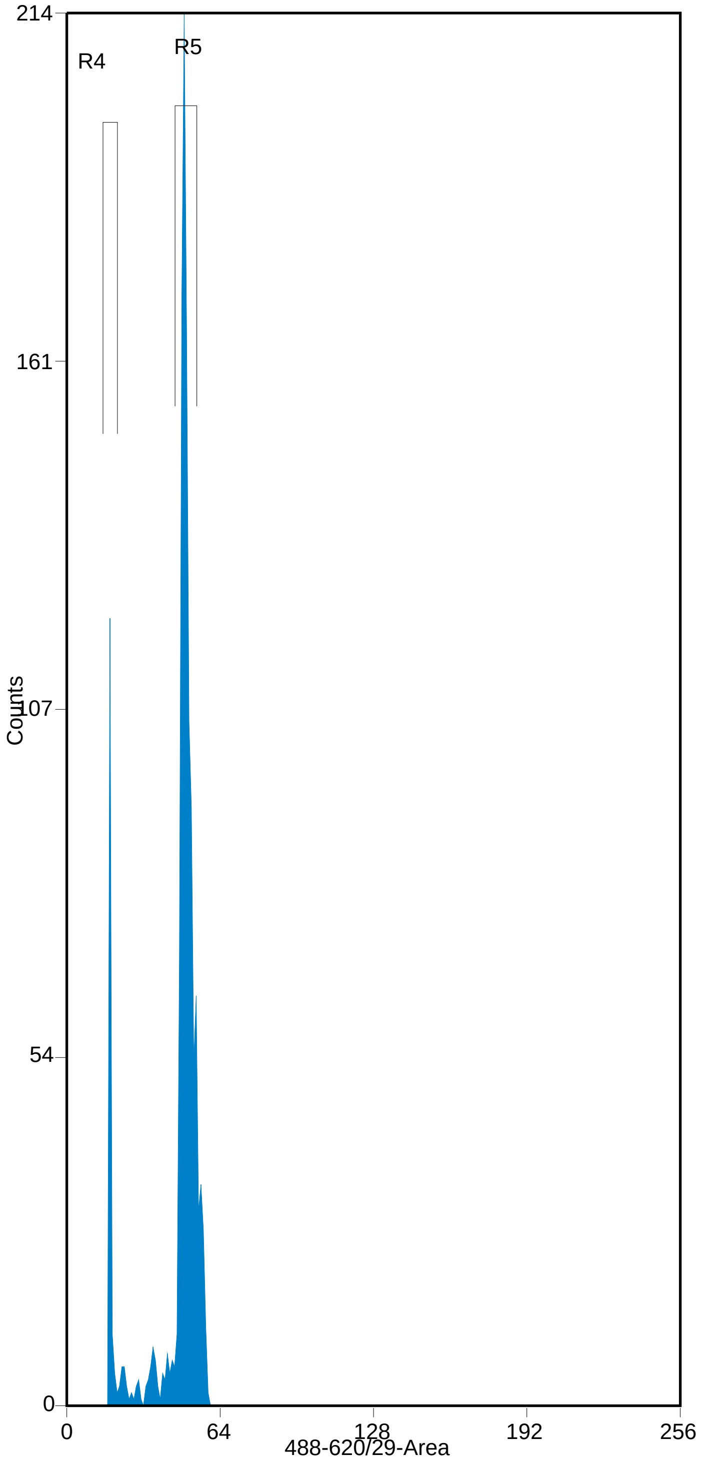

spiderling of AmC3_9

Region	Count	% Hist	Mean	CV
Total	1297	100.00	45.88	23.70
R4	139	10.72	18.19	3.08
R5	961	74.09	49.84	3.99

ADN_31_Jul_23_15 (G3: R1 & R2)

spiderling of AmC3_10

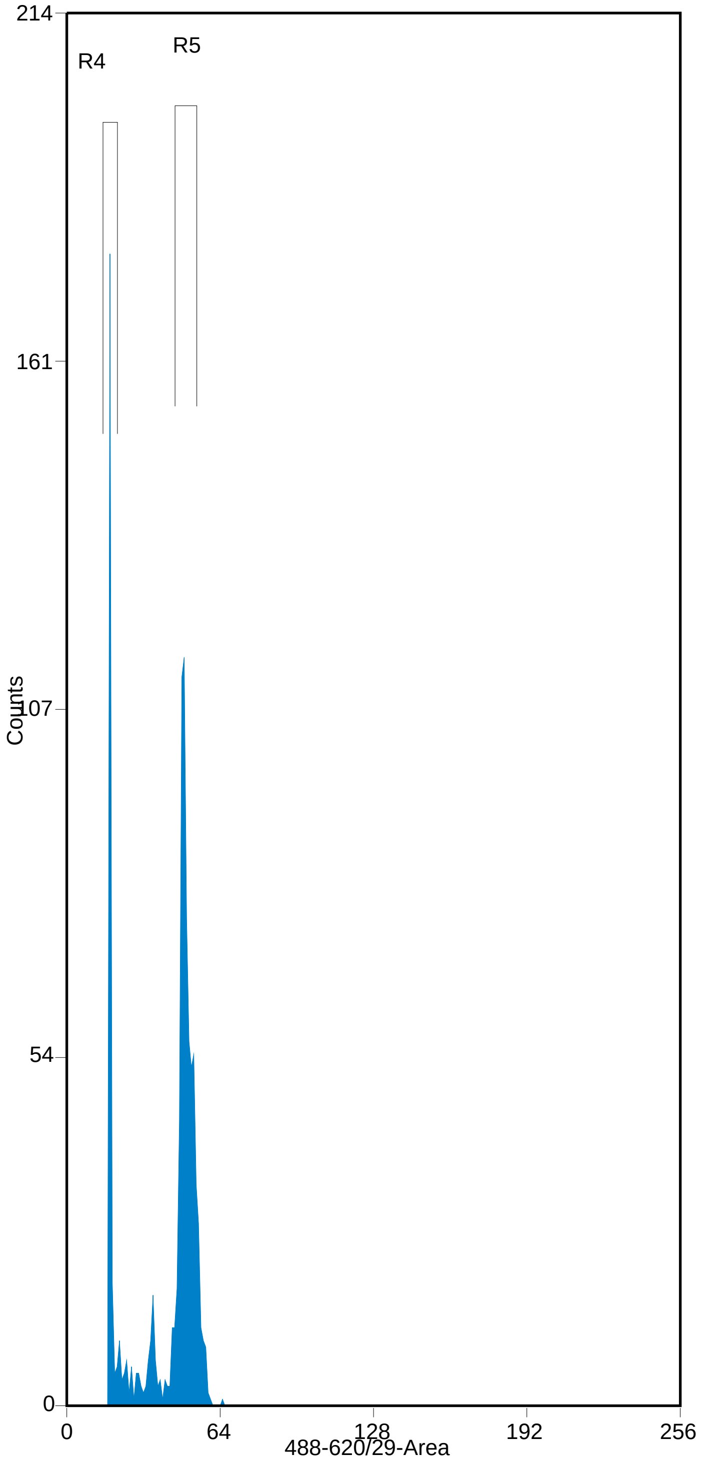

Region	Count	% Hist	Mean	CV
Total	966	100.00	41.30	33.25
R4	207	21.43	18.23	3.46
R5	572	59.21	49.75	4.45

spiderling of AmC3_11

ADN_31_Jul_23_16 (G3: R1 & R2)

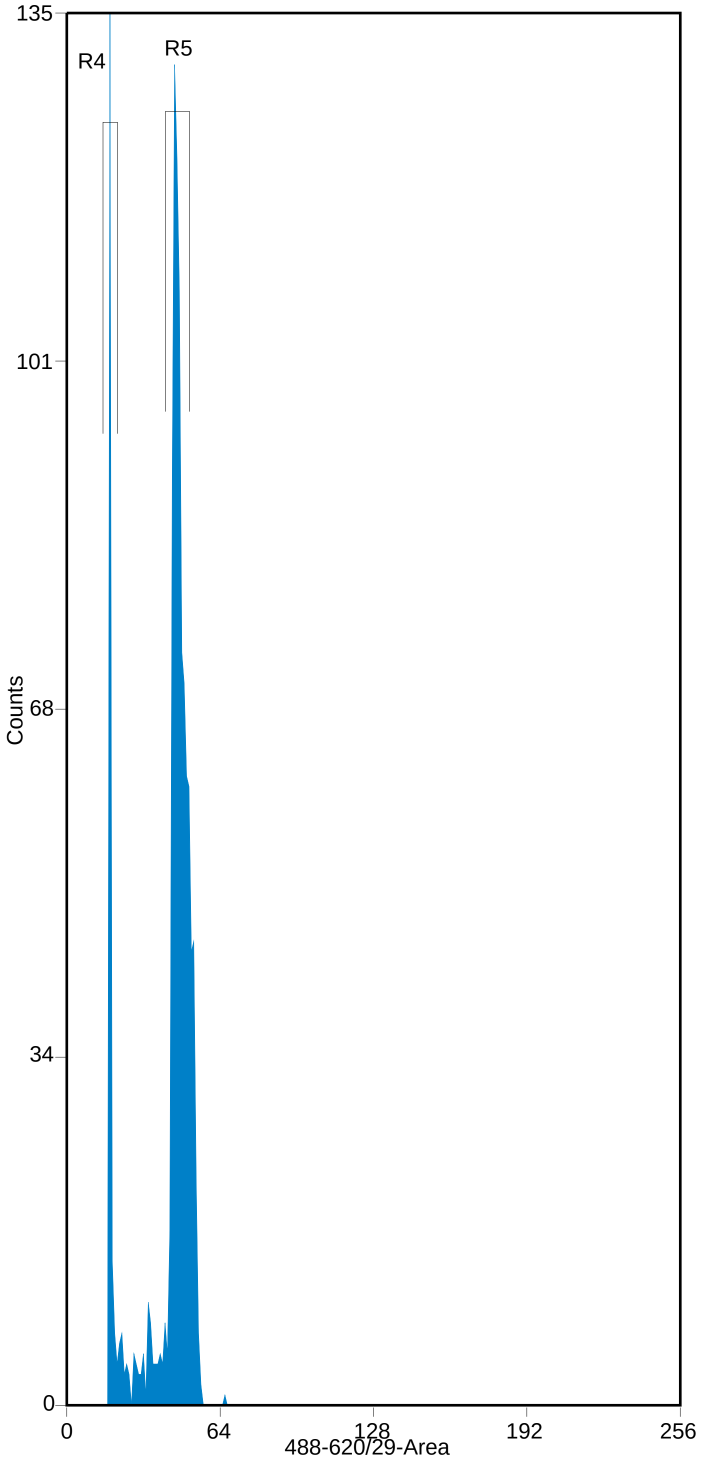

Region	Count	% Hist	Mean	CV
Total	1112	100.00	42.08	26.82
R4	165	14.84	18.24	3.53
R5	743	66.82	46.79	4.94

spiderling of AmC3_12

ADN_31_Jul_23_17 (G3: R1 & R2)

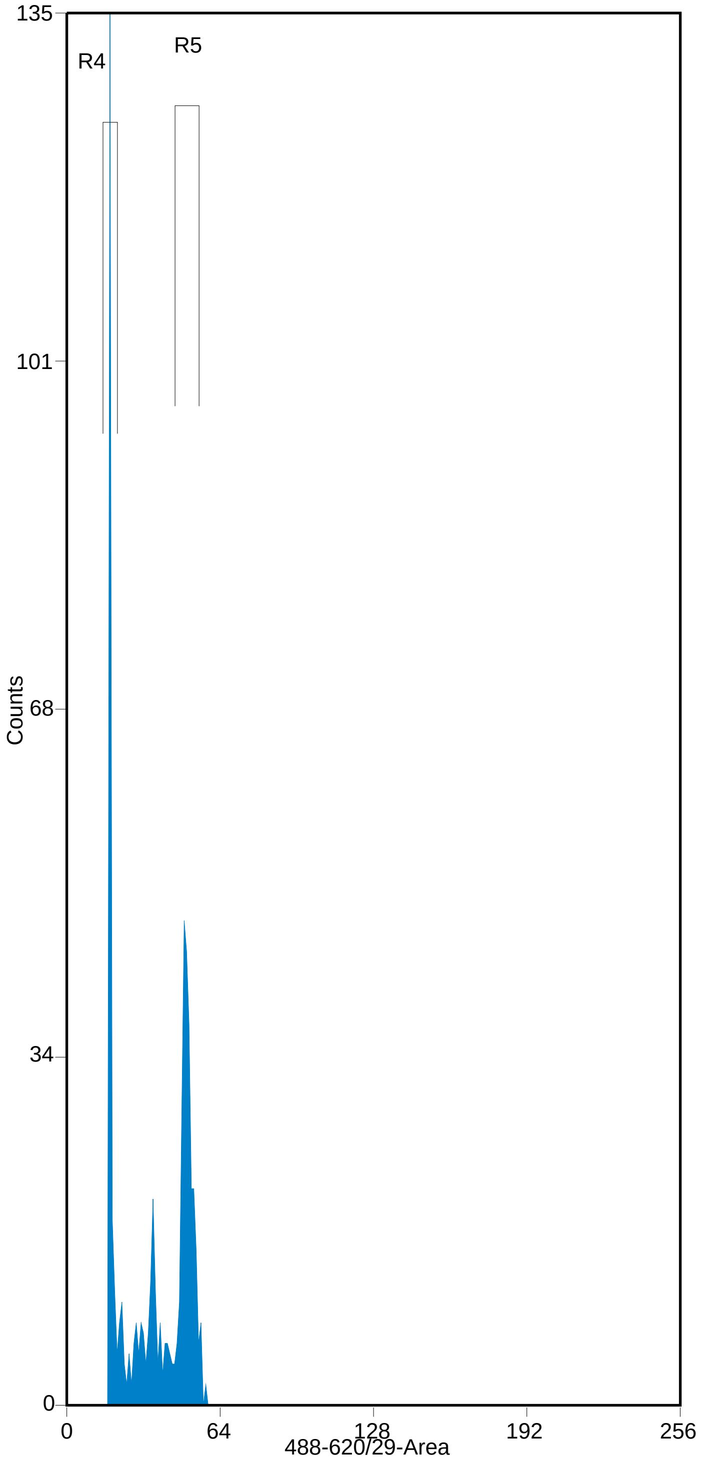

Region	Count	% Hist	Mean	CV
Total	592	100.00	35.79	39.72
R4	187	31.59	18.29	3.81
R5	240	40.54	50.22	4.33

ADN_31_Jul_23_19 (G3: R1 & R2)

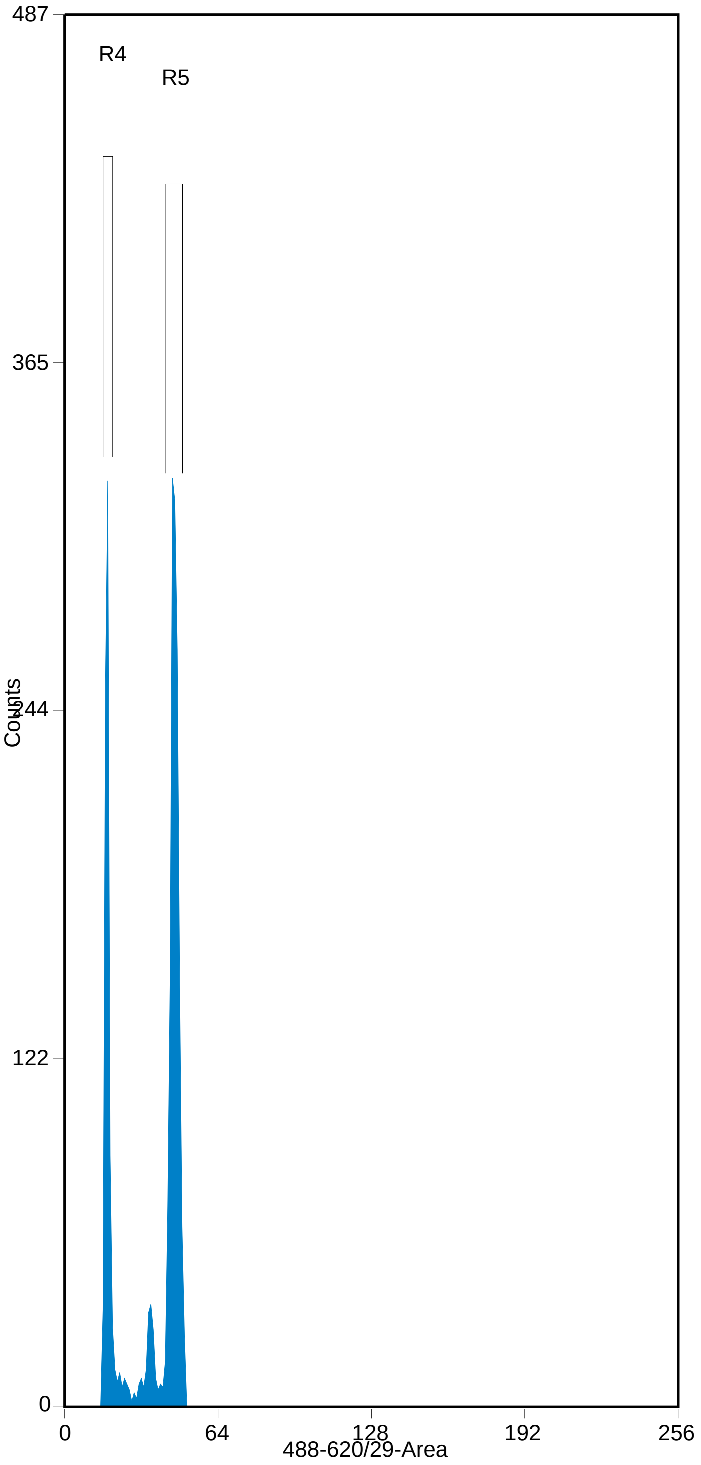

spiderling of AmC3_13

Region	Count	% Hist	Mean	CV
Total	2346	100.00	35.82	36.21
R4	727	30.99	17.76	4.88
R5	1354	57.72	45.89	3.36

BECKMAN COULTER

spiderling of AmC3_14

ADN_31_Jul_23_20 (G3: R1 & R2)

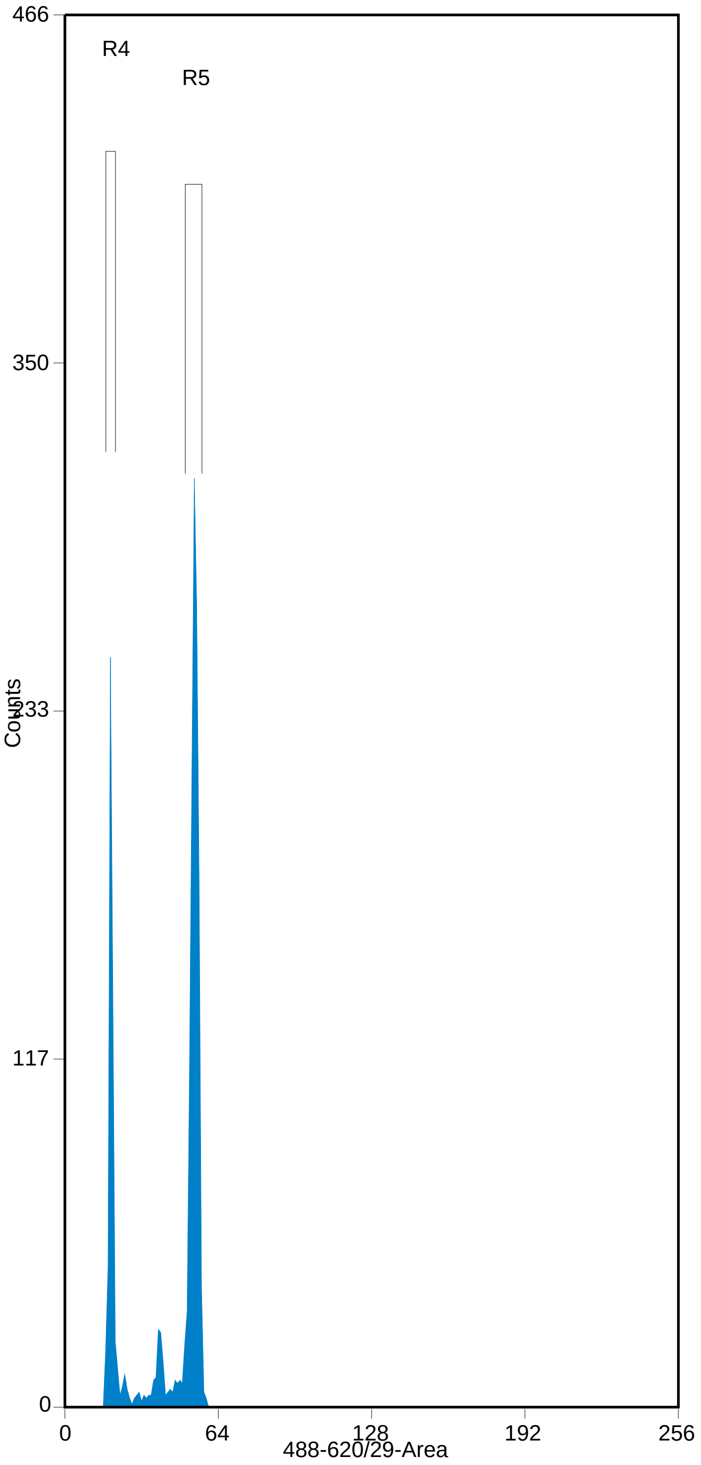

Region	Count	% Hist	Mean	CV
Total	1873	100.00	43.26	35.50
R4	478	25.52	19.21	4.33
R5	1174	62.68	54.11	2.75

spiderling of AmC3_15

ADN_31_Jul_23_21 (G3: R1 & R2)

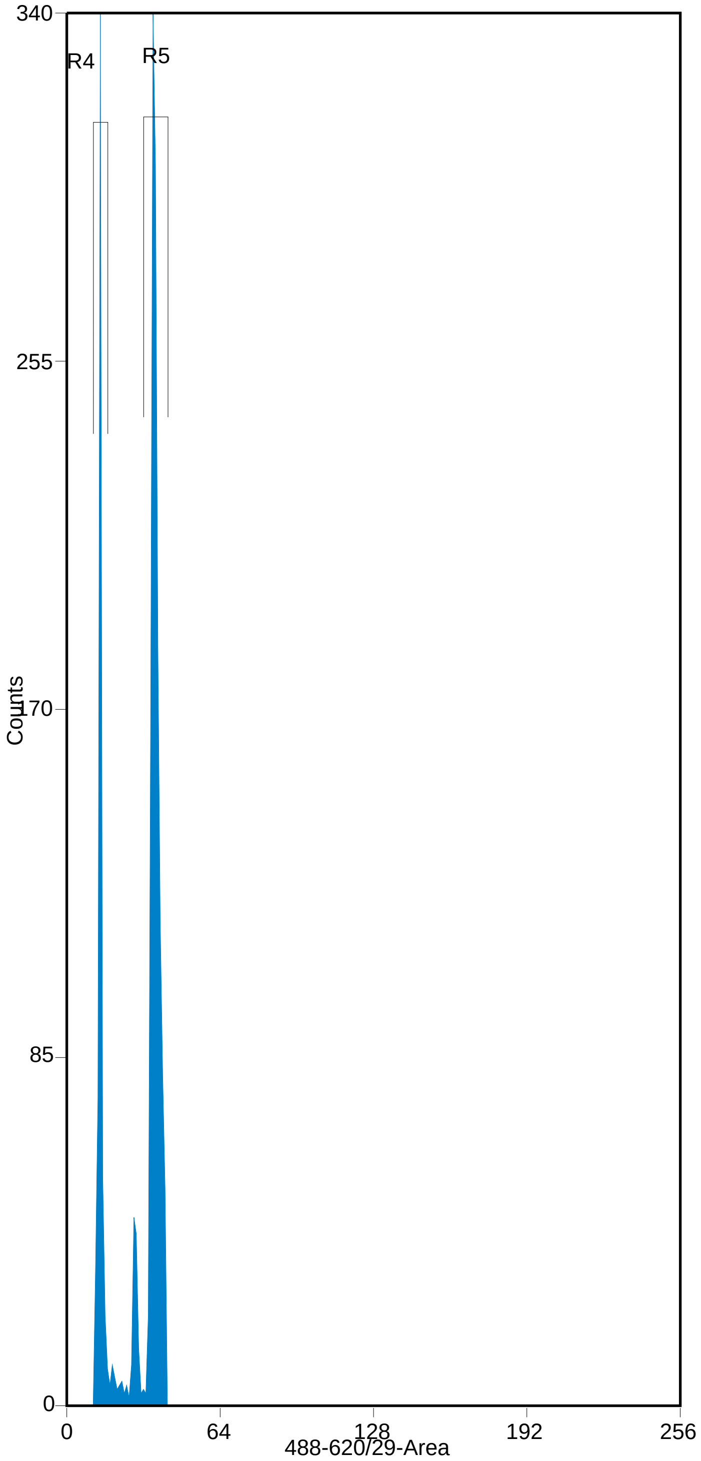

Region	Count	% Hist	Mean	CV
Total	2150	100.00	29.53	35.40
R4	624	29.02	13.98	5.95
R5	1364	63.44	37.01	4.43
